# Understanding the consequences of education inequality on cardiovascular disease: mendelian randomisation study

**DOI:** 10.1136/bmj.l1855

**Published:** 2019-05-22

**Authors:** Alice R Carter, Dipender Gill, Neil M Davies, Amy E Taylor, Taavi Tillmann, Julien Vaucher, Robyn E Wootton, Marcus R Munafò, Gibran Hemani, Rainer Malik, Sudha Seshadri, Daniel Woo, Stephen Burgess, George Davey Smith, Michael V Holmes, Ioanna Tzoulaki, Laura D Howe, Abbas Dehghan

**Affiliations:** 1 MRC Integrative Epidemiology Unit, University of Bristol, Bristol, UK; 2 Population Health Sciences, Bristol Medical School, University of Bristol, Oakfield House, Oakfield Grove, Bristol, BS8 2BN, UK; 3 Department of Biostatistics and Epidemiology, School of Public Health, Imperial College London, London, UK; 4 National Institute for Health Research Biomedical Research Centre at the University Hospitals Bristol NHS Foundation Trust and the University of Bristol, Bristol, UK; 5 Institute for Global Health, University College London, London, UK; 6 Lausanne University Hospital, Lausanne, Switzerland; 7 Faculty of Biology and Medicine, University of Lausanne, Lausanne, Switzerland; 8 School of Experimental Psychology, University of Bristol, Bristol, UK; 9 UK Centre for Tobacco and Alcohol Studies, School of Psychological Science, University of Bristol, Bristol, UK; 10 Institute for Stroke and Dementia Research, University Hospital of Ludwig-Maximilians University, Munich, Germany; 11 Glenn Biggs Institute for Alzheimer’s & Neurodegenerative Diseases, University of Texas Health Sciences Centre, San Antonio, TX, USA; 12 University of Texas Health Sciences Center, San Antonio, TX, USA; 13 Department of Neurology, Boston University School of Medicine, Boston, MA, USA; 14 The Framingham Heart Study, Framingham, MA, USA; 15 Department of Neurology and Rehabilitation Medicine, University of Cincinnati College of Medicine, Cincinnati, OH, USA; 16 MRC Biostatistics Unit, University of Cambridge, Cambridge, UK; 17 Department of Public Health and Primary Care, University of Cambridge, Cambridge, UK; 18 MRC Population Health Research Unit at the University of Oxford, Oxford, UK; 19 Clinical Trial Service Unit and Epidemiological Studies Unit (CTSU), Nuffield Department of Population Health, University of Oxford, Oxford, UK; 20 National Institute for Health Research, Oxford Biomedical Research Centre, Oxford University Hospital, Oxford, UK; 21 MRC-PHE Centre for Environment, School of Public Health, Imperial College London, London, UK; 22 Department of Hygiene and Epidemiology, University of Ioannina Medical School, Ioannina, Greece

## Abstract

**Objectives:**

To investigate the role of body mass index (BMI), systolic blood pressure, and smoking behaviour in explaining the effect of education on the risk of cardiovascular disease outcomes.

**Design:**

Mendelian randomisation study.

**Setting:**

UK Biobank and international genome-wide association study data.

**Participants:**

Predominantly participants of European ancestry.

**Exposure:**

Educational attainment, BMI, systolic blood pressure, and smoking behaviour in observational analysis, and randomly allocated genetic variants to instrument these traits in mendelian randomisation.

**Main outcomes measure:**

The risk of coronary heart disease, stroke, myocardial infarction, and cardiovascular disease (all subtypes; all measured in odds ratio), and the degree to which this is mediated through BMI, systolic blood pressure, and smoking behaviour respectively.

**Results:**

Each additional standard deviation of education (3.6 years) was associated with a 13% lower risk of coronary heart disease (odds ratio 0.86, 95% confidence interval 0.84 to 0.89) in observational analysis and a 37% lower risk (0.63, 0.60 to 0.67) in mendelian randomisation analysis. As a proportion of the total risk reduction, BMI was estimated to mediate 15% (95% confidence interval 13% to 17%) and 18% (14% to 23%) in the observational and mendelian randomisation estimates, respectively. Corresponding estimates were 11% (9% to 13%) and 21% (15% to 27%) for systolic blood pressure and 19% (15% to 22%) and 34% (17% to 50%) for smoking behaviour. All three risk factors combined were estimated to mediate 42% (36% to 48%) and 36% (5% to 68%) of the effect of education on coronary heart disease in observational and mendelian randomisation analyses, respectively. Similar results were obtained when investigating the risk of stroke, myocardial infarction, and cardiovascular disease.

**Conclusions:**

BMI, systolic blood pressure, and smoking behaviour mediate a substantial proportion of the protective effect of education on the risk of cardiovascular outcomes and intervening on these would lead to reductions in cases of cardiovascular disease attributable to lower levels of education. However, more than half of the protective effect of education remains unexplained and requires further investigation.

## Introduction

Cardiovascular disease is the leading cause of mortality worldwide, accounting for over 17 million deaths annually.^1^ Recent studies suggest that socioeconomic risk factors, such as education, play a causal role in the aetiology of cardiovascular disease.^2^ ^3^ ^4^ Tillmann and colleagues found that an additional 3.6 years of education reduced the risk of coronary heart disease by approximately one third.^2^ However, educational opportunities are not equitable throughout populations and education is inherently difficult to intervene on. Therefore, understanding the risk factors that might be driving the adverse later life outcomes associated with lower levels of education would provide the opportunity to reduce inequalities with interventions.

Existing studies suggest that body mass index (BMI), systolic blood pressure, and smoking behaviour partly explain differences in the risk of cardiovascular disease related to educational attainment.^5^ ^6^ ^7^ However, these studies have relied on observational mediation analyses that might suffer from biases. Traditional methods use one snapshot of a risk factor, which could incompletely capture a person’s lifetime exposure.^8^ For example, systolic****blood pressure measured at one time point will suffer from measurement error due to day-to-day fluctuations and will not capture changes across the life course. This measurement error can lead to an underestimation of mediation.^8^ Furthermore, other biases such as unmeasured confounding cannot be addressed by using observational methods.^9^

Mendelian randomisation uses genetic variants as instruments to estimate the effect of an exposure on an outcome of interest,^10^ exploiting the random allocation of genetic variants to infer causal effects that are robust to non-differential measurement error and confounding.^10^ Two-step mendelian randomisation for mediation analysis, unlike traditional observational mediation analysis approaches, is both sensitive to the causal effects of the mediator and corrects for its measurement error.^11^ Recent genome-wide association study meta-analyses have identified a number of genetic variants for educational attainment that could be used as instrumental variables.^12^ ^13^

Mendelian randomisation has previously been used to show the causal effects of education on BMI, systolic blood pressure, and smoking behaviour and also the effects of BMI and smoking behaviour on cardiovascular disease.^14^ ^15^ ^16^ ^17^ ^18^ ^19^ Although the results from these studies suggest that BMI, systolic blood pressure, and smoking behaviour are likely to explain some of the protective mechanisms of education on cardiovascular disease, they alone do not quantify the mediated effect. In this study, we investigated the role of BMI, systolic blood pressure, and lifetime smoking behaviour in mediating the causal effect of educational attainment on the risk of cardiovascular disease by using three complementary approaches: multivariable regression, one-sample mendelian randomisation, and two-sample mendelian randomisation. BMI, systolic blood pressure, and smoking behaviour were selected as intermediate risk factors based on the literature, implicating them as both being affected by education and as risk factors for cardiovascular disease, with availability of data across all three complementary methods. We consider the three risk factors both individually and simultaneously. Understanding the mechanisms by which education affects cardiovascular health could have powerful applications for public health policy. For this, it is important to understand the population-level implications of changes to BMI, systolic blood pressure, and smoking behaviour on inequalities in the risk of cardiovascular disease.

## Methods

### Overall study design

This study used multivariable regression of observational data, one-sample mendelian randomisation of individual level genetic data, and two-sample mendelian randomisation of summary level genetic data to investigate whether BMI, systolic blood pressure, and lifetime smoking behaviour explain the protective effect of education on the risk of coronary heart disease, stroke, myocardial infarction, and cardiovascular disease (all subtypes).

### Data sources

#### UK Biobank

The UK Biobank recruited 503 317 British adults between 2006 and 2010. Participants attended assessment centres involving questionnaires, interviews, anthropometric, physical, and genetic measurements.^20^ ^21^ In the observational analysis, we included 217 013 white British participants, with complete data on genotypes, age, sex, educational attainment, cardiovascular outcomes, BMI, systolic blood pressure, smoking behaviour, socioeconomic status (as measured by Townsend Deprivation Index at birth), and place of birth. Supplementary figure 1 shows the exclusion criteria for the main UK Biobank analyses, and supplementary figure 2 shows the exclusion criteria for the genome-wide association studies carried out for systolic blood pressure and smoking. White British participants were defined by using both self-reported questionnaire data and similar genetic ancestry to the European ancestry principal components computed from the 1000 genomes project.^22^

The supplementary methods show the data on educational attainment (as measure by highest achieved qualifications), BMI, systolic blood pressure, smoking behaviour, and all covariates. A continuous lifetime measure of smoking behaviour, incorporating smoking initiation, duration, heaviness, and cessation is used in this analysis. Full details are presented elsewhere.^23^ Participants reported their highest qualification and age of leaving school if they did not have a degree. These were converted to the International Standard Classification for Education coding of educational attainment (supplementary table 1).^13^ We used available follow-up data where baseline data were missing. To account for the effects of antihypertensive treatment, participants who were taking antihypertensive drugs had 10 mm Hg added to their measured systolic blood pressure.^24^ Supplementary table 2 shows that cardiovascular disease diagnoses (including diagnoses of coronary heart disease, stroke, and myocardial infarction) and events were ascertained through linkage mortality data and hospital episode statistics, with cases defined according to ICD-9 (international classification of diseases, ninth revision) and ICD-10 codes (international classification of diseases, 10th revision).^25^ We excluded participants who had experienced a cardiovascular disease event before the baseline assessment (prevalent cases) and we only considered first event, incident cases after the assessment centre.

#### Genome-wide association study meta-analyses

In the two-sample mendelian randomisation analysis, we obtained summary genetic associations from genome-wide association study data for each respective phenotype. For education, this was the Social Science Genetic Association Consortium genome-wide association study meta-analysis of years of schooling in 1 131 881 participants of European ancestry,^13^ with summary data made available for 766 345 of these participants. Instruments were selected as the 1271 independent (pairwise r^2^<0.1) genome-wide-wide significant single nucleotide polymorphisms after analysing data from the full sample.^13^ We obtained genetic estimates for BMI from the Genetic Investigation of Anthropometric Traits consortium’s 2018 genome-wide association study meta-analysis of 681 275 participants of European decent.^26^ Genetic association estimates for systolic blood pressure and smoking behaviour were estimated from a genome-wide association study of 318 147 white British participants in the UK Biobank (see supplementary methods). Instruments for BMI, systolic blood pressure, and smoking behaviour were identified as the lead single nucleotide polymorphisms in loci reaching genome-wide significance after clumping summary estimates from the largest available genome-wide association study for linkage disequilibrium threshold r^2^<0.001 and distance >10 000 kb, by using a 1000 genomes European reference panel through the TwoSampleMR package (default settings of the ‘clump_data’ command) in the statistical software R.^27^ For coronary heart disease, we used publicly available genetic association estimates from the CARDIoGRAMplusC4D 1000 Genomes-based genome-wide association study meta-analysis of 60 801 cases and 123 504 controls.^28^ The definition for coronary heart disease was broad and inclusive, considering acute coronary syndrome, myocardial infarction, angina with one or angiographic stenoses of greater than 50%, and chronic stable angina. Details of the genome-wide association study for stroke and myocardial infarction, and further details for other genome-wide association study are provided in the supplementary methods. All genetic association estimates used in each two-sample mendelian randomisation analyses are provided in supplementary tables 4 to 18.

### Statistical analysis

#### Effect of education on cardiovascular disease

In observational analysis of UK Biobank data, we used multivariable logistic regression to estimate the association of education with cardiovascular disease and its subtypes. We adjusted all analyses using UK Biobank data for potential confounders: age, sex, place of birth, birth distance from London, and Townsend Deprivation Index at birth. These confounders were determined a priori, with place of birth and birth distance from London included to control for population structure in the UK Biobank.^3^ ^29^

In two-sample mendelian randomisation analysis, the effects of education on cardiovascular subtypes were investigated using ratio method mendelian randomisation with standard errors derived using the delta method.^30^ We used fixed-effect inverse-variance weighted meta-analysis to pool mendelian randomisation estimates across individual single nucleotide polymorphisms.^31^

#### Mediation by BMI, systolic blood pressure, and smoking behaviour

In multivariable observational and one-sample mendelian randomisation analyses, when investigating the degree to which the effects of education on cardiovascular disease and its subtypes are mediated through each risk factor (BMI, systolic blood pressure, and smoking behaviour) individually, we used the product of coefficients method to estimate the indirect effect (that is, the effect of education on cardiovascular disease that goes through the risk factor).^32^ This involved first estimating the effect of education on each risk factor individually, then multiplying this with the effect of that risk factor on the outcome after adjusting for education. We estimated the proportion of the overall effect of education on cardiovascular disease subtypes that was mediated by each risk factor by dividing the indirect effect by the total effect. Standard errors were derived by bootstrapping in the observational and one-sample mendelian randomisation analysis and by using the delta method in the two-sample mendelian randomisation analysis.

We used multivariable linear regression in the observational analysis to estimate the association of education with each risk factor after adjusting for confounders (as in the total effects models). We then estimated the effect of each risk factor on the individual cardiovascular disease subtypes with additional adjustment for self-reported educational attainment.^33^ The two estimates were multiplied together to estimate the indirect effect (of education, through the risk factor).

We used the inverse-variance weighted mendelian randomisation approach for the two-sample mendelian randomisation to estimate the effect of education on each risk factor. We used regression-based multivariable mendelian randomisation to estimate the effect of each risk factor on the risk of the considered cardiovascular disease subtypes, adjusting for genetic effect of the instruments on education.^34^ We estimated the indirect effect of education on the risk of each cardiovascular disease subtype through the considered risk factor by multiplying results from these two mendelian randomisation analyses.

Methods for investigating the role of all three risk factors together are presented in the supplementary methods.

#### One-sample mendelian randomisation and sensitivity analyses

One-sample mendelian randomisation analysis was carried out in the UK Biobank. Full details of the genetic variants used are in the supplementary methods and supplementary tables 19 to 21. In brief, a weighted allele score was created based on genome-wide significant single nucleotide polymorphisms identified in recent genome-wide association study meta-analyses for the considered exposures (that is, educational attainment, BMI, systolic blood pressure, and smoking behaviour).^12^ ^23^ ^35^ These genome-wide association study estimates were selected from studies that did not include UK Biobank participants, so as to avoid participant overlap, and therefore, in some cases, the genome-wide association study and subsequent instruments differed from the genome-wide association study studies used for the two-sample mendelian randomisation described previously. Table 1 shows a summary of all phenotypes and genome-wide association study data used. The variants in each instrument were harmonised for consistent directions of association and each single nucleotide polymorphism in the genetic score was weighted by its relative effect size in the respective genome-wide association study, with effects combined in an additive model. Analyses using the UK Biobank were replicated using the risk difference scale. A range of sensitivity analyses were carried out, including exploring the assumption of no pleiotropy within mendelian randomisation analyses by using mendelian randomisation-Egger and weighted median mendelian randomisation.^34^ ^36^ One-sample mendelian randomisation analyses and multivariable observational associations between education and cardiovascular disease were repeated after stratifying for sex and age below or above the median (39-57 years compared with 58-72 years). On a subsample of participants with dietary recall data, an observational model was run considering diet and exercise as mediators in addition to BMI, systolic blood pressure, and smoking behaviour mediators. Full details of sensitivity analyses are provided in the supplementary methods.

**Table 1:** Summary of phenotypes and genome-wide association study (GWAS) data used as instrumental variables across analyses

Exposure	Multivariable observational analysis*	Mendelian randomisation	
		One-sample	Two-sample
Educational attainment	Self-reported highest qualification mapped to ISCED years of schooling	Weighted allele score, using genome-wide significance SNPs (n=74) and β weights from Okbay and colleagues*,* 2016 58	Individual SNPs from Lee and colleagues, 2018 13 (n=1271)
BMI	Measured weight and height	Weighted allele score, using genome-wide significance SNPs (n=77) and β weights from Locke and colleagues*,* 2015 35	Individual SNPs from Yengo and colleagues, 2018 26 (n=360)
Systolic blood pressure	Median of two automated blood pressure measurements	Weighted allele score, using genome-wide significance SNPs (n=65 and 55 sample 1 and 2 respectively) from a split sample GWAS in the UK Biobank†	Individual SNPs from SBP GWAS carried out as part of this work on full UK Biobank sample† (n=191)
Smoking behaviour	Estimate of lifetime smoking behaviour using self-report data	Weighted allele score, using genome-wide significance SNPs (n=18 and 15 sample 1 and 2 respectively) from a split sample GWAS in UK Biobank†^23^	Individual SNPs from Wootton and colleagues, 2018 using full UK Biobank sample 23 (n=126)

* All in the UK Biobank.

† Full methods in the supplementary material.

ISCED=International Standard Classification for Education; SNPs=single nucleotide polymorphisms; GWAS=genome-wide association study

#### Statistical software and ethical approval

We performed the analysis by using Stata version 14 (StataCorp LP) and R version 3.4.3 (The R Foundation for Statistical Computing). We used the mrrobust package for Stata and the TwoSample MR package for R to facilitate MR analyses.^27^ ^37^

### Patient and public involvement

No patients or participants were involved in setting the research question or the outcome measures, nor were they involved in developing plans for design or implementation of the study. No patients were asked to advise on interpretation or writing up of results. There are no plans to disseminate the results of the research to study participants or the relevant patient community.

## Results

### UK Biobank cohort description

The UK Biobank sample used in the observational and one-sample mendelian randomisation analysis was comparable to the participants in UK Biobank as a whole, although the UK Biobank is not representative of the wider UK population (participants are typically more educated and of a higher socioeconomic status as compared with the general population).^21^ In the analysis sample, 38% of participants had over 19 years of education, which is equivalent to a vocational qualification or degree and only 17% of participants left school with no formal qualifications after seven years (supplementary table 3). The standard deviation of educational attainment was 3.6 years, BMI was 4.69, and systolic blood pressure was 18.68 mm Hg. For lifetime smoking behaviour, one standard deviation increase is equivalent to, for example, an individual smoking 20 cigarettes a day for 15 years and stopping 17 years ago, or an individual smoking 60 cigarettes a day for 13 years and stopping 22 years ago.^23^

### Effect of education on the risk of coronary heart disease and stroke

**Fig 1:**
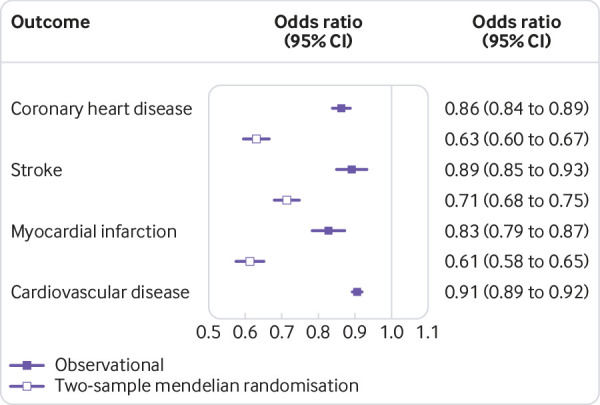
The effect of a one standard deviation increase in education on the risk of cardiovascular disease outcomes

Figure 1 shows that in observational analyses, one standard deviation of higher education was associated with a 14% lower risk of coronary heart disease with an odds ratio of 0.86 (95% confidence interval 0.84 to 0.89). Two-sample mendelian randomisation analysis indicated a stronger protective effect with an odds ratio of 0.63 (0.60 to 0.67).

Figure 1 shows that similar protective associations were found for the effect of education on other cardiovascular disease outcomes. In observational analyses, a one standard deviation higher education was associated with an 11% lower risk of stroke, with an odds ratio of 0.89 (95% confidence interval 0.85 to 0.93). In two-sample mendelian randomisation analyses the protective effect was stronger, with an odds ratio of 0.71 (0.68 to 0.75).

All three approaches also provided consistent evidence for a protective effect of education with risk of cardiovascular disease and its subtypes (supplementary figures 6 and 7).

### Effect of education on BMI, systolic blood pressure, and smoking behaviour

**Fig 2:**
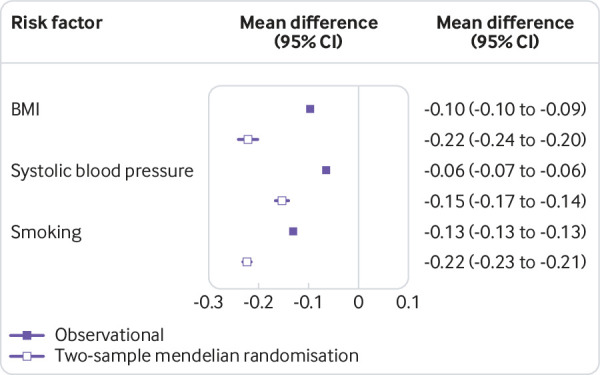
Estimates for the association between one standard deviation higher education and intermediate risk factors

Figure 2 and supplementary figures 8 to 11 show that in all methods, a longer time in education was associated with lower BMI, systolic blood pressure, and smoking behaviour.

### Effect of BMI, systolic blood pressure, and smoking behaviour on the risk of cardiovascular disease outcomes

**Fig 3:**
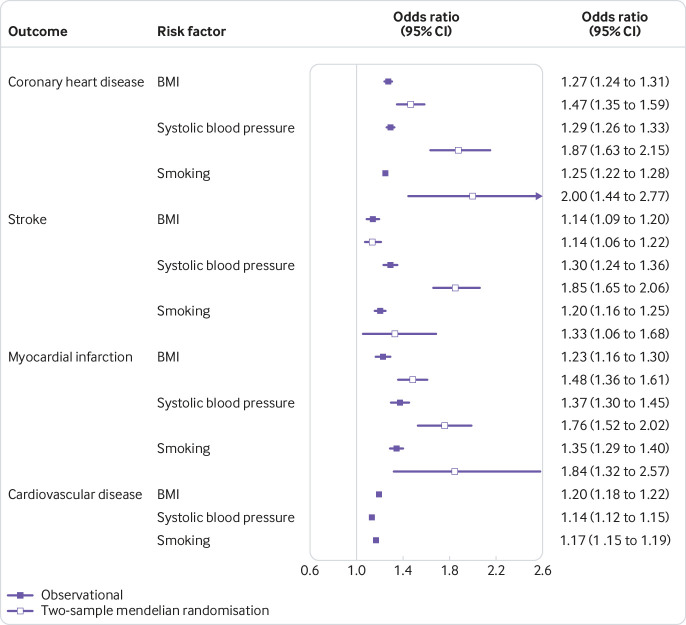
Associations of a one standard deviation higher risk factor on the risk of cardiovascular disease outcomes

Figure 3 shows that both observational and two-sample mendelian randomisation analyses consistently supported an increased risk of coronary heart disease with higher BMI, systolic blood pressure, and smoking behaviour, after adjusting for education. The effect of each risk factor was less consistent in the one-sample mendelian randomisation and estimates had wide confidence intervals (supplementary figure 12).

### Mediation by BMI, systolic blood pressure, and smoking behaviour

**Fig 4:**
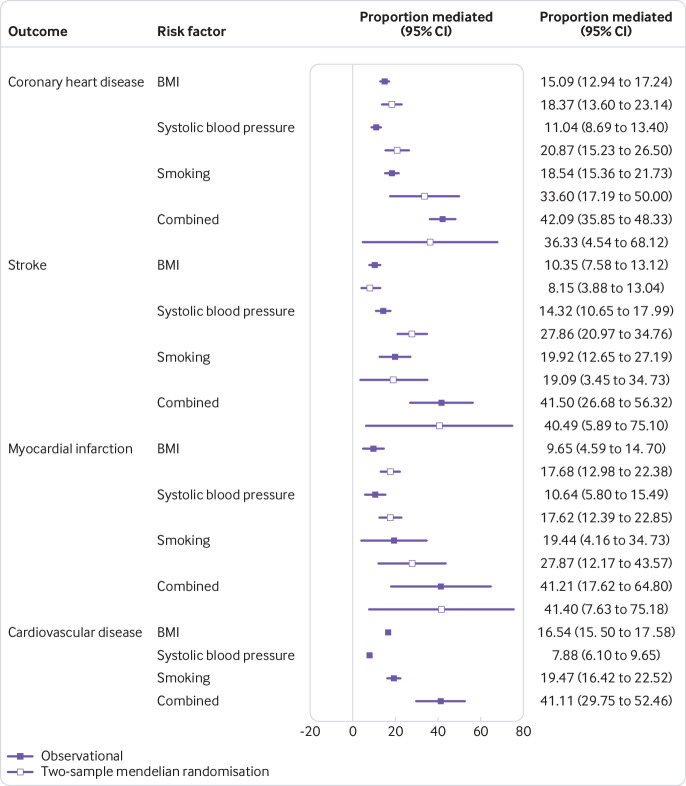
Estimates for the effect of education on cardiovascular disease outcomes explained by the risk factors. Combined estimates refer to the effect of BMI, systolic blood pressure, and smoking behaviour considered together in one model

Figure 4 shows that in the observational analysis, the proportion of the effect of education on the risk of coronary heart disease mediated by BMI was 15% (95% confidence interval 13% to 17%), 11% for systolic blood pressure (9% to 13%), and 19% for smoking behaviour (15% to 22%). In the two-sample mendelian randomisation analysis, the percentage mediated by BMI was 18% (14% to 23%), 21% for systolic blood pressure (15% to 26%), and 34% for smoking behaviour (95% confidence interval 17% to 50%). In observational analyses, combining all three risk factors explained 42% (36% to 48%) of the effect of education on the risk of coronary heart disease. In two-sample mendelian randomisation, combining all three risk factors explained 36% (5% to 68%) of the effect of education on coronary heart disease.

Similar results were found for other cardiovascular disease subtypes in multivariable observational analyses. Smoking behaviour consistently mediated around 20% of the association. BMI explained between 10% and 17% of the association between education and cardiovascular disease and its subtypes; systolic blood pressure explained between 8% and 18% of the association. In two-sample mendelian randomisation analyses, smoking behaviour explained up to 34% of the association between education and cardiovascular disease subtypes; BMI estimated up to 18% and systolic blood pressure up to 28% of the association. One-sample mendelian randomisation analyses estimated similar amounts of the association explained by systolic blood pressure and smoking behaviour but was less consistent for BMI (supplementary figure 13).

### Sensitivity analyses

Table 2 and supplementary figures 14 to 17 show that results from sensitivity analyses were comparable, but produced less precise estimates with wider confidence intervals. Unadjusted and age and sex adjusted models were also consistent with the main fully adjusted models for multivariable analyses (supplementary tables 22 and 23). Analyses stratified by age and separately by sex were consistent with the non-stratified main results, although confidence intervals were wide in the mendelian randomisation (supplementary figures 14 and 15). The effects of each mediator individually, and combined, estimated on the risk difference scale and by using the difference method in individual data were consistent with the main analyses on the log odds ratio scale (supplementary figure 16). Including diet and exercise measures in addition to BMI, systolic blood pressure, and smoking behaviour did not change the amount of the association that education to cardiovascular disease (all subtypes) explained (supplementary figure 17).

**Table 2:** Mendelian randomisation (MR) sensitivity analyses

Analysis	Two-sample			One-sample	
	Estimate (95% CI)	P value		Estimate (95% CI)	P value
**Education-coronary heart disease**					
IVW	0.63 (0.60 to 0.67)	<0.001		0.51 (0.26 to 1.00)	0.05
MR-Egger	0.68 (0.54 to 0.85)	0.001		0.54 (0.12 to 2.34)	0.41
MR-Egger intercept	NA	0.37		NA	0.93
Weighted median	0.62 (0.57 to 0.67)	<0.001		0.98 (0.96 to 0.99)	0.33
**Education-stroke**					
IVW	0.71 (0.68 to 0.75)	<0.001		0.46 (0.30 to 0.71)	<0.001
MR-Egger	0.72 (0.60 to 0.87)	0.001		0.57 (0.22 to 1.47)	0.25
MR-Egger intercept	NA	0.76		NA	0.60
Weighted median	0.71 (0.66 to 0.76)	<0.001		0.99 (0.98 to 1.00)	0.002
**Education-myocardial infarction**					
IVW	0.61 (0.58 to 0.65)	<0.001		0.18 (0.08 to 0.38)	<0.001
MR-Egger	0.67 (0.52 to 0.85)	0.001		0.20 (0.04 to 1.03)	0.05
MR-Egger intercept	NA	0.39		NA	0.88
Weighted median	0.59 (0.54 to 0.65)	<0.001		0.99 (0.98 to 1.00)	0.002
**Education-cardiovascular disease**					
IVW	NA	NA		0.64 (0.51 to 0.82)	<0.001
MR-Egger	NA	NA		0.57 (0.34 to 0.95)	0.03
MR-Egger intercept	NA	NA		NA	0.59
Weighted median	NA	NA		0.96 (0.94 to 0.98)	<0.001
**Education-BMI**					
IVW	−0.22 (−0.24 to −0.20)	<0.001		−0.36 (−0.49 to −0.23)	<0.001
MR-Egger	−0.28 (−0.49 to −0.07)	0.009		−0.15 (−0.41 to 0.12)	0.29
MR-Egger intercept	NA	0.99		NA	0.08
Weighted median	−0.27 (−0.30 to −0.23)	<0.001		−0.51 (−0.62 to −0.39)	<0.001
**Education-systolic blood pressure **					
IVW	−0.15 (−0.17 to −0.14)	<0.001		−0.14 (−0.24 to −0.04)	0.005
MR-Egger	−0.13 (−0.21 to −0.05)	0.002		−0.10 (−0.30 to 0.11)	0.37
MR-Egger intercept	NA	0.32		NA	0.65
Weighted median	−0.18 (−0.21 to −0.16)	<0.001		−0.12 (−0.21 to −0.03)	0.008
**Education-smoking**					
IVW	−0.32 (−0.33 to −0.31)	<0.001		−0.37 (−0.47 to −0.28)	<0.001
MR-Egger	−0.29 (−0.36 to −0.22)	<0.001		−0.40 (−0.60 to −0.20)	<0.001
MR-Egger intercept	NA	NA		NA	0.73
Weighted median	−0.35 (−0.37 to −0.33)	<0.001		−0.37 (−0.46 to −0.29)	<0.001

IVW=inverse-variance weighted; NA=not applicable

Coronary heart disease, stroke, and myocardial infarction are in odds ratio units. BMI, systolic blood pressure, and smoking behaviour are in standard deviation units. In one-sample analyses, the weighted median was estimated on the risk difference scale and converted to odds ratio by using linear combinations.

## Discussion

Our observational and genetic analyses support that the effect of education on the risk of cardiovascular disease is mediated by approximately up to one third through any of BMI, systolic blood pressure, or smoking behaviour. When investigating all three risk factors together, it was estimated that around 40% of the association between education and cardiovascular disease is explained by the three risk factors combined, both in observational and mendelian randomisation analyses. Note that over half of the effects of education remain unexplained in these analyses. Our main analysis did not consider the contributions of exercise, diet, health system factors, lipid profile, and glycaemic traits.^38^ ^39^ ^40^ ^41^ ^42^ ^43^ ^44^ However, these risk factors are likely to be inter-related with the risk factors already considered in our analysis. For example, much of the effect of diet and activity on cardiovascular disease is likely to act through BMI and systolic blood pressure, and therefore the cumulative effect of BMI, systolic blood pressure, and smoking behaviour together is likely to be capturing some of their effects. Indeed, in a sensitivity analysis including diet and exercise alongside BMI, systolic blood pressure, and smoking behaviour, they explain no more of the association between education and cardiovascular disease than the three mediators in our main analysis.

We have triangulated evidence across three distinct approaches. Although the point estimates vary, along with the mediation results, all three approaches indicate the same conclusions. Our mendelian randomisation estimates are much larger in magnitude than the observational results. In mendelian randomisation, the genetic instruments used to proxy the exposure and mediators estimate their lifetime effect, rather than one snapshot, which could explain the larger estimates in mendelian randomisation. Additionally, this could be owing to bias from negative confounding or measurement error in multivariable analyses. Cases recruited to the case-control studies included in two-sample analyses might represent a more extreme phenotype than in cohort studies such as the UK Biobank. The two-sample mendelian randomisation estimates are more precise than the one-sample mendelian randomisation results from the UK Biobank, likely related to the larger sample sizes and the number of cases.

### Findings in context

Mendelian randomisation studies have previously investigated the causal effects of education on coronary heart disease, BMI, systolic blood pressure, and smoking behaviour,^2^ ^14^ ^15^ ^16^ with others further estimating the effects of BMI and smoking behaviour on cardiovascular disease.^18^ ^19^ Our current study makes several notable advances. We have used the most recent genome-wide association study of educational attainment to optimise the power of our two-sample mendelian randomisation analysis. With the larger sample size, the instruments selected from this study explained approximately 12% of the variance in education, as compared with the 3% accounted for in the previous iteration.^12^ ^13^ Similarly, by leveraging the power of the UK Biobank and recent large-scale genome-wide association study meta-analyses, we were able to study additional cardiovascular outcomes, including stroke and myocardial infarction. In addition to the overall effects of the considered risk factors on cardiovascular disease, we were able to estimate the proportion of the effect of education that they mediate by using network (or two-step) mendelian randomisation, a recently developed method.^11^ ^32^ Genetic instruments for smoking behaviour are limited and are typically related to binary measures that would introduce severe bias in mendelian randomisation.^45^ The development of a genome-wide association study for the continuous measure of lifetime smoking behaviour has allowed us to include this in a mediation model.^23^

Several studies have used observational multivariable regression methods to support mediating roles of BMI, systolic blood pressure, and smoking behaviour in the pathway between education and the risk of cardiovascular disease,^5^ ^6^ ^46^ ^47^ with consistent results obtained by using various measures of education, including time spent in schooling and academic qualifications. In an analysis of Dutch participants, Kershaw and colleagues attributed almost 27% of the association between education and coronary heart disease to smoking behaviour, with 10% and 5% attributed to obesity and hypertension respectively.^46^ Similarly, Dégano and colleagues found 7% and 14% of the association between education and cardiovascular disease could be explained by BMI and hypertension respectively.^6^ However, they did not find any evidence of smoking behaviour mediating the association. Veronesi and colleagues analysed their data stratified by sex, but consistently found mediating effects of systolic blood pressure and smoking behaviour in both men and women.^47^ The findings in our study show that observational estimates underestimate the mediating role of smoking behaviour, BMI, and systolic blood pressure compared with mendelian randomisation, likely due to measurement error in the mediators that bias observational estimates towards the null, but do not affect mendelian randomisation estimates to the same degree.^11^ Given the importance of measurement error as a source of bias in mediation analysis,^8^ the mendelian randomisation approach offers favourable opportunities for understanding mediation.

### Strengths and limitations

The major strength of our work is that it allowed for assessment of the causal role of mediators by using mendelian randomisation, an approach that is robust to non-differential measurement error in the mediator. We have used multiple data sources and approaches, each with different potential sources of biases, to thus improve the reliability of our findings through triangulation.^48^ Furthermore, the mediated effects estimated were consistent across the two approaches and in the statistical sensitivity analyses. The imprecision in the one-sample mendelian randomisation analysis showed the need for very large sample sizes to achieve enough statistical power when estimating mediation in a mendelian randomisation framework. The results were complemented by the two-sample mendelian randomisation approach, which had greater statistical power, but might be susceptible to alternative sources of bias, including those related to participant overlap in the samples used to obtain genetic association estimates for the exposures and outcomes.^49^ Existing genome-wide association study meta-analyses of systolic blood pressure have adjusted for BMI as a covariate, which could introduce collider bias,^50^ ^51^ and for this reason we performed a genome-wide association study of systolic blood pressure in the UK Biobank to select instruments, without adjusting for BMI. We also applied a ‘split sample’ systolic blood pressure genome-wide association study approach on unrelated individuals in the UK Biobank for use in individual-level data (one-sample) mendelian randomisation to avoid overlapping populations in the genetic association estimates for the exposure and outcome,^52^ and any associated bias.^49^ ^53^ To this end, the one-sample mendelian randomisation entirely avoided any population overlap when obtaining genetic estimates for the exposures and the outcomes.

For all cardiovascular disease subtypes and individual risk factors considered, the largest effects of education were consistently seen with the mendelian randomisation approaches, with smaller effects seen in the analysis of observational data. Measurement error in a mediator leads to an underestimation of the proportion mediated, so the discrepancy between our observational and mendelian randomisation analyses could be attributable to mendelian randomisation analyses suffering less bias from measurement error.^8^ BMI is accurately measured and has little daily variation—and correspondingly the estimates of the proportion of effect mediated by BMI in the observational and mendelian randomisation analyses are similar (15% and 18% respectively). In contrast, systolic blood pressure and lifetime smoking behaviour are difficult to measure accurately—and the estimated proportion mediated is smaller in the observational analysis than the mendelian randomisation (11% and 1% respectively for systolic blood pressure, and 19% and 34% respectively for smoking behaviour). Measurement error could also be introduced by participants over-reporting traits perceived to be ‘desirable’ such as education and underreporting traits perceived to be ‘undesirable’ such as smoking behaviour.^54^ The estimates for all three risk factors together were more similar between observational and mendelian randomisation estimates, although for all models, the confidence intervals were wide. Note that although mendelian randomisation is more robust to measurement error, the instruments might not necessarily be capturing all aspects of the exposure phenotype under consideration. For example, the instruments for systolic blood pressure capture average systolic blood pressure but might not necessarily reflect the variability in blood pressure.

Estimates from mendelian randomisation analyses are robust to reverse causation bias, owing to the random allocation of genetic instruments at conception (and thus before development of the outcome under consideration). Although Tyrrell and colleagues have previously used mendelian randomisation to suggest an effect of BMI on education,^55^ and the relationship between education and BMI might be bi-directional, in our analyses where we focused on one direction of effect (that from education to BMI) the use of a large number of strong instruments for education makes it unlikely that the effect estimates derived from mendelian randomisation are biased due to reverse causation.

Another limitation of the mendelian randomisation approach is that estimates can be biased by pleiotropic pathways where the instrument is associated with the outcome through a phenotype independent of the exposure under consideration. To investigate this possibility, we additionally performed mendelian randomisation-Egger and weighted median sensitivity analyses that are more robust to such pleiotropy,^34^ ^36^ ^56^ which produced results consistent with those from the main mendelian randomisation analyses. If we assume that the genetic variants have a monotonic effect on the exposure, mendelian randomisation estimates will reflect the average effect of the exposure on the outcome for all individuals whose exposure was affected by the genetic instrument. We found little evidence of heterogeneity in the effect of the exposures. This suggests that the effects of the single nucleotide polymorphisms on the exposure might be similar across the population, in which case the mendelian randomisation estimate could be a relatively unbiased estimate of the average effect in the population.

Analyses in the UK Biobank were carried out on white, British participants, potentially limiting the generalisability of our results to other populations and ethnicities. However, two-sample mendelian randomisation analyses were not exclusive to white European participants (although proportions were low for other populations) and produced consistent results to one-sample mendelian randomisation analyses. The UK Biobank is not representative of the UK population as a whole and is subject to healthy volunteer bias.^56^ ^57^

When estimating the indirect effects of a mediator on a binary outcome, the product of the coefficients method results in the least amount of bias,^33^ and as such we used this approach for estimating the effects of education through each risk factor individually. However, this method cannot currently be used to consider multiple mediators simultaneously in a mendelian randomisation model. For this reason, we used the difference method for estimating the effect of education through the three risk factors collectively with mendelian randomisation. Although such an approach could introduce a theoretical risk of bias when investigating a binary outcome, individual level data analyses in the UK Biobank were also carried out on a linear risk difference scale to minimise any related issues. Estimates for the effect of education through the risk factors collectively were consistent between different scales in these analyses, and as such we would not expect any potential biases to alter the interpretation of our results.

### Clinical and public health implications

Past policies that increase the duration of compulsory education have improved health and such endeavours must continue.^4^ However, intervening directly on education is difficult to achieve without social and political reforms. The findings of this study have notable implications for policymakers as they identify potential strategies for reducing education inequalities in health. Furthermore, they also produce quantitative estimates of this, allowing specific consideration of the potential impact to public health. That BMI, systolic blood pressure, and smoking behaviour together explain less than half of the overall effect of education is an important finding of this work. Further research identifying the other related factors and the interplay between them will be key to reducing social inequalities in cardiovascular disease. Furthermore, work investigating more diverse populations will be necessary to support the extrapolation of these findings outside of the considered contexts.

### Conclusion

By using distinct analytical methods, including genetic approaches that can draw causal inference, our results suggest that interventions aimed at reducing BMI, systolic blood pressure, and smoking behaviour in European populations would lead to reductions in cases of cardiovascular disease attributable to lower levels of education. Importantly, over half of the effect of education on the risk of cardiovascular disease is not mediated through these risk factors and further work is required towards investigating this.

**What is already known on this topic**

Lower levels of education are causally related to a higher risk of cardiovascular disease

**What this study adds**

Body mass index, systolic blood pressure, and smoking behaviour mediate the effect of education
Cardiovascular disease attributable to lower levels of education can be reduced by intervening on these risk factors

## Web Extra

Extra material supplied by the author

## Notes

Contributors: ARC and DG contributed equally to this project and are joint first authors. AD and LDH contributed equally to this project and are joint senior authors. ARC and DG devised the project, analysed and cleaned the data, interpreted results, wrote and revised the manuscript. ARC primarily carried out analyses using the UK Biobank. DG primarily carried out two-sample mendelian randomisation analyses. AET, NMD, TT, JV, SB, GDS, MVH, IT, LDH, and AD devised the project, interpreted the results, and revised the manuscript. TT, JV, GH, SB, and GDS contributed to the design of the project and critically revised the manuscript. REW, MRM, RM, SS, and DW provided data, and critically reviewed and revised the manuscript. All authors had full access to the data in the study and can take responsibility for the integrity of the data and the accuracy of the data analysis. ARC and DG are the guarantors. The corresponding author attests that all listed authors meet authorship criteria and that no others meeting the criteria have been omitted.

Ethical approval: Not required. Project approval was obtained from the UK Biobank (study ID: 10953) and data will be returned to them for archiving.

Data sharing: Statistical code is available on request from the corresponding author at alice.carter@bristol.ac.uk. All summary statistics for two-sample mendelian randomisation are available online from each genome-wide association study consortia.

The manuscript's guarantors (ARC and DG) affirm that the manuscript is an honest, accurate, and transparent account of the study being reported; that no important aspects of the study have been omitted; and that any discrepancies from the study as originally planned have been explained.
