# Supplementary material for: Understanding the consequences of education inequality on cardiovascular disease: mendelian randomisation study
Source: BMJ. 2019 May 22;365:l1855. doi: 10.1136/bmj.l1855 (PMC6529852; doi:10.1136/bmj.l1855)
Supplement: Supplementary file 1 — Supplementary materials: Supplementary methods, figures, and tables [file cara048356.ww1.pdf]

Supplementary Methods, Figures and Tables

Contents

Contents 1

Supplementary methods 5

UK Biobank exclusion criteria 5

UK Biobank observational phenotypes 5

Body mass index 5

Blood pressure 5

Smoking 5

Covariates 6

Outcomes 6

One-sample MR instrument selection (including GWAS methods) 6

GWAS meta-analysis data: education coding 7

GWAS meta-analysis data: CHD, MI and stroke data sources 7

Statistical analysis for one-sample MR 7

Investigating all three risk factors combined 8

Sensitivity analyses 8

References 9

Supplementary Figures 11

Supplementary Figure 1: Flow chart for exclusions made in UK Biobank for resultant sample for mediation analysis 11

Supplementary Figure 2: Flow chart for exclusions made in UK Biobank for use in SBP and smoking GWAS analyses 12

Supplementary Figure 3: A scatter plot of SNP-Education (x-axis) and SNP-CHD risk (y-axis) association estimates for the education instruments in two-sample MR analysis. 13

Supplementary Figure 4: A scatter plot of SNP-Education (x-axis) and SNP-Stroke risk (y-axis) association estimates for the education instruments in two-sample MR analysis. 14

Supplementary Figure 5: A scatter plot of SNP-Education (x-axis) and SNP-MI risk (y-axis) association estimates for the education instruments in two-sample MR analysis. 15

Supplementary Figure 6: Total and Direct effects for the association between 1-SD higher education and CVD outcomes estimated in one-sample MR analyses. 16

Supplementary Figure 7: Association between 1-SD higher education and BMI, SBP and smoking in one-sample MR. 18

Supplementary Figure 8: A scatter plot of SNP-Education (x-axis) and SNP-BMI (y-axis) association estimates for the education instruments in two-sample MR analysis. 19

Supplementary Figure 9: A scatter plot of SNP-Education (x-axis) and SNP-SBP (y-axis) association estimates for the education instruments in two-sample MR analysis. 20

Supplementary Figure 10: A scatter plot of SNP-Education (x-axis) and SNP-Smoking (y-axis) association estimates for the education instruments in two-sample MR analysis. 21

Supplementary Figure 11: Associations between BMI, SBP and smoking, and risk of the CVD outcomes in one-sample MR. 22

Supplementary Figure 12: Total and Direct effects for the association between 1-SD higher education and CVD outcomes estimated in multivariable observational analyses and two-sample MR analyses. 16

Supplementary Figure 13: Estimates for effect of a 1-SD increase in education on CVD and its subtypes as explained by BMI, SBP and smoking respectively using one-sample MR 23

Supplementary Figure 14: Estimates of the proportion mediated in observational multivariable analysis and one-sample MR analysis stratified by sex 24

Supplementary Figure 15: Estimates of the proportion mediated in observational multivariable analysis and one-sample MR analysis stratified by below the median of age (39-57 years in pink) and above the median of age (58-72 years in navy) 25

Supplementary Figure 16: Estimates for the effect of education on CVD and its subtypes explained by BMI, SBP and smoking respectively, estimated on the risk difference scale using the difference method. Results are provided for the multivariable observational analysis (plotted in pink) and one-sample MR (plotted in navy). 26

Supplementary Figure 17: Estimate of the additional proportion mediated by exercise and diet compared with BMI, SBP and smoking in multivariable observational multiple mediator models (N = 20 298) 27

Supplementary Tables 28

Supplementary Table 1: International Standard for Classification of Education codes mapped to UK Biobank self-report highest qualification to estimate years of education 28

Supplementary Table 2: ICD codes for cardiovascular outcomes in UK Biobank 29

Supplementary Table 3: Cohort details for UK Biobank sample used in multivariable observational and one-sample MR analysis compared with the whole UK Biobank cohort 30

Supplementary Table 4: Genetic association estimates for the two-sample regression-based MVMR analysis estimating the effect of BMI on CAD, adjusted for education. ea=effect allele; gx=BMI; gy=CAD; gz=education; se=standard error 32

Supplementary Table 5: Genetic association estimates for the two-sample regression-based MVMR analysis estimating the effect of BMI on MI, adjusted for education. ea=effect allele; gx=BMI; gy=MI; gz=education; se=standard error 40

Supplementary Table 6: Genetic association estimates for the two-sample regression-based MVMR analysis estimating the effect of BMI on stroke, adjusted for education. ea=effect allele; gx=BMI; gy=stroke; gz=education; se=standard error 48

Supplementary Table 7: Genetic association estimates for the IVW ratio-method MR analysis estimating the effect of education on BMI. ea=effect allele; gx=education; gy=BMI; se=standard error 56

Supplementary Table 8: Genetic association estimates for the IVW ratio-method MR analysis estimating the effect of education on CAD. ea=effect allele; gx=education; gy=CAD; se=standard error 68

Supplementary Table 9: Genetic association estimates for the IVW ratio-method MR analysis estimating the effect of education on MI. ea=effect allele; gx=education; gy=MI; se=standard error 96

Supplementary Table 10: Genetic association estimates for the IVW ratio-method MR analysis estimating the effect of education on SBP. ea=effect allele; gx=education; gy=SBP; se=standard error 124

Supplementary Table 11: Genetic association estimates for the IVW ratio-method MR analysis estimating the effect of education on smoking. ea=effect allele; gx=education; gy=smoking; se=standard error 152

Supplementary Table 12: Genetic association estimates for the IVW ratio-method MR analysis estimating the effect of education on stroke. ea=effect allele; gx=education; gy=stroke; se=standard error 180

Supplementary Table 13: Genetic association estimates for the two-sample regression-based MVMR analysis estimating the effect of SBP on CAD, adjusted for education. ea=effect allele; gx=SBP; gy=CAD; gz=education; se=standard error 208

Supplementary Table 14: Genetic association estimates for the two-sample regression-based MVMR analysis estimating the effect of SBP on MI, adjusted for education. ea=effect allele; gx=SBP; gy=MI; gz=education; se=standard error 213

Supplementary Table 15: Genetic association estimates for the two-sample regression-based MVMR analysis estimating the effect of SBP on stroke, adjusted for education. ea=effect allele; gx=SBP; gy=stroke; gz=education; se=standard error 218

Supplementary Table 16: Genetic association estimates for the two-sample regression-based MVMR analysis estimating the effect of smoking on CAD, adjusted for education. ea=effect allele; gx=smoking; gy=CAD; gz=education; se=standard error 223

Supplementary Table 17: Genetic association estimates for the two-sample regression-based MVMR analysis estimating the effect of smoking on MI, adjusted for education. ea=effect allele; gx=smoking; gy=MI; gz=education; se=standard error 226

Supplementary Table 18: Genetic association estimates for the two-sample regression-based MVMR analysis estimating the effect of smoking on stroke, adjusted for education. ea=effect allele; gx=smoking; gy=stroke; gz=education; se=standard error 230

Supplementary Table 19: Proxy SNPs for education instrument used in one-sample MR analysis 233

Supplementary Table 20: Genome-wide significant SNPs for SBP from split sample GWAS analysis in UK Biobank 234

Supplementary Table 21: Genome-wide significant SNPs for lifetime smoking from split sample GWAS analysis in UK Biobank 237

Supplementary Table 22: Unadjusted estimates for the proportion mediated in individual level UK Biobank analyses estimated from the log OR scale 238

Supplementary Table 23: Age and sex adjusted estimates for mediation by BMI, SBP and smoking on the association of education to CVD in individual level UK Biobank analyses 239

Supplementary methods

UK Biobank exclusion criteria

Individuals were excluded if their genetic sex differed to their gender reported at the assessment centre or for having aneuploidy of their sex chromosomes. Further individuals were excluded for being outliers for their heterozygosity and any missing genetic data. Related individuals were also excluded from analyses, and the remaining subset was a maximal set of unrelated individuals. This exclusion list was derived in-house using an algorithm applied to the list of all the related pairs provided by UK Biobank (3rd degree or closer) (Supplementary Figure 1). It preferentially removes the individuals related to the greatest number of other individuals until no related pairs remain (1). Additionally, individuals were excluded if there were any missing data for education, body mass index (BMI), systolic blood pressure (SBP), smoking, cardiovascular disease (CVD) status or for any of the variables considered as confounders.

UK Biobank observational phenotypes

Body mass index

BMI was measured at the baseline assessment centre from baseline measures of height and weight used to calculate BMI (kg/m^2).

In individual level MR analysis, we used 77 SNPs which had attained genome-wide significance for BMI in the Genetic Investigation of ANthropometric Traits (GIANT) Consortium genome-wide association study (GWAS) analysis of individuals with European ancestry (2). Alleles were harmonised to all reflect BMI increasing SNPs and individual variants were recoded as 0, 1 or 2 according to the number of BMI increasing alleles. A genetic score for BMI was created by weighting each SNP by its relative effect size in the GWAS and summing all variants together in an additive model.

Blood pressure

Blood pressure was recorded both automatically at the baseline assessment centre for all participants. Each reading was taken twice, two minutes apart. This analysis uses the second reading of the automated blood pressure, where missing data were replaced with the first measure or any follow up assessment centre measures.

Participants were required to take all medication they are currently using to the assessment centre, details of which were recorded by nurses. A variable for antihypertensive use was generated based on the treatments recorded and 10mmHg was added to SBP measurements for these individuals, consistent with previous studies (3).

Smoking

A measure of lifetime smoking was constructed in the UK Biobank from self-reported age at initiation, age at cessation and cigarettes per day. From this information, smoking duration and time since cessation were calculated. The lifetime smoking measure further includes a simulated constant (half-life) which captures the exponentially decreasing effect of cigarettes on health over time. Aspects of smoking behaviour were combined into one score ranging from 0 (for non-smokers) to 4.17. The mean lifetime smoking score was 0.35 (standard deviation = 0.69). Full details of score construction can be found elsewhere (4).

Covariates

Variables considered as covariates were measured at the baseline assessment centres through interviews. Sex and ethnicity were confirmed according to genetic data. Place of Birth was adjusted for by the northing and easting birth location coordinates. Although the Townsend Deprivation Index (TDI) of historic birth locations are not recorded in UK Biobank, this has been estimated from the index of multiple deprivation indices using the current TDI of birth location as a proxy for historic birth place TDI. Mendelian randomisation (MR) models were also adjusted for the same confounders. Although a core assumption of MR is that the genetic variants are unrelated to confounders, there is some evidence of small associations with place of birth for the educational attainment variants in UK Biobank (5, 6). These were only considered in observational and one sample MR analyses, where individual level data were available.

Outcomes

Incident cases of CVD were defined according to hospital episode statistics (HES). Date of diagnoses are provided by HES data, which was linked with the date of assessment centre provided by UK Biobank.

One-sample MR instrument selection (including GWAS methods)

For the one-sample MR analysis using individual-level data, instruments were selected from analysis of populations that did not overlap with those considered in the outcome estimates. Accordingly, we used 74 independent single-nucleotide polymorphisms (SNPs) that attained genome-wide significance ($P<5x10^{-8}$) for education reported in main results from the 2016 SSGAC GWAS meta-analysis of 293,723 individuals that did not include UK Biobank participants, to create a weighted allele score (7). The 77 reported genome-wide significant SNPs from the GIANT consortium's meta-analysis of 322,154 individuals of European ancestry identified were used to create a weighted allele score for BMI (2). Five instruments for education were not available in UK Biobank and proxy SNPs in perfect LD ($r^2=1$) were used (Supplementary Table 4).

MR studies require the SNP-exposure and the SNP-outcome associations to be estimated in independent samples, otherwise the MR estimates can be overestimated (8, 9). Existing SBP and lifetime smoking GWASs have been estimated using UK Biobank data (4, 10, 11). To avoid participant overlap for exposure and outcome genetic estimates in the UK Biobank (8), split sample GWASs of SBP and smoking respectively were performed using the University of Bristol MRC Integrative Epidemiology Unit GWAS Pipeline (12). A total of 318,147 unrelated UK Biobank participants were eligible for inclusion to the GWAS (see Supplementary Figure 2). All the eligible participants were randomly allocated into one of two halves (sample 1 and sample 2). A GWAS was performed on both samples 1 and 2 separately, adjusted for age, sex and the first 40 principle components in UK Biobank. A BOLT-LMM model was used to account for population stratification. The top hit SNPs were determined using the 'clump_data' command in the Two-Sample MR R package ($r^2 > 0.001$, distance >10,000kb) (default settings of the 'clump_data' command) (13). This process was carried out for both SBP and lifetime smoking phenotypes.

The genetic score was created for each sample independently, by weighting each SNP by its relative effect size from the GWAS results of the opposing sample (i.e. the genome-wide significant SNPs and betas identified in the GWAS of sample 1 were used to generate the genetic score in sample 2 individuals). All genetic variants were summed together in an additive model. A total of 65 and 55 genome-wide significant SNPs were identified for SBP (with 10mmHg added for antihypertensive use) for sample 1 and sample 2 respectively (see Supplementary Table 5). In the split-sample GWAS

for smoking, 18 SNPs were identified in the GWAS of sample 1 individuals and 15 SNPs in sample 2 individuals (see Supplementary Table 6).

Additionally, published SBP GWAS have been adjusted for BMI. Given the consideration for BMI as a mediator in this analysis and the potential for collider bias, we carried out a further GWAS of SBP in UK Biobank using the full eligible sample in a single GWAS to provide instruments for two-sample MR analysis that were not adjusted for BMI. This used the same pipeline and adjustments as described previously for the split sample analysis. As with the split sample approach, the independent genome-wide significant SNPs were determined using the 'clump_data' command in the Two-Sample MR R package ($r^2 > 0.001$ and distance >10,000kb) (default settings of the 'clump_data' command) (14).

GWAS meta-analysis data: education coding

In the SSGAC education GWAS, education was assessed at or above the age of 30 years, with comparability between studies heterogeneous in their educational systems maximised by mapping major educational qualifications on to one of seven categories of the ISCED (7, 17).

GWAS meta-analysis data: CHD, MI and stroke data sources

For the risk of CHD we used publicly available genetic association estimates from the CARDIoGRAMplusC4D 1000 Genomes-based GWAS meta-analysis of 60,801 cases and 123,504 controls (15). Participants were of European, East Asian, South Asian, Hispanic and African American ancestry, and adjustment was made for population stratification using the genomic control method

For the risk of MI, we used genetic association estimates were generated from the CARDIoGRAMplusC4D subgroup analysis of approximately 70% of the total cases that had a reported history of MI (15). For risk of stroke risk, we used publicly available genetic association estimates from the MEGASTROKE consortium GWAS meta-analysis of 67,162 stroke cases (comprising of ischaemic stroke, intracerebral haemorrhage and stroke of unknown type) and 406,111 controls (16).

All genetic association estimates used in each two-sample MR analyses are provided in Supplementary Tables 7-21.

Statistical analysis for one-sample MR

In the one-sample MR of UK Biobank data, the total effect of education on CVD outcomes was investigated using two-stage least squares regression. In the first regression, we estimated the effect of the education weighted allele score on self-reported educational attainment. We used this estimate to generate a prediction of educational attainment. In the second stage, we estimate the effect of predicted educational attainment on the CVD outcome using robust standard errors (18). Both regression stages were adjusted for adjusted for age, sex, place of birth, birth distance from London, and TDI as well as the first ten principal components (PCs).

To estimate the effect of the education weighted allele score on each of BMI, SBP and smoking, the Stata IVREG2 package was used, adjusted covariates and PCs as above.

To then estimate the effect of each risk factor individually on the CVD outcomes, linear regression models were used to estimate the gene-exposure association between the weighted allele score for each risk factor and the observed value, whilst controlling for the weighted allele score of education. Additionally, the gene-education estimates whilst controlling for the allele score of the risk factor were calculated. For both models, the predicted values were stored for use in a second stage

regression, where they were regressed against each CVD outcome risk using logistic regression. The final estimate of interest came from the predicted value of the mediator, controlling for education and all other covariates and PCs as previously described. Where split sample GWAS estimates were used to create the allele score in SBP and smoking the MR analyses were run separately for each 50% sample and meta-analysed to estimate an overall effect.

These estimates were then multiplied to estimate the indirect effect, which is the amount of the association between education and CVD going via each of the three risk factors individually.

Investigating all three risk factors combined

When investigating the role of all three risk factors together on the association between education and CVD, we used the difference method (19). This involved estimating the total effect of education on each CVD subtype, as described in the main Methods. We estimated the direct effect of education on each CVD subtype controlling for all three risk factors together, using either multivariable regression or multivariable MR, in observational and MR analyses respectively. In two-sample MR the direct effect was divided by the total effect to give a proportion, and this was then subtracted from one to estimate the amount of indirect mediation through the risk factors. To estimate the total effect of education mediated indirectly through all three risk factors collectively using two-sample MR, the direct effect of education after adjusting for the three risk factors together was estimated using MVMR, with this estimate divided by the total effect and then subtracted from one. In observational analysis, a multivariable logistic model for the effect of education on CVD (and subtypes) adjusting for all three risk factors was used to estimate the direct effect of education independently of the risk factors. This was subtracted from the total effect to estimate the indirect effect of education through the three risk factors collectively.

Sensitivity analyses

MR estimates are prone to bias if the underlying assumptions of the analysis are violated (20). Horizontal pleiotropy, where a genetic variant is associated to the outcome of interest via an alternative pathway, can potentially bias the MR estimates (20). MR-Egger allows for directional (unbalanced) horizontal pleiotropy under the assumption that the size of the effects of the variants on the exposure are independent of their direct effects on the outcome (i.e. there is no dose-response confounding) (21). Furthermore, the weighted median estimator is able to provide robust MR estimates when more than half of the information for the analysis comes from valid instruments (22). In the MR analysis of the total effect of education on CVD outcome risk, and the effect of education on each risk factor, we also perform these techniques to investigate the robustness of our findings when relaxing assumptions on horizontal pleiotropy. Incidentally, these techniques are not yet developed for application in MR mediation analysis.

For all analyses in UK Biobank, models were replicated on the risk difference scale using multivariable linear regression. For the one-sample MR analyses, the IVREG2 Stata package was used for this (23). Additionally, all analyses were replicated using unadjusted models, models adjusted for age and sex only, and models stratified by sex and age dichotomized at the median (39-57 years compared with 58 to 72 years). On a subsample of UK Biobank participants with dietary recall questionnaires (including protein, carbohydrate, total fat, saturated fat, polyunsaturated fat, total sugar and fibre consumption) and exercise (weekly duration of moderate and vigorous physical activity) measures (N = 20,298), an observational multivariable multiple mediator model was analysed. This could not be completed using MR analyses as there are not suitable instruments for diet and exercise phenotypes. This analysis, and those stratified by age and sex, were carried out for the association between education and CVD (all subtypes) only, due to limited outcome events.

References

1. Mitchell R, Hemani, G, Dudding, T, Paternoster, L. UK Biobank Genetic Data: MRC-IEU Quality Control, Version 1. 2017.
2. Locke AE, Kahali B, Berndt SI, Justice AE, Pers TH, Day FR, et al. Genetic studies of body mass index yield new insights for obesity biology. Nature. 2015;518(7538):197-206.
3. Tobin MD, Sheehan NA, Scurrah KJ, Burton PR. Adjusting for treatment effects in studies of quantitative traits: antihypertensive therapy and systolic blood pressure. Stat Med. 2005;24(19):2911-35.
4. Wootton RE, Richmond RC, Stuijfzand BG, Lawn RB, Sallis HM, Taylor GMJ, et al. Causal effects of lifetime smoking on risk for depression and schizophrenia: Evidence from a Mendelian randomisation study. bioRxiv. 2018.
5. Davies NM, Dickson M, Davey Smith G, van den Berg GJ, Windmeijer F. The causal effects of education on health outcomes in the UK Biobank. Nature Human Behaviour. 2018;2(2):117-25.
6. Haworth S, Mitchell R, Corbin L, Wade KH, Dudding T, Budu-Aggrey A, et al. Apparent latent structure within the UK Biobank sample has implications for epidemiological analysis. Nat Commun. 2019;10(1):333.
7. Okbay A, Beauchamp JP, Fontana MA, Lee JJ, Pers TH, Rietveld CA, et al. Genome-wide association study identifies 74 loci associated with educational attainment. Nature. 2016;533(7604):539-42.
8. Burgess S, Davies NM, Thompson SG. Bias due to participant overlap in two-sample Mendelian randomization. Genet Epidemiol. 2016;40(7):597-608.
9. Haycock PC, Burgess S, Wade KH, Bowden J, Relton C, Davey Smith G. Best (but oft-forgotten) practices: the design, analysis, and interpretation of Mendelian randomization studies. Am J Clin Nutr. 2016;103(4):965-78.
10. Evangelou E, Warren HR, Mosen-Ansorena D, Mifsud B, Pazoki R, Gao H, et al. Genetic analysis of over 1 million people identifies 535 new loci associated with blood pressure traits. Nat Genet. 2018.
11. Warren HR, Evangelou E, Cabrera CP, Gao H, Ren M, Mifsud B, et al. Genome-wide association analysis identifies novel blood pressure loci and offers biological insights into cardiovascular risk. Nat Genet. 2017;49(3):403-15.
12. MRC IEU UK Biobank GWAS pipeline version 1, (2017).
13. Hemani G, Zheng J, Wade KH, Laurin C, Elsworth B, Burgess S, et al. MR-Base: a platform for systematic causal inference across the phenome using billions of genetic associations. bioRxiv. 2016.
14. Davey Smith G, Hemani G. Mendelian randomization: genetic anchors for causal inference in epidemiological studies. Hum Mol Genet. 2014;23(R1):R89-98.
15. Nikpay M, Goel A, Won HH, Hall LM, Willenborg C, Kanoni S, et al. A comprehensive 1,000 Genomes-based genome-wide association meta-analysis of coronary artery disease. Nat Genet. 2015;47(10):1121-30.
16. Malik R, Chauhan G, Traylor M, Sargurupremraj M, Okada Y, Mishra A, et al. Multiancestry genome-wide association study of 520,000 subjects identifies 32 loci associated with stroke and stroke subtypes. Nat Genet. 2018;50(4):524-37.
17. Lee JJ, Wedow R, Okbay A, Kong E, Maghzian O, Zacher M, et al. Gene discovery and polygenic prediction from a genome-wide association study of educational attainment in 1.1 million individuals. Nat Genet. 2018.
18. Palmer TM, Holmes MV, Keating BJ, Sheehan NA. Correcting the Standard Errors of 2-Stage Residual Inclusion Estimators for Mendelian Randomization Studies. Am J Epidemiol. 2017;186(9):1104-14.
19. VanderWeele TJ. Mediation Analysis: A Practitioner's Guide. Annu Rev Public Health. 2016;37:17-32.
20. Sheehan NA, Didelez V, Burton PR, Tobin MD. Mendelian randomisation and causal inference in observational epidemiology. PLoS Med. 2008;5(8):e177.

21. Burgess S, Bowden J, Fall T, Ingelsson E, Thompson SG. Sensitivity analyses for bobust causal inference from Mendelian randomization analyses with multiple genetic variants. Epidemiology. 2017;28(1):30-42.
22. Bowden J, Davey Smith G, Haycock PC, Burgess S. Consistent Estimation in Mendelian Randomization with Some Invalid Instruments Using a Weighted Median Estimator. Genet Epidemiol. 2016;40(4):304-14.
23. Christopher F Baum MES, Steven Stillman. IVREG2: Stata module for extended instrumental variables/2SLS and GMM estimation. Statistical Software Components. 2002.

Supplementary Figures

Supplementary Figure 1: Flow chart for exclusions made in UK Biobank for resultant sample for mediation analysis

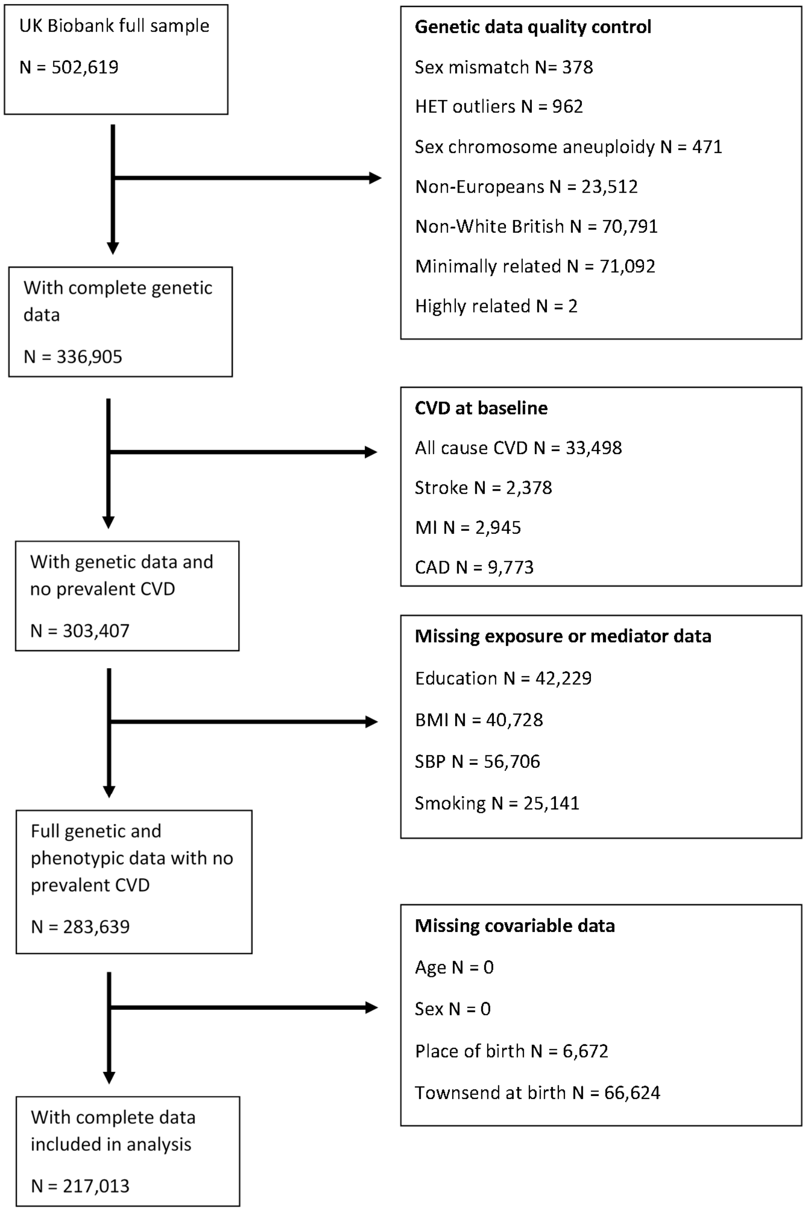

Supplementary Figure 2: Flow chart for exclusions made in UK Biobank for use in SBP and smoking GWAS analyses

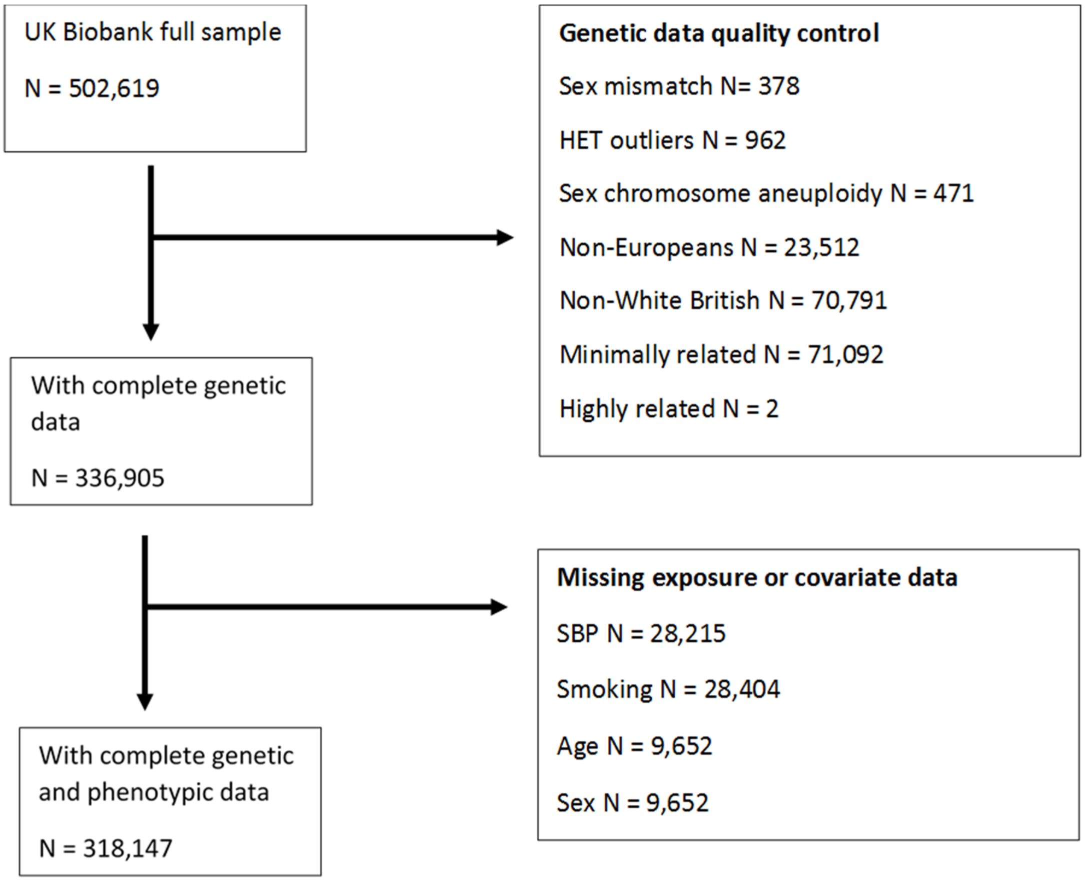

Supplementary Figure 3: A scatter plot of SNP-Education (x-axis) and SNP-CHD risk (y-axis) association estimates for the education instruments in two-sample MR analysis.

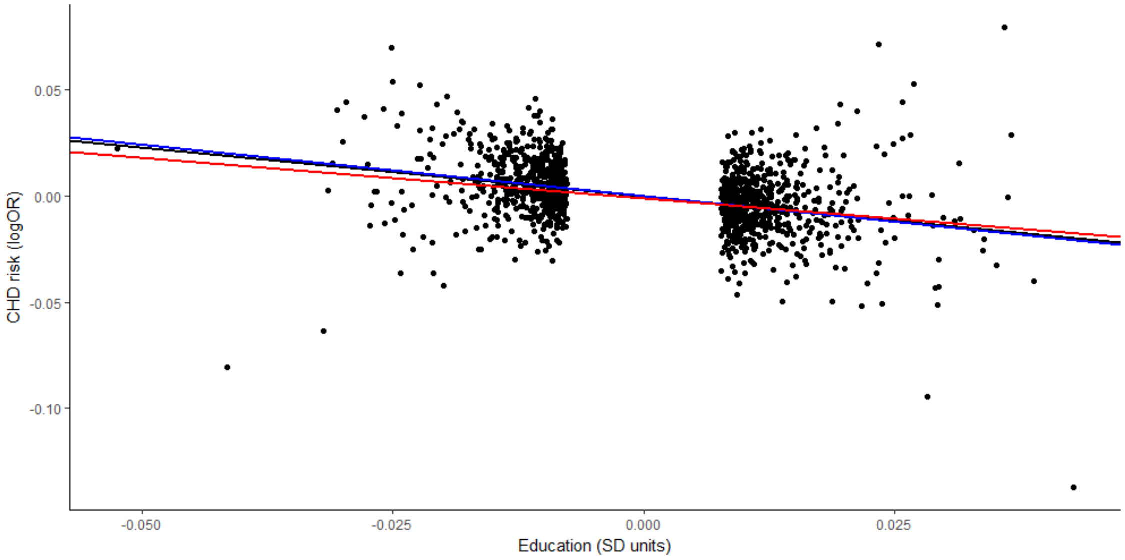

Black line = Inverse variance weighted MR estimate. estimate Blue line = Weighted median MR estimate. Red line = MR-Egger estimate

Supplementary Figure 4: A scatter plot of SNP-Education (x-axis) and SNP-Stroke risk (y-axis) association estimates for the education instruments in two-sample MR analysis.

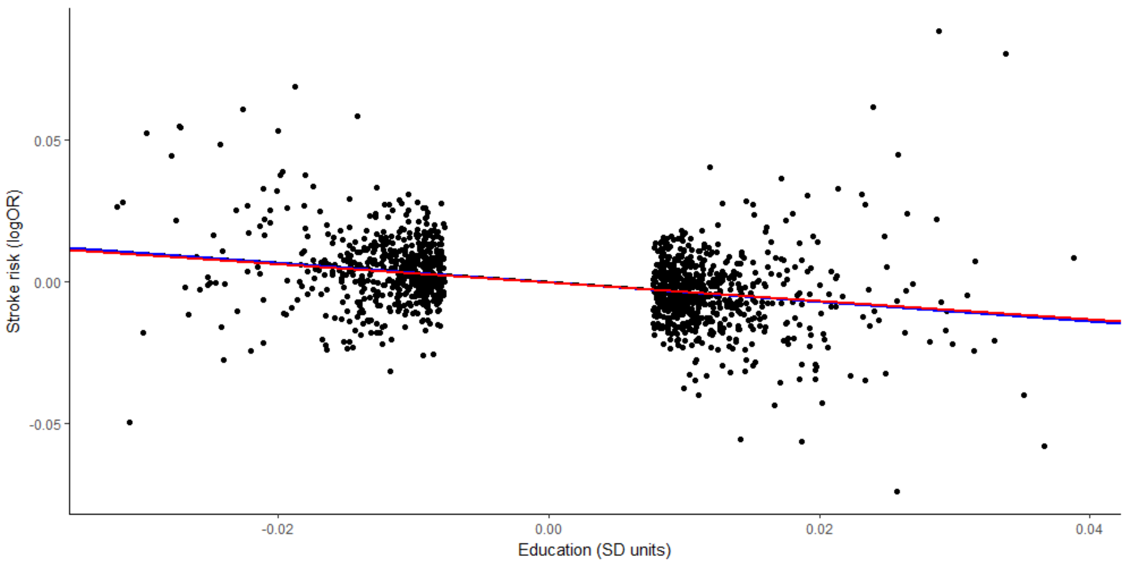

Black line = Inverse variance weighted MR estimate. estimate Blue line = Weighted median MR estimate. Red line = MR-Egger estimate

Supplementary Figure 5: A scatter plot of SNP-Education (x-axis) and SNP-MI risk (y-axis) association estimates for the education instruments in two-sample MR analysis.

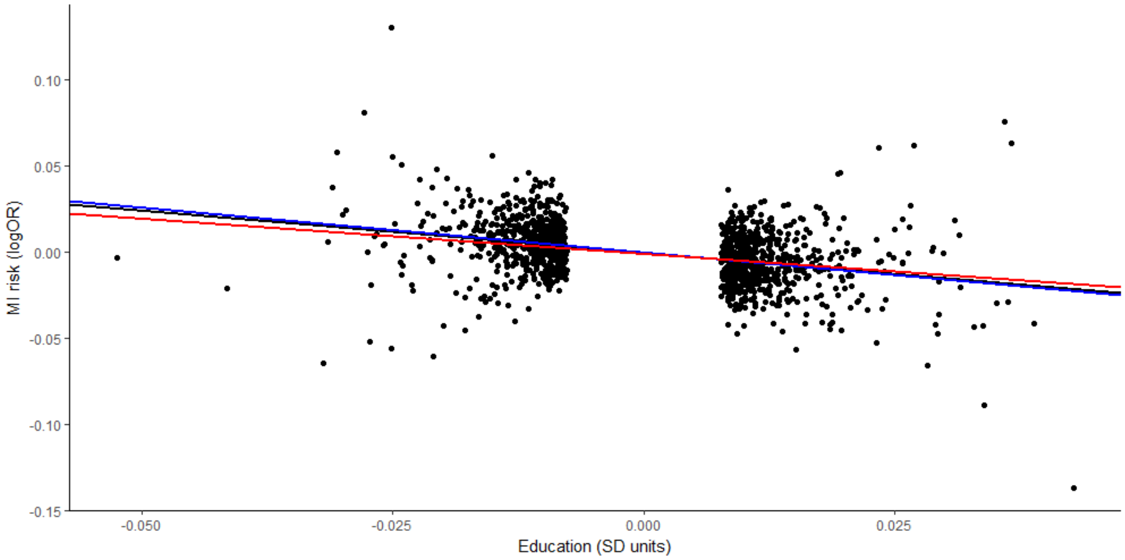

Black line = Inverse variance weighted MR estimate. estimate Blue line = Weighted median MR estimate. Red line = MR-Egger estimate

Supplementary Figure 6: Total and Direct effects for the association between 1-SD higher education and CVD outcomes estimated in multivariable observational analyses and two-sample MR analyses.

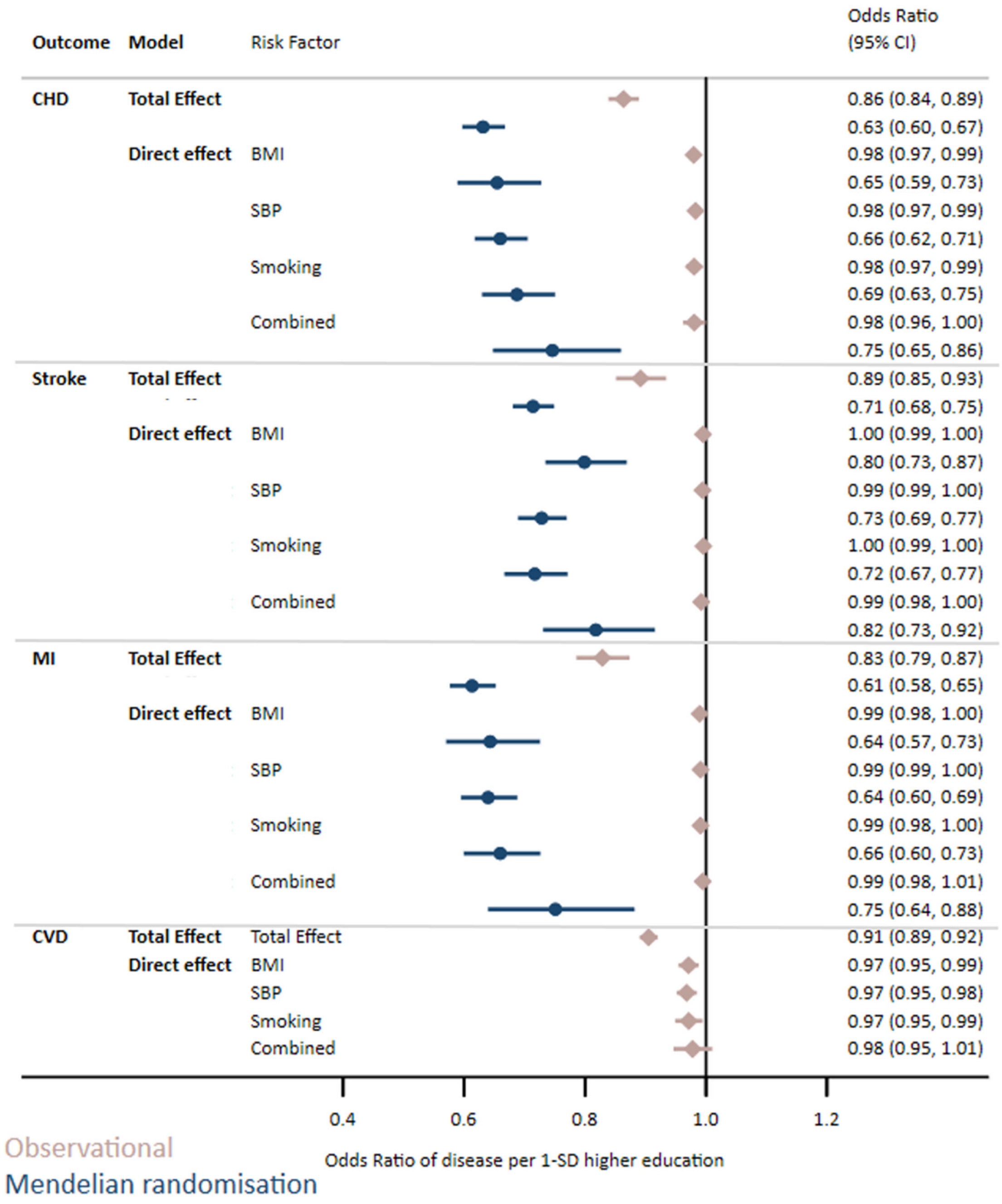

Total effect = overall effect of education on CVD, including that mediated by BMI, SBP, smoking, and all other risk factors on the causal pathway. Direct effect = the effect of education on CVD that is not mediated by the risk factor listed. E.g. direct effect of education on CHD with risk factor BMI is the effect of education on CHD that is not mediated by BMI. Analyses adjusted for: age, sex, place of birth and Townsend deprivation index at birth. BMI, SBP and smoking were measured in 1-SD units.

Supplementary Figure 7: Total and Direct effects for the association between 1-SD higher education and CVD outcomes estimated in one-sample MR analyses.

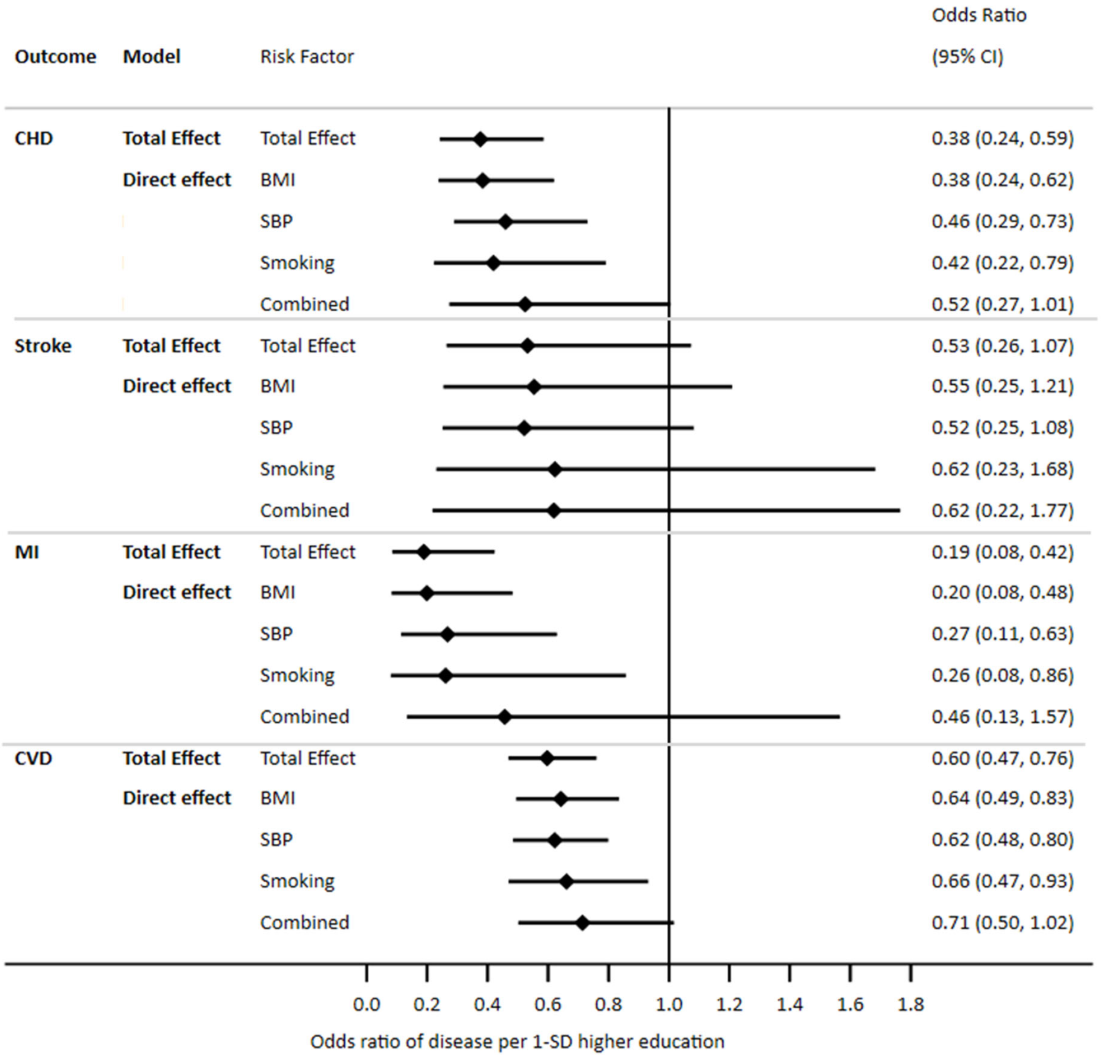

Total effect = overall effect of education on CVD, including that mediated by BMI, SBP, smoking, and all other risk factors on the causal pathway. Direct effect = the effect of education on CVD that is not mediated by the risk factor listed. E.g. direct effect of education on CHD with risk factor BMI is the effect of education on CHD that is not mediated by BMI. Analyses adjusted for: age, sex, place of birth and Townsend deprivation index at birth. BMI, SBP and smoking were measured in 1-SD units.

Supplementary Figure 8: Association between 1-SD higher education and BMI, SBP and smoking in one-sample MR.

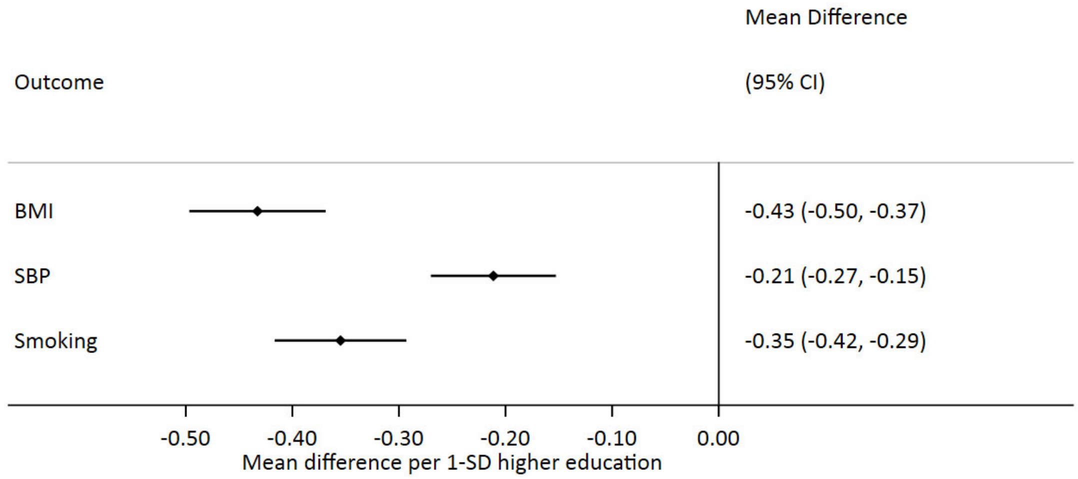

Analyses were adjusted for: age, sex, place of birth and Townsend deprivation index at birth. BMI, SBP and smoking were measured in 1-SD units.

Supplementary Figure 9: A scatter plot of SNP-Education (x-axis) and SNP-BMI (y-axis) association estimates for the education instruments in two-sample MR analysis.

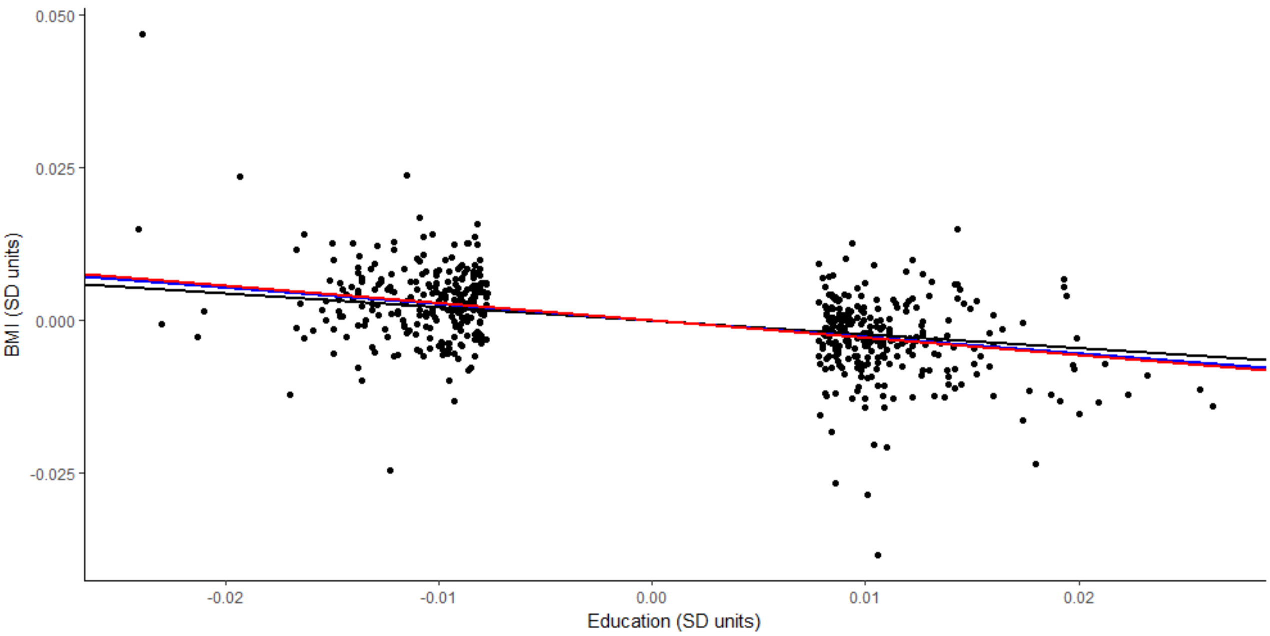

Black line = Inverse variance weighted MR estimate. estimate Blue line = Weighted median MR estimate. Red line = MR-Egger estimate

Supplementary Figure 10: A scatter plot of SNP-Education (x-axis) and SNP-SBP (y-axis) association estimates for the education instruments in two-sample MR analysis.

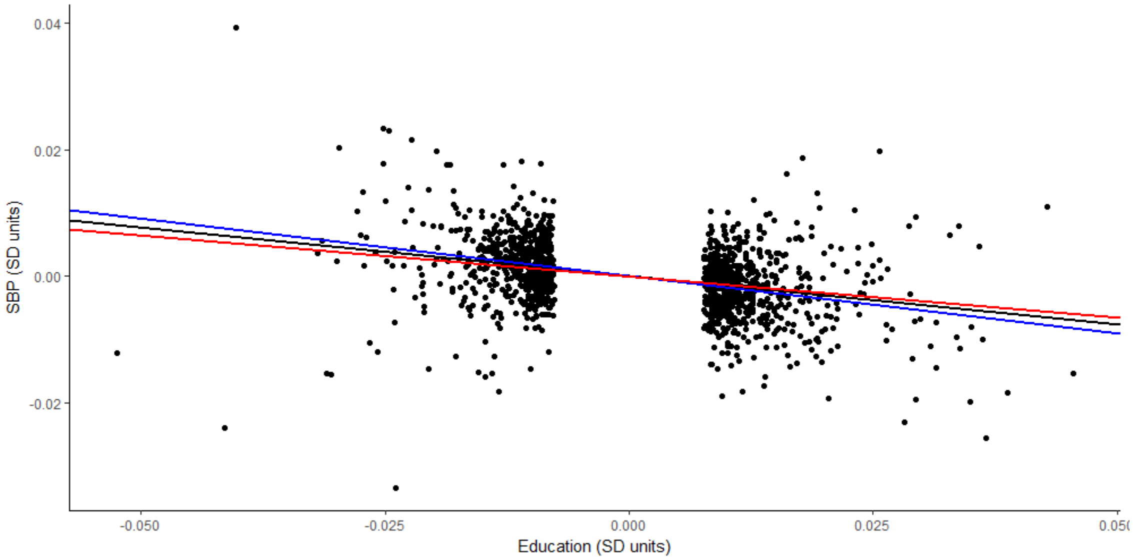

Black line = Inverse variance weighted MR estimate. estimate Blue line = Weighted median MR estimate. Red line = MR-Egger estimate

Supplementary Figure 11: A scatter plot of SNP-Education (x-axis) and SNP-Smoking (y-axis) association estimates for the education instruments in two-sample MR analysis.

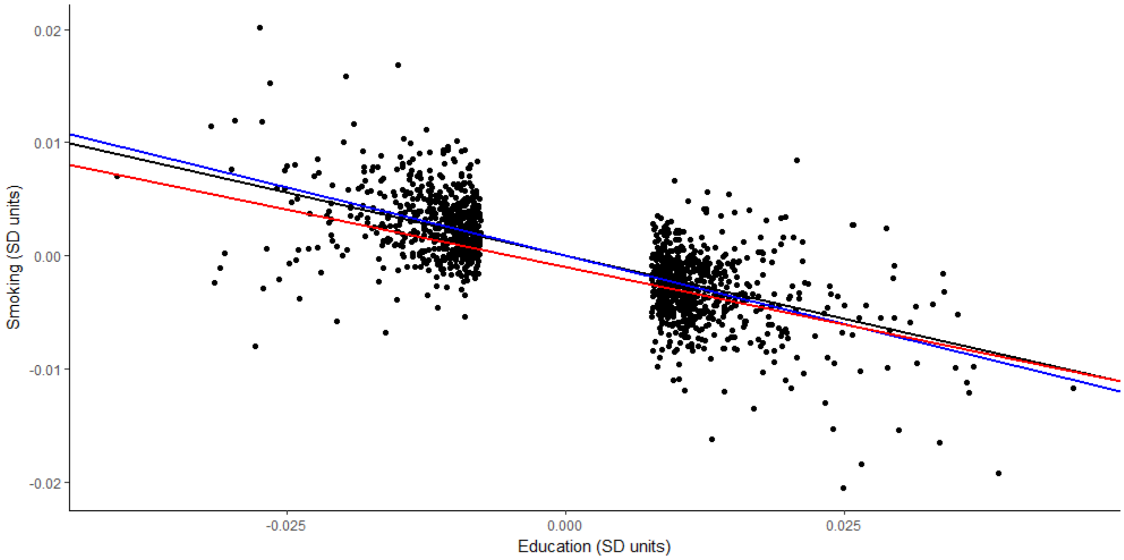

Black line = Inverse variance weighted MR estimate. estimate Blue line = Weighted median MR estimate. Red line = MR-Egger estimate

Supplementary Figure 12: Associations between BMI, SBP and smoking, and risk of the CVD outcomes in one-sample MR.

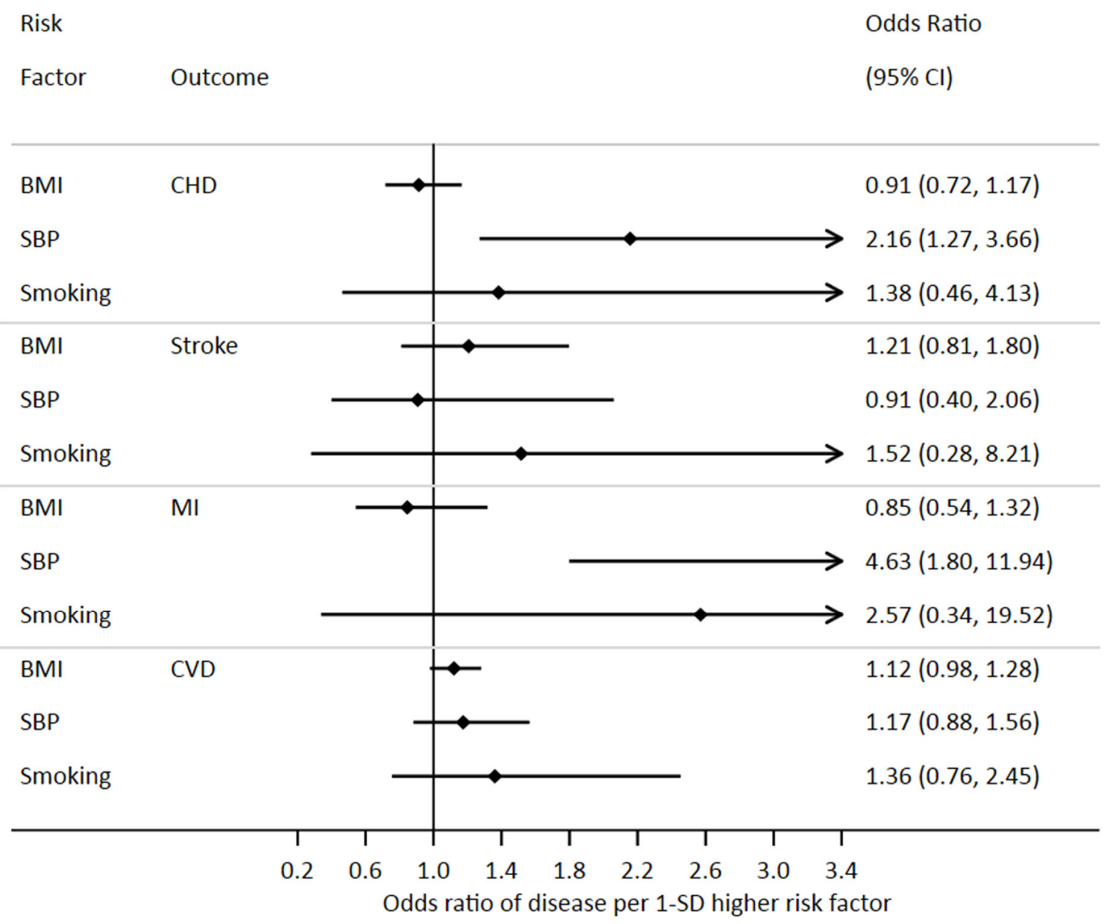

Analyses adjusted for: age, sex, place of birth and Townsend deprivation index at birth. BMI, SBP and smoking were measured in 1-SD units.

Supplementary Figure 13: Estimates for effect of a 1-SD increase in education on CVD and its subtypes as explained by BMI, SBP and smoking respectively using one-sample MR

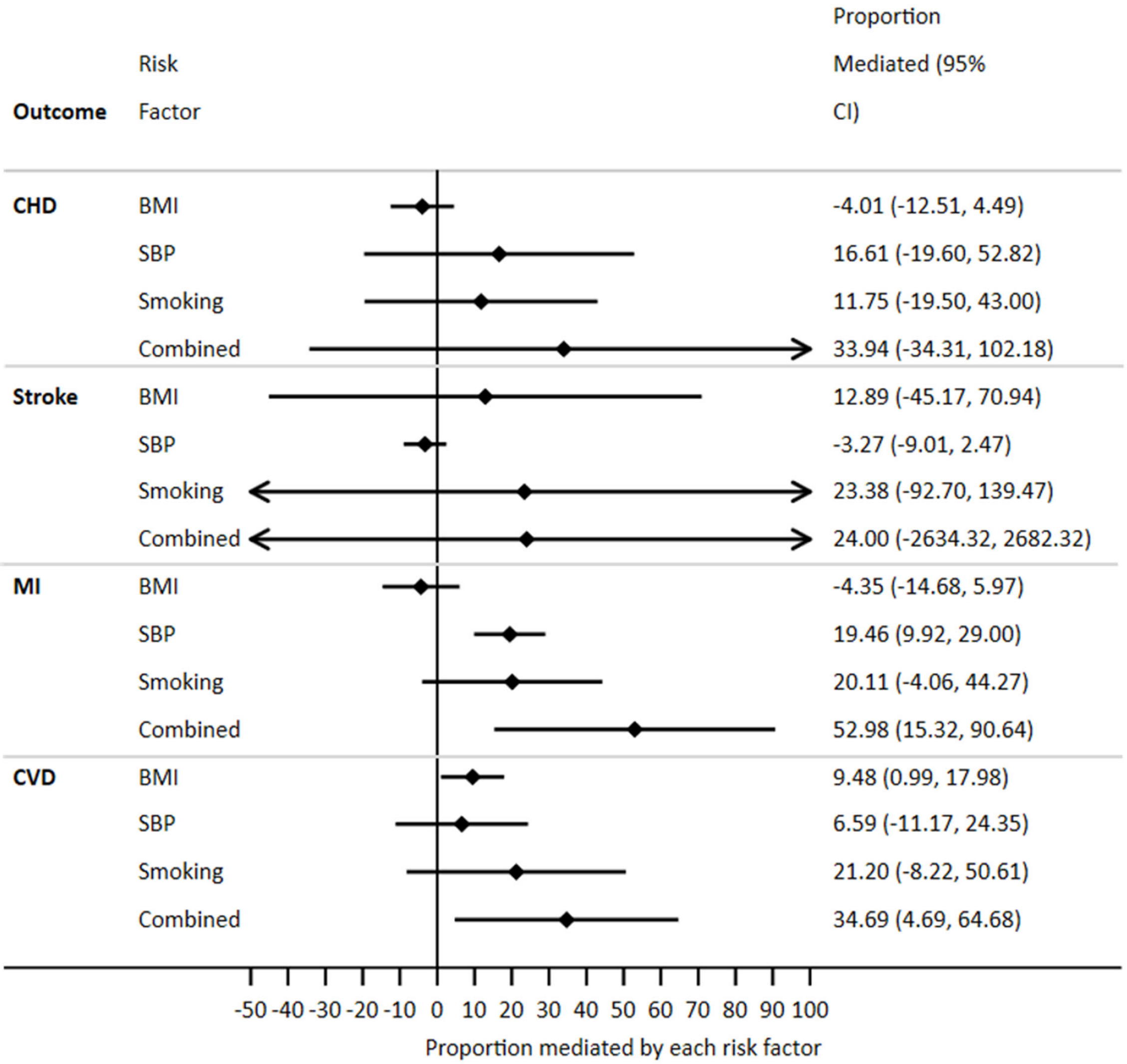

Supplementary Figure 14: Estimates of the proportion mediated in observational multivariable analysis and one-sample MR analysis stratified by sex

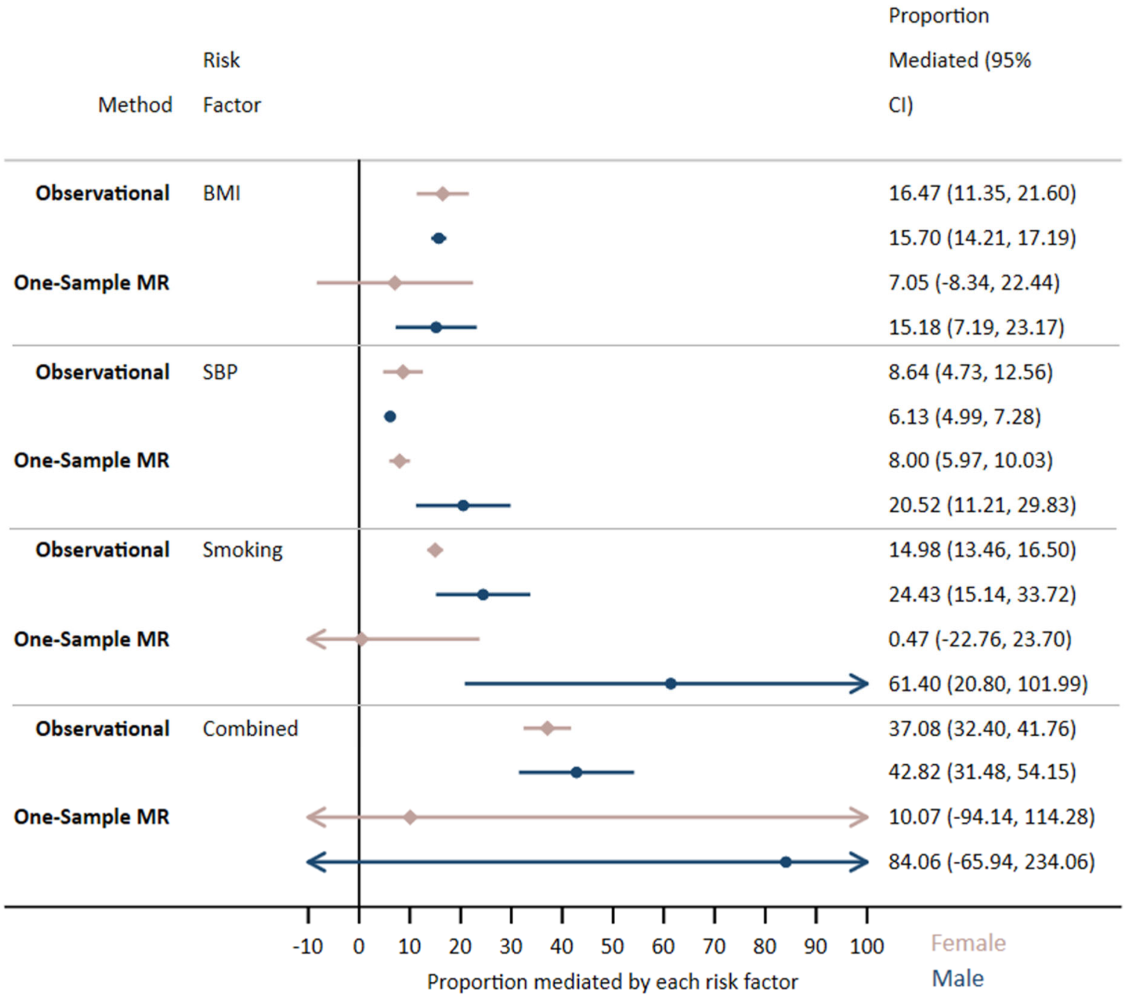

Analyses adjusted for: age, place of birth and Townsend deprivation index at birth. BMI, SBP and smoking were measured in 1-SD units. Male results are plotted in pink. Female results are plotted in navy.

Supplementary Figure 15: Estimates of the proportion mediated in observational multivariable analysis and one-sample MR analysis stratified by below the median of age (39-57 years in pink) and above the median of age (58-72 years in navy)

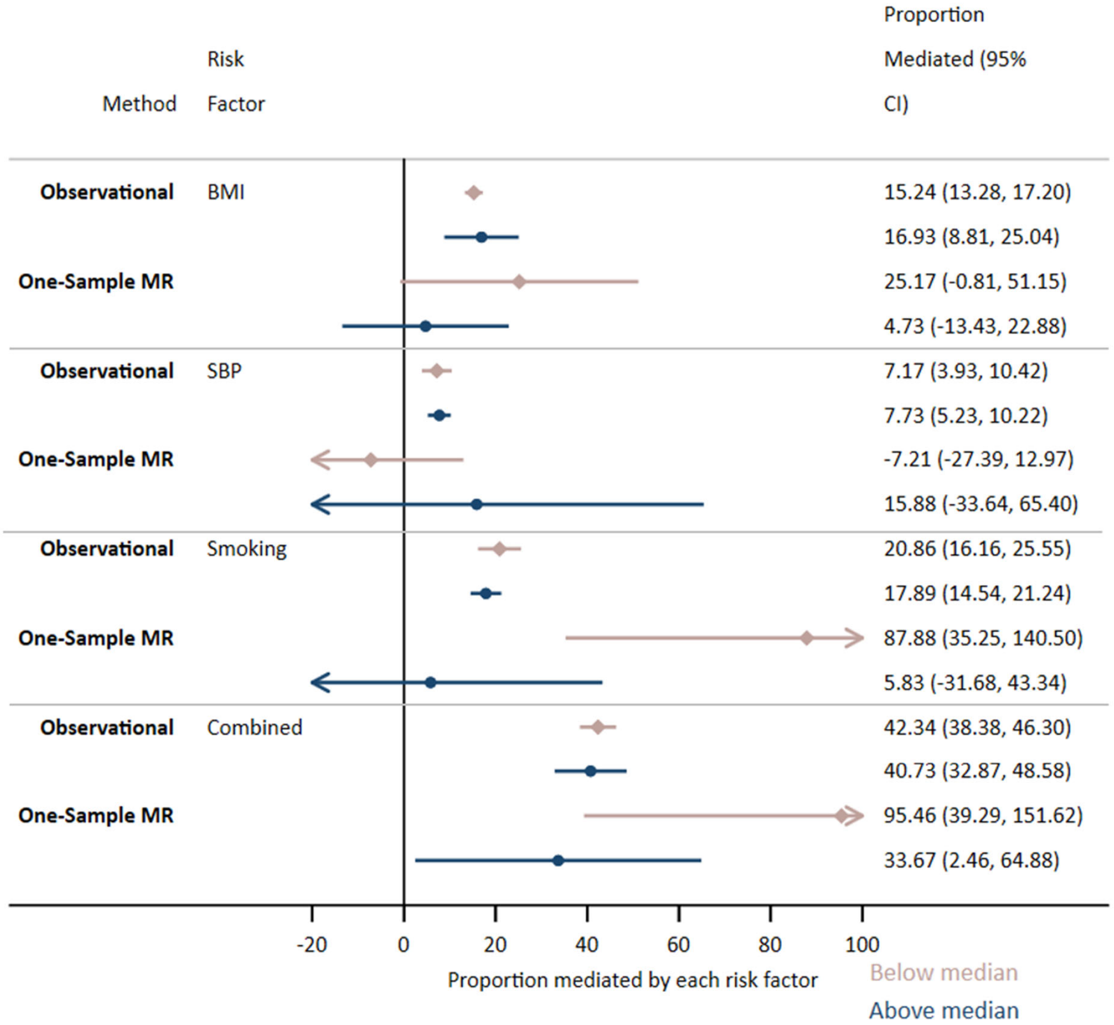

Analyses adjusted for: age, sex, place of birth and Townsend deprivation index at birth. BMI, SBP and smoking were measured in 1-SD units. Female results are plotted in pink. Male results are plotted in navy.

Supplementary Figure 16: Estimates for the effect of education on CVD and its subtypes explained by BMI, SBP and smoking respectively, estimated on the risk difference scale using the difference method. Results are provided for the multivariable observational analysis (plotted in pink) and one-sample MR (plotted in navy).

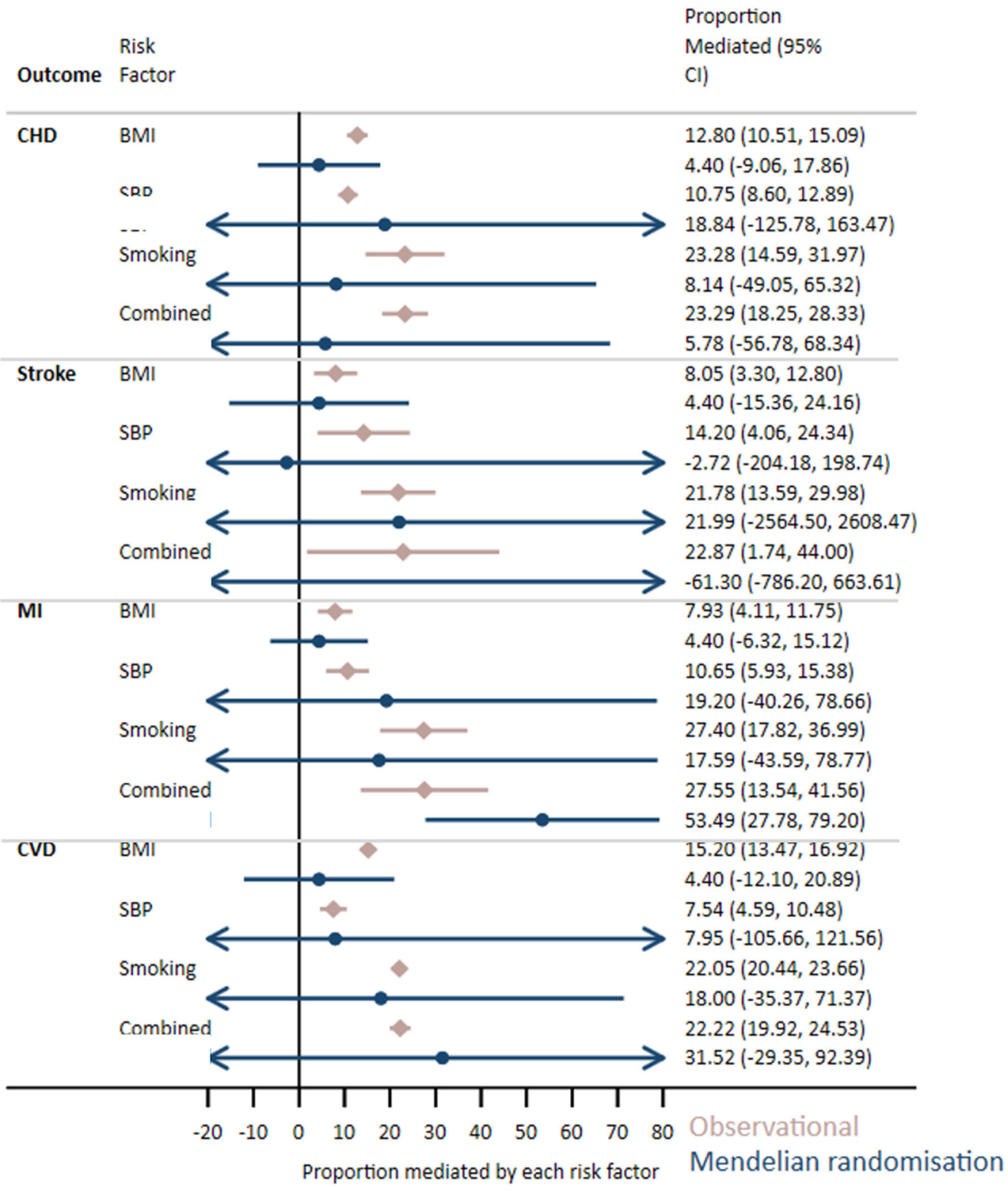

Supplementary Figure 17: Estimate of the additional proportion mediated by exercise and diet compared with BMI, SBP and smoking in multivariable observational multiple mediator models (N

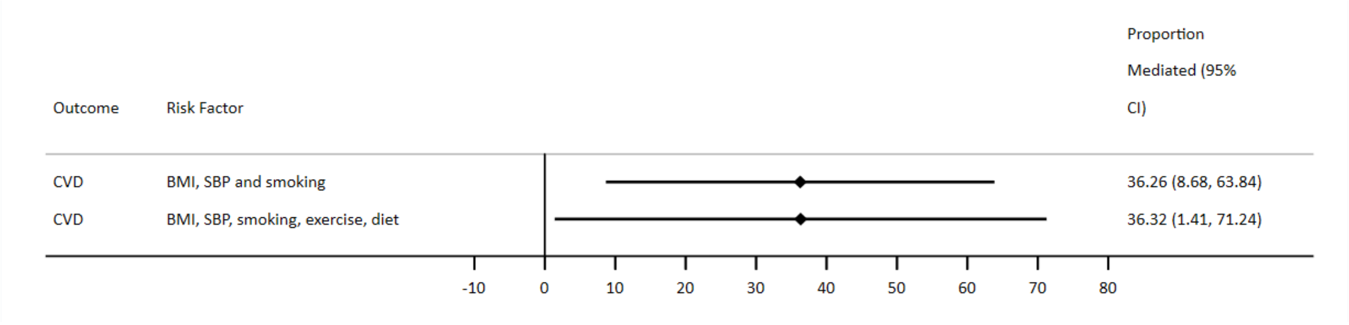

= 20 298)

Supplementary Tables

Supplementary Table 1: International Standard for Classification of Education codes mapped to UK Biobank self-report highest qualification to estimate years of education

Qualification (As reported in UK Biobank)	ISCED	Years of education
**College or University degree**	5	20
**NVQ or HND or HNC or equivalent**	5	19
**Other prof. qual. eg: nursing, teaching**	4	15
**A levels/AS levels or equivalent**	3	13
**O levels/GCSEs or equivalent**	2	10
**CSEs or equivalent**	2	10
**None of the above**	1	7
**Prefer not to answer**	Excluded	

Supplementary Table 2: ICD codes for cardiovascular outcomes in UK Biobank

Outcome	ICD-9 code	ICD-10 code
CVD (all subtypes)	390-459	I, G45
Stroke	434.91	I6, G45
MI	410.9, 412.9	I21, I22
CHD	410-414	I20-I25

Supplementary Table 3: Cohort details for UK Biobank sample used in multivariable observational and one-sample MR analysis compared with the whole UK Biobank cohort

Variable	Level	N Analysis Sample	% Analysis Sample		N Full UKBB	% Full UKBB
**Sex**	Male	108,563	46		229,164	45.63
	Female	127,867	54		273,076	54.37
**Age**	<40	2,342	0.99		5,424	1.08
	41-50	56,888	24.06		126,426	25.15
	51-60	83,219	35.2		177,264	35.27
	61-70	93,976	39.75		193,119	38.42
	71+	5	<0.01		422	0.08
**ISCED Category**	1	39,396	16.77		84,895	17.23
	2	41,501	28.27		82,757	16.79
	3	12,787	11.62		27,008	5.48
	4	29,298	5.13		58,680	11.91
	5	113,448	38.22		239,464	48.59
**BMI**	Underweight	1,143	0.48		2,624	0.52
	Normal	77,598	32.82		162,261	32.28
	Overweight	101,288	42.84		212,071	42.19
	Obese	56,401	23.86		125,699	25.01
**SBP**	(Mean)	136.39			135.95	
**SBP - Medication adjusted**	(Mean)	137.3			136.86	
**Smoking initiation**	Never	94,058	39.78		200,747	40.2
	Ever (inc current and former)	142,372	60.22		298,665	59.8
**CVD**	Case	198,207	92.77		418,126	92.38
	Control	15,448	7.23		34,513	7.62
**Stroke**	Case	198,207	99.18		418,126	99.09
	Control	1,640	0.82		3,840	0.91
**Acute Myocardial Infarction**	Case	198,207	99.37		418,126	99.32
	Control	1,265	0.63		2,860	0.68
**Coronary Heart Disease**	Case	198,207	97.82		418,126	97.74
	Control	4,407	2.18		9,677	2.26
**BMI**	R-square value for instrument	0.017				
**SBP**	Sample 1 - R-square value for instrument	0.0035				

	Sample 2 - R-square value for instrument	0.0027				
**Lifetime smoking**	Sample 1 - R-square value for instrument	0.0012				
	Sample 2 - R-square value for instrument	0.0014				

Supplementary Table 4: Genetic association estimates for the two-sample regression-based MVMR analysis estimating the effect of BMI on CAD, adjusted for education. ea=effect allele; gx=BMI; gy=CAD; gz=education; se=standard error

SNP	ea	gx	gx_se	gy	gy_se	gz	gz_se
rs10083803	T	-0.0139	0.0022	0.0051	0.0107	-0.0020	0.0019
rs10124645	A	0.0124	0.0020	0.0061	0.0101	-0.0041	0.0017
rs10128597	A	-0.0186	0.0022	-0.0107	0.0101	-0.0004	0.0019
rs1013293	A	-0.0177	0.0020	-0.0252	0.0098	0.0030	0.0017
rs10145154	T	0.0288	0.0024	0.0208	0.0120	0.0002	0.0021
rs10160769	C	-0.0154	0.0024	-0.0082	0.0111	0.0125	0.0021
rs10169594	T	-0.0122	0.0020	-0.0117	0.0100	0.0086	0.0018
rs10206166	T	0.0168	0.0020	0.0367	0.0099	-0.0111	0.0018
rs10236214	T	0.0145	0.0021	-0.0180	0.0106	0.0015	0.0018
rs10259490	A	-0.0129	0.0020	-0.0088	0.0094	-0.0023	0.0017
rs10423928	A	-0.0342	0.0025	-0.0399	0.0125	0.0031	0.0021
rs1045411	T	-0.0163	0.0022	-0.0011	0.0113	0.0032	0.0019
rs1048932	A	-0.0149	0.0020	-0.0022	0.0094	0.0041	0.0017
rs10514944	A	0.0247	0.0034	0.0150	0.0148	-0.0074	0.0029
rs10514975	A	-0.0139	0.0020	-0.0179	0.0095	0.0012	0.0017
rs10515237	A	-0.0151	0.0022	0.0008	0.0105	-0.0003	0.0019
rs1064213	A	0.0146	0.0019	0.0052	0.0096	-0.0026	0.0017
rs10742752	T	-0.0119	0.0020	0.0067	0.0095	-0.0026	0.0017
rs10756714	A	0.0199	0.0020	0.0072	0.0093	-0.0048	0.0017
rs10768994	T	0.0125	0.0020	0.0059	0.0094	-0.0017	0.0017
rs10790519	T	-0.0124	0.0020	-0.0179	0.0096	0.0041	0.0018
rs10818810	A	0.0125	0.0020	0.0248	0.0097	-0.0051	0.0018
rs10824218	A	0.0141	0.0020	0.0131	0.0100	0.0020	0.0017
rs10841188	A	-0.0135	0.0022	-0.0202	0.0101	0.0010	0.0020
rs10842240	C	0.0215	0.0031	0.0006	0.0120	-0.0071	0.0027
rs10878946	T	-0.0148	0.0022	-0.0110	0.0103	-0.0015	0.0019
rs10887578	C	0.0134	0.0020	-0.0014	0.0096	-0.0019	0.0017
rs10899736	A	-0.0116	0.0020	-0.0109	0.0093	-0.0020	0.0017
rs10921743	A	0.0138	0.0020	0.0148	0.0097	-0.0021	0.0018
rs10938397	A	-0.0292	0.0020	-0.0306	0.0093	0.0066	0.0017
rs10962550	C	0.0196	0.0025	0.0235	0.0115	-0.0093	0.0022
rs11075489	T	-0.0115	0.0020	-0.0069	0.0092	0.0023	0.0017
rs11079849	T	-0.0213	0.0021	-0.0506	0.0109	0.0074	0.0018
rs11084553	A	0.0236	0.0027	0.0239	0.0150	0.0045	0.0024
rs11134679	A	-0.0177	0.0021	-0.0032	0.0104	0.0025	0.0018
rs11150461	C	0.0148	0.0022	0.0099	0.0105	0.0017	0.0019
rs11161044	C	0.0175	0.0025	0.0336	0.0111	-0.0180	0.0021
rs11165493	A	0.0143	0.0021	0.0068	0.0101	0.0053	0.0018
rs11190658	T	0.0150	0.0024	0.0127	0.0112	0.0002	0.0021
rs11223970	A	-0.0193	0.0026	-0.0017	0.0134	0.0049	0.0023
rs11251352	A	-0.0117	0.0020	0.0066	0.0094	0.0038	0.0017

rs11496125	T	0.0189	0.0020	0.0102	0.0093	0.0025	0.0017
rs11505821	A	-0.0301	0.0040	-0.0355	0.0176	-0.0030	0.0036
rs11609297	T	-0.0172	0.0023	-0.0174	0.0122	0.0058	0.0020
rs11611246	T	0.0255	0.0024	0.0074	0.0114	0.0079	0.0021
rs11615578	T	0.0137	0.0023	-0.0068	0.0117	0.0022	0.0020
rs11675464	A	-0.0117	0.0020	0.0412	0.0096	-0.0001	0.0017
rs11713193	A	0.0266	0.0019	0.0301	0.0097	-0.0225	0.0017
rs11757278	T	0.0146	0.0021	-0.0071	0.0101	-0.0073	0.0018
rs11773362	T	-0.0125	0.0021	0.0073	0.0097	0.0033	0.0018
rs11846126	T	0.0158	0.0020	0.0119	0.0095	-0.0037	0.0017
rs11874191	T	-0.0151	0.0023	0.0109	0.0104	0.0069	0.0020
rs11895606	T	0.0131	0.0020	-0.0082	0.0099	0.0015	0.0017
rs11915371	A	-0.0148	0.0024	-0.0132	0.0116	0.0102	0.0021
rs11945861	A	-0.0143	0.0023	0.0047	0.0106	0.0068	0.0020
rs11976018	A	-0.0228	0.0027	-0.0100	0.0115	0.0041	0.0023
rs12049202	T	0.0245	0.0024	0.0273	0.0116	-0.0067	0.0021
rs1218822	A	0.0176	0.0021	0.0061	0.0098	-0.0024	0.0018
rs12334877	A	-0.0152	0.0025	-0.0151	0.0111	0.0047	0.0021
rs12369179	T	-0.0372	0.0034	-0.0101	0.0193	-0.0143	0.0030
rs12427047	T	-0.0184	0.0023	0.0065	0.0109	-0.0041	0.0020
rs12439798	T	0.0139	0.0020	0.0003	0.0094	-0.0038	0.0017
rs12448257	A	0.0157	0.0024	0.0097	0.0113	0.0074	0.0021
rs12512994	T	-0.0127	0.0021	-0.0067	0.0098	0.0140	0.0018
rs12564848	T	-0.0229	0.0031	-0.0032	0.0136	-0.0015	0.0027
rs12595749	A	0.0149	0.0020	-0.0037	0.0094	-0.0022	0.0017
rs12608738	A	0.0130	0.0022	-0.0028	0.0118	-0.0020	0.0019
rs1266874	A	-0.0147	0.0020	-0.0037	0.0097	0.0023	0.0018
rs12681792	A	0.0149	0.0025	-0.0024	0.0115	-0.0034	0.0021
rs12705977	T	0.0134	0.0020	0.0045	0.0096	-0.0079	0.0017
rs12762034	T	-0.0262	0.0037	-0.0037	0.0160	0.0059	0.0032
rs12878246	T	0.0137	0.0023	0.0172	0.0120	-0.0017	0.0020
rs12906208	A	-0.0149	0.0024	0.0089	0.0123	0.0039	0.0021
rs12936083	A	-0.0137	0.0021	-0.0002	0.0099	0.0014	0.0018
rs12936319	A	0.0167	0.0020	0.0038	0.0095	-0.0072	0.0017
rs12949279	T	0.0180	0.0020	0.0179	0.0095	-0.0031	0.0017
rs1296328	A	0.0190	0.0020	-0.0024	0.0099	-0.0024	0.0017
rs12981256	A	0.0140	0.0020	0.0139	0.0101	-0.0021	0.0017
rs13034320	C	0.0136	0.0023	0.0252	0.0108	-0.0012	0.0020
rs13047416	C	0.0143	0.0020	-0.0023	0.0096	0.0015	0.0018
rs13085002	T	-0.0168	0.0027	-0.0135	0.0124	0.0009	0.0023
rs13107325	T	0.0469	0.0037	-0.0067	0.0223	-0.0188	0.0034
rs13110266	A	-0.0119	0.0020	-0.0088	0.0093	0.0030	0.0017
rs13132853	A	0.0138	0.0021	-0.0105	0.0112	-0.0096	0.0018
rs13147390	T	-0.0122	0.0020	0.0219	0.0099	0.0034	0.0018
rs13174863	A	-0.0210	0.0028	-0.0098	0.0141	-0.0014	0.0024
rs13199223	T	0.0209	0.0028	0.0313	0.0140	-0.0076	0.0025
rs1320251	T	-0.0168	0.0020	-0.0188	0.0098	0.0038	0.0017

rs1320903	A	0.0229	0.0021	-0.0019	0.0101	-0.0004	0.0018
rs13209689	A	-0.0132	0.0021	-0.0120	0.0098	0.0056	0.0018
rs13209918	A	-0.0134	0.0020	-0.0079	0.0099	0.0018	0.0018
rs13218383	C	0.0135	0.0021	0.0133	0.0096	-0.0014	0.0018
rs13263601	A	-0.0150	0.0020	-0.0115	0.0105	0.0056	0.0018
rs1327259	A	0.0155	0.0020	0.0041	0.0094	-0.0031	0.0017
rs1328448	A	0.0127	0.0021	-0.0125	0.0100	-0.0019	0.0019
rs13288178	A	0.0141	0.0020	0.0175	0.0098	0.0012	0.0018
rs13299074	T	0.0147	0.0025	-0.0059	0.0126	-0.0007	0.0022
rs13427822	A	0.0178	0.0022	0.0284	0.0115	-0.0053	0.0019
rs1348716	A	-0.0117	0.0020	0.0148	0.0095	-0.0127	0.0018
rs13665	C	0.0160	0.0021	-0.0129	0.0100	-0.0041	0.0018
rs1406120	A	-0.0182	0.0031	-0.0250	0.0155	0.0123	0.0027
rs1412235	C	0.0246	0.0021	0.0231	0.0103	0.0013	0.0018
rs1421085	T	-0.0767	0.0020	-0.0297	0.0096	-0.0027	0.0017
rs1421334	A	0.0131	0.0020	0.0144	0.0096	-0.0112	0.0017
rs1431659	A	0.0222	0.0022	0.0010	0.0100	-0.0001	0.0019
rs1436344	C	0.0145	0.0020	0.0072	0.0093	-0.0019	0.0017
rs1454687	C	0.0226	0.0019	0.0068	0.0093	-0.0072	0.0017
rs1460940	A	0.0312	0.0024	0.0310	0.0133	0.0024	0.0022
rs1470153	A	-0.0138	0.0023	0.0212	0.0102	0.0067	0.0020
rs1477890	A	-0.0162	0.0020	0.0049	0.0091	0.0018	0.0017
rs1503526	T	-0.0144	0.0020	-0.0056	0.0094	0.0105	0.0017
rs1522569	T	0.0167	0.0025	-0.0080	0.0125	-0.0055	0.0022
rs1574389	T	0.0161	0.0024	0.0168	0.0107	-0.0031	0.0021
rs1579557	T	0.0225	0.0021	-0.0048	0.0104	-0.0037	0.0019
rs1582931	A	-0.0142	0.0020	-0.0161	0.0095	-0.0074	0.0017
rs1583123	A	0.0124	0.0020	-0.0094	0.0095	-0.0038	0.0018
rs1681740	A	0.0122	0.0020	-0.0040	0.0097	-0.0027	0.0017
rs16868443	C	0.0129	0.0020	-0.0058	0.0098	0.0035	0.0018
rs16882450	A	0.0132	0.0021	0.0210	0.0098	-0.0057	0.0019
rs16916303	A	0.0195	0.0031	0.0100	0.0131	-0.0026	0.0027
rs16973520	T	-0.0138	0.0023	-0.0234	0.0102	0.0045	0.0020
rs16996657	T	-0.0193	0.0029	0.0001	0.0156	0.0012	0.0025
rs17014375	T	-0.0187	0.0029	0.0075	0.0130	0.0004	0.0025
rs1701827	A	0.0145	0.0022	0.0115	0.0108	-0.0028	0.0019
rs17024258	T	0.0648	0.0061	-0.0490	0.0295	0.0039	0.0050
rs17056301	T	-0.0132	0.0023	-0.0131	0.0102	-0.0022	0.0019
rs17066856	T	0.0392	0.0034	0.0379	0.0155	-0.0042	0.0030
rs1711171	T	-0.0185	0.0023	-0.0302	0.0113	0.0029	0.0020
rs17132130	C	-0.0173	0.0024	-0.0218	0.0106	0.0108	0.0020
rs17207196	T	-0.0228	0.0020	-0.0150	0.0111	0.0091	0.0018
rs17379561	A	-0.0181	0.0028	-0.0186	0.0134	0.0190	0.0024
rs17399237	T	0.0120	0.0020	0.0211	0.0100	-0.0051	0.0017
rs17446299	C	-0.0157	0.0026	-0.0097	0.0135	-0.0014	0.0023
rs17513613	T	-0.0201	0.0021	0.0225	0.0104	-0.0010	0.0018
rs17636031	T	-0.0164	0.0022	-0.0026	0.0122	0.0041	0.0019

rs17639546	A	-0.0240	0.0027	-0.0185	0.0131	0.0035	0.0024
rs17724992	A	0.0177	0.0022	0.0139	0.0102	-0.0060	0.0019
rs17776719	A	-0.0171	0.0028	0.0228	0.0122	-0.0016	0.0024
rs17806379	T	-0.0267	0.0025	-0.0060	0.0123	0.0079	0.0022
rs17814208	A	-0.0134	0.0023	0.0111	0.0113	0.0018	0.0020
rs17827419	A	-0.0213	0.0030	-0.0080	0.0159	0.0033	0.0026
rs1786141	T	0.0136	0.0020	0.0184	0.0097	-0.0062	0.0017
rs1814170	A	0.0198	0.0033	-0.0142	0.0129	0.0040	0.0028
rs1861410	T	-0.0219	0.0020	-0.0067	0.0095	0.0031	0.0017
rs1884897	A	-0.0216	0.0020	0.0002	0.0096	-0.0010	0.0018
rs1928538	T	-0.0135	0.0020	-0.0245	0.0093	0.0024	0.0017
rs1951455	T	-0.0150	0.0022	0.0236	0.0107	0.0018	0.0019
rs1955540	T	-0.0154	0.0025	0.0067	0.0114	0.0038	0.0022
rs197374	T	0.0140	0.0020	-0.0112	0.0094	-0.0087	0.0018
rs1976053	C	-0.0186	0.0025	-0.0242	0.0110	0.0034	0.0022
rs2012286	A	0.0139	0.0020	0.0041	0.0094	-0.0044	0.0017
rs2051559	T	-0.0192	0.0029	-0.0111	0.0134	0.0152	0.0025
rs2065418	T	0.0184	0.0020	-0.0043	0.0097	0.0025	0.0018
rs2072858	T	0.0164	0.0021	-0.0085	0.0095	0.0045	0.0018
rs214247	T	0.0136	0.0020	-0.0043	0.0100	-0.0035	0.0018
rs2143253	A	-0.0201	0.0030	-0.0118	0.0125	0.0125	0.0026
rs215634	A	0.0158	0.0020	0.0193	0.0094	-0.0095	0.0018
rs2161796	T	0.0141	0.0020	-0.0018	0.0096	0.0005	0.0018
rs217671	A	-0.0169	0.0022	-0.0082	0.0102	0.0070	0.0019
rs2181375	A	-0.0201	0.0020	-0.0052	0.0093	0.0021	0.0017
rs2183947	A	-0.0162	0.0023	0.0019	0.0115	0.0106	0.0020
rs2253310	C	-0.0169	0.0020	-0.0009	0.0095	-0.0093	0.0018
rs2271189	A	-0.0161	0.0020	-0.0228	0.0100	0.0121	0.0018
rs2281819	A	-0.0162	0.0023	-0.0100	0.0112	0.0065	0.0020
rs2284746	C	0.0112	0.0019	0.0189	0.0094	-0.0026	0.0018
rs2306540	T	0.0137	0.0021	0.0023	0.0108	-0.0031	0.0018
rs2307111	T	0.0276	0.0020	0.0075	0.0095	-0.0052	0.0017
rs2414128	T	0.0131	0.0020	-0.0017	0.0097	0.0026	0.0017
rs241472	T	-0.0190	0.0021	-0.0063	0.0097	0.0021	0.0018
rs2425847	A	-0.0126	0.0020	-0.0038	0.0096	0.0031	0.0017
rs2433733	A	-0.0169	0.0021	0.0086	0.0101	0.0057	0.0018
rs244415	A	-0.0226	0.0020	-0.0265	0.0096	0.0011	0.0017
rs2444253	C	0.0138	0.0020	0.0033	0.0095	-0.0038	0.0017
rs2450444	A	-0.0127	0.0021	-0.0026	0.0101	0.0008	0.0018
rs2472297	T	0.0147	0.0022	-0.0030	0.0129	-0.0002	0.0020
rs2479958	A	0.0158	0.0020	0.0155	0.0097	-0.0021	0.0017
rs248139	A	0.0151	0.0025	-0.0116	0.0115	0.0021	0.0021
rs2591175	A	-0.0144	0.0025	0.0017	0.0117	-0.0013	0.0022
rs2605570	A	0.0151	0.0026	-0.0082	0.0123	-0.0019	0.0023
rs2616143	A	-0.0133	0.0021	0.0039	0.0106	0.0005	0.0018
rs2644112	T	-0.0241	0.0021	-0.0389	0.0100	0.0109	0.0018
rs2693826	A	-0.0136	0.0020	-0.0231	0.0092	0.0022	0.0017

rs2694047	A	-0.0211	0.0023	-0.0088	0.0107	0.0013	0.0020
rs2733287	A	-0.0155	0.0020	-0.0210	0.0091	-0.0067	0.0017
rs273512	T	0.0171	0.0020	0.0011	0.0098	-0.0054	0.0017
rs2781668	T	0.0159	0.0026	0.0217	0.0122	0.0048	0.0023
rs2791643	T	-0.0143	0.0023	0.0033	0.0112	0.0040	0.0020
rs2803316	A	-0.0148	0.0020	0.0156	0.0103	-0.0062	0.0017
rs2814942	A	0.0269	0.0021	0.0233	0.0100	-0.0038	0.0019
rs28350	A	0.0186	0.0026	0.0025	0.0124	0.0022	0.0022
rs2836968	A	0.0122	0.0020	0.0054	0.0102	-0.0052	0.0018
rs2861683	A	0.0171	0.0020	-0.0028	0.0095	0.0003	0.0017
rs2865384	A	-0.0121	0.0020	-0.0104	0.0095	0.0047	0.0018
rs2875762	C	0.0151	0.0023	-0.0035	0.0109	-0.0012	0.0020
rs2906132	T	0.0119	0.0020	0.0102	0.0100	-0.0016	0.0017
rs2920502	C	-0.0147	0.0021	-0.0058	0.0100	0.0030	0.0018
rs294704	T	-0.0146	0.0022	-0.0157	0.0108	-0.0007	0.0019
rs2952862	T	0.0143	0.0021	0.0211	0.0095	-0.0043	0.0018
rs326896	T	-0.0123	0.0020	-0.0138	0.0093	0.0038	0.0017
rs329651	T	0.0169	0.0025	-0.0006	0.0141	-0.0075	0.0022
rs355748	T	0.0162	0.0020	0.0128	0.0093	-0.0037	0.0018
rs3730071	A	-0.0340	0.0057	0.0064	0.0369	-0.0043	0.0052
rs3743950	A	-0.0119	0.0020	0.0048	0.0105	0.0013	0.0018
rs3754963	A	0.0139	0.0022	0.0187	0.0108	-0.0106	0.0019
rs3762444	T	-0.0144	0.0020	-0.0119	0.0097	0.0017	0.0017
rs3770451	T	0.0144	0.0022	-0.0077	0.0109	-0.0104	0.0019
rs3784710	T	0.0287	0.0023	0.0135	0.0107	-0.0005	0.0021
rs3803286	A	0.0193	0.0021	0.0212	0.0098	-0.0070	0.0018
rs3806114	A	-0.0125	0.0021	-0.0092	0.0108	0.0000	0.0018
rs3806572	A	-0.0154	0.0021	0.0200	0.0110	-0.0004	0.0019
rs3814883	T	0.0249	0.0020	-0.0097	0.0099	-0.0005	0.0017
rs3821709	T	-0.0338	0.0039	-0.0133	0.0165	0.0128	0.0035
rs38314	A	-0.0116	0.0020	0.0015	0.0097	-0.0057	0.0017
rs394608	T	-0.0177	0.0020	-0.0170	0.0092	0.0044	0.0017
rs40067	A	-0.0272	0.0026	-0.0389	0.0115	0.0011	0.0023
rs4072917	A	0.0118	0.0020	0.0108	0.0098	-0.0024	0.0017
rs4148155	A	0.0203	0.0031	-0.0238	0.0149	-0.0004	0.0027
rs4148866	T	0.0124	0.0020	-0.0028	0.0100	-0.0072	0.0017
rs4237195	A	0.0177	0.0021	0.0089	0.0098	-0.0046	0.0018
rs4237643	T	0.0237	0.0021	0.0282	0.0103	-0.0024	0.0019
rs4240673	T	0.0201	0.0020	0.0098	0.0100	-0.0026	0.0017
rs4284600	T	-0.0115	0.0020	-0.0005	0.0096	0.0019	0.0017
rs429343	A	0.0172	0.0020	0.0107	0.0097	-0.0006	0.0017
rs4307239	A	-0.0120	0.0020	-0.0017	0.0094	0.0000	0.0017
rs4343164	T	0.0165	0.0022	0.0031	0.0107	-0.0066	0.0019
rs4477562	T	0.0317	0.0030	0.0101	0.0130	-0.0001	0.0026
rs4482463	A	-0.0346	0.0037	0.0028	0.0161	-0.0043	0.0032
rs4490752	A	0.0172	0.0020	-0.0050	0.0097	0.0000	0.0018
rs4516268	A	-0.0241	0.0025	-0.0073	0.0129	0.0096	0.0022

rs4639527	A	-0.0174	0.0021	-0.0018	0.0102	-0.0012	0.0018
rs4652839	C	-0.0132	0.0021	-0.0005	0.0099	0.0027	0.0018
rs4654831	T	0.0188	0.0026	0.0091	0.0114	-0.0021	0.0023
rs4658403	T	-0.0186	0.0026	-0.0098	0.0114	0.0104	0.0023
rs4660443	T	0.0182	0.0024	0.0217	0.0118	-0.0010	0.0021
rs4670169	A	-0.0120	0.0020	-0.0163	0.0096	-0.0056	0.0018
rs4722398	T	0.0178	0.0028	0.0047	0.0139	0.0040	0.0026
rs4762369	A	0.0151	0.0025	0.0255	0.0120	0.0018	0.0022
rs4764949	A	0.0196	0.0021	0.0072	0.0096	0.0010	0.0018
rs4851716	A	0.0162	0.0020	0.0117	0.0101	0.0018	0.0018
rs4911382	T	0.0147	0.0020	0.0039	0.0096	-0.0034	0.0017
rs4936175	T	-0.0124	0.0020	-0.0005	0.0100	0.0036	0.0017
rs4942924	T	0.0158	0.0020	0.0338	0.0096	-0.0022	0.0017
rs4952843	A	0.0151	0.0020	0.0165	0.0099	0.0006	0.0017
rs4969387	C	-0.0146	0.0022	-0.0061	0.0123	-0.0027	0.0020
rs500764	T	0.0164	0.0020	0.0257	0.0093	0.0001	0.0017
rs538656	T	0.0544	0.0023	0.0562	0.0105	0.0056	0.0020
rs538740	T	-0.0145	0.0024	-0.0028	0.0114	0.0074	0.0021
rs5396	T	-0.0175	0.0022	0.0138	0.0103	0.0088	0.0019
rs543874	A	-0.0466	0.0024	-0.0076	0.0117	0.0047	0.0021
rs559231	T	0.0142	0.0020	0.0117	0.0094	0.0015	0.0017
rs577525	T	-0.0187	0.0020	-0.0179	0.0093	0.0066	0.0017
rs592483	T	-0.0152	0.0020	0.0231	0.0102	0.0029	0.0017
rs6011457	A	-0.0116	0.0020	-0.0054	0.0094	-0.0073	0.0017
rs6023649	A	0.0153	0.0023	0.0012	0.0109	-0.0074	0.0019
rs6050446	A	-0.0375	0.0056	-0.0342	0.0320	0.0121	0.0048
rs6098868	A	-0.0117	0.0020	0.0015	0.0092	-0.0010	0.0017
rs6265	T	-0.0405	0.0025	-0.0304	0.0115	0.0097	0.0022
rs6443750	T	-0.0152	0.0025	-0.0190	0.0149	0.0067	0.0022
rs6448587	A	0.0196	0.0026	0.0108	0.0110	-0.0004	0.0022
rs6479905	A	-0.0126	0.0020	0.0066	0.0092	0.0143	0.0017
rs6502482	C	-0.0133	0.0020	-0.0069	0.0094	-0.0041	0.0017
rs6542924	A	-0.0174	0.0021	-0.0163	0.0110	0.0174	0.0018
rs6551304	A	-0.0186	0.0026	-0.0063	0.0133	0.0047	0.0023
rs6577584	T	-0.0131	0.0021	-0.0025	0.0097	0.0023	0.0018
rs6587552	A	0.0175	0.0023	0.0128	0.0108	-0.0076	0.0020
rs6604866	C	-0.0161	0.0020	0.0032	0.0094	0.0003	0.0017
rs6674079	A	-0.0127	0.0020	0.0105	0.0095	0.0055	0.0018
rs6682	A	-0.0121	0.0020	-0.0043	0.0101	0.0038	0.0017
rs671722	A	-0.0126	0.0020	-0.0064	0.0105	0.0093	0.0017
rs6725931	T	0.0187	0.0027	0.0257	0.0134	-0.0115	0.0024
rs6728037	A	0.0198	0.0020	0.0081	0.0098	-0.0046	0.0017
rs6731688	A	-0.0564	0.0026	-0.0372	0.0124	0.0047	0.0023
rs6752378	A	0.0332	0.0019	0.0190	0.0093	0.0012	0.0017
rs6764533	A	0.0141	0.0020	0.0122	0.0101	-0.0073	0.0018
rs6831020	A	-0.0161	0.0021	-0.0056	0.0111	0.0027	0.0019
rs6870983	T	-0.0219	0.0024	-0.0186	0.0110	0.0141	0.0021

rs6905544	A	-0.0164	0.0020	0.0051	0.0097	0.0233	0.0017
rs698147	A	0.0130	0.0020	0.0022	0.0092	-0.0045	0.0017
rs7038943	T	0.0143	0.0021	0.0125	0.0096	-0.0075	0.0018
rs705158	A	0.0162	0.0023	0.0098	0.0103	0.0007	0.0020
rs705217	T	0.0123	0.0020	-0.0008	0.0096	-0.0040	0.0018
rs7084454	A	0.0210	0.0021	0.0035	0.0105	-0.0068	0.0018
rs7096307	T	-0.0117	0.0020	0.0105	0.0098	0.0024	0.0017
rs7102454	T	-0.0176	0.0020	0.0009	0.0104	0.0071	0.0018
rs7124681	A	0.0269	0.0020	-0.0007	0.0096	0.0002	0.0017
rs7132908	A	0.0296	0.0020	0.0107	0.0096	-0.0005	0.0018
rs7158822	T	-0.0162	0.0021	-0.0253	0.0097	0.0034	0.0018
rs7181498	T	0.0160	0.0021	0.0041	0.0097	-0.0052	0.0018
rs7196720	T	0.0131	0.0020	-0.0050	0.0095	-0.0045	0.0017
rs7258722	A	-0.0200	0.0020	-0.0139	0.0099	0.0015	0.0017
rs725959	T	-0.0133	0.0020	-0.0047	0.0096	0.0054	0.0017
rs7313220	A	0.0129	0.0020	-0.0057	0.0092	0.0020	0.0017
rs7334078	T	0.0125	0.0022	-0.0024	0.0104	-0.0103	0.0019
rs733758	T	-0.0313	0.0045	0.0091	0.0236	-0.0068	0.0039
rs738140	A	0.0147	0.0021	0.0037	0.0108	-0.0033	0.0018
rs741959	A	0.0173	0.0020	0.0145	0.0096	-0.0001	0.0017
rs7498665	A	-0.0256	0.0020	-0.0190	0.0101	0.0166	0.0017
rs7518221	T	0.0126	0.0020	-0.0031	0.0098	0.0003	0.0018
rs7519259	A	0.0137	0.0020	0.0196	0.0094	-0.0064	0.0017
rs754635	C	-0.0198	0.0031	-0.0058	0.0134	-0.0032	0.0027
rs7557796	T	0.0171	0.0020	0.0250	0.0099	-0.0030	0.0018
rs756717	A	-0.0158	0.0020	-0.0242	0.0099	-0.0006	0.0017
rs7568228	C	-0.0116	0.0019	-0.0024	0.0097	0.0050	0.0017
rs7619139	A	0.0146	0.0020	0.0086	0.0094	0.0007	0.0017
rs7626079	T	0.0121	0.0021	0.0021	0.0096	0.0052	0.0018
rs7637852	A	0.0143	0.0021	0.0150	0.0103	-0.0013	0.0019
rs7640424	T	-0.0128	0.0021	0.0023	0.0108	0.0107	0.0018
rs7646113	T	-0.0125	0.0020	0.0101	0.0095	0.0010	0.0018
rs765874	A	-0.0125	0.0020	0.0152	0.0093	0.0022	0.0017
rs7683836	A	-0.0135	0.0020	-0.0148	0.0094	0.0003	0.0017
rs769449	A	-0.0235	0.0030	0.0896	0.0165	0.0067	0.0026
rs7707981	A	0.0142	0.0021	-0.0012	0.0106	0.0009	0.0018
rs7715256	T	-0.0165	0.0020	-0.0024	0.0096	-0.0007	0.0017
rs7733438	T	-0.0365	0.0029	-0.0257	0.0129	-0.0173	0.0025
rs774246	A	-0.0169	0.0028	-0.0088	0.0143	0.0012	0.0024
rs7747583	A	-0.0136	0.0022	0.0047	0.0096	0.0074	0.0019
rs775403	T	0.0117	0.0020	0.0045	0.0094	-0.0044	0.0017
rs7792703	A	-0.0166	0.0024	-0.0072	0.0111	0.0010	0.0020
rs7869771	A	0.0156	0.0022	0.0001	0.0102	-0.0047	0.0019
rs7871866	C	0.0177	0.0027	0.0226	0.0128	-0.0062	0.0024
rs7893571	T	0.0146	0.0021	-0.0068	0.0102	0.0024	0.0018
rs7903146	T	-0.0157	0.0022	0.0331	0.0104	-0.0021	0.0019
rs7925214	T	0.0157	0.0020	0.0100	0.0095	-0.0046	0.0017

rs799449	T	0.0141	0.0020	0.0175	0.0097	-0.0070	0.0017
rs8008285	T	-0.0175	0.0028	0.0007	0.0135	-0.0046	0.0024
rs8015400	A	0.0226	0.0021	0.0092	0.0099	-0.0022	0.0018
rs8089514	A	0.0133	0.0021	0.0234	0.0103	-0.0038	0.0018
rs8181823	A	-0.0139	0.0023	-0.0058	0.0111	0.0061	0.0020
rs879620	T	0.0227	0.0020	-0.0029	0.0097	-0.0008	0.0018
rs890156	A	0.0125	0.0020	0.0066	0.0092	0.0023	0.0017
rs891387	T	0.0205	0.0020	0.0085	0.0094	-0.0137	0.0017
rs895330	C	0.0205	0.0025	0.0140	0.0148	-0.0034	0.0022
rs900144	T	0.0174	0.0020	0.0374	0.0095	0.0015	0.0017
rs902503	A	0.0130	0.0020	0.0020	0.0095	-0.0013	0.0017
rs903959	A	0.0116	0.0020	-0.0005	0.0100	-0.0157	0.0017
rs912690	C	-0.0152	0.0020	0.0068	0.0092	0.0030	0.0017
rs9294260	A	0.0158	0.0020	-0.0019	0.0094	-0.0054	0.0017
rs9296389	C	0.0122	0.0020	0.0051	0.0093	-0.0012	0.0017
rs930295	A	0.0214	0.0026	-0.0010	0.0132	0.0000	0.0023
rs9304665	A	0.0225	0.0023	0.0332	0.0106	-0.0003	0.0020
rs942065	A	0.0204	0.0020	0.0043	0.0098	-0.0071	0.0018
rs9446149	T	0.0145	0.0024	0.0137	0.0115	0.0017	0.0021
rs9478496	T	-0.0173	0.0026	-0.0009	0.0131	0.0013	0.0023
rs9496567	A	0.0163	0.0023	-0.0041	0.0119	0.0008	0.0020
rs9515448	A	-0.0141	0.0020	-0.0051	0.0095	0.0066	0.0017
rs9530843	A	0.0134	0.0020	-0.0052	0.0097	-0.0027	0.0017
rs9538162	T	0.0168	0.0020	-0.0070	0.0094	-0.0018	0.0017
rs9571687	A	-0.0126	0.0021	-0.0013	0.0099	-0.0016	0.0018
rs960923	A	-0.0210	0.0020	-0.0004	0.0093	-0.0029	0.0017
rs9630985	A	-0.0173	0.0021	-0.0205	0.0102	0.0058	0.0018
rs9650755	A	-0.0159	0.0022	0.0023	0.0101	0.0069	0.0019
rs9688431	T	0.0242	0.0042	-0.0041	0.0182	0.0000	0.0036
rs9816226	A	-0.0292	0.0025	0.0025	0.0123	-0.0078	0.0022
rs9827823	T	0.0219	0.0027	-0.0149	0.0128	-0.0017	0.0024
rs987237	A	-0.0395	0.0025	-0.0252	0.0116	0.0033	0.0022
rs9876664	T	-0.0181	0.0020	-0.0012	0.0095	0.0139	0.0018
rs9926784	T	0.0262	0.0025	-0.0034	0.0113	0.0032	0.0022
rs9927848	A	-0.0129	0.0022	0.0148	0.0103	-0.0022	0.0019
rs9951619	T	-0.0160	0.0024	-0.0021	0.0107	-0.0052	0.0020
rs9958909	T	-0.0214	0.0029	0.0045	0.0143	0.0027	0.0024

Supplementary Table 5: Genetic association estimates for the two-sample regression-based MVMR analysis estimating the effect of BMI on MI, adjusted for education. ea=effect allele; gx=BMI; gy=MI; gz=education; se=standard error

SNP	ea	gx	gx_se	gy	gy_se	gz	gz_se
rs10083803	T	-0.0139	0.0022	-0.0168	0.0119	-0.0020	0.0019
rs10124645	A	0.0124	0.0020	0.0228	0.0111	-0.0041	0.0017
rs10128597	A	-0.0186	0.0022	-0.0044	0.0111	-0.0004	0.0019
rs1013293	A	-0.0177	0.0020	-0.0256	0.0108	0.0030	0.0017
rs10145154	T	0.0288	0.0024	0.0221	0.0132	0.0002	0.0021
rs10160769	C	-0.0154	0.0024	-0.0154	0.0125	0.0125	0.0021
rs10169594	T	-0.0122	0.0020	-0.0208	0.0110	0.0086	0.0018
rs10206166	T	0.0168	0.0020	0.0366	0.0107	-0.0111	0.0018
rs10236214	T	0.0145	0.0021	-0.0056	0.0117	0.0015	0.0018
rs10259490	A	-0.0129	0.0020	0.0117	0.0104	-0.0023	0.0017
rs10423928	A	-0.0342	0.0025	-0.0355	0.0141	0.0031	0.0021
rs1045411	T	-0.0163	0.0022	-0.0081	0.0126	0.0032	0.0019
rs1048932	A	-0.0149	0.0020	-0.0015	0.0104	0.0041	0.0017
rs10514944	A	0.0247	0.0034	0.0038	0.0164	-0.0074	0.0029
rs10514975	A	-0.0139	0.0020	-0.0210	0.0105	0.0012	0.0017
rs10515237	A	-0.0151	0.0022	-0.0015	0.0115	-0.0003	0.0019
rs1064213	A	0.0146	0.0019	0.0109	0.0103	-0.0026	0.0017
rs10742752	T	-0.0119	0.0020	0.0198	0.0107	-0.0026	0.0017
rs10756714	A	0.0199	0.0020	0.0108	0.0103	-0.0048	0.0017
rs10768994	T	0.0125	0.0020	0.0119	0.0104	-0.0017	0.0017
rs10790519	T	-0.0124	0.0020	-0.0135	0.0107	0.0041	0.0018
rs10818810	A	0.0125	0.0020	0.0218	0.0110	-0.0051	0.0018
rs10824218	A	0.0141	0.0020	0.0078	0.0112	0.0020	0.0017
rs10841188	A	-0.0135	0.0022	-0.0157	0.0113	0.0010	0.0020
rs10842240	C	0.0215	0.0031	0.0101	0.0133	-0.0071	0.0027
rs10878946	T	-0.0148	0.0022	-0.0175	0.0114	-0.0015	0.0019
rs10887578	C	0.0134	0.0020	-0.0042	0.0108	-0.0019	0.0017
rs10899736	A	-0.0116	0.0020	-0.0066	0.0104	-0.0020	0.0017
rs10921743	A	0.0138	0.0020	0.0065	0.0107	-0.0021	0.0018
rs10938397	A	-0.0292	0.0020	-0.0225	0.0103	0.0066	0.0017
rs10962550	C	0.0196	0.0025	0.0226	0.0128	-0.0093	0.0022
rs11075489	T	-0.0115	0.0020	-0.0016	0.0102	0.0023	0.0017
rs11079849	T	-0.0213	0.0021	-0.0505	0.0119	0.0074	0.0018
rs11084553	A	0.0236	0.0027	0.0174	0.0165	0.0045	0.0024
rs11134679	A	-0.0177	0.0021	-0.0007	0.0118	0.0025	0.0018
rs11150461	C	0.0148	0.0022	0.0064	0.0119	0.0017	0.0019
rs11161044	C	0.0175	0.0025	0.0256	0.0123	-0.0180	0.0021
rs11165493	A	0.0143	0.0021	0.0135	0.0111	0.0053	0.0018
rs11190658	T	0.0150	0.0024	0.0044	0.0125	0.0002	0.0021
rs11223970	A	-0.0193	0.0026	-0.0082	0.0147	0.0049	0.0023
rs11251352	A	-0.0117	0.0020	0.0056	0.0105	0.0038	0.0017

rs11496125	T	0.0189	0.0020	-0.0077	0.0105	0.0025	0.0017
rs11505821	A	-0.0301	0.0040	-0.0338	0.0197	-0.0030	0.0036
rs11609297	T	-0.0172	0.0023	-0.0129	0.0134	0.0058	0.0020
rs11611246	T	0.0255	0.0024	0.0095	0.0128	0.0079	0.0021
rs11615578	T	0.0137	0.0023	-0.0052	0.0130	0.0022	0.0020
rs11675464	A	-0.0117	0.0020	0.0351	0.0103	-0.0001	0.0017
rs11713193	A	0.0266	0.0019	0.0270	0.0108	-0.0225	0.0017
rs11757278	T	0.0146	0.0021	-0.0073	0.0113	-0.0073	0.0018
rs11773362	T	-0.0125	0.0021	0.0058	0.0107	0.0033	0.0018
rs11846126	T	0.0158	0.0020	0.0098	0.0105	-0.0037	0.0017
rs11874191	T	-0.0151	0.0023	0.0002	0.0116	0.0069	0.0020
rs11895606	T	0.0131	0.0020	0.0027	0.0105	0.0015	0.0017
rs11915371	A	-0.0148	0.0024	-0.0169	0.0128	0.0102	0.0021
rs11945861	A	-0.0143	0.0023	0.0100	0.0117	0.0068	0.0020
rs11976018	A	-0.0228	0.0027	-0.0102	0.0129	0.0041	0.0023
rs12049202	T	0.0245	0.0024	0.0270	0.0131	-0.0067	0.0021
rs1218822	A	0.0176	0.0021	0.0063	0.0110	-0.0024	0.0018
rs12334877	A	-0.0152	0.0025	-0.0214	0.0123	0.0047	0.0021
rs12369179	T	-0.0372	0.0034	0.0076	0.0219	-0.0143	0.0030
rs12427047	T	-0.0184	0.0023	-0.0007	0.0122	-0.0041	0.0020
rs12439798	T	0.0139	0.0020	-0.0057	0.0104	-0.0038	0.0017
rs12448257	A	0.0157	0.0024	0.0073	0.0126	0.0074	0.0021
rs12512994	T	-0.0127	0.0021	-0.0116	0.0110	0.0140	0.0018
rs12564848	T	-0.0229	0.0031	0.0060	0.0152	-0.0015	0.0027
rs12595749	A	0.0149	0.0020	-0.0141	0.0105	-0.0022	0.0017
rs12608738	A	0.0130	0.0022	-0.0041	0.0130	-0.0020	0.0019
rs1266874	A	-0.0147	0.0020	-0.0098	0.0106	0.0023	0.0018
rs12681792	A	0.0149	0.0025	-0.0010	0.0128	-0.0034	0.0021
rs12705977	T	0.0134	0.0020	0.0114	0.0106	-0.0079	0.0017
rs12762034	T	-0.0262	0.0037	-0.0095	0.0178	0.0059	0.0032
rs12878246	T	0.0137	0.0023	0.0142	0.0132	-0.0017	0.0020
rs12906208	A	-0.0149	0.0024	0.0095	0.0139	0.0039	0.0021
rs12936083	A	-0.0137	0.0021	-0.0114	0.0110	0.0014	0.0018
rs12936319	A	0.0167	0.0020	-0.0004	0.0106	-0.0072	0.0017
rs12949279	T	0.0180	0.0020	0.0129	0.0106	-0.0031	0.0017
rs1296328	A	0.0190	0.0020	0.0020	0.0108	-0.0024	0.0017
rs12981256	A	0.0140	0.0020	0.0159	0.0112	-0.0021	0.0017
rs13034320	C	0.0136	0.0023	0.0163	0.0117	-0.0012	0.0020
rs13047416	C	0.0143	0.0020	-0.0031	0.0107	0.0015	0.0018
rs13085002	T	-0.0168	0.0027	-0.0082	0.0137	0.0009	0.0023
rs13107325	T	0.0469	0.0037	-0.0015	0.0244	-0.0188	0.0034
rs13110266	A	-0.0119	0.0020	-0.0064	0.0103	0.0030	0.0017
rs13132853	A	0.0138	0.0021	0.0059	0.0122	-0.0096	0.0018
rs13147390	T	-0.0122	0.0020	0.0132	0.0110	0.0034	0.0018
rs13174863	A	-0.0210	0.0028	-0.0139	0.0157	-0.0014	0.0024
rs13199223	T	0.0209	0.0028	0.0280	0.0152	-0.0076	0.0025

rs1320251	T	-0.0168	0.0020	-0.0133	0.0109	0.0038	0.0017
rs1320903	A	0.0229	0.0021	0.0018	0.0112	-0.0004	0.0018
rs13209689	A	-0.0132	0.0021	-0.0051	0.0108	0.0056	0.0018
rs13209918	A	-0.0134	0.0020	-0.0140	0.0109	0.0018	0.0018
rs13218383	C	0.0135	0.0021	0.0202	0.0107	-0.0014	0.0018
rs13263601	A	-0.0150	0.0020	-0.0001	0.0116	0.0056	0.0018
rs1327259	A	0.0155	0.0020	0.0024	0.0104	-0.0031	0.0017
rs1328448	A	0.0127	0.0021	-0.0093	0.0112	-0.0019	0.0019
rs13288178	A	0.0141	0.0020	0.0278	0.0108	0.0012	0.0018
rs13299074	T	0.0147	0.0025	0.0023	0.0140	-0.0007	0.0022
rs13427822	A	0.0178	0.0022	0.0129	0.0124	-0.0053	0.0019
rs1348716	A	-0.0117	0.0020	0.0091	0.0105	-0.0127	0.0018
rs13665	C	0.0160	0.0021	-0.0308	0.0110	-0.0041	0.0018
rs1406120	A	-0.0182	0.0031	-0.0236	0.0168	0.0123	0.0027
rs1412235	C	0.0246	0.0021	0.0279	0.0114	0.0013	0.0018
rs1421085	T	-0.0767	0.0020	-0.0266	0.0107	-0.0027	0.0017
rs1421334	A	0.0131	0.0020	0.0161	0.0106	-0.0112	0.0017
rs1431659	A	0.0222	0.0022	0.0061	0.0112	-0.0001	0.0019
rs1436344	C	0.0145	0.0020	0.0039	0.0103	-0.0019	0.0017
rs1454687	C	0.0226	0.0019	0.0033	0.0103	-0.0072	0.0017
rs1460940	A	0.0312	0.0024	0.0189	0.0147	0.0024	0.0022
rs1470153	A	-0.0138	0.0023	0.0262	0.0114	0.0067	0.0020
rs1477890	A	-0.0162	0.0020	-0.0014	0.0101	0.0018	0.0017
rs1503526	T	-0.0144	0.0020	-0.0097	0.0104	0.0105	0.0017
rs1522569	T	0.0167	0.0025	-0.0087	0.0137	-0.0055	0.0022
rs1574389	T	0.0161	0.0024	0.0176	0.0120	-0.0031	0.0021
rs1579557	T	0.0225	0.0021	-0.0021	0.0117	-0.0037	0.0019
rs1582931	A	-0.0142	0.0020	-0.0217	0.0106	-0.0074	0.0017
rs1583123	A	0.0124	0.0020	-0.0129	0.0106	-0.0038	0.0018
rs1681740	A	0.0122	0.0020	-0.0040	0.0107	-0.0027	0.0017
rs16868443	C	0.0129	0.0020	-0.0081	0.0108	0.0035	0.0018
rs16882450	A	0.0132	0.0021	0.0202	0.0109	-0.0057	0.0019
rs16916303	A	0.0195	0.0031	0.0184	0.0148	-0.0026	0.0027
rs16973520	T	-0.0138	0.0023	-0.0316	0.0114	0.0045	0.0020
rs16996657	T	-0.0193	0.0029	-0.0222	0.0173	0.0012	0.0025
rs17014375	T	-0.0187	0.0029	0.0056	0.0143	0.0004	0.0025
rs1701827	A	0.0145	0.0022	0.0106	0.0122	-0.0028	0.0019
rs17024258	T	0.0648	0.0061	-0.0360	0.0331	0.0039	0.0050
rs17056301	T	-0.0132	0.0023	-0.0167	0.0112	-0.0022	0.0019
rs17066856	T	0.0392	0.0034	0.0357	0.0174	-0.0042	0.0030
rs1711171	T	-0.0185	0.0023	-0.0274	0.0124	0.0029	0.0020
rs17132130	C	-0.0173	0.0024	-0.0281	0.0117	0.0108	0.0020
rs17207196	T	-0.0228	0.0020	-0.0126	0.0121	0.0091	0.0018
rs17379561	A	-0.0181	0.0028	-0.0218	0.0148	0.0190	0.0024
rs17399237	T	0.0120	0.0020	0.0108	0.0110	-0.0051	0.0017
rs17446299	C	-0.0157	0.0026	-0.0176	0.0149	-0.0014	0.0023

rs17513613	T	-0.0201	0.0021	0.0123	0.0114	-0.0010	0.0018
rs17636031	T	-0.0164	0.0022	0.0130	0.0133	0.0041	0.0019
rs17639546	A	-0.0240	0.0027	-0.0297	0.0146	0.0035	0.0024
rs17724992	A	0.0177	0.0022	0.0103	0.0113	-0.0060	0.0019
rs17776719	A	-0.0171	0.0028	0.0249	0.0135	-0.0016	0.0024
rs17806379	T	-0.0267	0.0025	-0.0097	0.0135	0.0079	0.0022
rs17814208	A	-0.0134	0.0023	0.0000	0.0121	0.0018	0.0020
rs17827419	A	-0.0213	0.0030	-0.0107	0.0173	0.0033	0.0026
rs1786141	T	0.0136	0.0020	0.0089	0.0108	-0.0062	0.0017
rs1814170	A	0.0198	0.0033	-0.0059	0.0145	0.0040	0.0028
rs1861410	T	-0.0219	0.0020	-0.0095	0.0102	0.0031	0.0017
rs1884897	A	-0.0216	0.0020	-0.0058	0.0107	-0.0010	0.0018
rs1928538	T	-0.0135	0.0020	-0.0368	0.0103	0.0024	0.0017
rs1951455	T	-0.0150	0.0022	0.0158	0.0119	0.0018	0.0019
rs1955540	T	-0.0154	0.0025	0.0039	0.0129	0.0038	0.0022
rs197374	T	0.0140	0.0020	-0.0092	0.0105	-0.0087	0.0018
rs1976053	C	-0.0186	0.0025	-0.0364	0.0124	0.0034	0.0022
rs2012286	A	0.0139	0.0020	-0.0010	0.0104	-0.0044	0.0017
rs2051559	T	-0.0192	0.0029	-0.0177	0.0150	0.0152	0.0025
rs2065418	T	0.0184	0.0020	-0.0043	0.0108	0.0025	0.0018
rs2072858	T	0.0164	0.0021	-0.0057	0.0106	0.0045	0.0018
rs214247	T	0.0136	0.0020	0.0027	0.0112	-0.0035	0.0018
rs2143253	A	-0.0201	0.0030	0.0023	0.0138	0.0125	0.0026
rs215634	A	0.0158	0.0020	0.0242	0.0106	-0.0095	0.0018
rs2161796	T	0.0141	0.0020	0.0005	0.0106	0.0005	0.0018
rs217671	A	-0.0169	0.0022	-0.0053	0.0113	0.0070	0.0019
rs2181375	A	-0.0201	0.0020	-0.0077	0.0102	0.0021	0.0017
rs2183947	A	-0.0162	0.0023	0.0160	0.0127	0.0106	0.0020
rs2253310	C	-0.0169	0.0020	-0.0209	0.0106	-0.0093	0.0018
rs2271189	A	-0.0161	0.0020	-0.0298	0.0114	0.0121	0.0018
rs2281819	A	-0.0162	0.0023	-0.0045	0.0124	0.0065	0.0020
rs2284746	C	0.0112	0.0019	0.0174	0.0104	-0.0026	0.0018
rs2306540	T	0.0137	0.0021	-0.0037	0.0120	-0.0031	0.0018
rs2307111	T	0.0276	0.0020	0.0086	0.0105	-0.0052	0.0017
rs2414128	T	0.0131	0.0020	0.0093	0.0110	0.0026	0.0017
rs241472	T	-0.0190	0.0021	-0.0042	0.0108	0.0021	0.0018
rs2425847	A	-0.0126	0.0020	0.0024	0.0106	0.0031	0.0017
rs2433733	A	-0.0169	0.0021	0.0108	0.0108	0.0057	0.0018
rs244415	A	-0.0226	0.0020	-0.0185	0.0107	0.0011	0.0017
rs2444253	C	0.0138	0.0020	0.0060	0.0105	-0.0038	0.0017
rs2450444	A	-0.0127	0.0021	-0.0065	0.0114	0.0008	0.0018
rs2472297	T	0.0147	0.0022	-0.0120	0.0146	-0.0002	0.0020
rs2479958	A	0.0158	0.0020	0.0172	0.0108	-0.0021	0.0017
rs248139	A	0.0151	0.0025	-0.0132	0.0129	0.0021	0.0021
rs2591175	A	-0.0144	0.0025	-0.0098	0.0127	-0.0013	0.0022
rs2605570	A	0.0151	0.0026	-0.0071	0.0136	-0.0019	0.0023

rs2616143	A	-0.0133	0.0021	0.0108	0.0119	0.0005	0.0018
rs2644112	T	-0.0241	0.0021	-0.0429	0.0111	0.0109	0.0018
rs2693826	A	-0.0136	0.0020	-0.0162	0.0103	0.0022	0.0017
rs2694047	A	-0.0211	0.0023	-0.0135	0.0119	0.0013	0.0020
rs2733287	A	-0.0155	0.0020	-0.0198	0.0102	-0.0067	0.0017
rs273512	T	0.0171	0.0020	-0.0112	0.0109	-0.0054	0.0017
rs2781668	T	0.0159	0.0026	0.0354	0.0136	0.0048	0.0023
rs2791643	T	-0.0143	0.0023	0.0033	0.0123	0.0040	0.0020
rs2803316	A	-0.0148	0.0020	-0.0023	0.0115	-0.0062	0.0017
rs2814942	A	0.0269	0.0021	0.0332	0.0111	-0.0038	0.0019
rs28350	A	0.0186	0.0026	0.0004	0.0138	0.0022	0.0022
rs2836968	A	0.0122	0.0020	0.0098	0.0112	-0.0052	0.0018
rs2861683	A	0.0171	0.0020	-0.0054	0.0103	0.0003	0.0017
rs2865384	A	-0.0121	0.0020	-0.0149	0.0105	0.0047	0.0018
rs2875762	C	0.0151	0.0023	-0.0029	0.0120	-0.0012	0.0020
rs2906132	T	0.0119	0.0020	0.0133	0.0110	-0.0016	0.0017
rs2920502	C	-0.0147	0.0021	-0.0111	0.0110	0.0030	0.0018
rs294704	T	-0.0146	0.0022	-0.0136	0.0119	-0.0007	0.0019
rs2952862	T	0.0143	0.0021	0.0204	0.0106	-0.0043	0.0018
rs326896	T	-0.0123	0.0020	-0.0176	0.0103	0.0038	0.0017
rs329651	T	0.0169	0.0025	0.0008	0.0154	-0.0075	0.0022
rs355748	T	0.0162	0.0020	0.0046	0.0104	-0.0037	0.0018
rs3730071	A	-0.0340	0.0057	0.0353	0.0415	-0.0043	0.0052
rs3743950	A	-0.0119	0.0020	0.0031	0.0116	0.0013	0.0018
rs3754963	A	0.0139	0.0022	0.0113	0.0117	-0.0106	0.0019
rs3762444	T	-0.0144	0.0020	-0.0233	0.0107	0.0017	0.0017
rs3770451	T	0.0144	0.0022	-0.0084	0.0118	-0.0104	0.0019
rs3784710	T	0.0287	0.0023	0.0156	0.0119	-0.0005	0.0021
rs3803286	A	0.0193	0.0021	0.0137	0.0110	-0.0070	0.0018
rs3806114	A	-0.0125	0.0021	-0.0062	0.0121	0.0000	0.0018
rs3806572	A	-0.0154	0.0021	0.0108	0.0117	-0.0004	0.0019
rs3814883	T	0.0249	0.0020	-0.0085	0.0111	-0.0005	0.0017
rs3821709	T	-0.0338	0.0039	-0.0183	0.0184	0.0128	0.0035
rs38314	A	-0.0116	0.0020	-0.0004	0.0108	-0.0057	0.0017
rs394608	T	-0.0177	0.0020	-0.0139	0.0102	0.0044	0.0017
rs40067	A	-0.0272	0.0026	-0.0322	0.0126	0.0011	0.0023
rs4072917	A	0.0118	0.0020	0.0068	0.0109	-0.0024	0.0017
rs4148155	A	0.0203	0.0031	-0.0218	0.0168	-0.0004	0.0027
rs4148866	T	0.0124	0.0020	-0.0003	0.0113	-0.0072	0.0017
rs4237195	A	0.0177	0.0021	0.0098	0.0110	-0.0046	0.0018
rs4237643	T	0.0237	0.0021	0.0370	0.0113	-0.0024	0.0019
rs4240673	T	0.0201	0.0020	0.0007	0.0110	-0.0026	0.0017
rs4284600	T	-0.0115	0.0020	0.0063	0.0105	0.0019	0.0017
rs429343	A	0.0172	0.0020	0.0139	0.0104	-0.0006	0.0017
rs4307239	A	-0.0120	0.0020	-0.0089	0.0105	0.0000	0.0017
rs4343164	T	0.0165	0.0022	-0.0007	0.0120	-0.0066	0.0019

rs4477562	T	0.0317	0.0030	0.0134	0.0145	-0.0001	0.0026
rs4482463	A	-0.0346	0.0037	0.0038	0.0178	-0.0043	0.0032
rs4490752	A	0.0172	0.0020	-0.0027	0.0108	0.0000	0.0018
rs4516268	A	-0.0241	0.0025	0.0092	0.0145	0.0096	0.0022
rs4639527	A	-0.0174	0.0021	-0.0035	0.0114	-0.0012	0.0018
rs4652839	C	-0.0132	0.0021	-0.0017	0.0109	0.0027	0.0018
rs4654831	T	0.0188	0.0026	-0.0003	0.0125	-0.0021	0.0023
rs4658403	T	-0.0186	0.0026	-0.0040	0.0127	0.0104	0.0023
rs4660443	T	0.0182	0.0024	0.0169	0.0131	-0.0010	0.0021
rs4670169	A	-0.0120	0.0020	-0.0104	0.0107	-0.0056	0.0018
rs4722398	T	0.0178	0.0028	-0.0086	0.0151	0.0040	0.0026
rs4762369	A	0.0151	0.0025	0.0280	0.0134	0.0018	0.0022
rs4764949	A	0.0196	0.0021	0.0045	0.0107	0.0010	0.0018
rs4851716	A	0.0162	0.0020	0.0030	0.0108	0.0018	0.0018
rs4911382	T	0.0147	0.0020	0.0088	0.0106	-0.0034	0.0017
rs4936175	T	-0.0124	0.0020	0.0001	0.0112	0.0036	0.0017
rs4942924	T	0.0158	0.0020	0.0428	0.0107	-0.0022	0.0017
rs4952843	A	0.0151	0.0020	0.0192	0.0109	0.0006	0.0017
rs4969387	C	-0.0146	0.0022	-0.0033	0.0138	-0.0027	0.0020
rs500764	T	0.0164	0.0020	0.0273	0.0103	0.0001	0.0017
rs538656	T	0.0544	0.0023	0.0520	0.0116	0.0056	0.0020
rs538740	T	-0.0145	0.0024	-0.0097	0.0128	0.0074	0.0021
rs5396	T	-0.0175	0.0022	0.0269	0.0115	0.0088	0.0019
rs543874	A	-0.0466	0.0024	-0.0238	0.0132	0.0047	0.0021
rs559231	T	0.0142	0.0020	0.0170	0.0104	0.0015	0.0017
rs577525	T	-0.0187	0.0020	-0.0181	0.0104	0.0066	0.0017
rs592483	T	-0.0152	0.0020	0.0137	0.0113	0.0029	0.0017
rs6011457	A	-0.0116	0.0020	0.0049	0.0104	-0.0073	0.0017
rs6023649	A	0.0153	0.0023	0.0137	0.0121	-0.0074	0.0019
rs6050446	A	-0.0375	0.0056	-0.0113	0.0350	0.0121	0.0048
rs6098868	A	-0.0117	0.0020	0.0041	0.0101	-0.0010	0.0017
rs6265	T	-0.0405	0.0025	-0.0303	0.0129	0.0097	0.0022
rs6443750	T	-0.0152	0.0025	-0.0286	0.0169	0.0067	0.0022
rs6448587	A	0.0196	0.0026	0.0098	0.0122	-0.0004	0.0022
rs6479905	A	-0.0126	0.0020	0.0026	0.0101	0.0143	0.0017
rs6502482	C	-0.0133	0.0020	-0.0031	0.0103	-0.0041	0.0017
rs6542924	A	-0.0174	0.0021	-0.0094	0.0119	0.0174	0.0018
rs6551304	A	-0.0186	0.0026	-0.0074	0.0148	0.0047	0.0023
rs6577584	T	-0.0131	0.0021	0.0065	0.0108	0.0023	0.0018
rs6587552	A	0.0175	0.0023	0.0056	0.0121	-0.0076	0.0020
rs6604866	C	-0.0161	0.0020	0.0045	0.0104	0.0003	0.0017
rs6674079	A	-0.0127	0.0020	0.0048	0.0105	0.0055	0.0018
rs6682	A	-0.0121	0.0020	-0.0131	0.0114	0.0038	0.0017
rs671722	A	-0.0126	0.0020	-0.0080	0.0113	0.0093	0.0017
rs6725931	T	0.0187	0.0027	0.0226	0.0140	-0.0115	0.0024
rs6728037	A	0.0198	0.0020	0.0101	0.0106	-0.0046	0.0017

rs6731688	A	-0.0564	0.0026	-0.0143	0.0136	0.0047	0.0023
rs6752378	A	0.0332	0.0019	0.0329	0.0102	0.0012	0.0017
rs6764533	A	0.0141	0.0020	0.0138	0.0112	-0.0073	0.0018
rs6831020	A	-0.0161	0.0021	-0.0085	0.0122	0.0027	0.0019
rs6870983	T	-0.0219	0.0024	-0.0143	0.0120	0.0141	0.0021
rs6905544	A	-0.0164	0.0020	-0.0036	0.0107	0.0233	0.0017
rs698147	A	0.0130	0.0020	0.0063	0.0102	-0.0045	0.0017
rs7038943	T	0.0143	0.0021	0.0209	0.0107	-0.0075	0.0018
rs705158	A	0.0162	0.0023	-0.0014	0.0114	0.0007	0.0020
rs705217	T	0.0123	0.0020	-0.0048	0.0106	-0.0040	0.0018
rs7084454	A	0.0210	0.0021	0.0018	0.0115	-0.0068	0.0018
rs7096307	T	-0.0117	0.0020	0.0101	0.0108	0.0024	0.0017
rs7102454	T	-0.0176	0.0020	-0.0130	0.0116	0.0071	0.0018
rs7124681	A	0.0269	0.0020	-0.0092	0.0108	0.0002	0.0017
rs7132908	A	0.0296	0.0020	0.0041	0.0107	-0.0005	0.0018
rs7158822	T	-0.0162	0.0021	-0.0184	0.0107	0.0034	0.0018
rs7181498	T	0.0160	0.0021	0.0053	0.0108	-0.0052	0.0018
rs7196720	T	0.0131	0.0020	-0.0088	0.0106	-0.0045	0.0017
rs7258722	A	-0.0200	0.0020	-0.0252	0.0109	0.0015	0.0017
rs725959	T	-0.0133	0.0020	-0.0169	0.0108	0.0054	0.0017
rs7313220	A	0.0129	0.0020	-0.0068	0.0102	0.0020	0.0017
rs7334078	T	0.0125	0.0022	0.0059	0.0116	-0.0103	0.0019
rs733758	T	-0.0313	0.0045	0.0066	0.0260	-0.0068	0.0039
rs738140	A	0.0147	0.0021	-0.0050	0.0118	-0.0033	0.0018
rs741959	A	0.0173	0.0020	0.0100	0.0106	-0.0001	0.0017
rs7498665	A	-0.0256	0.0020	-0.0180	0.0113	0.0166	0.0017
rs7518221	T	0.0126	0.0020	0.0062	0.0108	0.0003	0.0018
rs7519259	A	0.0137	0.0020	0.0148	0.0104	-0.0064	0.0017
rs754635	C	-0.0198	0.0031	0.0026	0.0152	-0.0032	0.0027
rs7557796	T	0.0171	0.0020	0.0265	0.0106	-0.0030	0.0018
rs756717	A	-0.0158	0.0020	-0.0209	0.0112	-0.0006	0.0017
rs7568228	C	-0.0116	0.0019	-0.0120	0.0105	0.0050	0.0017
rs7619139	A	0.0146	0.0020	0.0115	0.0104	0.0007	0.0017
rs7626079	T	0.0121	0.0021	-0.0016	0.0107	0.0052	0.0018
rs7637852	A	0.0143	0.0021	0.0195	0.0114	-0.0013	0.0019
rs7640424	T	-0.0128	0.0021	-0.0074	0.0119	0.0107	0.0018
rs7646113	T	-0.0125	0.0020	0.0117	0.0106	0.0010	0.0018
rs765874	A	-0.0125	0.0020	0.0188	0.0104	0.0022	0.0017
rs7683836	A	-0.0135	0.0020	-0.0086	0.0104	0.0003	0.0017
rs769449	A	-0.0235	0.0030	0.0855	0.0183	0.0067	0.0026
rs7707981	A	0.0142	0.0021	-0.0128	0.0117	0.0009	0.0018
rs7715256	T	-0.0165	0.0020	-0.0047	0.0105	-0.0007	0.0017
rs7733438	T	-0.0365	0.0029	-0.0278	0.0142	-0.0173	0.0025
rs774246	A	-0.0169	0.0028	0.0087	0.0161	0.0012	0.0024
rs7747583	A	-0.0136	0.0022	0.0046	0.0108	0.0074	0.0019
rs775403	T	0.0117	0.0020	0.0054	0.0104	-0.0044	0.0017

rs7792703	A	-0.0166	0.0024	-0.0133	0.0122	0.0010	0.0020
rs7869771	A	0.0156	0.0022	0.0124	0.0114	-0.0047	0.0019
rs7871866	C	0.0177	0.0027	0.0155	0.0145	-0.0062	0.0024
rs7893571	T	0.0146	0.0021	0.0012	0.0111	0.0024	0.0018
rs7903146	T	-0.0157	0.0022	0.0241	0.0114	-0.0021	0.0019
rs7925214	T	0.0157	0.0020	0.0162	0.0106	-0.0046	0.0017
rs799449	T	0.0141	0.0020	0.0227	0.0108	-0.0070	0.0017
rs8008285	T	-0.0175	0.0028	0.0076	0.0150	-0.0046	0.0024
rs8015400	A	0.0226	0.0021	-0.0017	0.0110	-0.0022	0.0018
rs8089514	A	0.0133	0.0021	0.0203	0.0115	-0.0038	0.0018
rs8181823	A	-0.0139	0.0023	-0.0042	0.0125	0.0061	0.0020
rs879620	T	0.0227	0.0020	0.0013	0.0110	-0.0008	0.0018
rs890156	A	0.0125	0.0020	0.0004	0.0103	0.0023	0.0017
rs891387	T	0.0205	0.0020	0.0173	0.0104	-0.0137	0.0017
rs895330	C	0.0205	0.0025	0.0371	0.0168	-0.0034	0.0022
rs900144	T	0.0174	0.0020	0.0227	0.0105	0.0015	0.0017
rs902503	A	0.0130	0.0020	0.0003	0.0105	-0.0013	0.0017
rs903959	A	0.0116	0.0020	-0.0169	0.0111	-0.0157	0.0017
rs912690	C	-0.0152	0.0020	0.0146	0.0102	0.0030	0.0017
rs9294260	A	0.0158	0.0020	-0.0028	0.0103	-0.0054	0.0017
rs9296389	C	0.0122	0.0020	0.0107	0.0103	-0.0012	0.0017
rs930295	A	0.0214	0.0026	0.0112	0.0142	0.0000	0.0023
rs9304665	A	0.0225	0.0023	0.0241	0.0119	-0.0003	0.0020
rs942065	A	0.0204	0.0020	0.0150	0.0108	-0.0071	0.0018
rs9446149	T	0.0145	0.0024	0.0119	0.0128	0.0017	0.0021
rs9478496	T	-0.0173	0.0026	-0.0178	0.0145	0.0013	0.0023
rs9496567	A	0.0163	0.0023	0.0004	0.0130	0.0008	0.0020
rs9515448	A	-0.0141	0.0020	-0.0008	0.0105	0.0066	0.0017
rs9530843	A	0.0134	0.0020	-0.0050	0.0107	-0.0027	0.0017
rs9538162	T	0.0168	0.0020	-0.0082	0.0104	-0.0018	0.0017
rs9571687	A	-0.0126	0.0021	0.0035	0.0110	-0.0016	0.0018
rs960923	A	-0.0210	0.0020	0.0064	0.0102	-0.0029	0.0017
rs9630985	A	-0.0173	0.0021	-0.0214	0.0110	0.0058	0.0018
rs9650755	A	-0.0159	0.0022	-0.0003	0.0112	0.0069	0.0019
rs9688431	T	0.0242	0.0042	-0.0092	0.0209	0.0000	0.0036
rs9816226	A	-0.0292	0.0025	0.0009	0.0134	-0.0078	0.0022
rs9827823	T	0.0219	0.0027	-0.0225	0.0141	-0.0017	0.0024
rs987237	A	-0.0395	0.0025	-0.0177	0.0130	0.0033	0.0022
rs9876664	T	-0.0181	0.0020	-0.0014	0.0105	0.0139	0.0018
rs9926784	T	0.0262	0.0025	0.0041	0.0128	0.0032	0.0022
rs9927848	A	-0.0129	0.0022	0.0177	0.0115	-0.0022	0.0019
rs9951619	T	-0.0160	0.0024	-0.0103	0.0119	-0.0052	0.0020
rs9958909	T	-0.0214	0.0029	-0.0123	0.0157	0.0027	0.0024

Supplementary Table 6: Genetic association estimates for the two-sample regression-based MVMR analysis estimating the effect of BMI on stroke, adjusted for education. ea=effect allele; gx=BMI; gy=stroke; gz=education; se=standard error

SNP	ea	gx	gx_se	gy	gy_se	gz	gz_se
rs10083803	T	-0.0139	0.0022	-0.0112	0.0089	-0.0020	0.0019
rs10124645	A	0.0124	0.0020	0.0094	0.0088	-0.0041	0.0017
rs10128597	A	-0.0186	0.0022	-0.0061	0.0087	-0.0004	0.0019
rs1013293	A	-0.0177	0.0020	-0.0050	0.0087	0.0030	0.0017
rs10145154	T	0.0288	0.0024	0.0139	0.0102	0.0002	0.0021
rs10160769	C	-0.0154	0.0024	-0.0120	0.0093	0.0125	0.0021
rs10169594	T	-0.0122	0.0020	-0.0175	0.0091	0.0086	0.0018
rs10206166	T	0.0168	0.0020	0.0089	0.0081	-0.0111	0.0018
rs10236214	T	0.0145	0.0021	-0.0288	0.0090	0.0015	0.0018
rs10259490	A	-0.0129	0.0020	-0.0030	0.0080	-0.0023	0.0017
rs10423928	A	-0.0342	0.0025	0.0034	0.0098	0.0031	0.0021
rs1045411	T	-0.0163	0.0022	-0.0125	0.0096	0.0032	0.0019
rs1048932	A	-0.0149	0.0020	-0.0034	0.0079	0.0041	0.0017
rs10514944	A	0.0247	0.0034	-0.0106	0.0124	-0.0074	0.0029
rs10514975	A	-0.0139	0.0020	0.0120	0.0080	0.0012	0.0017
rs10515237	A	-0.0151	0.0022	-0.0078	0.0087	-0.0003	0.0019
rs1064213	A	0.0146	0.0019	0.0033	0.0081	-0.0026	0.0017
rs10742752	T	-0.0119	0.0020	-0.0068	0.0081	-0.0026	0.0017
rs10756714	A	0.0199	0.0020	0.0023	0.0080	-0.0048	0.0017
rs10768994	T	0.0125	0.0020	0.0004	0.0078	-0.0017	0.0017
rs10790519	T	-0.0124	0.0020	0.0023	0.0079	0.0041	0.0018
rs10818810	A	0.0125	0.0020	-0.0016	0.0082	-0.0051	0.0018
rs10824218	A	0.0141	0.0020	0.0033	0.0085	0.0020	0.0017
rs10841188	A	-0.0135	0.0022	-0.0048	0.0088	0.0010	0.0020
rs10842240	C	0.0215	0.0031	-0.0081	0.0108	-0.0071	0.0027
rs10878946	T	-0.0148	0.0022	0.0105	0.0089	-0.0015	0.0019
rs10887578	C	0.0134	0.0020	-0.0143	0.0081	-0.0019	0.0017
rs10899736	A	-0.0116	0.0020	-0.0016	0.0078	-0.0020	0.0017
rs10921743	A	0.0138	0.0020	0.0094	0.0083	-0.0021	0.0018
rs10938397	A	-0.0292	0.0020	-0.0112	0.0083	0.0066	0.0017
rs10962550	C	0.0196	0.0025	-0.0043	0.0098	-0.0093	0.0022
rs11075489	T	-0.0115	0.0020	-0.0069	0.0081	0.0023	0.0017
rs11079849	T	-0.0213	0.0021	-0.0058	0.0094	0.0074	0.0018
rs11084553	A	0.0236	0.0027	0.0000	0.0129	0.0045	0.0024
rs11134679	A	-0.0177	0.0021	-0.0131	0.0091	0.0025	0.0018
rs11150461	C	0.0148	0.0022	0.0097	0.0090	0.0017	0.0019
rs11161044	C	0.0175	0.0025	0.0071	0.0094	-0.0180	0.0021
rs11165493	A	0.0143	0.0021	-0.0132	0.0095	0.0053	0.0018
rs11190658	T	0.0150	0.0024	0.0100	0.0095	0.0002	0.0021
rs11223970	A	-0.0193	0.0026	0.0048	0.0126	0.0049	0.0023
rs11251352	A	-0.0117	0.0020	0.0088	0.0081	0.0038	0.0017

rs11496125	T	0.0189	0.0020	-0.0053	0.0079	0.0025	0.0017
rs11505821	A	-0.0301	0.0040	0.0137	0.0156	-0.0030	0.0036
rs11609297	T	-0.0172	0.0023	-0.0010	0.0106	0.0058	0.0020
rs11611246	T	0.0255	0.0024	-0.0014	0.0097	0.0079	0.0021
rs11615578	T	0.0137	0.0023	-0.0047	0.0104	0.0022	0.0020
rs11675464	A	-0.0117	0.0020	0.0126	0.0081	-0.0001	0.0017
rs11713193	A	0.0266	0.0019	0.0168	0.0084	-0.0225	0.0017
rs11757278	T	0.0146	0.0021	0.0058	0.0084	-0.0073	0.0018
rs11773362	T	-0.0125	0.0021	-0.0104	0.0082	0.0033	0.0018
rs11846126	T	0.0158	0.0020	0.0147	0.0081	-0.0037	0.0017
rs11874191	T	-0.0151	0.0023	0.0167	0.0088	0.0069	0.0020
rs11895606	T	0.0131	0.0020	0.0087	0.0086	0.0015	0.0017
rs11915371	A	-0.0148	0.0024	-0.0082	0.0101	0.0102	0.0021
rs11945861	A	-0.0143	0.0023	-0.0008	0.0092	0.0068	0.0020
rs11976018	A	-0.0228	0.0027	-0.0138	0.0103	0.0041	0.0023
rs12049202	T	0.0245	0.0024	0.0200	0.0095	-0.0067	0.0021
rs1218822	A	0.0176	0.0021	-0.0172	0.0083	-0.0024	0.0018
rs12334877	A	-0.0152	0.0025	-0.0076	0.0095	0.0047	0.0021
rs12369179	T	-0.0372	0.0034	0.0270	0.0168	-0.0143	0.0030
rs12427047	T	-0.0184	0.0023	-0.0199	0.0091	-0.0041	0.0020
rs12439798	T	0.0139	0.0020	0.0027	0.0080	-0.0038	0.0017
rs12448257	A	0.0157	0.0024	0.0042	0.0097	0.0074	0.0021
rs12512994	T	-0.0127	0.0021	-0.0051	0.0092	0.0140	0.0018
rs12564848	T	-0.0229	0.0031	0.0365	0.0120	-0.0015	0.0027
rs12595749	A	0.0149	0.0020	-0.0119	0.0079	-0.0022	0.0017
rs12608738	A	0.0130	0.0022	0.0079	0.0098	-0.0020	0.0019
rs1266874	A	-0.0147	0.0020	0.0088	0.0084	0.0023	0.0018
rs12681792	A	0.0149	0.0025	0.0260	0.0096	-0.0034	0.0021
rs12705977	T	0.0134	0.0020	0.0011	0.0085	-0.0079	0.0017
rs12762034	T	-0.0262	0.0037	-0.0372	0.0140	0.0059	0.0032
rs12878246	T	0.0137	0.0023	0.0023	0.0101	-0.0017	0.0020
rs12906208	A	-0.0149	0.0024	0.0011	0.0104	0.0039	0.0021
rs12936083	A	-0.0137	0.0021	0.0000	0.0083	0.0014	0.0018
rs12936319	A	0.0167	0.0020	-0.0143	0.0084	-0.0072	0.0017
rs12949279	T	0.0180	0.0020	0.0070	0.0080	-0.0031	0.0017
rs1296328	A	0.0190	0.0020	0.0086	0.0090	-0.0024	0.0017
rs12981256	A	0.0140	0.0020	0.0036	0.0086	-0.0021	0.0017
rs13034320	C	0.0136	0.0023	0.0102	0.0088	-0.0012	0.0020
rs13047416	C	0.0143	0.0020	-0.0065	0.0083	0.0015	0.0018
rs13085002	T	-0.0168	0.0027	0.0041	0.0114	0.0009	0.0023
rs13107325	T	0.0469	0.0037	-0.0005	0.0193	-0.0188	0.0034
rs13110266	A	-0.0119	0.0020	0.0031	0.0080	0.0030	0.0017
rs13132853	A	0.0138	0.0021	-0.0083	0.0096	-0.0096	0.0018
rs13147390	T	-0.0122	0.0020	-0.0059	0.0082	0.0034	0.0018
rs13174863	A	-0.0210	0.0028	0.0084	0.0125	-0.0014	0.0024
rs13199223	T	0.0209	0.0028	-0.0213	0.0124	-0.0076	0.0025

rs1320251	T	-0.0168	0.0020	-0.0104	0.0084	0.0038	0.0017
rs1320903	A	0.0229	0.0021	-0.0012	0.0087	-0.0004	0.0018
rs13209689	A	-0.0132	0.0021	0.0018	0.0086	0.0056	0.0018
rs13209918	A	-0.0134	0.0020	-0.0093	0.0084	0.0018	0.0018
rs13218383	C	0.0135	0.0021	0.0175	0.0082	-0.0014	0.0018
rs13263601	A	-0.0150	0.0020	-0.0074	0.0086	0.0056	0.0018
rs1327259	A	0.0155	0.0020	-0.0101	0.0083	-0.0031	0.0017
rs1328448	A	0.0127	0.0021	-0.0007	0.0085	-0.0019	0.0019
rs13288178	A	0.0141	0.0020	-0.0014	0.0083	0.0012	0.0018
rs13299074	T	0.0147	0.0025	-0.0260	0.0106	-0.0007	0.0022
rs13427822	A	0.0178	0.0022	-0.0069	0.0096	-0.0053	0.0019
rs1348716	A	-0.0117	0.0020	0.0022	0.0083	-0.0127	0.0018
rs13665	C	0.0160	0.0021	-0.0064	0.0086	-0.0041	0.0018
rs1406120	A	-0.0182	0.0031	-0.0206	0.0130	0.0123	0.0027
rs1412235	C	0.0246	0.0021	0.0051	0.0088	0.0013	0.0018
rs1421085	T	-0.0767	0.0020	-0.0071	0.0085	-0.0027	0.0017
rs1421334	A	0.0131	0.0020	-0.0076	0.0082	-0.0112	0.0017
rs1431659	A	0.0222	0.0022	0.0142	0.0088	-0.0001	0.0019
rs1436344	C	0.0145	0.0020	0.0077	0.0081	-0.0019	0.0017
rs1454687	C	0.0226	0.0019	0.0066	0.0081	-0.0072	0.0017
rs1460940	A	0.0312	0.0024	-0.0167	0.0108	0.0024	0.0022
rs1470153	A	-0.0138	0.0023	-0.0008	0.0088	0.0067	0.0020
rs1477890	A	-0.0162	0.0020	-0.0065	0.0079	0.0018	0.0017
rs1503526	T	-0.0144	0.0020	-0.0187	0.0083	0.0105	0.0017
rs1522569	T	0.0167	0.0025	0.0074	0.0112	-0.0055	0.0022
rs1574389	T	0.0161	0.0024	-0.0093	0.0091	-0.0031	0.0021
rs1579557	T	0.0225	0.0021	0.0008	0.0087	-0.0037	0.0019
rs1582931	A	-0.0142	0.0020	-0.0283	0.0079	-0.0074	0.0017
rs1583123	A	0.0124	0.0020	-0.0054	0.0082	-0.0038	0.0018
rs1681740	A	0.0122	0.0020	-0.0020	0.0085	-0.0027	0.0017
rs16868443	C	0.0129	0.0020	-0.0012	0.0086	0.0035	0.0018
rs16882450	A	0.0132	0.0021	0.0154	0.0088	-0.0057	0.0019
rs16916303	A	0.0195	0.0031	0.0028	0.0111	-0.0026	0.0027
rs16973520	T	-0.0138	0.0023	-0.0173	0.0087	0.0045	0.0020
rs16996657	T	-0.0193	0.0029	-0.0177	0.0126	0.0012	0.0025
rs17014375	T	-0.0187	0.0029	-0.0209	0.0114	0.0004	0.0025
rs1701827	A	0.0145	0.0022	0.0112	0.0091	-0.0028	0.0019
rs17024258	T	0.0648	0.0061	-0.0185	0.0239	0.0039	0.0050
rs17056301	T	-0.0132	0.0023	0.0075	0.0090	-0.0022	0.0019
rs17066856	T	0.0392	0.0034	-0.0159	0.0121	-0.0042	0.0030
rs1711171	T	-0.0185	0.0023	0.0003	0.0093	0.0029	0.0020
rs17132130	C	-0.0173	0.0024	-0.0094	0.0094	0.0108	0.0020
rs17207196	T	-0.0228	0.0020	-0.0356	0.0096	0.0091	0.0018
rs17379561	A	-0.0181	0.0028	-0.0013	0.0121	0.0190	0.0024
rs17399237	T	0.0120	0.0020	-0.0114	0.0087	-0.0051	0.0017
rs17446299	C	-0.0157	0.0026	0.0028	0.0116	-0.0014	0.0023

rs17513613	T	-0.0201	0.0021	0.0067	0.0090	-0.0010	0.0018
rs17636031	T	-0.0164	0.0022	-0.0031	0.0107	0.0041	0.0019
rs17639546	A	-0.0240	0.0027	-0.0011	0.0110	0.0035	0.0024
rs17724992	A	0.0177	0.0022	0.0233	0.0087	-0.0060	0.0019
rs17776719	A	-0.0171	0.0028	0.0131	0.0108	-0.0016	0.0024
rs17806379	T	-0.0267	0.0025	-0.0203	0.0106	0.0079	0.0022
rs17814208	A	-0.0134	0.0023	-0.0049	0.0095	0.0018	0.0020
rs17827419	A	-0.0213	0.0030	-0.0329	0.0139	0.0033	0.0026
rs1786141	T	0.0136	0.0020	0.0026	0.0087	-0.0062	0.0017
rs1814170	A	0.0198	0.0033	-0.0108	0.0110	0.0040	0.0028
rs1861410	T	-0.0219	0.0020	0.0020	0.0079	0.0031	0.0017
rs1884897	A	-0.0216	0.0020	-0.0024	0.0086	-0.0010	0.0018
rs1928538	T	-0.0135	0.0020	-0.0138	0.0078	0.0024	0.0017
rs1951455	T	-0.0150	0.0022	0.0162	0.0093	0.0018	0.0019
rs1955540	T	-0.0154	0.0025	-0.0013	0.0098	0.0038	0.0022
rs197374	T	0.0140	0.0020	-0.0064	0.0081	-0.0087	0.0018
rs1976053	C	-0.0186	0.0025	-0.0143	0.0093	0.0034	0.0022
rs2012286	A	0.0139	0.0020	0.0083	0.0081	-0.0044	0.0017
rs2051559	T	-0.0192	0.0029	-0.0268	0.0112	0.0152	0.0025
rs2065418	T	0.0184	0.0020	0.0183	0.0083	0.0025	0.0018
rs2072858	T	0.0164	0.0021	-0.0143	0.0083	0.0045	0.0018
rs214247	T	0.0136	0.0020	0.0026	0.0083	-0.0035	0.0018
rs2143253	A	-0.0201	0.0030	-0.0191	0.0108	0.0125	0.0026
rs215634	A	0.0158	0.0020	0.0100	0.0082	-0.0095	0.0018
rs2161796	T	0.0141	0.0020	0.0069	0.0080	0.0005	0.0018
rs217671	A	-0.0169	0.0022	0.0116	0.0088	0.0070	0.0019
rs2181375	A	-0.0201	0.0020	0.0039	0.0081	0.0021	0.0017
rs2183947	A	-0.0162	0.0023	-0.0064	0.0096	0.0106	0.0020
rs2253310	C	-0.0169	0.0020	0.0085	0.0081	-0.0093	0.0018
rs2271189	A	-0.0161	0.0020	-0.0095	0.0081	0.0121	0.0018
rs2281819	A	-0.0162	0.0023	-0.0122	0.0095	0.0065	0.0020
rs2284746	C	0.0112	0.0019	-0.0020	0.0083	-0.0026	0.0018
rs2306540	T	0.0137	0.0021	0.0096	0.0098	-0.0031	0.0018
rs2307111	T	0.0276	0.0020	0.0103	0.0083	-0.0052	0.0017
rs2414128	T	0.0131	0.0020	0.0025	0.0081	0.0026	0.0017
rs241472	T	-0.0190	0.0021	0.0049	0.0084	0.0021	0.0018
rs2425847	A	-0.0126	0.0020	-0.0147	0.0083	0.0031	0.0017
rs2433733	A	-0.0169	0.0021	-0.0070	0.0085	0.0057	0.0018
rs244415	A	-0.0226	0.0020	-0.0091	0.0083	0.0011	0.0017
rs2444253	C	0.0138	0.0020	-0.0029	0.0079	-0.0038	0.0017
rs2450444	A	-0.0127	0.0021	0.0220	0.0087	0.0008	0.0018
rs2472297	T	0.0147	0.0022	-0.0003	0.0111	-0.0002	0.0020
rs2479958	A	0.0158	0.0020	0.0084	0.0085	-0.0021	0.0017
rs248139	A	0.0151	0.0025	-0.0118	0.0097	0.0021	0.0021
rs2591175	A	-0.0144	0.0025	0.0001	0.0095	-0.0013	0.0022
rs2605570	A	0.0151	0.0026	0.0133	0.0104	-0.0019	0.0023

rs2616143	A	-0.0133	0.0021	-0.0117	0.0093	0.0005	0.0018
rs2644112	T	-0.0241	0.0021	-0.0187	0.0088	0.0109	0.0018
rs2693826	A	-0.0136	0.0020	-0.0150	0.0079	0.0022	0.0017
rs2694047	A	-0.0211	0.0023	-0.0057	0.0089	0.0013	0.0020
rs2733287	A	-0.0155	0.0020	-0.0068	0.0078	-0.0067	0.0017
rs273512	T	0.0171	0.0020	0.0131	0.0082	-0.0054	0.0017
rs2781668	T	0.0159	0.0026	0.0017	0.0102	0.0048	0.0023
rs2791643	T	-0.0143	0.0023	0.0014	0.0097	0.0040	0.0020
rs2803316	A	-0.0148	0.0020	0.0079	0.0088	-0.0062	0.0017
rs2814942	A	0.0269	0.0021	0.0111	0.0082	-0.0038	0.0019
rs28350	A	0.0186	0.0026	-0.0133	0.0108	0.0022	0.0022
rs2836968	A	0.0122	0.0020	-0.0040	0.0089	-0.0052	0.0018
rs2861683	A	0.0171	0.0020	0.0085	0.0080	0.0003	0.0017
rs2865384	A	-0.0121	0.0020	-0.0112	0.0082	0.0047	0.0018
rs2875762	C	0.0151	0.0023	-0.0001	0.0094	-0.0012	0.0020
rs2906132	T	0.0119	0.0020	0.0094	0.0088	-0.0016	0.0017
rs2920502	C	-0.0147	0.0021	0.0091	0.0086	0.0030	0.0018
rs294704	T	-0.0146	0.0022	0.0091	0.0096	-0.0007	0.0019
rs2952862	T	0.0143	0.0021	0.0189	0.0090	-0.0043	0.0018
rs326896	T	-0.0123	0.0020	0.0001	0.0082	0.0038	0.0017
rs329651	T	0.0169	0.0025	-0.0174	0.0121	-0.0075	0.0022
rs355748	T	0.0162	0.0020	0.0009	0.0079	-0.0037	0.0018
rs3730071	A	-0.0340	0.0057	0.0424	0.0294	-0.0043	0.0052
rs3743950	A	-0.0119	0.0020	-0.0001	0.0090	0.0013	0.0018
rs3754963	A	0.0139	0.0022	0.0208	0.0090	-0.0106	0.0019
rs3762444	T	-0.0144	0.0020	-0.0035	0.0081	0.0017	0.0017
rs3770451	T	0.0144	0.0022	-0.0019	0.0088	-0.0104	0.0019
rs3784710	T	0.0287	0.0023	0.0241	0.0089	-0.0005	0.0021
rs3803286	A	0.0193	0.0021	-0.0074	0.0081	-0.0070	0.0018
rs3806114	A	-0.0125	0.0021	-0.0045	0.0088	0.0000	0.0018
rs3806572	A	-0.0154	0.0021	-0.0079	0.0094	-0.0004	0.0019
rs3814883	T	0.0249	0.0020	-0.0208	0.0083	-0.0005	0.0017
rs3821709	T	-0.0338	0.0039	-0.0134	0.0134	0.0128	0.0035
rs38314	A	-0.0116	0.0020	-0.0231	0.0085	-0.0057	0.0017
rs394608	T	-0.0177	0.0020	0.0028	0.0080	0.0044	0.0017
rs40067	A	-0.0272	0.0026	-0.0266	0.0099	0.0011	0.0023
rs4072917	A	0.0118	0.0020	-0.0051	0.0079	-0.0024	0.0017
rs4148155	A	0.0203	0.0031	0.0039	0.0119	-0.0004	0.0027
rs4148866	T	0.0124	0.0020	0.0129	0.0084	-0.0072	0.0017
rs4237195	A	0.0177	0.0021	0.0094	0.0088	-0.0046	0.0018
rs4237643	T	0.0237	0.0021	0.0259	0.0086	-0.0024	0.0019
rs4240673	T	0.0201	0.0020	0.0153	0.0088	-0.0026	0.0017
rs4284600	T	-0.0115	0.0020	-0.0125	0.0087	0.0019	0.0017
rs429343	A	0.0172	0.0020	0.0194	0.0083	-0.0006	0.0017
rs4307239	A	-0.0120	0.0020	0.0087	0.0082	0.0000	0.0017
rs4343164	T	0.0165	0.0022	0.0086	0.0097	-0.0066	0.0019

rs4477562	T	0.0317	0.0030	0.0258	0.0112	-0.0001	0.0026
rs4482463	A	-0.0346	0.0037	-0.0381	0.0120	-0.0043	0.0032
rs4490752	A	0.0172	0.0020	0.0101	0.0082	0.0000	0.0018
rs4516268	A	-0.0241	0.0025	-0.0193	0.0102	0.0096	0.0022
rs4639527	A	-0.0174	0.0021	0.0214	0.0087	-0.0012	0.0018
rs4652839	C	-0.0132	0.0021	-0.0116	0.0086	0.0027	0.0018
rs4654831	T	0.0188	0.0026	0.0089	0.0098	-0.0021	0.0023
rs4658403	T	-0.0186	0.0026	-0.0075	0.0098	0.0104	0.0023
rs4660443	T	0.0182	0.0024	-0.0003	0.0098	-0.0010	0.0021
rs4670169	A	-0.0120	0.0020	-0.0061	0.0089	-0.0056	0.0018
rs4722398	T	0.0178	0.0028	0.0102	0.0122	0.0040	0.0026
rs4762369	A	0.0151	0.0025	-0.0053	0.0105	0.0018	0.0022
rs4764949	A	0.0196	0.0021	-0.0013	0.0083	0.0010	0.0018
rs4851716	A	0.0162	0.0020	-0.0024	0.0082	0.0018	0.0018
rs4911382	T	0.0147	0.0020	0.0061	0.0080	-0.0034	0.0017
rs4936175	T	-0.0124	0.0020	0.0000	0.0090	0.0036	0.0017
rs4942924	T	0.0158	0.0020	0.0036	0.0082	-0.0022	0.0017
rs4952843	A	0.0151	0.0020	-0.0022	0.0087	0.0006	0.0017
rs4969387	C	-0.0146	0.0022	0.0065	0.0095	-0.0027	0.0020
rs500764	T	0.0164	0.0020	-0.0035	0.0080	0.0001	0.0017
rs538656	T	0.0544	0.0023	0.0077	0.0089	0.0056	0.0020
rs538740	T	-0.0145	0.0024	-0.0082	0.0095	0.0074	0.0021
rs5396	T	-0.0175	0.0022	-0.0135	0.0096	0.0088	0.0019
rs543874	A	-0.0466	0.0024	0.0008	0.0098	0.0047	0.0021
rs559231	T	0.0142	0.0020	0.0029	0.0082	0.0015	0.0017
rs577525	T	-0.0187	0.0020	0.0122	0.0081	0.0066	0.0017
rs592483	T	-0.0152	0.0020	0.0148	0.0084	0.0029	0.0017
rs6011457	A	-0.0116	0.0020	0.0051	0.0081	-0.0073	0.0017
rs6023649	A	0.0153	0.0023	-0.0076	0.0095	-0.0074	0.0019
rs6050446	A	-0.0375	0.0056	-0.0066	0.0292	0.0121	0.0048
rs6098868	A	-0.0117	0.0020	0.0145	0.0079	-0.0010	0.0017
rs6265	T	-0.0405	0.0025	-0.0136	0.0100	0.0097	0.0022
rs6443750	T	-0.0152	0.0025	-0.0048	0.0127	0.0067	0.0022
rs6448587	A	0.0196	0.0026	0.0057	0.0097	-0.0004	0.0022
rs6479905	A	-0.0126	0.0020	-0.0062	0.0078	0.0143	0.0017
rs6502482	C	-0.0133	0.0020	0.0112	0.0081	-0.0041	0.0017
rs6542924	A	-0.0174	0.0021	-0.0119	0.0092	0.0174	0.0018
rs6551304	A	-0.0186	0.0026	0.0148	0.0106	0.0047	0.0023
rs6577584	T	-0.0131	0.0021	0.0000	0.0085	0.0023	0.0018
rs6587552	A	0.0175	0.0023	-0.0040	0.0093	-0.0076	0.0020
rs6604866	C	-0.0161	0.0020	0.0064	0.0090	0.0003	0.0017
rs6674079	A	-0.0127	0.0020	0.0062	0.0082	0.0055	0.0018
rs6682	A	-0.0121	0.0020	-0.0155	0.0083	0.0038	0.0017
rs671722	A	-0.0126	0.0020	-0.0073	0.0090	0.0093	0.0017
rs6725931	T	0.0187	0.0027	0.0096	0.0112	-0.0115	0.0024
rs6728037	A	0.0198	0.0020	0.0125	0.0081	-0.0046	0.0017

rs6731688	A	-0.0564	0.0026	-0.0037	0.0109	0.0047	0.0023
rs6752378	A	0.0332	0.0019	0.0002	0.0081	0.0012	0.0017
rs6764533	A	0.0141	0.0020	0.0176	0.0087	-0.0073	0.0018
rs6831020	A	-0.0161	0.0021	0.0015	0.0099	0.0027	0.0019
rs6870983	T	-0.0219	0.0024	-0.0170	0.0101	0.0141	0.0021
rs6905544	A	-0.0164	0.0020	-0.0018	0.0080	0.0233	0.0017
rs698147	A	0.0130	0.0020	0.0005	0.0081	-0.0045	0.0017
rs7038943	T	0.0143	0.0021	0.0012	0.0080	-0.0075	0.0018
rs705158	A	0.0162	0.0023	-0.0001	0.0091	0.0007	0.0020
rs705217	T	0.0123	0.0020	0.0059	0.0083	-0.0040	0.0018
rs7084454	A	0.0210	0.0021	-0.0183	0.0090	-0.0068	0.0018
rs7096307	T	-0.0117	0.0020	-0.0063	0.0085	0.0024	0.0017
rs7102454	T	-0.0176	0.0020	-0.0097	0.0084	0.0071	0.0018
rs7124681	A	0.0269	0.0020	0.0177	0.0080	0.0002	0.0017
rs7132908	A	0.0296	0.0020	0.0057	0.0088	-0.0005	0.0018
rs7158822	T	-0.0162	0.0021	-0.0052	0.0079	0.0034	0.0018
rs7181498	T	0.0160	0.0021	0.0006	0.0082	-0.0052	0.0018
rs7196720	T	0.0131	0.0020	0.0087	0.0081	-0.0045	0.0017
rs7258722	A	-0.0200	0.0020	-0.0155	0.0084	0.0015	0.0017
rs725959	T	-0.0133	0.0020	-0.0088	0.0081	0.0054	0.0017
rs7313220	A	0.0129	0.0020	-0.0079	0.0080	0.0020	0.0017
rs7334078	T	0.0125	0.0022	0.0090	0.0091	-0.0103	0.0019
rs733758	T	-0.0313	0.0045	0.0108	0.0192	-0.0068	0.0039
rs738140	A	0.0147	0.0021	0.0103	0.0096	-0.0033	0.0018
rs741959	A	0.0173	0.0020	0.0070	0.0087	-0.0001	0.0017
rs7498665	A	-0.0256	0.0020	-0.0014	0.0085	0.0166	0.0017
rs7518221	T	0.0126	0.0020	0.0056	0.0084	0.0003	0.0018
rs7519259	A	0.0137	0.0020	0.0246	0.0083	-0.0064	0.0017
rs754635	C	-0.0198	0.0031	0.0059	0.0110	-0.0032	0.0027
rs7557796	T	0.0171	0.0020	-0.0021	0.0083	-0.0030	0.0018
rs756717	A	-0.0158	0.0020	0.0037	0.0083	-0.0006	0.0017
rs7568228	C	-0.0116	0.0019	-0.0030	0.0086	0.0050	0.0017
rs7619139	A	0.0146	0.0020	0.0052	0.0079	0.0007	0.0017
rs7626079	T	0.0121	0.0021	0.0013	0.0082	0.0052	0.0018
rs7637852	A	0.0143	0.0021	0.0050	0.0092	-0.0013	0.0019
rs7640424	T	-0.0128	0.0021	-0.0127	0.0097	0.0107	0.0018
rs7646113	T	-0.0125	0.0020	-0.0033	0.0080	0.0010	0.0018
rs765874	A	-0.0125	0.0020	0.0130	0.0082	0.0022	0.0017
rs7683836	A	-0.0135	0.0020	-0.0020	0.0082	0.0003	0.0017
rs769449	A	-0.0235	0.0030	-0.0060	0.0137	0.0067	0.0026
rs7707981	A	0.0142	0.0021	0.0014	0.0091	0.0009	0.0018
rs7715256	T	-0.0165	0.0020	-0.0063	0.0083	-0.0007	0.0017
rs7733438	T	-0.0365	0.0029	-0.0154	0.0107	-0.0173	0.0025
rs774246	A	-0.0169	0.0028	0.0113	0.0123	0.0012	0.0024
rs7747583	A	-0.0136	0.0022	-0.0127	0.0084	0.0074	0.0019
rs775403	T	0.0117	0.0020	0.0094	0.0088	-0.0044	0.0017

rs7792703	A	-0.0166	0.0024	0.0059	0.0097	0.0010	0.0020
rs7869771	A	0.0156	0.0022	-0.0033	0.0087	-0.0047	0.0019
rs7871866	C	0.0177	0.0027	0.0023	0.0108	-0.0062	0.0024
rs7893571	T	0.0146	0.0021	0.0007	0.0089	0.0024	0.0018
rs7903146	T	-0.0157	0.0022	0.0324	0.0097	-0.0021	0.0019
rs7925214	T	0.0157	0.0020	0.0070	0.0079	-0.0046	0.0017
rs799449	T	0.0141	0.0020	0.0082	0.0086	-0.0070	0.0017
rs8008285	T	-0.0175	0.0028	0.0101	0.0119	-0.0046	0.0024
rs8015400	A	0.0226	0.0021	-0.0134	0.0084	-0.0022	0.0018
rs8089514	A	0.0133	0.0021	0.0071	0.0090	-0.0038	0.0018
rs8181823	A	-0.0139	0.0023	0.0058	0.0094	0.0061	0.0020
rs879620	T	0.0227	0.0020	0.0069	0.0084	-0.0008	0.0018
rs890156	A	0.0125	0.0020	-0.0022	0.0078	0.0023	0.0017
rs891387	T	0.0205	0.0020	0.0197	0.0083	-0.0137	0.0017
rs895330	C	0.0205	0.0025	0.0319	0.0113	-0.0034	0.0022
rs900144	T	0.0174	0.0020	-0.0007	0.0083	0.0015	0.0017
rs902503	A	0.0130	0.0020	-0.0009	0.0080	-0.0013	0.0017
rs903959	A	0.0116	0.0020	0.0087	0.0085	-0.0157	0.0017
rs912690	C	-0.0152	0.0020	-0.0134	0.0078	0.0030	0.0017
rs9294260	A	0.0158	0.0020	0.0138	0.0082	-0.0054	0.0017
rs9296389	C	0.0122	0.0020	-0.0146	0.0078	-0.0012	0.0017
rs930295	A	0.0214	0.0026	0.0040	0.0111	0.0000	0.0023
rs9304665	A	0.0225	0.0023	0.0058	0.0098	-0.0003	0.0020
rs942065	A	0.0204	0.0020	0.0047	0.0084	-0.0071	0.0018
rs9446149	T	0.0145	0.0024	-0.0007	0.0100	0.0017	0.0021
rs9478496	T	-0.0173	0.0026	-0.0364	0.0111	0.0013	0.0023
rs9496567	A	0.0163	0.0023	0.0146	0.0104	0.0008	0.0020
rs9515448	A	-0.0141	0.0020	-0.0135	0.0081	0.0066	0.0017
rs9530843	A	0.0134	0.0020	-0.0048	0.0083	-0.0027	0.0017
rs9538162	T	0.0168	0.0020	0.0005	0.0082	-0.0018	0.0017
rs9571687	A	-0.0126	0.0021	-0.0013	0.0082	-0.0016	0.0018
rs960923	A	-0.0210	0.0020	0.0035	0.0080	-0.0029	0.0017
rs9630985	A	-0.0173	0.0021	0.0045	0.0085	0.0058	0.0018
rs9650755	A	-0.0159	0.0022	0.0034	0.0087	0.0069	0.0019
rs9688431	T	0.0242	0.0042	0.0130	0.0136	0.0000	0.0036
rs9816226	A	-0.0292	0.0025	-0.0253	0.0111	-0.0078	0.0022
rs9827823	T	0.0219	0.0027	-0.0070	0.0111	-0.0017	0.0024
rs987237	A	-0.0395	0.0025	-0.0172	0.0102	0.0033	0.0022
rs9876664	T	-0.0181	0.0020	-0.0042	0.0082	0.0139	0.0018
rs9926784	T	0.0262	0.0025	-0.0014	0.0097	0.0032	0.0022
rs9927848	A	-0.0129	0.0022	-0.0031	0.0089	-0.0022	0.0019
rs9951619	T	-0.0160	0.0024	0.0026	0.0094	-0.0052	0.0020
rs9958909	T	-0.0214	0.0029	-0.0027	0.0120	0.0027	0.0024

Supplementary Table 7: Genetic association estimates for the IVW ratio-method MR analysis estimating the effect of education on BMI. ea=effect allele; gx=education; gy=BMI; se=standard error

SNP	ea	gx	gx_se	gy	gy_se
rs10032941	T	-0.0080	0.0015	-0.0026	0.0020
rs10041403	T	0.0151	0.0019	-0.0047	0.0026
rs10042828	A	-0.0145	0.0022	0.0051	0.0030
rs10057590	A	0.0106	0.0014	-0.0108	0.0020
rs10061420	A	0.0082	0.0014	0.0019	0.0020
rs1007731	A	-0.0143	0.0022	-0.0028	0.0031
rs10080647	A	0.0124	0.0020	0.0022	0.0028
rs1009470	T	0.0086	0.0014	0.0033	0.0020
rs10098073	A	-0.0091	0.0014	-0.0064	0.0020
rs10169002	A	-0.0080	0.0014	-0.0033	0.0020
rs10181071	A	0.0102	0.0019	-0.0035	0.0026
rs10189857	A	0.0158	0.0014	-0.0076	0.0020
rs10192369	A	-0.0078	0.0014	0.0025	0.0019
rs10192834	T	0.0083	0.0015	0.0020	0.0020
rs10204051	T	-0.0101	0.0014	0.0059	0.0020
rs10205057	T	0.0090	0.0014	-0.0012	0.0020
rs10208	T	0.0091	0.0015	0.0100	0.0021
rs10251438	A	0.0078	0.0014	-0.0058	0.0020
rs1035578	A	-0.0094	0.0014	0.0024	0.0020
rs10480450	A	0.0158	0.0028	-0.0021	0.0042
rs10496091	A	-0.0131	0.0016	0.0028	0.0022
rs10499535	A	0.0080	0.0014	-0.0047	0.0020
rs1050847	T	0.0092	0.0014	0.0016	0.0020
rs10509251	T	-0.0093	0.0017	0.0023	0.0024
rs10515007	T	0.0143	0.0019	0.0035	0.0027
rs1051860	A	-0.0079	0.0014	0.0047	0.0020
rs1054442	A	-0.0136	0.0015	-0.0098	0.0020
rs1066769	A	-0.0230	0.0041	-0.0006	0.0053
rs10750539	A	-0.0095	0.0015	-0.0047	0.0021
rs10761202	T	0.0088	0.0014	-0.0010	0.0020
rs10782651	T	-0.0082	0.0014	0.0125	0.0020
rs10783243	A	-0.0092	0.0014	-0.0011	0.0020
rs10789285	T	0.0091	0.0016	-0.0020	0.0023
rs10795831	T	-0.0099	0.0017	0.0039	0.0024
rs10798888	T	-0.0140	0.0019	0.0126	0.0026
rs10799615	A	-0.0089	0.0016	0.0069	0.0022
rs10805383	A	-0.0103	0.0014	0.0142	0.0020
rs10810099	A	-0.0146	0.0016	0.0062	0.0022
rs10831656	T	0.0090	0.0015	-0.0017	0.0021
rs10845051	A	0.0083	0.0014	-0.0019	0.0020
rs10875121	C	0.0174	0.0019	-0.0164	0.0026

rs10877283	T	0.0085	0.0014	-0.0010	0.0020
rs10879676	T	-0.0083	0.0014	0.0046	0.0020
rs10928190	T	-0.0087	0.0014	0.0002	0.0020
rs10937240	T	-0.0123	0.0018	-0.0246	0.0025
rs10940921	T	0.0111	0.0014	0.0002	0.0020
rs10947439	T	0.0090	0.0015	-0.0021	0.0021
rs10974256	A	-0.0093	0.0015	-0.0028	0.0021
rs1097784	T	-0.0083	0.0014	-0.0031	0.0020
rs10979613	T	-0.0117	0.0015	0.0000	0.0020
rs10985402	T	0.0089	0.0016	-0.0010	0.0023
rs11030084	T	0.0106	0.0018	-0.0385	0.0025
rs11030102	C	0.0101	0.0016	-0.0286	0.0022
rs1106090	A	0.0094	0.0014	-0.0128	0.0020
rs11076962	T	0.0086	0.0016	-0.0044	0.0022
rs11081529	T	0.0126	0.0015	-0.0009	0.0022
rs11082011	T	0.0194	0.0015	0.0039	0.0021
rs11100237	A	-0.0101	0.0014	0.0000	0.0020
rs11115056	A	0.0187	0.0023	-0.0123	0.0033
rs11130380	T	0.0090	0.0014	0.0042	0.0020
rs11138947	T	0.0105	0.0016	0.0034	0.0022
rs11155813	T	-0.0129	0.0023	0.0026	0.0033
rs11158800	A	-0.0091	0.0014	0.0085	0.0020
rs1117152	T	0.0106	0.0015	-0.0069	0.0022
rs11174399	A	0.0111	0.0018	-0.0021	0.0024
rs11190955	T	-0.0083	0.0015	-0.0058	0.0020
rs1120924	T	0.0108	0.0015	-0.0057	0.0021
rs11210400	A	0.0120	0.0014	0.0032	0.0020
rs11213482	A	-0.0106	0.0019	0.0006	0.0026
rs1123285	C	-0.0094	0.0015	0.0016	0.0021
rs11265191	T	0.0083	0.0015	0.0001	0.0021
rs1146079	C	-0.0087	0.0016	0.0050	0.0022
rs11588857	A	0.0199	0.0017	-0.0030	0.0024
rs11599236	T	-0.0106	0.0014	0.0080	0.0020
rs11613431	A	-0.0094	0.0016	0.0014	0.0022
rs11623285	T	-0.0135	0.0021	-0.0009	0.0029
rs11644446	A	0.0117	0.0019	-0.0037	0.0028
rs11663602	A	-0.0130	0.0016	0.0051	0.0022
rs11663678	T	-0.0121	0.0022	0.0129	0.0030
rs11675476	T	-0.0127	0.0014	0.0068	0.0019
rs1167827	A	0.0104	0.0014	-0.0204	0.0020
rs11693885	A	-0.0091	0.0014	0.0045	0.0020
rs1171040	A	-0.0113	0.0017	0.0064	0.0024
rs11724690	T	0.0093	0.0015	0.0001	0.0022
rs11732160	A	0.0105	0.0016	-0.0079	0.0023
rs11780023	T	0.0085	0.0014	0.0005	0.0020
rs11789013	T	0.0106	0.0016	0.0020	0.0023

rs11845781	T	-0.0079	0.0014	0.0017	0.0020
rs11871429	A	0.0128	0.0017	-0.0040	0.0023
rs11876620	T	0.0143	0.0024	0.0150	0.0033
rs11894424	A	0.0209	0.0033	-0.0135	0.0047
rs11917701	A	-0.0087	0.0014	-0.0025	0.0020
rs11919835	C	0.0089	0.0015	0.0002	0.0020
rs1196760	C	0.0139	0.0025	-0.0105	0.0034
rs12028010	T	0.0164	0.0017	-0.0014	0.0023
rs12113634	T	0.0088	0.0015	0.0004	0.0021
rs12123293	T	0.0086	0.0015	0.0003	0.0020
rs12151248	T	-0.0149	0.0023	-0.0055	0.0031
rs12170452	A	0.0105	0.0014	-0.0033	0.0020
rs12234369	A	0.0087	0.0015	-0.0048	0.0021
rs12238011	T	-0.0141	0.0026	0.0055	0.0036
rs12342546	A	-0.0128	0.0017	0.0009	0.0023
rs12375949	T	-0.0138	0.0014	0.0087	0.0020
rs12405889	T	-0.0098	0.0014	-0.0056	0.0019
rs12431682	T	-0.0093	0.0015	-0.0132	0.0020
rs12438177	A	0.0093	0.0015	-0.0006	0.0020
rs12453682	T	0.0102	0.0015	-0.0020	0.0022
rs1245829	A	-0.0099	0.0014	-0.0040	0.0020
rs12467175	T	-0.0081	0.0014	0.0099	0.0019
rs12477385	T	0.0109	0.0017	-0.0144	0.0023
rs12478156	T	0.0108	0.0015	-0.0033	0.0020
rs12503522	T	-0.0113	0.0016	0.0037	0.0022
rs12516990	C	-0.0099	0.0017	0.0042	0.0025
rs12519073	T	-0.0107	0.0017	0.0042	0.0024
rs12571549	A	0.0151	0.0020	-0.0072	0.0028
rs12601380	A	-0.0082	0.0014	0.0158	0.0020
rs12602286	T	0.0160	0.0021	-0.0043	0.0030
rs12613500	C	0.0098	0.0014	-0.0082	0.0020
rs12646523	T	-0.0132	0.0016	0.0084	0.0022
rs12670376	A	0.0092	0.0014	-0.0063	0.0020
rs12682775	T	-0.0119	0.0017	0.0010	0.0024
rs12789313	T	0.0092	0.0014	-0.0035	0.0020
rs12810587	T	0.0094	0.0015	0.0127	0.0021
rs12891042	T	0.0092	0.0014	0.0039	0.0020
rs12891191	C	0.0080	0.0014	-0.0022	0.0020
rs12952191	T	-0.0081	0.0014	0.0073	0.0020
rs12957463	A	0.0146	0.0018	0.0027	0.0024
rs12962845	C	-0.0123	0.0021	0.0055	0.0029
rs13009915	T	0.0212	0.0037	-0.0072	0.0052
rs13010288	T	0.0198	0.0021	-0.0080	0.0029
rs13010566	A	-0.0091	0.0014	0.0019	0.0019
rs13015496	C	0.0119	0.0018	0.0015	0.0025
rs13016316	A	-0.0138	0.0024	-0.0078	0.0034

rs13029602	T	-0.0092	0.0014	0.0060	0.0020
rs13034349	T	-0.0082	0.0014	0.0048	0.0020
rs13050131	A	-0.0081	0.0015	0.0014	0.0021
rs13107325	T	-0.0239	0.0027	0.0469	0.0037
rs13117856	A	0.0084	0.0015	0.0022	0.0022
rs13133213	A	0.0096	0.0014	0.0005	0.0019
rs13163845	T	-0.0149	0.0020	-0.0015	0.0028
rs13169187	A	-0.0079	0.0014	0.0013	0.0020
rs13177031	A	-0.0085	0.0015	-0.0077	0.0021
rs13190235	T	-0.0138	0.0024	-0.0009	0.0034
rs13212041	T	0.0101	0.0018	-0.0071	0.0024
rs13261773	C	0.0122	0.0019	0.0099	0.0027
rs13284516	C	-0.0082	0.0014	-0.0019	0.0020
rs1329044	T	0.0134	0.0019	-0.0056	0.0026
rs1329125	T	-0.0097	0.0015	0.0036	0.0021
rs13296345	T	0.0090	0.0015	-0.0088	0.0020
rs1334297	A	0.0257	0.0016	-0.0113	0.0022
rs13388333	A	0.0085	0.0015	0.0005	0.0020
rs13397529	C	-0.0097	0.0017	0.0098	0.0023
rs13398860	A	0.0086	0.0015	-0.0119	0.0020
rs13422673	T	-0.0110	0.0014	-0.0006	0.0020
rs1392816	T	0.0082	0.0014	-0.0124	0.0020
rs1404549	A	-0.0089	0.0015	0.0026	0.0021
rs1408430	C	0.0096	0.0016	0.0052	0.0022
rs1426619	T	0.0096	0.0014	0.0023	0.0020
rs1427298	T	0.0086	0.0014	-0.0058	0.0020
rs1427829	A	-0.0083	0.0014	0.0137	0.0020
rs1434630	T	-0.0137	0.0020	0.0106	0.0027
rs1464297	T	-0.0109	0.0015	0.0029	0.0021
rs1467737	T	-0.0084	0.0014	0.0017	0.0020
rs1485300	C	0.0098	0.0015	0.0041	0.0021
rs1490612	T	0.0193	0.0026	0.0054	0.0037
rs1527878	A	-0.0105	0.0016	0.0058	0.0023
rs1529597	A	0.0223	0.0038	-0.0122	0.0058
rs1538389	T	-0.0109	0.0018	0.0097	0.0025
rs1542354	A	-0.0084	0.0014	0.0075	0.0020
rs1544	A	-0.0096	0.0016	0.0063	0.0022
rs1564347	T	0.0088	0.0015	-0.0073	0.0021
rs1569092	A	0.0160	0.0020	0.0009	0.0027
rs1603460	T	0.0090	0.0014	0.0009	0.0020
rs162445	A	0.0152	0.0026	0.0032	0.0037
rs1637770	T	0.0191	0.0028	-0.0133	0.0041
rs164938	T	-0.0088	0.0014	0.0078	0.0020
rs1671269	T	-0.0100	0.0016	-0.0042	0.0023
rs16851779	T	-0.0153	0.0027	0.0000	0.0035
rs16901689	T	0.0111	0.0019	-0.0021	0.0026

rs1693584	T	-0.0087	0.0015	0.0028	0.0021
rs16958559	T	0.0096	0.0017	-0.0069	0.0023
rs16966271	T	0.0092	0.0016	0.0011	0.0022
rs16975275	A	-0.0102	0.0017	0.0004	0.0024
rs17048801	A	-0.0106	0.0014	0.0012	0.0021
rs17048855	A	0.0108	0.0015	-0.0010	0.0020
rs17069646	T	0.0085	0.0015	0.0074	0.0021
rs17110109	T	-0.0093	0.0014	0.0124	0.0020
rs17113730	A	-0.0170	0.0027	-0.0122	0.0038
rs17133297	A	-0.0146	0.0023	0.0007	0.0034
rs17144467	A	0.0098	0.0015	-0.0093	0.0021
rs17148998	A	0.0101	0.0017	-0.0060	0.0024
rs17170519	C	0.0132	0.0023	-0.0054	0.0032
rs17190418	T	-0.0213	0.0031	-0.0028	0.0042
rs17205908	T	-0.0092	0.0015	0.0068	0.0021
rs17248751	A	-0.0136	0.0017	0.0024	0.0023
rs1728118	A	0.0089	0.0016	-0.0078	0.0022
rs1729412	T	-0.0107	0.0014	-0.0058	0.0020
rs1734370	A	0.0099	0.0016	0.0012	0.0021
rs1738050	C	-0.0093	0.0014	-0.0003	0.0020
rs17428076	C	0.0137	0.0016	-0.0126	0.0023
rs17440885	A	0.0160	0.0029	-0.0125	0.0040
rs1747817	T	-0.0092	0.0016	0.0022	0.0023
rs17489649	A	0.0127	0.0015	-0.0018	0.0021
rs17502934	T	-0.0167	0.0020	0.0116	0.0028
rs17536059	C	-0.0108	0.0019	0.0075	0.0026
rs17551064	A	0.0141	0.0019	-0.0045	0.0027
rs17563464	A	-0.0147	0.0017	0.0015	0.0024
rs17565975	A	-0.0106	0.0014	-0.0010	0.0020
rs17568389	A	0.0127	0.0014	-0.0025	0.0020
rs17574007	A	-0.0129	0.0021	0.0122	0.0031
rs1758747	A	0.0092	0.0015	0.0005	0.0021
rs17598675	T	-0.0116	0.0014	-0.0011	0.0020
rs17604349	A	0.0127	0.0018	0.0076	0.0026
rs17609255	T	-0.0087	0.0014	0.0025	0.0020
rs176218	T	0.0200	0.0018	-0.0154	0.0025
rs17650634	T	-0.0104	0.0019	-0.0012	0.0026
rs17667540	A	-0.0087	0.0015	0.0126	0.0021
rs17669337	T	-0.0110	0.0014	-0.0048	0.0020
rs17686649	T	-0.0098	0.0017	-0.0049	0.0024
rs17732878	T	-0.0095	0.0017	0.0080	0.0024
rs17742342	A	-0.0119	0.0018	-0.0057	0.0024
rs17747544	A	-0.0090	0.0014	0.0089	0.0020
rs1779549	A	0.0081	0.0014	-0.0026	0.0020
rs1792602	A	-0.0097	0.0014	0.0041	0.0020
rs1861786	A	-0.0081	0.0014	-0.0004	0.0020

rs1865955	T	0.0122	0.0019	-0.0126	0.0026
rs1866823	A	0.0107	0.0014	-0.0015	0.0020
rs187580	T	-0.0104	0.0017	-0.0046	0.0023
rs1880692	A	0.0086	0.0014	0.0037	0.0020
rs1890132	T	-0.0089	0.0015	0.0024	0.0021
rs1898111	A	0.0101	0.0018	-0.0062	0.0026
rs1910005	T	-0.0090	0.0015	0.0021	0.0021
rs1918394	T	0.0119	0.0019	-0.0034	0.0026
rs1933264	T	0.0100	0.0016	-0.0129	0.0023
rs1952183	A	-0.0089	0.0014	0.0031	0.0020
rs1955250	A	-0.0140	0.0025	0.0033	0.0035
rs1991585	T	-0.0101	0.0015	0.0074	0.0021
rs2011603	A	-0.0091	0.0016	0.0028	0.0022
rs2014830	T	0.0110	0.0015	-0.0209	0.0021
rs2023016	C	0.0107	0.0017	0.0000	0.0024
rs2029401	A	-0.0091	0.0014	0.0012	0.0020
rs2034631	T	0.0089	0.0015	-0.0003	0.0021
rs2039204	A	0.0082	0.0014	-0.0012	0.0020
rs2055940	A	0.0082	0.0015	0.0010	0.0021
rs2098526	A	-0.0241	0.0043	0.0150	0.0060
rs2179152	T	-0.0131	0.0015	0.0017	0.0020
rs2183271	T	0.0084	0.0015	-0.0183	0.0020
rs2199409	T	0.0103	0.0017	-0.0051	0.0025
rs2212430	T	-0.0100	0.0016	0.0083	0.0022
rs2216144	T	0.0098	0.0014	0.0017	0.0020
rs2220926	T	-0.0095	0.0014	-0.0038	0.0020
rs2252098	T	-0.0077	0.0014	0.0044	0.0020
rs2256965	A	0.0106	0.0014	-0.0061	0.0020
rs2276209	A	0.0085	0.0015	0.0037	0.0022
rs2283076	A	0.0115	0.0017	-0.0043	0.0023
rs2287838	A	-0.0104	0.0014	-0.0030	0.0020
rs2290601	T	-0.0149	0.0017	0.0098	0.0023
rs2297293	C	0.0099	0.0015	-0.0001	0.0021
rs2299156	T	0.0099	0.0018	0.0022	0.0026
rs2321157	A	-0.0081	0.0014	0.0056	0.0020
rs232496	T	0.0090	0.0015	0.0004	0.0021
rs2358628	A	-0.0091	0.0015	0.0052	0.0021
rs2365376	A	0.0092	0.0015	-0.0041	0.0021
rs2373124	A	-0.0096	0.0016	-0.0037	0.0023
rs2406253	A	0.0135	0.0018	-0.0038	0.0025
rs2416214	A	0.0079	0.0014	-0.0072	0.0020
rs2416845	T	0.0263	0.0048	-0.0141	0.0068
rs2436760	T	0.0232	0.0042	-0.0090	0.0057
rs2447097	T	0.0096	0.0014	-0.0026	0.0020
rs2470966	C	-0.0103	0.0015	0.0045	0.0021
rs2478208	C	-0.0103	0.0014	0.0016	0.0020

rs2496482	T	0.0121	0.0015	-0.0030	0.0020
rs2517086	C	-0.0088	0.0014	0.0018	0.0020
rs2522545	T	-0.0083	0.0014	-0.0023	0.0020
rs252991	A	0.0095	0.0015	-0.0046	0.0020
rs2570497	T	-0.0121	0.0015	0.0115	0.0020
rs2718277	T	0.0142	0.0026	-0.0080	0.0035
rs27220	A	-0.0114	0.0015	-0.0016	0.0020
rs2736752	T	0.0095	0.0017	-0.0058	0.0024
rs2764684	T	0.0143	0.0019	0.0058	0.0026
rs2805064	C	0.0093	0.0016	0.0015	0.0022
rs2838006	T	0.0132	0.0015	-0.0125	0.0021
rs2852349	T	-0.0093	0.0014	0.0046	0.0020
rs2885198	A	0.0088	0.0014	-0.0042	0.0020
rs2926702	T	-0.0124	0.0021	-0.0027	0.0030
rs2929032	A	-0.0081	0.0014	0.0046	0.0020
rs2929860	T	0.0110	0.0018	-0.0048	0.0025
rs2942884	A	-0.0090	0.0014	0.0041	0.0020
rs2958182	A	0.0082	0.0015	0.0009	0.0021
rs2964255	A	0.0088	0.0015	0.0004	0.0021
rs2976397	T	0.0081	0.0014	-0.0080	0.0020
rs2977464	T	0.0102	0.0018	-0.0010	0.0025
rs29792	A	-0.0093	0.0015	0.0033	0.0021
rs2980813	A	0.0088	0.0014	0.0011	0.0020
rs2989476	C	0.0078	0.0014	-0.0033	0.0020
rs3111251	T	-0.0090	0.0014	0.0014	0.0020
rs312927	A	-0.0147	0.0022	0.0033	0.0031
rs322614	A	-0.0087	0.0014	-0.0002	0.0020
rs339057	A	-0.0081	0.0014	0.0019	0.0020
rs341504	A	0.0099	0.0015	-0.0034	0.0021
rs34309	A	0.0080	0.0014	0.0069	0.0020
rs34410	C	-0.0083	0.0014	0.0090	0.0020
rs347661	T	-0.0081	0.0014	0.0084	0.0020
rs350281	T	-0.0117	0.0015	0.0027	0.0021
rs356999	A	-0.0101	0.0014	0.0080	0.0020
rs363096	T	-0.0143	0.0014	0.0043	0.0020
rs3735478	T	0.0108	0.0016	-0.0124	0.0021
rs3768480	C	0.0101	0.0014	-0.0042	0.0020
rs3781339	T	-0.0104	0.0018	0.0054	0.0025
rs3790609	T	0.0107	0.0019	-0.0043	0.0026
rs3809634	A	-0.0109	0.0015	-0.0050	0.0022
rs3812281	T	0.0113	0.0014	0.0034	0.0020
rs382196	T	-0.0082	0.0014	-0.0032	0.0020
rs3847228	T	0.0083	0.0014	-0.0028	0.0020
rs3848715	A	-0.0080	0.0014	-0.0037	0.0020
rs3859523	T	0.0124	0.0018	-0.0027	0.0026
rs3895736	A	0.0144	0.0019	0.0050	0.0026

rs3897821	A	0.0154	0.0015	-0.0058	0.0021
rs3948495	T	-0.0089	0.0014	0.0091	0.0020
rs399821	A	-0.0078	0.0014	0.0037	0.0020
rs39998	A	0.0090	0.0015	0.0008	0.0022
rs4127499	A	0.0087	0.0015	-0.0042	0.0020
rs42302	A	0.0086	0.0015	-0.0268	0.0021
rs4255791	A	-0.0084	0.0015	0.0057	0.0021
rs429150	T	0.0085	0.0014	0.0002	0.0020
rs4298514	T	0.0104	0.0015	0.0091	0.0021
rs4320563	A	0.0109	0.0014	-0.0062	0.0020
rs4328757	T	0.0096	0.0014	-0.0058	0.0020
rs4358081	A	-0.0095	0.0014	-0.0098	0.0019
rs4396896	A	-0.0107	0.0014	0.0137	0.0020
rs4423373	A	-0.0087	0.0014	0.0007	0.0020
rs4434676	A	-0.0089	0.0014	0.0005	0.0020
rs4458044	C	0.0101	0.0016	-0.0027	0.0023
rs4480339	A	-0.0097	0.0015	-0.0052	0.0021
rs4490539	A	0.0142	0.0015	0.0060	0.0021
rs4500930	T	-0.0109	0.0015	0.0168	0.0020
rs4500960	T	-0.0131	0.0014	-0.0045	0.0020
rs4502401	T	0.0085	0.0015	-0.0008	0.0021
rs4658019	T	0.0084	0.0014	-0.0077	0.0020
rs4673840	T	-0.0138	0.0019	0.0078	0.0027
rs4712371	A	-0.0124	0.0021	0.0051	0.0030
rs4719944	T	-0.0121	0.0014	-0.0058	0.0020
rs4724083	T	0.0083	0.0015	0.0071	0.0021
rs4726070	A	0.0122	0.0014	-0.0043	0.0020
rs4731413	A	0.0111	0.0017	-0.0072	0.0024
rs4735297	A	-0.0086	0.0015	0.0126	0.0021
rs4739235	A	0.0109	0.0019	-0.0107	0.0026
rs4757957	C	0.0133	0.0015	-0.0031	0.0021
rs4766424	C	0.0142	0.0021	-0.0111	0.0029
rs4785187	A	-0.0100	0.0017	0.0032	0.0024
rs4787028	T	0.0086	0.0015	0.0000	0.0021
rs481940	T	0.0100	0.0016	0.0062	0.0022
rs482787	T	0.0100	0.0015	-0.0144	0.0021
rs4839155	T	0.0115	0.0017	-0.0059	0.0023
rs4846010	A	0.0099	0.0018	0.0000	0.0025
rs4846724	A	0.0098	0.0014	-0.0072	0.0020
rs4851263	A	-0.0151	0.0023	0.0065	0.0032
rs4877516	A	-0.0101	0.0014	0.0046	0.0020
rs4881269	A	0.0100	0.0014	-0.0042	0.0020
rs4904523	A	-0.0082	0.0014	0.0047	0.0020
rs4919624	A	0.0193	0.0018	0.0067	0.0024
rs4925065	T	-0.0078	0.0014	-0.0032	0.0020
rs4925109	A	-0.0096	0.0015	-0.0054	0.0021

rs4938815	T	0.0085	0.0015	0.0019	0.0021
rs4941735	T	0.0092	0.0014	-0.0006	0.0020
rs4972748	T	0.0105	0.0018	-0.0010	0.0025
rs4984541	A	-0.0138	0.0017	0.0048	0.0023
rs4984613	T	0.0174	0.0028	-0.0004	0.0040
rs4984682	C	-0.0138	0.0017	0.0045	0.0023
rs548897	A	0.0081	0.0014	-0.0013	0.0020
rs563954	A	-0.0081	0.0014	-0.0023	0.0020
rs567003	A	0.0094	0.0015	-0.0002	0.0021
rs5754581	T	-0.0082	0.0014	0.0057	0.0020
rs5754762	A	0.0197	0.0030	-0.0073	0.0043
rs6020560	T	-0.0083	0.0014	0.0037	0.0020
rs6065080	T	-0.0130	0.0015	-0.0052	0.0021
rs6091570	A	0.0081	0.0015	-0.0119	0.0021
rs613872	T	-0.0163	0.0019	0.0141	0.0026
rs631287	A	0.0084	0.0014	-0.0038	0.0020
rs635754	A	0.0119	0.0014	0.0081	0.0020
rs6428587	T	0.0084	0.0015	0.0004	0.0020
rs6435326	A	-0.0091	0.0014	0.0017	0.0020
rs6442126	A	-0.0121	0.0015	0.0047	0.0021
rs6445633	A	0.0088	0.0015	-0.0012	0.0021
rs6461536	A	0.0088	0.0015	0.0002	0.0022
rs6469654	C	-0.0103	0.0017	0.0011	0.0024
rs6480234	T	-0.0084	0.0014	0.0040	0.0020
rs6493265	T	-0.0122	0.0014	0.0011	0.0020
rs6493275	A	0.0131	0.0017	0.0063	0.0024
rs6503409	T	0.0087	0.0015	-0.0067	0.0021
rs6534338	T	0.0103	0.0015	-0.0031	0.0022
rs6557171	T	-0.0155	0.0015	0.0016	0.0021
rs6567288	A	-0.0079	0.0014	0.0061	0.0020
rs6689641	A	0.0078	0.0014	0.0030	0.0020
rs6695132	T	0.0097	0.0017	-0.0069	0.0024
rs6696068	T	-0.0088	0.0014	0.0044	0.0020
rs6704768	A	-0.0116	0.0014	-0.0008	0.0020
rs6706275	T	0.0083	0.0015	-0.0035	0.0021
rs6715321	T	-0.0097	0.0014	0.0048	0.0020
rs6720515	A	0.0103	0.0017	-0.0092	0.0024
rs6729612	T	0.0092	0.0015	0.0002	0.0021
rs6731373	A	-0.0115	0.0015	0.0032	0.0020
rs6736025	T	0.0079	0.0014	-0.0156	0.0020
rs6743032	A	-0.0165	0.0024	0.0027	0.0033
rs6744040	A	-0.0080	0.0014	0.0049	0.0020
rs6752228	T	-0.0080	0.0014	0.0061	0.0020
rs6757087	T	-0.0085	0.0014	0.0034	0.0020
rs6780414	T	0.0105	0.0018	-0.0003	0.0025
rs6792805	A	-0.0090	0.0016	-0.0016	0.0022

rs6824567	T	0.0130	0.0016	-0.0081	0.0022
rs6861925	C	-0.0090	0.0014	-0.0007	0.0020
rs6871635	A	-0.0087	0.0014	0.0084	0.0020
rs6881733	T	-0.0086	0.0016	-0.0082	0.0022
rs6917154	T	0.0127	0.0021	-0.0081	0.0029
rs6917204	T	0.0119	0.0017	0.0026	0.0025
rs6918506	C	0.0113	0.0014	-0.0032	0.0020
rs6932108	C	-0.0210	0.0027	0.0014	0.0035
rs6946136	A	0.0081	0.0014	0.0054	0.0020
rs6969783	A	-0.0098	0.0014	0.0026	0.0020
rs6977237	T	0.0082	0.0014	-0.0063	0.0020
rs6994287	A	0.0102	0.0014	0.0005	0.0020
rs7016302	C	-0.0125	0.0019	-0.0014	0.0026
rs7030373	A	-0.0098	0.0017	0.0022	0.0024
rs7035315	A	-0.0090	0.0015	-0.0039	0.0020
rs7041702	A	-0.0111	0.0016	-0.0024	0.0022
rs7108020	A	0.0111	0.0015	-0.0014	0.0021
rs7127580	C	-0.0150	0.0023	0.0126	0.0031
rs713584	A	-0.0080	0.0014	0.0010	0.0020
rs7158218	A	-0.0090	0.0015	0.0033	0.0021
rs716513	A	0.0084	0.0014	0.0043	0.0020
rs7171405	A	-0.0101	0.0016	0.0062	0.0023
rs717996	T	-0.0122	0.0014	0.0048	0.0020
rs7182216	C	-0.0096	0.0017	0.0017	0.0023
rs721579	T	-0.0110	0.0016	0.0051	0.0022
rs7226824	T	-0.0078	0.0014	0.0060	0.0020
rs7254263	T	-0.0119	0.0016	0.0015	0.0022
rs7255223	A	0.0097	0.0016	-0.0110	0.0022
rs728054	A	-0.0128	0.0015	0.0060	0.0021
rs730384	A	0.0101	0.0014	-0.0020	0.0020
rs7321274	A	0.0114	0.0018	0.0027	0.0024
rs7323027	A	0.0130	0.0015	0.0041	0.0021
rs736281	T	0.0086	0.0014	-0.0038	0.0020
rs736471	T	0.0083	0.0014	0.0043	0.0020
rs737945	C	-0.0115	0.0014	-0.0022	0.0020
rs743316	T	0.0112	0.0017	-0.0034	0.0024
rs7560871	A	-0.0193	0.0027	0.0236	0.0038
rs7561705	A	0.0078	0.0014	0.0093	0.0019
rs7575637	A	0.0107	0.0014	-0.0028	0.0020
rs7597412	C	0.0088	0.0015	-0.0021	0.0020
rs7625428	T	0.0098	0.0014	-0.0007	0.0020
rs763553	A	0.0087	0.0014	-0.0054	0.0020
rs7640424	T	0.0113	0.0015	-0.0128	0.0021
rs7672622	A	-0.0088	0.0016	0.0001	0.0023
rs767943	A	-0.0122	0.0016	0.0076	0.0023
rs7683416	T	0.0139	0.0014	-0.0001	0.0020

rs7692359	T	-0.0109	0.0017	0.0009	0.0024
rs7772172	A	0.0091	0.0014	0.0001	0.0020
rs7803932	A	0.0127	0.0019	-0.0002	0.0026
rs780569	A	-0.0085	0.0016	0.0004	0.0022
rs7849480	A	-0.0084	0.0014	0.0081	0.0020
rs7849487	T	-0.0167	0.0015	-0.0013	0.0021
rs7855503	C	0.0088	0.0015	0.0020	0.0020
rs7875078	A	-0.0078	0.0014	0.0061	0.0020
rs7894722	T	0.0085	0.0014	-0.0019	0.0020
rs7899270	A	-0.0084	0.0015	0.0030	0.0021
rs790647	A	-0.0145	0.0017	0.0007	0.0023
rs7910403	T	0.0122	0.0018	-0.0057	0.0025
rs7920624	A	0.0120	0.0014	-0.0050	0.0020
rs7931563	T	-0.0109	0.0014	0.0016	0.0020
rs795230	T	0.0081	0.0014	0.0024	0.0020
rs7958371	A	-0.0085	0.0015	0.0053	0.0021
rs7965154	A	-0.0085	0.0015	0.0033	0.0021
rs7974852	A	0.0117	0.0014	-0.0075	0.0020
rs7977614	A	-0.0134	0.0016	-0.0001	0.0021
rs8009933	A	0.0088	0.0015	0.0033	0.0021
rs8024	A	-0.0115	0.0015	0.0237	0.0021
rs8052523	T	-0.0084	0.0014	0.0069	0.0020
rs8097125	T	0.0086	0.0014	-0.0036	0.0020
rs852771	T	0.0092	0.0015	-0.0034	0.0021
rs870589	A	-0.0079	0.0014	0.0009	0.0020
rs884108	A	-0.0094	0.0017	0.0006	0.0023
rs893522	A	0.0149	0.0025	0.0019	0.0034
rs902820	A	-0.0096	0.0014	0.0016	0.0020
rs911149	T	0.0091	0.0017	-0.0033	0.0023
rs912883	T	0.0087	0.0015	0.0031	0.0021
rs913509	A	0.0096	0.0015	-0.0077	0.0021
rs9267658	T	0.0138	0.0020	-0.0083	0.0027
rs9267677	T	0.0180	0.0024	-0.0236	0.0033
rs9291437	C	-0.0081	0.0014	-0.0002	0.0020
rs9294770	T	0.0083	0.0014	-0.0059	0.0020
rs9300612	T	0.0084	0.0015	-0.0005	0.0021
rs9349956	A	-0.0159	0.0018	-0.0016	0.0025
rs9373363	A	-0.0111	0.0016	0.0064	0.0023
rs9375403	C	0.0126	0.0016	-0.0090	0.0023
rs9386110	T	-0.0163	0.0023	-0.0030	0.0032
rs9388490	T	0.0091	0.0014	-0.0034	0.0020
rs939400	T	-0.0095	0.0015	0.0050	0.0020
rs9411331	A	0.0145	0.0015	-0.0106	0.0021
rs9442750	A	-0.0089	0.0016	-0.0043	0.0022
rs9490512	A	-0.0136	0.0014	0.0069	0.0020
rs9492774	C	0.0080	0.0015	-0.0039	0.0020

rs9513416	A	-0.0116	0.0019	0.0084	0.0027
rs9527905	A	-0.0094	0.0014	0.0104	0.0020
rs9540718	A	-0.0089	0.0014	0.0067	0.0020
rs9545395	T	0.0138	0.0021	-0.0026	0.0029
rs9556958	T	-0.0110	0.0014	0.0100	0.0020
rs9563168	A	0.0101	0.0017	-0.0094	0.0024
rs9568798	T	-0.0093	0.0016	-0.0055	0.0022
rs9597907	T	0.0152	0.0027	-0.0089	0.0039
rs9611597	A	-0.0107	0.0019	0.0108	0.0027
rs9616906	A	0.0122	0.0014	0.0035	0.0020
rs9649	T	0.0108	0.0019	-0.0040	0.0026
rs9655780	A	-0.0153	0.0019	0.0031	0.0027
rs9666728	T	-0.0082	0.0014	0.0078	0.0020
rs9771228	T	0.0096	0.0015	-0.0116	0.0020
rs977143	A	0.0085	0.0015	0.0038	0.0020
rs978807	A	0.0139	0.0018	-0.0092	0.0025
rs981230	T	0.0100	0.0014	-0.0073	0.0020
rs981883	A	0.0099	0.0014	-0.0022	0.0020
rs9820604	T	0.0115	0.0018	0.0021	0.0026
rs9853928	T	-0.0127	0.0017	0.0063	0.0024
rs9858921	A	-0.0087	0.0014	0.0004	0.0020
rs9870317	A	0.0086	0.0015	-0.0048	0.0021
rs9882532	T	0.0124	0.0015	-0.0033	0.0021
rs9886703	A	-0.0136	0.0019	0.0063	0.0026
rs9926649	T	0.0177	0.0031	-0.0116	0.0045
rs9927842	T	-0.0130	0.0020	0.0093	0.0027
rs9929762	A	0.0096	0.0014	-0.0030	0.0020
rs9929993	T	-0.0102	0.0015	0.0055	0.0021
rs995698	A	-0.0099	0.0014	-0.0035	0.0020

Supplementary Table 8: Genetic association estimates for the IVW ratio-method MR analysis estimating the effect of education on CAD. ea=effect allele; gx=education; gy=CAD; se=standard error

SNP	ea	gx	gx_se	gy	gy_se
rs10006235	T	-0.0093	0.0016	0.0000	0.0101
rs10009513	A	0.0088	0.0014	-0.0071	0.0093
rs10021733	T	-0.0164	0.0027	0.0039	0.0141
rs10032941	T	-0.0080	0.0015	0.0149	0.0095
rs10041403	T	0.0151	0.0019	-0.0031	0.0131
rs10042828	A	-0.0145	0.0022	0.0149	0.0158
rs10046069	A	0.0127	0.0022	0.0008	0.0141
rs10057590	A	0.0106	0.0014	-0.0050	0.0095
rs10060023	T	0.0110	0.0015	-0.0028	0.0104
rs10061420	A	0.0082	0.0014	0.0014	0.0095
rs1006749	A	0.0079	0.0014	-0.0042	0.0097
rs10074178	A	0.0079	0.0014	0.0011	0.0094
rs1007731	A	-0.0143	0.0022	0.0114	0.0174
rs10080647	A	0.0124	0.0020	-0.0121	0.0125
rs1008078	T	-0.0182	0.0014	0.0027	0.0096
rs1009470	T	0.0086	0.0014	-0.0134	0.0092
rs10098073	A	-0.0091	0.0014	0.0002	0.0098
rs10122669	A	-0.0079	0.0014	-0.0017	0.0094
rs10128888	A	-0.0083	0.0015	0.0217	0.0105
rs10145520	T	-0.0122	0.0018	-0.0095	0.0119
rs10166286	T	0.0151	0.0019	-0.0065	0.0116
rs10169002	A	-0.0080	0.0014	-0.0030	0.0097
rs10181071	A	0.0102	0.0019	0.0033	0.0132
rs10189857	A	0.0158	0.0014	-0.0216	0.0097
rs10192369	A	-0.0078	0.0014	0.0024	0.0099
rs10192834	T	0.0083	0.0015	0.0021	0.0101
rs10193498	A	0.0097	0.0016	0.0031	0.0108
rs10204051	T	-0.0101	0.0014	0.0043	0.0101
rs10205057	T	0.0090	0.0014	-0.0110	0.0095
rs10208	T	0.0091	0.0015	-0.0022	0.0100
rs10215082	A	-0.0149	0.0014	0.0148	0.0098
rs1024268	T	-0.0084	0.0014	0.0060	0.0093
rs10251438	A	0.0078	0.0014	-0.0268	0.0093
rs10264573	A	0.0175	0.0032	0.0124	0.0216
rs1035578	A	-0.0094	0.0014	-0.0080	0.0095
rs10402747	T	0.0077	0.0014	-0.0350	0.0098
rs10411759	A	0.0116	0.0020	-0.0050	0.0135
rs10444280	T	0.0100	0.0018	-0.0005	0.0119
rs10460095	A	-0.0124	0.0014	0.0065	0.0095
rs10480450	A	0.0158	0.0028	0.0051	0.0172
rs10496091	A	-0.0131	0.0016	0.0171	0.0113

rs10499535	A	0.0080	0.0014	0.0085	0.0093
rs1050847	T	0.0092	0.0014	-0.0262	0.0103
rs10509251	T	-0.0093	0.0017	0.0215	0.0105
rs10515007	T	0.0143	0.0019	-0.0127	0.0120
rs1051860	A	-0.0079	0.0014	0.0172	0.0096
rs10519504	T	0.0119	0.0019	-0.0111	0.0126
rs1054442	A	-0.0136	0.0015	0.0006	0.0097
rs1061801	A	-0.0110	0.0018	0.0157	0.0125
rs1066769	A	-0.0230	0.0041	-0.0249	0.0279
rs10742591	A	-0.0084	0.0014	0.0054	0.0095
rs10748841	A	-0.0111	0.0020	0.0085	0.0099
rs10750539	A	-0.0095	0.0015	0.0000	0.0098
rs10752262	T	0.0102	0.0014	0.0032	0.0097
rs1075228	A	-0.0081	0.0014	-0.0064	0.0101
rs10761202	T	0.0088	0.0014	0.0210	0.0094
rs10761251	A	0.0113	0.0015	0.0091	0.0096
rs10772644	C	0.0142	0.0022	-0.0022	0.0141
rs10773002	A	0.0185	0.0016	-0.0060	0.0104
rs10773208	T	-0.0110	0.0016	-0.0133	0.0110
rs10782651	T	-0.0082	0.0014	0.0070	0.0094
rs10783243	A	-0.0092	0.0014	0.0142	0.0093
rs10789285	T	0.0091	0.0016	-0.0128	0.0104
rs10793903	A	0.0126	0.0022	-0.0222	0.0140
rs10795831	T	-0.0099	0.0017	0.0186	0.0107
rs10798888	T	-0.0140	0.0019	0.0219	0.0117
rs10799615	A	-0.0089	0.0016	0.0190	0.0101
rs10805383	A	-0.0103	0.0014	0.0062	0.0094
rs10810099	A	-0.0146	0.0016	-0.0016	0.0101
rs10810145	A	0.0078	0.0014	-0.0081	0.0094
rs10830858	T	0.0092	0.0014	-0.0107	0.0093
rs10831656	T	0.0090	0.0015	-0.0069	0.0103
rs10844179	A	0.0104	0.0017	-0.0107	0.0110
rs10845051	A	0.0083	0.0014	-0.0052	0.0096
rs10875121	C	0.0174	0.0019	-0.0198	0.0127
rs10877283	T	0.0085	0.0014	0.0005	0.0096
rs10879676	T	-0.0083	0.0014	-0.0037	0.0095
rs10886012	C	0.0078	0.0014	0.0011	0.0093
rs10892807	T	0.0109	0.0014	0.0093	0.0099
rs10899282	A	0.0140	0.0017	-0.0071	0.0114
rs10927053	A	-0.0125	0.0022	0.0099	0.0138
rs10928190	T	-0.0087	0.0014	0.0083	0.0096
rs10931821	A	-0.0141	0.0014	0.0111	0.0096
rs10937240	T	-0.0123	0.0018	-0.0047	0.0110
rs10940540	A	0.0078	0.0014	0.0162	0.0096
rs10940921	T	0.0111	0.0014	0.0057	0.0093
rs10943588	A	0.0084	0.0015	-0.0224	0.0095

rs10947439	T	0.0090	0.0015	-0.0067	0.0100
rs10949263	A	-0.0091	0.0016	-0.0098	0.0104
rs10951590	T	-0.0109	0.0015	0.0154	0.0099
rs10974256	A	-0.0093	0.0015	-0.0046	0.0106
rs1097784	T	-0.0083	0.0014	0.0066	0.0094
rs10979613	T	-0.0117	0.0015	0.0037	0.0096
rs10983324	A	0.0084	0.0015	-0.0141	0.0100
rs10984445	A	0.0115	0.0014	-0.0191	0.0092
rs10985402	T	0.0089	0.0016	-0.0078	0.0103
rs10995639	T	0.0078	0.0014	-0.0155	0.0094
rs10996167	C	0.0113	0.0020	0.0077	0.0098
rs11003463	T	0.0091	0.0015	-0.0096	0.0112
rs11021432	A	0.0103	0.0015	0.0147	0.0102
rs11023764	A	0.0091	0.0015	0.0202	0.0102
rs11030084	T	0.0106	0.0018	-0.0306	0.0116
rs11030102	C	0.0101	0.0016	-0.0261	0.0118
rs1106090	A	0.0094	0.0014	-0.0052	0.0096
rs11076962	T	0.0086	0.0016	0.0059	0.0101
rs1107871	A	-0.0083	0.0014	0.0098	0.0092
rs11081529	T	0.0126	0.0015	-0.0138	0.0103
rs11082011	T	0.0194	0.0015	0.0090	0.0097
rs11100237	A	-0.0101	0.0014	0.0104	0.0092
rs11115056	A	0.0187	0.0023	-0.0033	0.0182
rs11121177	A	0.0151	0.0018	-0.0083	0.0118
rs111226181	T	-0.0201	0.0018	0.0246	0.0132
rs111235962	T	0.0133	0.0024	-0.0029	0.0165
rs11130380	T	0.0090	0.0014	0.0138	0.0098
rs111370527	T	0.0151	0.0027	0.0289	0.0245
rs11138947	T	0.0105	0.0016	-0.0204	0.0099
rs111530150	T	0.0269	0.0048	0.0530	0.0324
rs11155813	T	-0.0129	0.0023	-0.0298	0.0133
rs11157931	A	-0.0139	0.0014	-0.0210	0.0098
rs11158800	A	-0.0091	0.0014	0.0158	0.0096
rs1117152	T	0.0106	0.0015	-0.0090	0.0099
rs11174399	A	0.0111	0.0018	-0.0033	0.0116
rs111821073	T	0.0124	0.0019	-0.0141	0.0138
rs111852224	T	0.0156	0.0022	0.0105	0.0174
rs11190955	T	-0.0083	0.0015	0.0018	0.0101
rs11191237	T	0.0132	0.0024	-0.0119	0.0112
rs1120924	T	0.0108	0.0015	-0.0031	0.0102
rs11209894	A	0.0119	0.0021	-0.0090	0.0130
rs11210228	T	-0.0105	0.0019	-0.0001	0.0098
rs11210400	A	0.0120	0.0014	0.0095	0.0096
rs11211123	A	0.0104	0.0017	0.0060	0.0110
rs11213482	A	-0.0106	0.0019	0.0016	0.0147
rs112210983	A	0.0105	0.0017	0.0012	0.0119

rs1123285	C	-0.0094	0.0015	0.0055	0.0100
rs112375785	A	0.0184	0.0021	-0.0084	0.0180
rs112379405	C	-0.0082	0.0014	-0.0123	0.0095
rs112512729	T	-0.0166	0.0027	0.0142	0.0208
rs112603734	A	-0.0131	0.0022	0.0041	0.0117
rs11265191	T	0.0083	0.0015	-0.0042	0.0096
rs112806496	C	-0.0160	0.0025	0.0019	0.0191
rs1128956	T	-0.0140	0.0019	0.0188	0.0134
rs113205706	A	-0.0135	0.0024	-0.0106	0.0184
rs113520408	A	0.0155	0.0016	-0.0100	0.0118
rs113552169	C	0.0294	0.0053	-0.0428	0.0383
rs113588399	T	-0.0104	0.0017	0.0052	0.0124
rs113615161	T	-0.0123	0.0021	0.0210	0.0134
rs113720505	A	0.0164	0.0029	0.0022	0.0264
rs113731629	T	0.0339	0.0061	-0.0205	0.0497
rs114192810	T	-0.0279	0.0051	0.0375	0.0353
rs114468556	A	0.0293	0.0036	-0.0511	0.0320
rs114593137	A	-0.0122	0.0017	0.0080	0.0122
rs1146079	C	-0.0087	0.0016	0.0050	0.0109
rs114810763	A	-0.0121	0.0020	-0.0014	0.0162
rs114952970	T	0.0265	0.0040	0.0289	0.0304
rs114958262	T	-0.0220	0.0039	-0.0194	0.0338
rs115000530	A	-0.0250	0.0031	0.0537	0.0237
rs115017135	A	-0.0206	0.0038	0.0049	0.0309
rs11542663	A	0.0112	0.0015	0.0065	0.0098
rs115438240	T	-0.0169	0.0030	0.0315	0.0244
rs115693355	A	0.0309	0.0053	-0.0115	0.0428
rs115732722	A	-0.0415	0.0072	-0.0805	0.0530
rs11588857	A	0.0199	0.0017	0.0190	0.0110
rs11591870	T	0.0098	0.0017	-0.0100	0.0128
rs11592299	A	0.0097	0.0018	0.0220	0.0129
rs11596387	T	0.0258	0.0046	0.0273	0.0404
rs11598765	A	-0.0106	0.0018	0.0012	0.0112
rs11599236	T	-0.0106	0.0014	0.0126	0.0094
rs11613431	A	-0.0094	0.0016	-0.0097	0.0114
rs11620355	A	0.0182	0.0025	-0.0175	0.0176
rs11623285	T	-0.0135	0.0021	0.0199	0.0146
rs116386746	C	0.0249	0.0045	-0.0102	0.0397
rs11640569	A	0.0112	0.0020	0.0023	0.0144
rs11644446	A	0.0117	0.0019	0.0156	0.0145
rs11647188	A	0.0079	0.0014	0.0022	0.0097
rs11652522	A	-0.0154	0.0023	0.0168	0.0156
rs11657342	A	0.0140	0.0019	-0.0018	0.0131
rs11657979	A	-0.0091	0.0017	0.0316	0.0113
rs11663602	A	-0.0130	0.0016	-0.0032	0.0100
rs11663678	T	-0.0121	0.0022	-0.0047	0.0149

rs116656374	T	-0.0242	0.0038	0.0022	0.0302
rs11675476	T	-0.0127	0.0014	0.0131	0.0094
rs1167827	A	0.0104	0.0014	-0.0116	0.0098
rs11678980	A	-0.0166	0.0014	0.0092	0.0106
rs11687736	A	-0.0105	0.0015	-0.0004	0.0102
rs11693885	A	-0.0091	0.0014	0.0104	0.0097
rs116967397	A	0.0315	0.0056	-0.0109	0.0439
rs117005905	T	0.0150	0.0022	0.0007	0.0140
rs11703948	A	-0.0168	0.0024	0.0081	0.0156
rs1171040	A	-0.0113	0.0017	0.0302	0.0113
rs11716398	A	0.0159	0.0021	0.0144	0.0131
rs11720093	T	0.0150	0.0026	0.0168	0.0177
rs11720985	T	0.0085	0.0015	-0.0046	0.0106
rs11724690	T	0.0093	0.0015	0.0187	0.0107
rs117273411	T	-0.0211	0.0034	0.0317	0.0297
rs11732160	A	0.0105	0.0016	0.0071	0.0106
rs11732657	A	-0.0126	0.0016	0.0074	0.0105
rs11733439	A	-0.0096	0.0017	0.0127	0.0108
rs11736863	A	0.0142	0.0018	0.0075	0.0122
rs117398064	C	-0.0141	0.0025	0.0045	0.0179
rs117468730	A	-0.0310	0.0050	0.0154	0.0322
rs117623407	A	-0.0118	0.0020	-0.0102	0.0147
rs11765387	T	0.0086	0.0015	0.0001	0.0095
rs117668569	T	0.0234	0.0042	-0.0318	0.0293
rs117677995	A	-0.0180	0.0032	0.0345	0.0300
rs11772232	T	0.0177	0.0019	-0.0276	0.0132
rs11774212	T	0.0128	0.0014	-0.0057	0.0099
rs117799466	C	0.0106	0.0016	-0.0111	0.0124
rs11780023	T	0.0085	0.0014	-0.0012	0.0094
rs11789013	T	0.0106	0.0016	-0.0157	0.0106
rs117895796	T	0.0362	0.0064	-0.0004	0.0517
rs118040169	A	-0.0248	0.0038	-0.0110	0.0313
rs118093058	T	0.0146	0.0021	0.0149	0.0163
rs118134876	T	-0.0223	0.0030	0.0521	0.0239
rs11845781	T	-0.0079	0.0014	-0.0050	0.0094
rs11871429	A	0.0128	0.0017	0.0050	0.0107
rs11876620	T	0.0143	0.0024	0.0054	0.0165
rs11894424	A	0.0209	0.0033	-0.0161	0.0174
rs11917701	A	-0.0087	0.0014	0.0068	0.0094
rs11919835	C	0.0089	0.0015	-0.0215	0.0099
rs1196760	C	0.0139	0.0025	0.0113	0.0192
rs12005151	A	0.0087	0.0015	0.0051	0.0103
rs12028010	T	0.0164	0.0017	-0.0118	0.0114
rs12028229	T	0.0099	0.0016	0.0225	0.0123
rs12054166	C	0.0096	0.0016	-0.0113	0.0110
rs12076635	C	0.0207	0.0017	-0.0143	0.0106

rs12113634	T	0.0088	0.0015	-0.0117	0.0095
rs12123293	T	0.0086	0.0015	-0.0026	0.0098
rs12126231	A	0.0095	0.0014	-0.0097	0.0094
rs12127928	T	0.0108	0.0018	-0.0100	0.0128
rs12145078	A	-0.0127	0.0017	0.0063	0.0113
rs12151248	T	-0.0149	0.0023	-0.0062	0.0185
rs12170452	A	0.0105	0.0014	-0.0009	0.0097
rs1220779	A	-0.0079	0.0014	0.0149	0.0095
rs12234369	A	0.0087	0.0015	0.0070	0.0101
rs12238011	T	-0.0141	0.0026	0.0081	0.0157
rs12273435	A	-0.0132	0.0017	0.0216	0.0120
rs12285074	A	0.0161	0.0023	0.0164	0.0127
rs12304188	A	0.0111	0.0019	-0.0035	0.0117
rs12325727	A	0.0088	0.0014	-0.0330	0.0096
rs12332731	A	0.0135	0.0018	-0.0142	0.0131
rs12342546	A	-0.0128	0.0017	-0.0058	0.0109
rs12359372	T	-0.0092	0.0015	0.0232	0.0101
rs12375949	T	-0.0138	0.0014	0.0044	0.0094
rs12405889	T	-0.0098	0.0014	0.0002	0.0092
rs12431682	T	-0.0093	0.0015	-0.0043	0.0098
rs12438177	A	0.0093	0.0015	-0.0192	0.0094
rs12453682	T	0.0102	0.0015	-0.0246	0.0103
rs1245829	A	-0.0099	0.0014	0.0179	0.0101
rs12467175	T	-0.0081	0.0014	0.0250	0.0096
rs12468040	T	0.0137	0.0014	0.0026	0.0094
rs12473986	T	-0.0085	0.0015	0.0139	0.0102
rs12477385	T	0.0109	0.0017	-0.0231	0.0117
rs12478156	T	0.0108	0.0015	0.0129	0.0098
rs12503522	T	-0.0113	0.0016	0.0172	0.0102
rs12506221	T	-0.0130	0.0014	0.0165	0.0092
rs12516990	C	-0.0099	0.0017	0.0103	0.0122
rs12519073	T	-0.0107	0.0017	-0.0098	0.0112
rs12524795	T	0.0102	0.0014	-0.0111	0.0097
rs12568153	T	-0.0091	0.0015	0.0117	0.0104
rs12571549	A	0.0151	0.0020	-0.0072	0.0119
rs12574281	A	-0.0080	0.0015	-0.0070	0.0102
rs12591647	T	-0.0152	0.0018	0.0175	0.0112
rs12601380	A	-0.0082	0.0014	0.0061	0.0096
rs12602286	T	0.0160	0.0021	-0.0306	0.0121
rs12613500	C	0.0098	0.0014	-0.0058	0.0096
rs12614263	A	0.0083	0.0014	-0.0080	0.0095
rs12620796	A	0.0121	0.0019	-0.0140	0.0102
rs12638072	A	-0.0107	0.0015	0.0017	0.0105
rs12643771	T	0.0130	0.0015	-0.0079	0.0105
rs12646216	T	0.0084	0.0014	-0.0173	0.0098
rs12646523	T	-0.0132	0.0016	0.0036	0.0104

rs12659776	T	0.0085	0.0014	-0.0186	0.0092
rs12670376	A	0.0092	0.0014	-0.0129	0.0092
rs1267062	C	0.0113	0.0018	-0.0045	0.0128
rs12682775	T	-0.0119	0.0017	0.0034	0.0107
rs12694681	T	0.0102	0.0015	-0.0062	0.0105
rs12709186	A	-0.0091	0.0015	0.0062	0.0103
rs12712269	T	-0.0107	0.0014	0.0078	0.0097
rs12746551	C	0.0248	0.0041	0.0243	0.0301
rs12750688	C	0.0116	0.0016	0.0249	0.0117
rs12761729	T	-0.0098	0.0016	-0.0018	0.0118
rs12761761	T	0.0153	0.0017	-0.0011	0.0119
rs12764593	C	0.0202	0.0029	0.0015	0.0221
rs12765185	A	-0.0088	0.0016	0.0170	0.0114
rs12774577	T	0.0142	0.0020	-0.0175	0.0142
rs12789313	T	0.0092	0.0014	-0.0042	0.0094
rs12790196	T	-0.0101	0.0015	-0.0090	0.0103
rs12810587	T	0.0094	0.0015	-0.0080	0.0100
rs12875339	A	-0.0108	0.0015	-0.0007	0.0094
rs12888615	T	0.0120	0.0018	-0.0093	0.0122
rs12891042	T	0.0092	0.0014	-0.0041	0.0094
rs12891191	C	0.0080	0.0014	-0.0086	0.0099
rs12912465	T	-0.0104	0.0016	0.0348	0.0122
rs12952191	T	-0.0081	0.0014	0.0055	0.0094
rs12953422	A	-0.0080	0.0014	0.0152	0.0097
rs12957463	A	0.0146	0.0018	-0.0158	0.0115
rs12962845	C	-0.0123	0.0021	0.0138	0.0136
rs12967010	T	0.0114	0.0017	0.0002	0.0122
rs12970264	A	-0.0085	0.0014	0.0009	0.0098
rs12981405	T	-0.0112	0.0019	0.0003	0.0120
rs13009915	T	0.0212	0.0037	0.0401	0.0343
rs13010288	T	0.0198	0.0021	-0.0061	0.0154
rs13010566	A	-0.0091	0.0014	-0.0002	0.0097
rs13015496	C	0.0119	0.0018	-0.0113	0.0115
rs13016316	A	-0.0138	0.0024	-0.0091	0.0145
rs1301838	T	-0.0096	0.0015	0.0109	0.0105
rs13018640	T	-0.0215	0.0014	0.0133	0.0099
rs13026611	A	-0.0084	0.0015	-0.0221	0.0105
rs13029602	T	-0.0092	0.0014	-0.0050	0.0094
rs13034349	T	-0.0082	0.0014	0.0161	0.0103
rs13050131	A	-0.0081	0.0015	-0.0019	0.0099
rs13085461	C	0.0103	0.0014	-0.0051	0.0092
rs13099165	T	-0.0118	0.0017	-0.0218	0.0121
rs13107325	T	-0.0239	0.0027	-0.0067	0.0223
rs13117856	A	0.0084	0.0015	-0.0037	0.0105
rs13130765	C	-0.0091	0.0014	0.0363	0.0105
rs13133213	A	0.0096	0.0014	-0.0206	0.0092

rs13145650	T	-0.0173	0.0025	0.0246	0.0170
rs13154429	C	-0.0136	0.0023	0.0023	0.0153
rs13163062	T	0.0115	0.0014	0.0014	0.0095
rs13163845	T	-0.0149	0.0020	-0.0106	0.0136
rs13168136	A	-0.0092	0.0017	-0.0069	0.0105
rs13169187	A	-0.0079	0.0014	-0.0008	0.0094
rs13177031	A	-0.0085	0.0015	0.0059	0.0097
rs13190235	T	-0.0138	0.0024	-0.0034	0.0149
rs13197257	T	0.0109	0.0016	0.0027	0.0108
rs1320139	C	-0.0149	0.0014	0.0270	0.0096
rs13212041	T	0.0101	0.0018	-0.0069	0.0120
rs13240401	T	0.0176	0.0017	-0.0116	0.0113
rs13246220	T	-0.0093	0.0014	0.0027	0.0096
rs13261773	C	0.0122	0.0019	-0.0031	0.0120
rs13266287	A	-0.0085	0.0015	-0.0025	0.0099
rs13281564	A	0.0088	0.0014	0.0019	0.0094
rs13284516	C	-0.0082	0.0014	0.0024	0.0099
rs1329044	T	0.0134	0.0019	-0.0229	0.0120
rs1329125	T	-0.0097	0.0015	0.0186	0.0109
rs13296345	T	0.0090	0.0015	-0.0181	0.0094
rs13318986	A	0.0124	0.0015	-0.0055	0.0099
rs13327482	A	-0.0121	0.0018	-0.0006	0.0131
rs1334297	A	0.0257	0.0016	-0.0001	0.0111
rs1335482	T	0.0096	0.0014	-0.0066	0.0098
rs13361043	T	-0.0158	0.0028	0.0188	0.0164
rs13381557	A	-0.0087	0.0014	-0.0019	0.0093
rs13388333	A	0.0085	0.0015	-0.0050	0.0104
rs13397529	C	-0.0097	0.0017	0.0269	0.0116
rs13398860	A	0.0086	0.0015	-0.0140	0.0102
rs13402497	A	-0.0080	0.0014	-0.0087	0.0095
rs13422673	T	-0.0110	0.0014	0.0114	0.0098
rs13425585	C	-0.0089	0.0014	0.0109	0.0096
rs1368250	T	0.0250	0.0045	-0.0197	0.0416
rs137079	T	0.0128	0.0020	0.0185	0.0140
rs137858393	A	0.0193	0.0029	0.0343	0.0218
rs138096147	A	0.0234	0.0042	0.0714	0.0366
rs138484388	T	-0.0306	0.0051	0.0404	0.0419
rs1391513	T	-0.0095	0.0015	0.0032	0.0101
rs139244147	A	-0.0193	0.0030	0.0062	0.0186
rs1392816	T	0.0082	0.0014	-0.0101	0.0097
rs139980871	T	0.0180	0.0029	-0.0091	0.0210
rs1404549	A	-0.0089	0.0015	0.0127	0.0101
rs1405876	T	0.0109	0.0015	-0.0007	0.0102
rs140711597	C	0.0366	0.0055	0.0285	0.0425
rs1408284	C	-0.0127	0.0020	0.0146	0.0145
rs1408430	C	0.0096	0.0016	0.0068	0.0102

rs141586924	A	-0.0090	0.0015	0.0037	0.0109
rs141729694	T	0.0195	0.0026	0.0433	0.0175
rs142014757	A	0.0102	0.0018	0.0162	0.0137
rs1426619	T	0.0096	0.0014	0.0186	0.0092
rs1427298	T	0.0086	0.0014	-0.0091	0.0098
rs142747148	A	0.0329	0.0052	-0.0160	0.0432
rs1427829	A	-0.0083	0.0014	0.0146	0.0092
rs143148393	T	-0.0211	0.0039	0.0006	0.0307
rs1434630	T	-0.0137	0.0020	-0.0037	0.0124
rs143743568	A	0.0118	0.0020	-0.0119	0.0136
rs143812851	A	-0.0113	0.0019	-0.0071	0.0126
rs1440930	C	0.0081	0.0014	0.0137	0.0094
rs144336753	A	0.0388	0.0053	-0.0402	0.0396
rs1445591	A	0.0085	0.0015	0.0033	0.0111
rs145018899	A	0.0095	0.0015	0.0082	0.0109
rs1464297	T	-0.0109	0.0015	-0.0164	0.0098
rs1467737	T	-0.0084	0.0014	0.0167	0.0095
rs1485300	C	0.0098	0.0015	-0.0059	0.0100
rs1490612	T	0.0193	0.0026	0.0065	0.0173
rs150252215	A	0.0204	0.0035	0.0032	0.0263
rs150537577	A	-0.0151	0.0026	0.0265	0.0182
rs1505676	C	0.0084	0.0015	-0.0157	0.0094
rs151381	T	-0.0087	0.0014	0.0192	0.0094
rs1518890	T	-0.0090	0.0016	-0.0024	0.0104
rs1527878	A	-0.0105	0.0016	0.0266	0.0116
rs1529597	A	0.0223	0.0038	-0.0413	0.0250
rs1538389	T	-0.0109	0.0018	0.0179	0.0120
rs1542354	A	-0.0084	0.0014	-0.0115	0.0098
rs1544	A	-0.0096	0.0016	0.0064	0.0104
rs1554798	A	-0.0080	0.0014	0.0161	0.0098
rs1564347	T	0.0088	0.0015	-0.0246	0.0097
rs1569092	A	0.0160	0.0020	-0.0055	0.0134
rs1593022	T	-0.0099	0.0017	0.0061	0.0115
rs1603460	T	0.0090	0.0014	-0.0048	0.0093
rs162445	A	0.0152	0.0026	0.0037	0.0150
rs163229	C	-0.0260	0.0047	0.0412	0.0315
rs1637770	T	0.0191	0.0028	-0.0141	0.0192
rs164938	T	-0.0088	0.0014	0.0068	0.0095
rs165633	A	-0.0116	0.0016	0.0180	0.0116
rs1656614	C	0.0122	0.0015	-0.0016	0.0106
rs1671269	T	-0.0100	0.0016	0.0093	0.0101
rs1671770	A	0.0125	0.0018	0.0260	0.0118
rs16851779	T	-0.0153	0.0027	0.0285	0.0196
rs16871807	T	0.0090	0.0015	-0.0086	0.0098
rs1689510	C	0.0194	0.0015	0.0017	0.0103
rs16901689	T	0.0111	0.0019	-0.0238	0.0125

rs1693584	T	-0.0087	0.0015	0.0147	0.0100
rs16958559	T	0.0096	0.0017	0.0163	0.0128
rs16966271	T	0.0092	0.0016	-0.0066	0.0105
rs16975275	A	-0.0102	0.0017	-0.0210	0.0114
rs17048801	A	-0.0106	0.0014	0.0378	0.0101
rs17048855	A	0.0108	0.0015	-0.0013	0.0097
rs17069646	T	0.0085	0.0015	0.0058	0.0099
rs17110109	T	-0.0093	0.0014	0.0132	0.0095
rs17113730	A	-0.0170	0.0027	0.0090	0.0184
rs17131123	A	-0.0243	0.0039	-0.0364	0.0370
rs17133297	A	-0.0146	0.0023	0.0121	0.0137
rs17144467	A	0.0098	0.0015	-0.0032	0.0098
rs17148998	A	0.0101	0.0017	-0.0202	0.0124
rs171697	C	0.0133	0.0015	-0.0055	0.0101
rs17170519	C	0.0132	0.0023	-0.0066	0.0137
rs1717204	A	-0.0117	0.0018	0.0052	0.0127
rs1718188	A	0.0096	0.0014	-0.0215	0.0094
rs17186106	T	0.0108	0.0018	-0.0171	0.0129
rs17190418	T	-0.0213	0.0031	0.0198	0.0252
rs17205908	T	-0.0092	0.0015	0.0000	0.0110
rs17248751	A	-0.0136	0.0017	0.0250	0.0129
rs1728118	A	0.0089	0.0016	-0.0082	0.0101
rs1729412	T	-0.0107	0.0014	0.0012	0.0096
rs173003	A	-0.0082	0.0014	-0.0083	0.0096
rs1734370	A	0.0099	0.0016	-0.0034	0.0101
rs1738050	C	-0.0093	0.0014	-0.0024	0.0096
rs17411339	A	0.0140	0.0014	-0.0132	0.0100
rs17428076	C	0.0137	0.0016	0.0030	0.0114
rs17440885	A	0.0160	0.0029	0.0226	0.0222
rs1747714	T	0.0106	0.0014	0.0205	0.0093
rs1747817	T	-0.0092	0.0016	0.0317	0.0114
rs17489649	A	0.0127	0.0015	-0.0008	0.0112
rs17502934	T	-0.0167	0.0020	-0.0001	0.0134
rs175325	A	-0.0100	0.0014	0.0018	0.0095
rs17536059	C	-0.0108	0.0019	-0.0010	0.0130
rs17551064	A	0.0141	0.0019	0.0190	0.0132
rs17563464	A	-0.0147	0.0017	-0.0065	0.0145
rs17565975	A	-0.0106	0.0014	-0.0260	0.0093
rs17568389	A	0.0127	0.0014	-0.0073	0.0094
rs17574007	A	-0.0129	0.0021	-0.0027	0.0160
rs1758747	A	0.0092	0.0015	-0.0143	0.0100
rs17598675	T	-0.0116	0.0014	0.0084	0.0096
rs17604349	A	0.0127	0.0018	-0.0067	0.0122
rs17609255	T	-0.0087	0.0014	0.0225	0.0094
rs176218	T	0.0200	0.0018	-0.0340	0.0111
rs17622379	T	-0.0113	0.0018	-0.0106	0.0116

rs17650634	T	-0.0104	0.0019	0.0177	0.0138
rs17667540	A	-0.0087	0.0015	0.0034	0.0101
rs17669337	T	-0.0110	0.0014	0.0035	0.0097
rs17680712	T	-0.0092	0.0014	0.0083	0.0093
rs17686649	T	-0.0098	0.0017	0.0272	0.0113
rs17732878	T	-0.0095	0.0017	-0.0234	0.0115
rs17742342	A	-0.0119	0.0018	0.0192	0.0126
rs17747544	A	-0.0090	0.0014	0.0141	0.0095
rs1779549	A	0.0081	0.0014	0.0012	0.0096
rs178183	T	0.0145	0.0016	-0.0150	0.0106
rs17882802	A	0.0084	0.0014	-0.0017	0.0101
rs17883331	A	-0.0108	0.0018	0.0459	0.0129
rs1792602	A	-0.0097	0.0014	-0.0042	0.0097
rs181214	T	-0.0104	0.0017	0.0229	0.0132
rs182355396	A	-0.0525	0.0084	0.0223	0.0653
rs182902112	A	-0.0300	0.0053	0.0255	0.0335
rs1835339	T	0.0084	0.0014	0.0161	0.0098
rs183869217	C	0.0174	0.0030	-0.0025	0.0219
rs1841023	A	-0.0092	0.0015	-0.0064	0.0106
rs1842713	A	-0.0117	0.0017	0.0016	0.0108
rs1843815	A	0.0077	0.0014	-0.0185	0.0093
rs1861786	A	-0.0081	0.0014	0.0001	0.0094
rs1865955	T	0.0122	0.0019	0.0024	0.0114
rs1866823	A	0.0107	0.0014	-0.0172	0.0096
rs187580	T	-0.0104	0.0017	0.0255	0.0123
rs187951956	A	-0.0315	0.0058	0.0025	0.0376
rs1880088	A	-0.0097	0.0016	0.0236	0.0101
rs1880692	A	0.0086	0.0014	-0.0007	0.0093
rs188251563	A	0.0428	0.0070	-0.1372	0.0593
rs1890132	T	-0.0089	0.0015	0.0036	0.0106
rs1898111	A	0.0101	0.0018	-0.0010	0.0115
rs190102446	T	-0.0210	0.0037	-0.0366	0.0270
rs1905616	A	0.0083	0.0015	-0.0222	0.0098
rs1910005	T	-0.0090	0.0015	0.0004	0.0103
rs1918394	T	0.0119	0.0019	-0.0099	0.0121
rs192436652	T	-0.0319	0.0045	-0.0637	0.0385
rs1933264	T	0.0100	0.0016	0.0015	0.0105
rs1952183	A	-0.0089	0.0014	-0.0017	0.0095
rs1955250	A	-0.0140	0.0025	0.0145	0.0171
rs1963381	A	0.0099	0.0016	0.0081	0.0106
rs1980129	A	0.0085	0.0014	0.0055	0.0092
rs198262	T	-0.0223	0.0035	0.0309	0.0262
rs1991585	T	-0.0101	0.0015	-0.0013	0.0098
rs1995181	A	-0.0078	0.0014	-0.0143	0.0093
rs2007655	T	0.0086	0.0014	-0.0034	0.0092
rs2011603	A	-0.0091	0.0016	0.0314	0.0108

rs2014830	T	0.0110	0.0015	-0.0253	0.0102
rs201495	T	-0.0112	0.0019	0.0160	0.0101
rs2023016	C	0.0107	0.0017	-0.0097	0.0109
rs2024568	T	-0.0097	0.0016	0.0055	0.0105
rs2029401	A	-0.0091	0.0014	0.0223	0.0097
rs2034631	T	0.0089	0.0015	-0.0090	0.0096
rs2039204	A	0.0082	0.0014	-0.0052	0.0096
rs2043187	A	0.0107	0.0015	-0.0016	0.0108
rs2052285	A	0.0112	0.0014	-0.0100	0.0104
rs2055940	A	0.0082	0.0015	-0.0069	0.0109
rs2077235	T	0.0097	0.0017	0.0122	0.0107
rs2081652	A	0.0123	0.0015	0.0121	0.0102
rs2082317	T	-0.0078	0.0014	-0.0106	0.0095
rs2088913	A	0.0081	0.0014	0.0047	0.0092
rs2092248	T	-0.0083	0.0015	0.0054	0.0103
rs2098526	A	-0.0241	0.0043	0.0387	0.0255
rs2100249	T	-0.0089	0.0015	0.0108	0.0096
rs2126069	T	0.0080	0.0014	0.0021	0.0095
rs2131167	A	0.0080	0.0014	-0.0151	0.0097
rs214626	A	0.0101	0.0018	-0.0059	0.0112
rs2160514	A	-0.0094	0.0014	0.0040	0.0094
rs2179152	T	-0.0131	0.0015	-0.0022	0.0099
rs2183271	T	0.0084	0.0015	0.0002	0.0100
rs2199409	T	0.0103	0.0017	-0.0104	0.0117
rs2212430	T	-0.0100	0.0016	0.0018	0.0101
rs2216144	T	0.0098	0.0014	-0.0096	0.0093
rs2220926	T	-0.0095	0.0014	0.0021	0.0093
rs2250660	C	0.0080	0.0014	-0.0115	0.0098
rs2252098	T	-0.0077	0.0014	-0.0108	0.0096
rs2254681	A	0.0101	0.0016	-0.0104	0.0106
rs2256965	A	0.0106	0.0014	-0.0126	0.0101
rs2276209	A	0.0085	0.0015	-0.0043	0.0097
rs2283076	A	0.0115	0.0017	0.0021	0.0115
rs2287838	A	-0.0104	0.0014	-0.0010	0.0095
rs2290601	T	-0.0149	0.0017	0.0020	0.0106
rs2297293	C	0.0099	0.0015	0.0067	0.0100
rs2299156	T	0.0099	0.0018	-0.0065	0.0114
rs2314338	T	0.0093	0.0016	-0.0157	0.0121
rs2321157	A	-0.0081	0.0014	-0.0040	0.0093
rs232496	T	0.0090	0.0015	0.0301	0.0094
rs2332179	A	0.0121	0.0020	-0.0156	0.0128
rs2336721	T	0.0095	0.0015	-0.0409	0.0099
rs2358628	A	-0.0091	0.0015	0.0095	0.0100
rs236318	C	-0.0102	0.0017	-0.0042	0.0105
rs2364544	A	-0.0105	0.0014	0.0116	0.0097
rs2365376	A	0.0092	0.0015	0.0039	0.0098

rs2368831	T	-0.0092	0.0014	0.0017	0.0103
rs2373124	A	-0.0096	0.0016	0.0171	0.0116
rs2406253	A	0.0135	0.0018	0.0193	0.0129
rs2414072	A	-0.0083	0.0014	-0.0028	0.0093
rs2416214	A	0.0079	0.0014	-0.0021	0.0095
rs2416759	A	-0.0093	0.0015	0.0090	0.0102
rs2416845	T	0.0263	0.0048	-0.0091	0.0301
rs2431023	A	0.0093	0.0014	-0.0020	0.0093
rs2434672	A	0.0091	0.0014	0.0075	0.0095
rs2436760	T	0.0232	0.0042	0.0233	0.0265
rs2447097	T	0.0096	0.0014	0.0131	0.0097
rs2458370	T	-0.0086	0.0015	0.0173	0.0095
rs2469226	A	0.0096	0.0017	-0.0166	0.0115
rs2470966	C	-0.0103	0.0015	0.0059	0.0102
rs2478208	C	-0.0103	0.0014	-0.0101	0.0094
rs2496482	T	0.0121	0.0015	-0.0160	0.0104
rs2517086	C	-0.0088	0.0014	-0.0108	0.0099
rs2521602	A	-0.0266	0.0049	0.0022	0.0313
rs2522545	T	-0.0083	0.0014	0.0033	0.0094
rs2526398	C	-0.0222	0.0015	0.0174	0.0108
rs2529069	T	-0.0106	0.0016	0.0143	0.0101
rs252991	A	0.0095	0.0015	-0.0054	0.0098
rs2542673	A	0.0088	0.0015	0.0100	0.0100
rs2545795	A	0.0125	0.0014	0.0017	0.0094
rs255053	A	-0.0111	0.0018	-0.0014	0.0115
rs2554835	A	0.0088	0.0014	0.0060	0.0099
rs2568955	T	-0.0165	0.0016	0.0069	0.0112
rs2569041	T	0.0077	0.0014	-0.0061	0.0097
rs2570497	T	-0.0121	0.0015	0.0087	0.0098
rs2588959	T	-0.0080	0.0014	0.0056	0.0092
rs2657283	T	0.0085	0.0014	-0.0110	0.0097
rs2665668	A	-0.0094	0.0015	0.0227	0.0107
rs2702576	A	-0.0090	0.0014	0.0053	0.0094
rs2718277	T	0.0142	0.0026	-0.0179	0.0186
rs2718791	T	-0.0081	0.0015	-0.0148	0.0101
rs27220	A	-0.0114	0.0015	0.0027	0.0095
rs2725370	T	-0.0141	0.0015	0.0165	0.0102
rs2736752	T	0.0095	0.0017	-0.0032	0.0124
rs2740795	A	0.0098	0.0016	-0.0040	0.0102
rs2761438	A	0.0098	0.0015	-0.0145	0.0098
rs2764684	T	0.0143	0.0019	-0.0132	0.0141
rs2805064	C	0.0093	0.0016	0.0002	0.0111
rs2838006	T	0.0132	0.0015	-0.0117	0.0101
rs28482086	A	0.0086	0.0015	-0.0135	0.0097
rs28505285	C	0.0106	0.0018	0.0217	0.0130
rs28512462	C	0.0112	0.0014	-0.0021	0.0094

rs28513882	A	-0.0110	0.0018	-0.0014	0.0136
rs2852349	T	-0.0093	0.0014	0.0077	0.0092
rs28587776	T	0.0088	0.0015	0.0214	0.0102
rs28669886	A	-0.0084	0.0015	0.0128	0.0099
rs2870281	A	-0.0083	0.0014	-0.0050	0.0093
rs28735993	A	0.0162	0.0022	-0.0319	0.0158
rs2885198	A	0.0088	0.0014	-0.0207	0.0095
rs2898191	A	0.0093	0.0015	0.0116	0.0099
rs2910823	T	0.0115	0.0014	0.0215	0.0096
rs2916490	A	-0.0092	0.0015	0.0070	0.0103
rs2923424	A	0.0120	0.0014	0.0073	0.0095
rs2926702	T	-0.0124	0.0021	-0.0210	0.0122
rs2929032	A	-0.0081	0.0014	-0.0060	0.0092
rs2929860	T	0.0110	0.0018	0.0128	0.0132
rs2942884	A	-0.0090	0.0014	0.0047	0.0097
rs2954114	A	-0.0081	0.0014	0.0090	0.0099
rs2958182	A	0.0082	0.0015	-0.0216	0.0101
rs2962378	A	0.0101	0.0017	-0.0084	0.0110
rs2964199	T	-0.0099	0.0015	0.0172	0.0100
rs2964255	A	0.0088	0.0015	0.0012	0.0100
rs2976397	T	0.0081	0.0014	0.0117	0.0094
rs2977464	T	0.0102	0.0018	0.0009	0.0115
rs29792	A	-0.0093	0.0015	0.0016	0.0104
rs2980813	A	0.0088	0.0014	0.0037	0.0094
rs2989476	C	0.0078	0.0014	-0.0080	0.0093
rs2989751	A	-0.0092	0.0017	0.0104	0.0105
rs2992037	A	0.0149	0.0015	0.0170	0.0109
rs2994326	T	-0.0100	0.0018	-0.0036	0.0124
rs2998299	T	-0.0136	0.0017	-0.0019	0.0118
rs3026996	A	0.0138	0.0016	-0.0163	0.0122
rs303752	A	-0.0110	0.0014	0.0235	0.0104
rs3111251	T	-0.0090	0.0014	0.0000	0.0096
rs312927	A	-0.0147	0.0022	-0.0193	0.0169
rs312945	A	-0.0091	0.0015	-0.0306	0.0101
rs317050	T	0.0078	0.0014	-0.0071	0.0093
rs322614	A	-0.0087	0.0014	0.0102	0.0095
rs322744	T	-0.0090	0.0016	-0.0035	0.0102
rs324885	A	0.0142	0.0014	-0.0084	0.0096
rs32940	T	-0.0096	0.0015	0.0261	0.0102
rs339057	A	-0.0081	0.0014	-0.0052	0.0094
rs34067381	T	-0.0104	0.0015	0.0094	0.0098
rs34098770	A	-0.0160	0.0020	0.0023	0.0124
rs34106693	C	0.0159	0.0019	-0.0248	0.0154
rs34122915	C	0.0084	0.0014	-0.0006	0.0109
rs341504	A	0.0099	0.0015	-0.0048	0.0097
rs34155847	A	0.0135	0.0016	-0.0133	0.0112

rs34262657	T	-0.0240	0.0038	-0.0183	0.0214
rs34286836	T	-0.0089	0.0014	0.0080	0.0094
rs34298584	A	0.0111	0.0019	-0.0231	0.0128
rs34305371	A	0.0314	0.0024	0.0156	0.0182
rs34309	A	0.0080	0.0014	0.0056	0.0096
rs34316274	A	0.0097	0.0017	0.0140	0.0109
rs34363861	A	0.0087	0.0014	-0.0189	0.0104
rs34394051	A	-0.0117	0.0020	0.0166	0.0122
rs34410	C	-0.0083	0.0014	0.0204	0.0097
rs34624793	T	0.0116	0.0021	0.0200	0.0171
rs34720381	T	-0.0180	0.0024	-0.0028	0.0176
rs34748029	A	0.0185	0.0025	-0.0268	0.0227
rs347661	T	-0.0081	0.0014	-0.0124	0.0097
rs34780702	A	0.0111	0.0017	-0.0002	0.0106
rs34807077	A	0.0128	0.0019	-0.0060	0.0122
rs34811474	A	0.0104	0.0017	-0.0108	0.0150
rs34967082	A	-0.0080	0.0014	-0.0009	0.0098
rs35016816	T	0.0176	0.0023	-0.0186	0.0146
rs350281	T	-0.0117	0.0015	0.0181	0.0116
rs35104491	A	0.0124	0.0018	0.0009	0.0114
rs35111506	C	-0.0175	0.0030	0.0269	0.0214
rs35192107	T	-0.0091	0.0017	-0.0078	0.0119
rs35319653	T	0.0119	0.0015	0.0044	0.0101
rs35417702	T	-0.0152	0.0014	0.0188	0.0092
rs35493937	C	0.0124	0.0017	-0.0133	0.0114
rs35606437	A	0.0105	0.0016	-0.0144	0.0104
rs356999	A	-0.0101	0.0014	0.0148	0.0101
rs35745455	A	0.0080	0.0014	-0.0088	0.0095
rs35751693	T	-0.0246	0.0040	0.0328	0.0389
rs35754740	T	0.0083	0.0014	-0.0127	0.0094
rs35811586	T	0.0217	0.0031	-0.0516	0.0283
rs35929923	A	-0.0114	0.0016	0.0293	0.0108
rs36085856	C	0.0201	0.0036	-0.0029	0.0246
rs36120534	T	0.0100	0.0018	0.0119	0.0131
rs362307	T	-0.0226	0.0027	0.0177	0.0199
rs363096	T	-0.0143	0.0014	0.0182	0.0093
rs3735478	T	0.0108	0.0016	-0.0317	0.0109
rs3751331	A	-0.0097	0.0014	0.0212	0.0096
rs3766979	A	0.0085	0.0015	-0.0050	0.0097
rs3768480	C	0.0101	0.0014	-0.0218	0.0096
rs3781339	T	-0.0104	0.0018	0.0398	0.0123
rs3790609	T	0.0107	0.0019	0.0021	0.0125
rs3809169	T	0.0140	0.0025	0.0054	0.0162
rs3809634	A	-0.0109	0.0015	0.0074	0.0107
rs3812281	T	0.0113	0.0014	-0.0136	0.0094
rs3817923	A	-0.0121	0.0022	0.0320	0.0143

rs382196	T	-0.0082	0.0014	0.0082	0.0095
rs3847228	T	0.0083	0.0014	0.0078	0.0098
rs3848715	A	-0.0080	0.0014	0.0108	0.0092
rs3859523	T	0.0124	0.0018	-0.0257	0.0122
rs387027	A	-0.0083	0.0015	0.0230	0.0097
rs3895736	A	0.0144	0.0019	0.0170	0.0126
rs3897821	A	0.0154	0.0015	-0.0086	0.0098
rs3948495	T	-0.0089	0.0014	0.0135	0.0098
rs399821	A	-0.0078	0.0014	-0.0038	0.0093
rs39998	A	0.0090	0.0015	-0.0164	0.0108
rs401526	T	-0.0104	0.0014	0.0117	0.0093
rs401966	C	-0.0097	0.0014	-0.0216	0.0100
rs4127499	A	0.0087	0.0015	-0.0037	0.0107
rs41282553	A	0.0237	0.0040	-0.0510	0.0294
rs42210	C	-0.0098	0.0016	-0.0257	0.0101
rs42302	A	0.0086	0.0015	0.0006	0.0097
rs4255791	A	-0.0084	0.0015	-0.0018	0.0113
rs4263475	A	0.0078	0.0014	0.0076	0.0102
rs4283754	A	-0.0084	0.0014	-0.0055	0.0115
rs429150	T	0.0085	0.0014	-0.0129	0.0100
rs4298514	T	0.0104	0.0015	-0.0015	0.0099
rs4320563	A	0.0109	0.0014	-0.0206	0.0092
rs4328757	T	0.0096	0.0014	0.0058	0.0096
rs4352658	T	-0.0190	0.0026	0.0295	0.0167
rs4358081	A	-0.0095	0.0014	0.0104	0.0092
rs4358358	A	0.0109	0.0015	-0.0141	0.0101
rs4369924	A	0.0125	0.0019	-0.0103	0.0123
rs4384309	A	0.0102	0.0014	-0.0060	0.0098
rs4396896	A	-0.0107	0.0014	-0.0060	0.0094
rs4423373	A	-0.0087	0.0014	0.0060	0.0098
rs4426420	T	-0.0140	0.0019	0.0214	0.0124
rs4434676	A	-0.0089	0.0014	0.0157	0.0095
rs4458044	C	0.0101	0.0016	0.0077	0.0127
rs4467547	T	0.0121	0.0014	-0.0030	0.0104
rs4469771	T	0.0101	0.0015	-0.0129	0.0098
rs4480339	A	-0.0097	0.0015	0.0114	0.0108
rs4490539	A	0.0142	0.0015	-0.0002	0.0106
rs4497562	A	0.0112	0.0016	-0.0083	0.0105
rs4500930	T	-0.0109	0.0015	0.0377	0.0100
rs4500960	T	-0.0131	0.0014	0.0183	0.0096
rs4502401	T	0.0085	0.0015	-0.0074	0.0102
rs4652135	A	0.0103	0.0016	0.0057	0.0102
rs4658019	T	0.0084	0.0014	-0.0131	0.0094
rs4663617	A	0.0104	0.0017	-0.0018	0.0116
rs4664983	T	-0.0103	0.0018	0.0012	0.0115
rs4673840	T	-0.0138	0.0019	0.0077	0.0126

rs4675248	A	-0.0094	0.0014	-0.0034	0.0097
rs4685405	T	-0.0111	0.0018	-0.0020	0.0108
rs4687735	T	-0.0200	0.0033	-0.0421	0.0262
rs4691601	A	-0.0115	0.0014	0.0097	0.0093
rs4705763	C	0.0089	0.0014	0.0170	0.0095
rs4712371	A	-0.0124	0.0021	0.0114	0.0129
rs4719460	T	-0.0088	0.0015	0.0116	0.0100
rs4719944	T	-0.0121	0.0014	-0.0045	0.0092
rs4724083	T	0.0083	0.0015	-0.0098	0.0098
rs4726070	A	0.0122	0.0014	-0.0087	0.0094
rs4730020	T	0.0090	0.0016	-0.0003	0.0116
rs4731413	A	0.0111	0.0017	-0.0017	0.0127
rs4735297	A	-0.0086	0.0015	-0.0089	0.0097
rs4739235	A	0.0109	0.0019	-0.0087	0.0119
rs4741571	A	-0.0102	0.0015	0.0034	0.0107
rs4757957	C	0.0133	0.0015	-0.0131	0.0100
rs4766424	C	0.0142	0.0021	-0.0407	0.0161
rs4778058	T	-0.0092	0.0014	0.0038	0.0098
rs4785187	A	-0.0100	0.0017	-0.0155	0.0106
rs4787028	T	0.0086	0.0015	0.0053	0.0099
rs4787457	A	0.0162	0.0015	-0.0142	0.0100
rs4788115	A	0.0111	0.0019	-0.0242	0.0157
rs479018	A	0.0109	0.0015	-0.0002	0.0112
rs4793090	A	0.0084	0.0015	-0.0391	0.0096
rs4810894	A	-0.0085	0.0015	0.0165	0.0096
rs4812697	T	0.0171	0.0027	-0.0065	0.0188
rs481940	T	0.0100	0.0016	-0.0047	0.0113
rs482787	T	0.0100	0.0015	-0.0146	0.0100
rs4839155	T	0.0115	0.0017	-0.0089	0.0110
rs4846010	A	0.0099	0.0018	-0.0313	0.0130
rs4846724	A	0.0098	0.0014	-0.0108	0.0092
rs4848924	A	-0.0117	0.0015	-0.0051	0.0100
rs4850954	T	0.0077	0.0014	0.0030	0.0100
rs4851263	A	-0.0151	0.0023	0.0342	0.0157
rs4877516	A	-0.0101	0.0014	0.0157	0.0093
rs4881269	A	0.0100	0.0014	0.0199	0.0095
rs488476	C	-0.0113	0.0015	0.0014	0.0103
rs4894658	C	0.0091	0.0016	-0.0145	0.0102
rs4895650	T	0.0093	0.0014	0.0003	0.0101
rs4899012	C	-0.0103	0.0014	0.0144	0.0096
rs4904523	A	-0.0082	0.0014	0.0087	0.0093
rs4915735	A	0.0116	0.0020	0.0128	0.0142
rs4919624	A	0.0193	0.0018	-0.0195	0.0119
rs4925065	T	-0.0078	0.0014	0.0007	0.0095
rs4925109	A	-0.0096	0.0015	0.0296	0.0100
rs4938815	T	0.0085	0.0015	-0.0034	0.0102

rs4941735	T	0.0092	0.0014	-0.0027	0.0096
rs4972748	T	0.0105	0.0018	0.0039	0.0116
rs4977885	A	-0.0084	0.0014	0.0203	0.0097
rs4981245	A	0.0080	0.0014	-0.0107	0.0099
rs4984541	A	-0.0138	0.0017	0.0026	0.0115
rs4984613	T	0.0174	0.0028	-0.0009	0.0174
rs4984682	C	-0.0138	0.0017	-0.0042	0.0110
rs548897	A	0.0081	0.0014	-0.0128	0.0095
rs55675587	T	-0.0103	0.0018	0.0143	0.0115
rs55736314	C	-0.0154	0.0014	0.0216	0.0102
rs55826493	T	-0.0127	0.0021	0.0272	0.0145
rs55986781	T	0.0187	0.0026	-0.0499	0.0209
rs56085180	A	-0.0269	0.0037	0.0019	0.0238
rs56099375	T	0.0120	0.0017	-0.0083	0.0125
rs56171318	T	-0.0137	0.0020	-0.0096	0.0128
rs56174996	A	-0.0143	0.0020	0.0079	0.0136
rs56306882	A	0.0086	0.0016	-0.0188	0.0115
rs56319902	T	-0.0206	0.0017	0.0433	0.0136
rs56391344	A	0.0148	0.0016	-0.0328	0.0117
rs563954	A	-0.0081	0.0014	0.0044	0.0096
rs56405138	A	0.0153	0.0022	-0.0020	0.0139
rs56408528	T	-0.0082	0.0014	0.0115	0.0096
rs567003	A	0.0094	0.0015	0.0113	0.0103
rs56794817	A	0.0122	0.0019	-0.0068	0.0121
rs57016874	T	0.0209	0.0037	0.0050	0.0293
rs57148205	A	0.0095	0.0016	0.0069	0.0115
rs57204268	A	0.0114	0.0020	0.0000	0.0138
rs57349798	A	0.0093	0.0014	-0.0462	0.0100
rs57352738	A	-0.0152	0.0017	-0.0002	0.0123
rs57437407	A	-0.0145	0.0023	-0.0140	0.0181
rs575113	A	0.0133	0.0015	-0.0159	0.0104
rs5754581	T	-0.0082	0.0014	0.0127	0.0093
rs5754753	T	-0.0126	0.0016	0.0001	0.0106
rs5754762	A	0.0197	0.0030	-0.0235	0.0174
rs57661533	T	-0.0114	0.0021	0.0029	0.0120
rs580652	T	-0.0147	0.0024	-0.0112	0.0135
rs585557	A	0.0128	0.0018	-0.0220	0.0121
rs58779949	A	0.0110	0.0019	-0.0319	0.0137
rs58859557	T	0.0187	0.0028	0.0124	0.0192
rs58921703	T	0.0109	0.0015	-0.0045	0.0099
rs58950082	T	0.0095	0.0017	-0.0058	0.0112
rs58996896	A	-0.0094	0.0016	-0.0071	0.0113
rs590013	T	0.0100	0.0015	-0.0062	0.0099
rs59300999	T	-0.0164	0.0028	0.0078	0.0147
rs59480703	C	-0.0123	0.0018	-0.0235	0.0114
rs59813324	T	0.0097	0.0018	-0.0053	0.0111

rs59967356	T	-0.0127	0.0021	0.0258	0.0180
rs60096640	A	0.0159	0.0023	0.0005	0.0125
rs6020560	T	-0.0083	0.0014	-0.0057	0.0092
rs6043521	T	-0.0083	0.0014	0.0159	0.0100
rs60589532	A	0.0168	0.0028	-0.0078	0.0188
rs6060308	A	0.0095	0.0016	-0.0184	0.0103
rs6065080	T	-0.0130	0.0015	0.0042	0.0095
rs6065784	C	0.0110	0.0015	-0.0012	0.0104
rs60717745	C	-0.0118	0.0019	0.0148	0.0121
rs60726488	T	-0.0090	0.0016	0.0002	0.0111
rs6091570	A	0.0081	0.0015	-0.0086	0.0096
rs61104616	A	-0.0172	0.0014	-0.0007	0.0094
rs6123924	A	0.0129	0.0019	-0.0348	0.0119
rs613872	T	-0.0163	0.0019	0.0232	0.0131
rs61387839	A	0.0088	0.0015	-0.0123	0.0111
rs61527214	A	0.0110	0.0014	-0.0121	0.0101
rs61739710	A	-0.0099	0.0016	-0.0133	0.0106
rs61748951	A	-0.0273	0.0048	-0.0138	0.0458
rs61755388	T	0.0125	0.0017	-0.0005	0.0121
rs61757207	A	0.0359	0.0063	0.0794	0.0445
rs61798586	A	-0.0112	0.0020	0.0160	0.0141
rs61853335	T	-0.0109	0.0018	-0.0084	0.0116
rs61958175	A	-0.0198	0.0034	0.0285	0.0299
rs61997667	T	-0.0144	0.0020	0.0074	0.0135
rs62007304	A	0.0264	0.0047	-0.0001	0.0457
rs62018216	T	0.0113	0.0019	0.0087	0.0137
rs62051146	A	-0.0134	0.0022	0.0248	0.0139
rs62090515	A	-0.0090	0.0015	0.0059	0.0098
rs62092949	T	-0.0087	0.0014	-0.0132	0.0096
rs62103198	T	-0.0147	0.0025	0.0077	0.0208
rs62109862	A	0.0136	0.0020	0.0155	0.0129
rs62142891	A	0.0108	0.0016	0.0029	0.0117
rs62155350	A	0.0338	0.0053	-0.0257	0.0431
rs62155770	A	-0.0196	0.0032	0.0468	0.0270
rs62155873	T	-0.0151	0.0021	-0.0192	0.0158
rs62174974	A	-0.0105	0.0018	0.0115	0.0114
rs62177359	A	-0.0275	0.0038	0.0148	0.0300
rs62179650	A	0.0112	0.0016	0.0055	0.0107
rs62182994	T	0.0137	0.0015	-0.0077	0.0108
rs62190914	T	0.0089	0.0015	-0.0062	0.0103
rs62194170	A	0.0111	0.0017	-0.0125	0.0119
rs622169	T	0.0086	0.0014	-0.0114	0.0112
rs62247449	C	0.0114	0.0014	-0.0062	0.0097
rs62252819	A	-0.0094	0.0017	-0.0032	0.0113
rs62256284	T	0.0111	0.0017	0.0079	0.0113
rs62260764	C	-0.0132	0.0016	-0.0168	0.0109

rs62262671	A	0.0213	0.0020	-0.0199	0.0156
rs62340636	T	0.0103	0.0014	-0.0043	0.0094
rs62370510	A	-0.0109	0.0019	-0.0070	0.0133
rs62379838	T	0.0121	0.0015	-0.0192	0.0103
rs62409395	T	0.0101	0.0018	-0.0111	0.0167
rs62420387	T	-0.0184	0.0027	0.0316	0.0204
rs62506074	T	0.0091	0.0015	0.0058	0.0110
rs62506104	A	-0.0098	0.0018	0.0124	0.0115
rs628993	A	0.0128	0.0023	-0.0068	0.0145
rs631287	A	0.0084	0.0014	-0.0255	0.0092
rs633279	A	-0.0089	0.0015	-0.0061	0.0102
rs635754	A	0.0119	0.0014	-0.0004	0.0092
rs6428587	T	0.0084	0.0015	-0.0039	0.0095
rs6435326	A	-0.0091	0.0014	-0.0057	0.0097
rs6442126	A	-0.0121	0.0015	0.0301	0.0103
rs6445633	A	0.0088	0.0015	-0.0003	0.0112
rs6449503	A	0.0185	0.0014	0.0042	0.0095
rs6450476	A	-0.0116	0.0015	0.0075	0.0102
rs6452793	T	0.0175	0.0017	-0.0176	0.0111
rs6461536	A	0.0088	0.0015	0.0054	0.0102
rs6469654	C	-0.0103	0.0017	-0.0092	0.0104
rs6472208	T	-0.0091	0.0014	0.0039	0.0097
rs6480234	T	-0.0084	0.0014	0.0043	0.0094
rs648044	A	0.0083	0.0015	0.0155	0.0104
rs6490618	T	0.0088	0.0015	-0.0020	0.0098
rs6493265	T	-0.0122	0.0014	0.0137	0.0097
rs6493275	A	0.0131	0.0017	0.0108	0.0120
rs6503409	T	0.0087	0.0015	0.0047	0.0105
rs6534338	T	0.0103	0.0015	-0.0153	0.0105
rs6543810	T	0.0082	0.0015	0.0137	0.0095
rs6557171	T	-0.0155	0.0015	-0.0031	0.0096
rs6567288	A	-0.0079	0.0014	0.0047	0.0093
rs6569077	T	0.0118	0.0014	-0.0033	0.0094
rs6573552	T	-0.0090	0.0014	0.0031	0.0092
rs6573559	T	0.0105	0.0015	0.0105	0.0100
rs660001	A	-0.0164	0.0017	0.0210	0.0114
rs663251	T	0.0081	0.0015	0.0064	0.0095
rs66482320	C	0.0124	0.0022	0.0104	0.0149
rs66568921	T	-0.0181	0.0015	0.0078	0.0097
rs66671632	T	-0.0133	0.0021	0.0084	0.0133
rs66721975	A	-0.0087	0.0015	0.0008	0.0107
rs6672986	C	0.0094	0.0014	0.0020	0.0092
rs6689641	A	0.0078	0.0014	-0.0002	0.0094
rs6690195	T	-0.0113	0.0014	0.0110	0.0098
rs6695132	T	0.0097	0.0017	-0.0148	0.0105
rs6696068	T	-0.0088	0.0014	0.0036	0.0098

rs6697584	T	-0.0126	0.0017	0.0258	0.0121
rs6704768	A	-0.0116	0.0014	0.0237	0.0096
rs6706275	T	0.0083	0.0015	-0.0075	0.0108
rs6711399	T	-0.0107	0.0019	0.0175	0.0114
rs6715321	T	-0.0097	0.0014	0.0099	0.0099
rs6715849	A	-0.0122	0.0014	0.0021	0.0097
rs6720515	A	0.0103	0.0017	-0.0042	0.0111
rs67224963	A	0.0113	0.0017	-0.0037	0.0113
rs6729612	T	0.0092	0.0015	0.0056	0.0101
rs6731373	A	-0.0115	0.0015	-0.0103	0.0113
rs6736025	T	0.0079	0.0014	-0.0164	0.0095
rs6738860	A	-0.0088	0.0014	0.0040	0.0096
rs6743032	A	-0.0165	0.0024	-0.0253	0.0180
rs6744040	A	-0.0080	0.0014	0.0074	0.0093
rs67456868	A	0.0119	0.0018	-0.0031	0.0132
rs6752228	T	-0.0080	0.0014	-0.0016	0.0094
rs6757087	T	-0.0085	0.0014	0.0024	0.0100
rs6760887	T	0.0155	0.0022	-0.0152	0.0119
rs67661275	A	0.0134	0.0025	-0.0104	0.0177
rs6774533	T	0.0090	0.0015	0.0059	0.0099
rs67790232	A	0.0101	0.0017	0.0036	0.0126
rs6780414	T	0.0105	0.0018	0.0058	0.0119
rs6792805	A	-0.0090	0.0016	-0.0034	0.0101
rs6812533	T	0.0106	0.0016	0.0107	0.0103
rs68145588	T	-0.0127	0.0021	0.0021	0.0148
rs6824567	T	0.0130	0.0016	-0.0049	0.0111
rs6853599	T	0.0108	0.0019	-0.0116	0.0140
rs6861925	C	-0.0090	0.0014	0.0049	0.0092
rs6871635	A	-0.0087	0.0014	-0.0151	0.0099
rs6881581	A	-0.0096	0.0015	0.0301	0.0101
rs6881733	T	-0.0086	0.0016	0.0023	0.0104
rs6917154	T	0.0127	0.0021	0.0033	0.0159
rs6917204	T	0.0119	0.0017	-0.0270	0.0105
rs6918506	C	0.0113	0.0014	0.0132	0.0093
rs6924023	A	0.0107	0.0016	-0.0012	0.0106
rs6932108	C	-0.0210	0.0027	0.0182	0.0195
rs6946136	A	0.0081	0.0014	0.0001	0.0097
rs6946362	T	0.0091	0.0015	-0.0193	0.0109
rs6951996	A	-0.0182	0.0032	0.0352	0.0205
rs6956283	T	0.0098	0.0016	-0.0102	0.0105
rs6969783	A	-0.0098	0.0014	0.0209	0.0093
rs6977237	T	0.0082	0.0014	-0.0061	0.0094
rs6989141	A	0.0100	0.0017	-0.0077	0.0131
rs6994287	A	0.0102	0.0014	-0.0039	0.0096
rs7012546	T	0.0092	0.0014	-0.0101	0.0095
rs7016302	C	-0.0125	0.0019	0.0080	0.0130

rs702606	T	0.0118	0.0021	-0.0001	0.0134
rs7029718	A	0.0244	0.0014	-0.0030	0.0095
rs7030373	A	-0.0098	0.0017	0.0092	0.0107
rs7035315	A	-0.0090	0.0015	-0.0177	0.0096
rs7040995	C	0.0097	0.0014	-0.0240	0.0093
rs7041702	A	-0.0111	0.0016	0.0149	0.0110
rs7084508	T	-0.0085	0.0015	-0.0067	0.0103
rs710629	A	0.0082	0.0015	-0.0081	0.0096
rs7108020	A	0.0111	0.0015	-0.0089	0.0101
rs711793	T	0.0103	0.0015	-0.0046	0.0117
rs7127580	C	-0.0150	0.0023	0.0042	0.0170
rs713584	A	-0.0080	0.0014	0.0011	0.0093
rs71413877	A	0.0351	0.0036	-0.0326	0.0307
rs7146625	A	0.0091	0.0016	-0.0215	0.0109
rs7147473	A	-0.0103	0.0015	0.0161	0.0106
rs7158218	A	-0.0090	0.0015	-0.0015	0.0106
rs71646142	T	0.0104	0.0018	-0.0003	0.0124
rs716513	A	0.0084	0.0014	-0.0092	0.0094
rs7167688	T	-0.0094	0.0014	0.0023	0.0093
rs7171405	A	-0.0101	0.0016	0.0135	0.0107
rs717996	T	-0.0122	0.0014	0.0217	0.0099
rs7182216	C	-0.0096	0.0017	-0.0125	0.0122
rs7190	A	-0.0095	0.0015	-0.0009	0.0110
rs721579	T	-0.0110	0.0016	0.0102	0.0104
rs7218235	A	-0.0097	0.0017	0.0198	0.0124
rs7223311	C	-0.0079	0.0014	0.0109	0.0100
rs7226824	T	-0.0078	0.0014	-0.0031	0.0093
rs72482130	T	0.0141	0.0019	-0.0249	0.0128
rs72486027	T	-0.0104	0.0016	0.0021	0.0105
rs7254263	T	-0.0119	0.0016	0.0052	0.0102
rs7255223	A	0.0097	0.0016	-0.0110	0.0103
rs72622559	T	-0.0120	0.0017	0.0086	0.0110
rs72624911	T	0.0282	0.0034	-0.0947	0.0277
rs72636697	T	-0.0140	0.0020	0.0175	0.0129
rs72667460	T	0.0197	0.0031	-0.0122	0.0215
rs72671456	A	-0.0174	0.0020	0.0294	0.0147
rs72672052	A	-0.0101	0.0018	0.0198	0.0120
rs72673097	A	0.0257	0.0042	0.0444	0.0410
rs72677177	A	0.0100	0.0014	-0.0009	0.0098
rs72694479	T	0.0127	0.0020	0.0005	0.0142
rs72709560	A	0.0085	0.0015	-0.0143	0.0112
rs72771860	T	-0.0211	0.0029	-0.0217	0.0253
rs72792395	T	-0.0145	0.0022	0.0051	0.0146
rs728054	A	-0.0128	0.0015	0.0109	0.0096
rs72819118	T	-0.0231	0.0024	-0.0043	0.0195
rs72824753	T	0.0133	0.0024	-0.0158	0.0152

rs72828517	T	-0.0168	0.0019	0.0066	0.0127
rs72829857	A	-0.0149	0.0016	0.0162	0.0109
rs72834698	A	0.0117	0.0020	-0.0140	0.0160
rs72881110	A	0.0146	0.0027	-0.0056	0.0176
rs72883760	A	0.0101	0.0018	-0.0363	0.0121
rs72896637	T	0.0162	0.0028	-0.0202	0.0245
rs72902523	T	-0.0103	0.0016	-0.0192	0.0104
rs72906124	T	0.0185	0.0030	-0.0254	0.0227
rs72917504	T	-0.0178	0.0030	-0.0167	0.0217
rs72919450	T	-0.0132	0.0023	0.0009	0.0156
rs72944064	T	-0.0098	0.0016	-0.0132	0.0115
rs72962169	T	-0.0174	0.0019	-0.0069	0.0125
rs72972965	A	0.0083	0.0015	0.0037	0.0106
rs72993796	T	0.0127	0.0022	0.0106	0.0181
rs73034295	A	0.0099	0.0018	-0.0151	0.0129
rs730384	A	0.0101	0.0014	-0.0108	0.0096
rs73039077	C	0.0092	0.0016	-0.0097	0.0119
rs73055556	A	0.0111	0.0020	-0.0058	0.0134
rs73082325	C	-0.0252	0.0044	0.0696	0.0393
rs73106136	T	-0.0147	0.0026	-0.0057	0.0192
rs73154546	A	0.0197	0.0036	-0.0070	0.0291
rs7317761	C	0.0109	0.0016	0.0071	0.0100
rs73191311	A	-0.0094	0.0015	-0.0093	0.0103
rs7321274	A	0.0114	0.0018	-0.0181	0.0115
rs73219806	A	0.0106	0.0019	-0.0096	0.0133
rs7323027	A	0.0130	0.0015	-0.0081	0.0096
rs7326331	A	-0.0128	0.0016	0.0060	0.0103
rs73344830	A	0.0170	0.0014	-0.0020	0.0098
rs73405293	A	0.0113	0.0020	-0.0022	0.0137
rs73457936	A	0.0144	0.0025	0.0203	0.0194
rs73518807	C	-0.0128	0.0021	0.0179	0.0109
rs7356536	T	-0.0129	0.0021	0.0021	0.0143
rs736281	T	0.0086	0.0014	0.0161	0.0098
rs73643713	T	-0.0165	0.0027	0.0046	0.0177
rs736471	T	0.0083	0.0014	0.0049	0.0101
rs73648455	T	-0.0183	0.0026	0.0148	0.0167
rs737945	C	-0.0115	0.0014	0.0044	0.0094
rs73874335	T	-0.0178	0.0029	-0.0046	0.0170
rs73961845	A	-0.0114	0.0014	0.0035	0.0102
rs74091672	A	-0.0153	0.0022	0.0101	0.0159
rs7430651	T	-0.0111	0.0016	0.0088	0.0101
rs743316	T	0.0112	0.0017	0.0071	0.0119
rs74415461	T	0.0176	0.0026	-0.0227	0.0211
rs74453875	A	0.0214	0.0037	-0.0112	0.0219
rs74462621	T	0.0125	0.0020	0.0129	0.0146
rs7449561	A	0.0102	0.0017	0.0192	0.0110

rs7451726	A	0.0109	0.0017	0.0070	0.0112
rs74545339	A	-0.0158	0.0022	-0.0141	0.0149
rs7460106	T	-0.0123	0.0017	0.0137	0.0120
rs74615093	A	0.0145	0.0026	-0.0002	0.0190
rs746839	C	0.0135	0.0015	0.0021	0.0103
rs74747621	T	0.0153	0.0022	-0.0274	0.0159
rs74787922	A	0.0182	0.0028	-0.0133	0.0208
rs74944275	T	0.0231	0.0035	-0.0363	0.0287
rs74944857	T	-0.0089	0.0015	0.0078	0.0105
rs7495033	A	0.0095	0.0015	-0.0116	0.0100
rs75033012	C	-0.0258	0.0038	-0.0127	0.0343
rs75177132	T	0.0298	0.0036	-0.0102	0.0386
rs75203411	T	0.0135	0.0019	-0.0070	0.0132
rs7526112	T	0.0124	0.0015	0.0036	0.0096
rs75308819	A	0.0287	0.0047	0.0006	0.0375
rs75434274	A	0.0151	0.0026	-0.0382	0.0201
rs75500877	T	0.0207	0.0033	-0.0145	0.0255
rs7552964	A	0.0148	0.0023	-0.0223	0.0158
rs7560871	A	-0.0193	0.0027	0.0067	0.0198
rs7561705	A	0.0078	0.0014	-0.0076	0.0098
rs7575637	A	0.0107	0.0014	0.0301	0.0096
rs75756843	A	0.0288	0.0049	-0.0137	0.0353
rs7575938	A	0.0090	0.0015	-0.0033	0.0099
rs7590368	T	-0.0146	0.0016	-0.0031	0.0108
rs7597126	T	-0.0094	0.0014	-0.0010	0.0101
rs7597412	C	0.0088	0.0015	0.0093	0.0103
rs7603132	A	0.0137	0.0018	-0.0240	0.0116
rs76076331	T	0.0206	0.0021	-0.0116	0.0144
rs76077165	A	0.0165	0.0025	-0.0169	0.0186
rs76167224	T	-0.0187	0.0033	-0.0182	0.0310
rs7617204	A	-0.0097	0.0014	-0.0058	0.0093
rs76235882	A	0.0241	0.0039	-0.0220	0.0278
rs76241605	A	0.0139	0.0022	0.0092	0.0186
rs76246107	A	-0.0162	0.0026	-0.0253	0.0227
rs7625428	T	0.0098	0.0014	-0.0008	0.0096
rs76267866	A	0.0104	0.0017	-0.0124	0.0118
rs7630133	A	0.0082	0.0015	-0.0118	0.0094
rs763553	A	0.0087	0.0014	-0.0026	0.0093
rs7640424	T	0.0113	0.0015	0.0023	0.0108
rs7650602	T	-0.0089	0.0014	0.0224	0.0097
rs76552497	T	-0.0120	0.0019	0.0192	0.0128
rs76577427	C	0.0203	0.0024	-0.0196	0.0201
rs7672622	A	-0.0088	0.0016	0.0111	0.0102
rs767943	A	-0.0122	0.0016	0.0224	0.0102
rs7683416	T	0.0139	0.0014	-0.0046	0.0094
rs76876592	A	0.0126	0.0020	0.0062	0.0138

rs76878669	C	0.0141	0.0017	-0.0052	0.0112
rs7692359	T	-0.0109	0.0017	0.0264	0.0117
rs76957677	T	-0.0297	0.0054	0.0441	0.0619
rs77025239	A	-0.0140	0.0019	-0.0016	0.0127
rs7714719	C	0.0116	0.0020	0.0314	0.0133
rs7716161	C	0.0105	0.0019	0.0237	0.0128
rs77201694	A	0.0156	0.0022	-0.0284	0.0188
rs77370942	A	-0.0147	0.0027	-0.0055	0.0216
rs77554090	T	-0.0186	0.0027	0.0396	0.0209
rs77609760	A	0.0236	0.0028	-0.0159	0.0218
rs7766240	T	-0.0134	0.0024	0.0205	0.0168
rs77702622	A	-0.0213	0.0029	0.0268	0.0230
rs77702819	T	0.0187	0.0025	-0.0072	0.0198
rs7772172	A	0.0091	0.0014	-0.0081	0.0096
rs7775100	T	-0.0087	0.0014	-0.0210	0.0096
rs77826402	T	0.0151	0.0027	0.0042	0.0180
rs7796103	C	-0.0077	0.0014	0.0058	0.0092
rs7799141	A	0.0099	0.0015	-0.0139	0.0098
rs77999825	A	0.0294	0.0039	-0.0297	0.0332
rs7803932	A	0.0127	0.0019	-0.0150	0.0135
rs780569	A	-0.0085	0.0016	0.0061	0.0104
rs78116078	C	0.0086	0.0016	0.0049	0.0111
rs7816777	T	-0.0088	0.0015	-0.0031	0.0100
rs78193153	A	0.0191	0.0029	-0.0338	0.0164
rs7823700	T	0.0132	0.0024	-0.0152	0.0180
rs78365243	T	0.0181	0.0033	0.0190	0.0268
rs78440611	A	-0.0147	0.0024	0.0228	0.0156
rs7849480	A	-0.0084	0.0014	0.0180	0.0094
rs7849487	T	-0.0167	0.0015	-0.0149	0.0098
rs7855503	C	0.0088	0.0015	0.0048	0.0098
rs78648104	T	-0.0141	0.0025	0.0118	0.0168
rs78702390	C	0.0167	0.0030	-0.0148	0.0226
rs78714229	T	0.0172	0.0030	0.0320	0.0217
rs7875078	A	-0.0078	0.0014	0.0082	0.0095
rs78918150	T	-0.0080	0.0015	0.0048	0.0099
rs7894722	T	0.0085	0.0014	0.0007	0.0099
rs7899270	A	-0.0084	0.0015	0.0078	0.0101
rs7905192	T	0.0084	0.0014	0.0284	0.0093
rs790647	A	-0.0145	0.0017	-0.0077	0.0107
rs7910403	T	0.0122	0.0018	-0.0048	0.0128
rs7914674	T	-0.0139	0.0022	0.0041	0.0119
rs7920624	A	0.0120	0.0014	-0.0094	0.0092
rs7924036	T	0.0133	0.0014	0.0061	0.0092
rs79265434	A	-0.0197	0.0022	-0.0005	0.0162
rs7928017	A	0.0096	0.0014	-0.0081	0.0095
rs7931563	T	-0.0109	0.0014	0.0028	0.0096

rs795230	T	0.0081	0.0014	-0.0045	0.0094
rs7958371	A	-0.0085	0.0015	0.0145	0.0096
rs79585412	T	-0.0178	0.0031	-0.0166	0.0214
rs7965154	A	-0.0085	0.0015	-0.0005	0.0099
rs7967550	A	-0.0086	0.0014	0.0068	0.0094
rs7972246	T	0.0110	0.0015	-0.0179	0.0097
rs79728014	A	-0.0134	0.0020	0.0073	0.0145
rs7974852	A	0.0117	0.0014	-0.0130	0.0093
rs7977614	A	-0.0134	0.0016	0.0310	0.0113
rs79798166	A	0.0172	0.0025	0.0039	0.0199
rs79855925	T	-0.0194	0.0033	-0.0026	0.0262
rs79994730	T	0.0240	0.0035	0.0198	0.0283
rs79997166	A	0.0172	0.0030	-0.0110	0.0254
rs80037907	T	-0.0107	0.0019	-0.0010	0.0138
rs8008382	T	-0.0106	0.0015	0.0000	0.0099
rs8009933	A	0.0088	0.0015	0.0092	0.0097
rs8016504	A	-0.0087	0.0014	0.0051	0.0097
rs80223410	T	-0.0118	0.0020	0.0172	0.0179
rs8024	A	-0.0115	0.0015	0.0416	0.0101
rs80257979	T	-0.0252	0.0040	-0.0033	0.0285
rs8030487	A	0.0085	0.0015	-0.0276	0.0104
rs803619	T	-0.0173	0.0023	0.0223	0.0153
rs8046072	A	0.0101	0.0018	-0.0054	0.0116
rs8052523	T	-0.0084	0.0014	-0.0032	0.0101
rs8066044	A	0.0090	0.0016	-0.0187	0.0105
rs806816	A	0.0101	0.0016	-0.0034	0.0113
rs8097125	T	0.0086	0.0014	-0.0015	0.0099
rs8103741	A	-0.0108	0.0019	0.0075	0.0133
rs818415	T	-0.0110	0.0018	0.0032	0.0112
rs8192465	A	-0.0272	0.0049	-0.0044	0.0428
rs852771	T	0.0092	0.0015	0.0074	0.0104
rs853286	T	0.0124	0.0023	-0.0049	0.0131
rs854796	A	0.0090	0.0015	0.0043	0.0100
rs868456	A	0.0089	0.0015	0.0044	0.0104
rs870589	A	-0.0079	0.0014	0.0156	0.0093
rs884108	A	-0.0094	0.0017	-0.0138	0.0117
rs891793	C	-0.0099	0.0014	-0.0071	0.0094
rs893522	A	0.0149	0.0025	-0.0183	0.0168
rs902820	A	-0.0096	0.0014	-0.0078	0.0096
rs911149	T	0.0091	0.0017	-0.0094	0.0109
rs912883	T	0.0087	0.0015	-0.0037	0.0098
rs913509	A	0.0096	0.0015	-0.0077	0.0098
rs925161	C	-0.0078	0.0014	-0.0033	0.0094
rs9267658	T	0.0138	0.0020	-0.0496	0.0170
rs9267677	T	0.0180	0.0024	0.0231	0.0154
rs9289300	T	-0.0164	0.0019	0.0247	0.0129

rs9291437	C	-0.0081	0.0014	0.0000	0.0094
rs9294770	T	0.0083	0.0014	-0.0117	0.0095
rs929511	T	-0.0160	0.0021	0.0046	0.0138
rs9300612	T	0.0084	0.0015	0.0066	0.0097
rs933738	A	-0.0110	0.0018	0.0132	0.0122
rs9349956	A	-0.0159	0.0018	-0.0089	0.0126
rs9359939	A	-0.0105	0.0016	-0.0118	0.0105
rs936496	A	-0.0079	0.0014	0.0015	0.0096
rs9371883	C	-0.0087	0.0015	0.0124	0.0098
rs9373363	A	-0.0111	0.0016	-0.0097	0.0107
rs9375188	T	0.0212	0.0014	-0.0012	0.0094
rs9375403	C	0.0126	0.0016	-0.0134	0.0113
rs9386110	T	-0.0163	0.0023	0.0106	0.0152
rs9388490	T	0.0091	0.0014	-0.0356	0.0097
rs939400	T	-0.0095	0.0015	0.0047	0.0098
rs9411331	A	0.0145	0.0015	-0.0148	0.0097
rs9435340	A	0.0086	0.0015	-0.0111	0.0097
rs9442750	A	-0.0089	0.0016	-0.0017	0.0111
rs9446060	A	0.0077	0.0014	-0.0004	0.0094
rs9465509	A	-0.0084	0.0014	0.0109	0.0092
rs9490512	A	-0.0136	0.0014	0.0008	0.0094
rs9492774	C	0.0080	0.0015	-0.0240	0.0094
rs9513416	A	-0.0116	0.0019	0.0026	0.0137
rs9513754	T	0.0096	0.0016	0.0062	0.0111
rs9527662	A	0.0094	0.0014	0.0083	0.0101
rs9527905	A	-0.0094	0.0014	-0.0121	0.0093
rs9529146	T	0.0127	0.0017	-0.0037	0.0105
rs9536462	A	0.0133	0.0020	-0.0102	0.0130
rs9540718	A	-0.0089	0.0014	0.0191	0.0093
rs9545395	T	0.0138	0.0021	0.0037	0.0130
rs9556958	T	-0.0110	0.0014	-0.0053	0.0094
rs9563168	A	0.0101	0.0017	-0.0100	0.0116
rs9568798	T	-0.0093	0.0016	0.0157	0.0103
rs9597907	T	0.0152	0.0027	0.0065	0.0163
rs9611597	A	-0.0107	0.0019	0.0147	0.0151
rs9616906	A	0.0122	0.0014	-0.0136	0.0098
rs9616947	T	-0.0134	0.0015	0.0233	0.0106
rs9633970	T	0.0091	0.0016	-0.0037	0.0109
rs9649	T	0.0108	0.0019	-0.0150	0.0122
rs9655780	A	-0.0153	0.0019	-0.0204	0.0139
rs9666728	T	-0.0082	0.0014	-0.0051	0.0094
rs9683585	C	0.0084	0.0014	0.0019	0.0092
rs969512	A	-0.0106	0.0015	0.0054	0.0095
rs9771228	T	0.0096	0.0015	-0.0181	0.0095
rs977143	A	0.0085	0.0015	0.0024	0.0101
rs978807	A	0.0139	0.0018	-0.0074	0.0116

rs981230	T	0.0100	0.0014	0.0109	0.0094
rs981883	A	0.0099	0.0014	-0.0162	0.0095
rs9820604	T	0.0115	0.0018	-0.0055	0.0116
rs9821664	A	0.0094	0.0017	-0.0100	0.0105
rs9830359	T	-0.0147	0.0022	0.0091	0.0145
rs9844755	A	0.0083	0.0015	0.0075	0.0099
rs9849884	A	-0.0090	0.0014	0.0076	0.0098
rs9853928	T	-0.0127	0.0017	0.0220	0.0105
rs9858921	A	-0.0087	0.0014	0.0031	0.0093
rs9859556	T	0.0290	0.0015	-0.0431	0.0103
rs9866123	A	0.0086	0.0014	-0.0128	0.0096
rs9870317	A	0.0086	0.0015	-0.0290	0.0097
rs9877225	T	0.0090	0.0016	-0.0080	0.0106
rs9882532	T	0.0124	0.0015	0.0021	0.0100
rs9886703	A	-0.0136	0.0019	0.0333	0.0121
rs9888796	T	0.0113	0.0016	-0.0066	0.0107
rs9916901	T	0.0092	0.0016	-0.0218	0.0100
rs9924031	C	0.0093	0.0015	0.0066	0.0104
rs9926649	T	0.0177	0.0031	-0.0121	0.0223
rs9927049	A	0.0099	0.0016	0.0127	0.0117
rs9927137	A	0.0082	0.0014	0.0003	0.0104
rs9927842	T	-0.0130	0.0020	0.0031	0.0127
rs9929556	T	-0.0099	0.0014	0.0123	0.0095
rs9929762	A	0.0096	0.0014	-0.0007	0.0093
rs9929993	T	-0.0102	0.0015	0.0011	0.0097
rs9933256	A	0.0119	0.0014	0.0077	0.0110
rs995698	A	-0.0099	0.0014	0.0001	0.0096
rs9974899	T	-0.0093	0.0016	0.0022	0.0107
rs9977825	T	-0.0092	0.0015	0.0260	0.0104

Supplementary Table 9: Genetic association estimates for the IVW ratio-method MR analysis estimating the effect of education on MI. ea=effect allele; gx=education; gy=MI; se=standard error

SNP	ea	gx	gx_se	gy	gy_se
rs10006235	T	-0.0093	0.0016	-0.0077	0.0112
rs10009513	A	0.0088	0.0014	-0.0174	0.0103
rs10021733	T	-0.0164	0.0027	-0.0023	0.0154
rs10032941	T	-0.0080	0.0015	0.0127	0.0106
rs10041403	T	0.0151	0.0019	-0.0142	0.0142
rs10042828	A	-0.0145	0.0022	0.0007	0.0172
rs10046069	A	0.0127	0.0022	0.0075	0.0154
rs10057590	A	0.0106	0.0014	-0.0049	0.0106
rs10060023	T	0.0110	0.0015	0.0019	0.0116
rs10061420	A	0.0082	0.0014	-0.0027	0.0104
rs1006749	A	0.0079	0.0014	-0.0013	0.0108
rs10074178	A	0.0079	0.0014	-0.0025	0.0104
rs1007731	A	-0.0143	0.0022	0.0031	0.0192
rs10080647	A	0.0124	0.0020	-0.0220	0.0141
rs1008078	T	-0.0182	0.0014	0.0058	0.0106
rs1009470	T	0.0086	0.0014	-0.0198	0.0103
rs10098073	A	-0.0091	0.0014	-0.0033	0.0109
rs10122669	A	-0.0079	0.0014	0.0039	0.0104
rs10128888	A	-0.0083	0.0015	0.0334	0.0119
rs10145520	T	-0.0122	0.0018	-0.0182	0.0133
rs10166286	T	0.0151	0.0019	-0.0035	0.0125
rs10169002	A	-0.0080	0.0014	0.0044	0.0104
rs10181071	A	0.0102	0.0019	-0.0008	0.0144
rs10189857	A	0.0158	0.0014	-0.0086	0.0105
rs10192369	A	-0.0078	0.0014	-0.0010	0.0106
rs10192834	T	0.0083	0.0015	-0.0087	0.0111
rs10193498	A	0.0097	0.0016	0.0107	0.0116
rs10204051	T	-0.0101	0.0014	0.0000	0.0111
rs10205057	T	0.0090	0.0014	-0.0191	0.0102
rs10208	T	0.0091	0.0015	-0.0124	0.0111
rs10215082	A	-0.0149	0.0014	0.0030	0.0109
rs1024268	T	-0.0084	0.0014	0.0025	0.0103
rs10251438	A	0.0078	0.0014	-0.0195	0.0103
rs10264573	A	0.0175	0.0032	0.0262	0.0238
rs1035578	A	-0.0094	0.0014	-0.0111	0.0106
rs10402747	T	0.0077	0.0014	-0.0253	0.0108
rs10411759	A	0.0116	0.0020	-0.0069	0.0153
rs10444280	T	0.0100	0.0018	0.0044	0.0134
rs10460095	A	-0.0124	0.0014	0.0057	0.0105
rs10480450	A	0.0158	0.0028	0.0190	0.0197
rs10496091	A	-0.0131	0.0016	0.0213	0.0124
rs10499535	A	0.0080	0.0014	0.0059	0.0104
rs1050847	T	0.0092	0.0014	-0.0304	0.0116

rs10509251	T	-0.0093	0.0017	0.0227	0.0116
rs10515007	T	0.0143	0.0019	-0.0287	0.0134
rs1051860	A	-0.0079	0.0014	0.0240	0.0108
rs10519504	T	0.0119	0.0019	-0.0104	0.0141
rs1054442	A	-0.0136	0.0015	-0.0026	0.0109
rs1061801	A	-0.0110	0.0018	0.0112	0.0137
rs1066769	A	-0.0230	0.0041	-0.0222	0.0319
rs10742591	A	-0.0084	0.0014	0.0022	0.0105
rs10748841	A	-0.0111	0.0020	0.0084	0.0110
rs10750539	A	-0.0095	0.0015	0.0025	0.0109
rs10752262	T	0.0102	0.0014	-0.0032	0.0108
rs1075228	A	-0.0081	0.0014	-0.0029	0.0112
rs10761202	T	0.0088	0.0014	0.0211	0.0104
rs10761251	A	0.0113	0.0015	0.0111	0.0107
rs10772644	C	0.0142	0.0022	-0.0278	0.0157
rs10773002	A	0.0185	0.0016	-0.0135	0.0117
rs10773208	T	-0.0110	0.0016	-0.0124	0.0121
rs10782651	T	-0.0082	0.0014	0.0037	0.0104
rs10783243	A	-0.0092	0.0014	0.0076	0.0105
rs10789285	T	0.0091	0.0016	-0.0160	0.0116
rs10793903	A	0.0126	0.0022	-0.0248	0.0159
rs10795831	T	-0.0099	0.0017	0.0152	0.0118
rs10798888	T	-0.0140	0.0019	0.0197	0.0129
rs10799615	A	-0.0089	0.0016	0.0225	0.0112
rs10805383	A	-0.0103	0.0014	0.0088	0.0103
rs10810099	A	-0.0146	0.0016	0.0015	0.0112
rs10810145	A	0.0078	0.0014	-0.0123	0.0104
rs10830858	T	0.0092	0.0014	-0.0100	0.0103
rs10831656	T	0.0090	0.0015	-0.0006	0.0114
rs10844179	A	0.0104	0.0017	-0.0208	0.0124
rs10845051	A	0.0083	0.0014	0.0010	0.0107
rs10875121	C	0.0174	0.0019	-0.0192	0.0142
rs10877283	T	0.0085	0.0014	0.0014	0.0109
rs10879676	T	-0.0083	0.0014	0.0064	0.0106
rs10886012	C	0.0078	0.0014	-0.0027	0.0103
rs10892807	T	0.0109	0.0014	0.0098	0.0110
rs10899282	A	0.0140	0.0017	-0.0165	0.0128
rs10927053	A	-0.0125	0.0022	0.0159	0.0152
rs10928190	T	-0.0087	0.0014	0.0058	0.0104
rs10931821	A	-0.0141	0.0014	0.0109	0.0103
rs10937240	T	-0.0123	0.0018	-0.0028	0.0122
rs10940540	A	0.0078	0.0014	0.0149	0.0106
rs10940921	T	0.0111	0.0014	0.0105	0.0102
rs10943588	A	0.0084	0.0015	-0.0196	0.0106
rs10947439	T	0.0090	0.0015	-0.0173	0.0111
rs10949263	A	-0.0091	0.0016	-0.0131	0.0115

rs10951590	T	-0.0109	0.0015	0.0069	0.0110
rs10974256	A	-0.0093	0.0015	-0.0030	0.0119
rs1097784	T	-0.0083	0.0014	-0.0012	0.0105
rs10979613	T	-0.0117	0.0015	-0.0031	0.0108
rs10983324	A	0.0084	0.0015	-0.0259	0.0112
rs10984445	A	0.0115	0.0014	-0.0217	0.0103
rs10985402	T	0.0089	0.0016	-0.0058	0.0115
rs10995639	T	0.0078	0.0014	-0.0117	0.0103
rs10996167	C	0.0113	0.0020	0.0085	0.0108
rs11003463	T	0.0091	0.0015	-0.0086	0.0123
rs11021432	A	0.0103	0.0015	0.0086	0.0113
rs11023764	A	0.0091	0.0015	0.0148	0.0112
rs11030084	T	0.0106	0.0018	-0.0276	0.0128
rs11030102	C	0.0101	0.0016	-0.0239	0.0131
rs1106090	A	0.0094	0.0014	-0.0005	0.0103
rs11076962	T	0.0086	0.0016	0.0006	0.0113
rs1107871	A	-0.0083	0.0014	0.0158	0.0103
rs11081529	T	0.0126	0.0015	-0.0199	0.0116
rs11082011	T	0.0194	0.0015	0.0141	0.0107
rs11100237	A	-0.0101	0.0014	0.0028	0.0102
rs11115056	A	0.0187	0.0023	-0.0012	0.0203
rs11121177	A	0.0151	0.0018	-0.0106	0.0129
rs111226181	T	-0.0201	0.0018	0.0140	0.0147
rs111235962	T	0.0133	0.0024	-0.0034	0.0182
rs11130380	T	0.0090	0.0014	0.0181	0.0109
rs111370527	T	0.0151	0.0027	0.0077	0.0275
rs11138947	T	0.0105	0.0016	-0.0148	0.0110
rs111530150	T	0.0269	0.0048	0.0620	0.0354
rs11155813	T	-0.0129	0.0023	-0.0401	0.0151
rs11157931	A	-0.0139	0.0014	-0.0191	0.0109
rs11158800	A	-0.0091	0.0014	0.0117	0.0108
rs1117152	T	0.0106	0.0015	-0.0225	0.0111
rs11174399	A	0.0111	0.0018	-0.0123	0.0129
rs111821073	T	0.0124	0.0019	-0.0162	0.0152
rs111852224	T	0.0156	0.0022	0.0197	0.0194
rs11190955	T	-0.0083	0.0015	0.0044	0.0111
rs11191237	T	0.0132	0.0024	-0.0154	0.0123
rs1120924	T	0.0108	0.0015	-0.0029	0.0113
rs11209894	A	0.0119	0.0021	-0.0125	0.0144
rs11210228	T	-0.0105	0.0019	0.0070	0.0108
rs11210400	A	0.0120	0.0014	0.0299	0.0106
rs11211123	A	0.0104	0.0017	0.0081	0.0121
rs11213482	A	-0.0106	0.0019	-0.0006	0.0161
rs112210983	A	0.0105	0.0017	-0.0005	0.0131
rs1123285	C	-0.0094	0.0015	-0.0100	0.0112
rs112375785	A	0.0184	0.0021	-0.0098	0.0194

rs112379405	C	-0.0082	0.0014	-0.0143	0.0105
rs112512729	T	-0.0166	0.0027	-0.0019	0.0230
rs112603734	A	-0.0131	0.0022	-0.0021	0.0128
rs11265191	T	0.0083	0.0015	0.0078	0.0107
rs112806496	C	-0.0160	0.0025	0.0160	0.0205
rs1128956	T	-0.0140	0.0019	0.0265	0.0149
rs113205706	A	-0.0135	0.0024	-0.0305	0.0203
rs113520408	A	0.0155	0.0016	-0.0102	0.0132
rs113552169	C	0.0294	0.0053	-0.0360	0.0415
rs113588399	T	-0.0104	0.0017	0.0019	0.0137
rs113615161	T	-0.0123	0.0021	0.0300	0.0149
rs113720505	A	0.0164	0.0029	-0.0029	0.0292
rs113731629	T	0.0339	0.0061	-0.0889	0.0544
rs114192810	T	-0.0279	0.0051	0.0809	0.0387
rs114468556	A	0.0293	0.0036	-0.0472	0.0344
rs114593137	A	-0.0122	0.0017	0.0131	0.0133
rs1146079	C	-0.0087	0.0016	0.0129	0.0121
rs114810763	A	-0.0121	0.0020	0.0027	0.0175
rs114952970	T	0.0265	0.0040	0.0274	0.0336
rs114958262	T	-0.0220	0.0039	0.0180	0.0375
rs115000530	A	-0.0250	0.0031	0.0557	0.0259
rs115017135	A	-0.0206	0.0038	0.0112	0.0348
rs11542663	A	0.0112	0.0015	0.0000	0.0109
rs115438240	T	-0.0169	0.0030	0.0126	0.0261
rs115693355	A	0.0309	0.0053	0.0185	0.0484
rs115732722	A	-0.0415	0.0072	-0.0209	0.0590
rs11588857	A	0.0199	0.0017	0.0202	0.0122
rs11591870	T	0.0098	0.0017	-0.0161	0.0140
rs11592299	A	0.0097	0.0018	0.0180	0.0141
rs11596387	T	0.0258	0.0046	0.0192	0.0456
rs11598765	A	-0.0106	0.0018	0.0029	0.0123
rs11599236	T	-0.0106	0.0014	0.0171	0.0105
rs11613431	A	-0.0094	0.0016	-0.0062	0.0126
rs11620355	A	0.0182	0.0025	-0.0312	0.0193
rs11623285	T	-0.0135	0.0021	0.0294	0.0162
rs11640569	A	0.0112	0.0020	-0.0063	0.0160
rs11644446	A	0.0117	0.0019	0.0067	0.0163
rs11647188	A	0.0079	0.0014	-0.0036	0.0110
rs11652522	A	-0.0154	0.0023	0.0086	0.0173
rs11657342	A	0.0140	0.0019	0.0043	0.0147
rs11657979	A	-0.0091	0.0017	0.0301	0.0126
rs11663602	A	-0.0130	0.0016	0.0020	0.0112
rs11663678	T	-0.0121	0.0022	-0.0054	0.0166
rs116656374	T	-0.0242	0.0038	-0.0131	0.0332
rs11675476	T	-0.0127	0.0014	0.0062	0.0102
rs1167827	A	0.0104	0.0014	-0.0050	0.0110

rs11678980	A	-0.0166	0.0014	0.0175	0.0114
rs11687736	A	-0.0105	0.0015	0.0067	0.0110
rs11693885	A	-0.0091	0.0014	0.0154	0.0104
rs116967397	A	0.0315	0.0056	-0.0204	0.0474
rs117005905	T	0.0150	0.0022	-0.0034	0.0156
rs11703948	A	-0.0168	0.0024	0.0054	0.0174
rs1171040	A	-0.0113	0.0017	0.0303	0.0126
rs11716398	A	0.0159	0.0021	0.0042	0.0149
rs11720093	T	0.0150	0.0026	0.0065	0.0197
rs11720985	T	0.0085	0.0015	-0.0094	0.0117
rs11724690	T	0.0093	0.0015	0.0079	0.0119
rs117273411	T	-0.0211	0.0034	0.0376	0.0327
rs11732160	A	0.0105	0.0016	0.0229	0.0117
rs11732657	A	-0.0126	0.0016	0.0039	0.0117
rs11733439	A	-0.0096	0.0017	0.0089	0.0119
rs11736863	A	0.0142	0.0018	-0.0020	0.0135
rs117398064	C	-0.0141	0.0025	0.0003	0.0196
rs117468730	A	-0.0310	0.0050	0.0376	0.0348
rs117623407	A	-0.0118	0.0020	-0.0137	0.0161
rs11765387	T	0.0086	0.0015	0.0065	0.0106
rs117668569	T	0.0234	0.0042	-0.0060	0.0321
rs117677995	A	-0.0180	0.0032	0.0296	0.0329
rs11772232	T	0.0177	0.0019	-0.0407	0.0145
rs11774212	T	0.0128	0.0014	-0.0003	0.0110
rs117799466	C	0.0106	0.0016	0.0017	0.0136
rs11780023	T	0.0085	0.0014	-0.0047	0.0104
rs11789013	T	0.0106	0.0016	-0.0185	0.0118
rs117895796	T	0.0362	0.0064	-0.0286	0.0601
rs118040169	A	-0.0248	0.0038	0.0163	0.0346
rs118093058	T	0.0146	0.0021	-0.0028	0.0181
rs118134876	T	-0.0223	0.0030	0.0424	0.0266
rs11845781	T	-0.0079	0.0014	-0.0032	0.0105
rs11871429	A	0.0128	0.0017	0.0135	0.0119
rs11876620	T	0.0143	0.0024	-0.0077	0.0180
rs11894424	A	0.0209	0.0033	-0.0015	0.0188
rs11917701	A	-0.0087	0.0014	0.0102	0.0105
rs11919835	C	0.0089	0.0015	-0.0233	0.0110
rs1196760	C	0.0139	0.0025	0.0274	0.0212
rs12005151	A	0.0087	0.0015	0.0067	0.0114
rs12028010	T	0.0164	0.0017	-0.0139	0.0127
rs12028229	T	0.0099	0.0016	0.0195	0.0135
rs12054166	C	0.0096	0.0016	-0.0156	0.0121
rs12076635	C	0.0207	0.0017	-0.0195	0.0117
rs12113634	T	0.0088	0.0015	-0.0149	0.0106
rs12123293	T	0.0086	0.0015	0.0029	0.0110
rs12126231	A	0.0095	0.0014	-0.0109	0.0104

rs12127928	T	0.0108	0.0018	-0.0115	0.0140
rs12145078	A	-0.0127	0.0017	0.0143	0.0124
rs12151248	T	-0.0149	0.0023	-0.0240	0.0207
rs12170452	A	0.0105	0.0014	-0.0064	0.0106
rs1220779	A	-0.0079	0.0014	0.0139	0.0105
rs12234369	A	0.0087	0.0015	0.0178	0.0112
rs12238011	T	-0.0141	0.0026	0.0124	0.0174
rs12273435	A	-0.0132	0.0017	0.0193	0.0133
rs12285074	A	0.0161	0.0023	0.0135	0.0142
rs12304188	A	0.0111	0.0019	-0.0063	0.0132
rs12325727	A	0.0088	0.0014	-0.0268	0.0107
rs12332731	A	0.0135	0.0018	-0.0186	0.0143
rs12342546	A	-0.0128	0.0017	-0.0038	0.0123
rs12359372	T	-0.0092	0.0015	0.0218	0.0111
rs12375949	T	-0.0138	0.0014	0.0056	0.0105
rs12405889	T	-0.0098	0.0014	0.0013	0.0102
rs12431682	T	-0.0093	0.0015	-0.0004	0.0108
rs12438177	A	0.0093	0.0015	-0.0208	0.0105
rs12453682	T	0.0102	0.0015	-0.0184	0.0114
rs1245829	A	-0.0099	0.0014	0.0269	0.0112
rs12467175	T	-0.0081	0.0014	0.0281	0.0104
rs12468040	T	0.0137	0.0014	-0.0035	0.0104
rs12473986	T	-0.0085	0.0015	0.0070	0.0110
rs12477385	T	0.0109	0.0017	-0.0158	0.0128
rs12478156	T	0.0108	0.0015	0.0074	0.0107
rs12503522	T	-0.0113	0.0016	0.0175	0.0114
rs12506221	T	-0.0130	0.0014	0.0156	0.0103
rs12516990	C	-0.0099	0.0017	0.0031	0.0135
rs12519073	T	-0.0107	0.0017	-0.0101	0.0123
rs12524795	T	0.0102	0.0014	-0.0070	0.0107
rs12568153	T	-0.0091	0.0015	-0.0028	0.0115
rs12571549	A	0.0151	0.0020	-0.0051	0.0131
rs12574281	A	-0.0080	0.0015	-0.0123	0.0113
rs12591647	T	-0.0152	0.0018	0.0070	0.0125
rs12601380	A	-0.0082	0.0014	0.0011	0.0108
rs12602286	T	0.0160	0.0021	-0.0415	0.0133
rs12613500	C	0.0098	0.0014	-0.0169	0.0104
rs12614263	A	0.0083	0.0014	-0.0190	0.0105
rs12620796	A	0.0121	0.0019	-0.0237	0.0111
rs12638072	A	-0.0107	0.0015	-0.0008	0.0116
rs12643771	T	0.0130	0.0015	-0.0137	0.0117
rs12646216	T	0.0084	0.0014	-0.0087	0.0108
rs12646523	T	-0.0132	0.0016	0.0035	0.0116
rs12659776	T	0.0085	0.0014	-0.0233	0.0102
rs12670376	A	0.0092	0.0014	-0.0154	0.0102
rs1267062	C	0.0113	0.0018	0.0105	0.0137

rs12682775	T	-0.0119	0.0017	0.0024	0.0119
rs12694681	T	0.0102	0.0015	-0.0172	0.0115
rs12709186	A	-0.0091	0.0015	0.0111	0.0114
rs12712269	T	-0.0107	0.0014	0.0057	0.0104
rs12746551	C	0.0248	0.0041	-0.0033	0.0329
rs12750688	C	0.0116	0.0016	0.0293	0.0129
rs12761729	T	-0.0098	0.0016	-0.0106	0.0129
rs12761761	T	0.0153	0.0017	0.0017	0.0131
rs12764593	C	0.0202	0.0029	0.0057	0.0243
rs12765185	A	-0.0088	0.0016	0.0184	0.0125
rs12774577	T	0.0142	0.0020	-0.0218	0.0158
rs12789313	T	0.0092	0.0014	-0.0076	0.0104
rs12790196	T	-0.0101	0.0015	-0.0005	0.0113
rs12810587	T	0.0094	0.0015	-0.0072	0.0112
rs12875339	A	-0.0108	0.0015	-0.0050	0.0104
rs12888615	T	0.0120	0.0018	-0.0059	0.0134
rs12891042	T	0.0092	0.0014	-0.0054	0.0105
rs12891191	C	0.0080	0.0014	-0.0127	0.0112
rs12912465	T	-0.0104	0.0016	0.0385	0.0136
rs12952191	T	-0.0081	0.0014	0.0020	0.0104
rs12953422	A	-0.0080	0.0014	0.0055	0.0107
rs12957463	A	0.0146	0.0018	-0.0061	0.0127
rs12962845	C	-0.0123	0.0021	0.0067	0.0150
rs12967010	T	0.0114	0.0017	-0.0157	0.0136
rs12970264	A	-0.0085	0.0014	0.0049	0.0108
rs12981405	T	-0.0112	0.0019	0.0072	0.0134
rs13009915	T	0.0212	0.0037	-0.0288	0.0370
rs13010288	T	0.0198	0.0021	-0.0164	0.0167
rs13010566	A	-0.0091	0.0014	0.0020	0.0104
rs13015496	C	0.0119	0.0018	-0.0163	0.0124
rs13016316	A	-0.0138	0.0024	-0.0211	0.0155
rs1301838	T	-0.0096	0.0015	0.0291	0.0115
rs13018640	T	-0.0215	0.0014	0.0226	0.0108
rs13026611	A	-0.0084	0.0015	-0.0224	0.0114
rs13029602	T	-0.0092	0.0014	-0.0008	0.0104
rs13034349	T	-0.0082	0.0014	0.0044	0.0113
rs13050131	A	-0.0081	0.0015	-0.0022	0.0109
rs13085461	C	0.0103	0.0014	-0.0080	0.0104
rs13099165	T	-0.0118	0.0017	-0.0165	0.0134
rs13107325	T	-0.0239	0.0027	-0.0015	0.0244
rs13117856	A	0.0084	0.0015	-0.0144	0.0116
rs13130765	C	-0.0091	0.0014	0.0212	0.0116
rs13133213	A	0.0096	0.0014	-0.0301	0.0102
rs13145650	T	-0.0173	0.0025	0.0092	0.0190
rs13154429	C	-0.0136	0.0023	0.0045	0.0167
rs13163062	T	0.0115	0.0014	-0.0130	0.0103

rs13163845	T	-0.0149	0.0020	-0.0024	0.0151
rs13168136	A	-0.0092	0.0017	-0.0032	0.0116
rs13169187	A	-0.0079	0.0014	-0.0055	0.0104
rs13177031	A	-0.0085	0.0015	-0.0114	0.0108
rs13190235	T	-0.0138	0.0024	0.0090	0.0163
rs13197257	T	0.0109	0.0016	-0.0063	0.0118
rs1320139	C	-0.0149	0.0014	0.0234	0.0103
rs13212041	T	0.0101	0.0018	-0.0065	0.0136
rs13240401	T	0.0176	0.0017	-0.0032	0.0124
rs13246220	T	-0.0093	0.0014	0.0056	0.0107
rs13261773	C	0.0122	0.0019	0.0127	0.0131
rs13266287	A	-0.0085	0.0015	-0.0008	0.0111
rs13281564	A	0.0088	0.0014	-0.0004	0.0104
rs13284516	C	-0.0082	0.0014	-0.0024	0.0110
rs1329044	T	0.0134	0.0019	-0.0283	0.0135
rs1329125	T	-0.0097	0.0015	0.0127	0.0121
rs13296345	T	0.0090	0.0015	-0.0192	0.0105
rs13318986	A	0.0124	0.0015	-0.0101	0.0110
rs13327482	A	-0.0121	0.0018	-0.0073	0.0145
rs1334297	A	0.0257	0.0016	-0.0058	0.0124
rs1335482	T	0.0096	0.0014	-0.0094	0.0107
rs13361043	T	-0.0158	0.0028	0.0180	0.0186
rs13381557	A	-0.0087	0.0014	-0.0035	0.0102
rs13388333	A	0.0085	0.0015	-0.0203	0.0113
rs13397529	C	-0.0097	0.0017	0.0123	0.0124
rs13398860	A	0.0086	0.0015	-0.0095	0.0111
rs13402497	A	-0.0080	0.0014	-0.0094	0.0103
rs13422673	T	-0.0110	0.0014	0.0070	0.0107
rs13425585	C	-0.0089	0.0014	0.0178	0.0104
rs1368250	T	0.0250	0.0045	0.0132	0.0437
rs137079	T	0.0128	0.0020	0.0144	0.0154
rs137858393	A	0.0193	0.0029	0.0456	0.0239
rs138096147	A	0.0234	0.0042	0.0607	0.0408
rs138484388	T	-0.0306	0.0051	0.0580	0.0462
rs1391513	T	-0.0095	0.0015	0.0048	0.0111
rs139244147	A	-0.0193	0.0030	-0.0136	0.0208
rs1392816	T	0.0082	0.0014	-0.0158	0.0107
rs139980871	T	0.0180	0.0029	-0.0098	0.0228
rs1404549	A	-0.0089	0.0015	0.0086	0.0112
rs1405876	T	0.0109	0.0015	-0.0018	0.0113
rs140711597	C	0.0366	0.0055	0.0637	0.0466
rs1408284	C	-0.0127	0.0020	0.0193	0.0159
rs1408430	C	0.0096	0.0016	0.0108	0.0115
rs141586924	A	-0.0090	0.0015	0.0024	0.0120
rs141729694	T	0.0195	0.0026	0.0461	0.0189
rs142014757	A	0.0102	0.0018	0.0024	0.0150

rs1426619	T	0.0096	0.0014	0.0123	0.0104
rs1427298	T	0.0086	0.0014	-0.0138	0.0106
rs142747148	A	0.0329	0.0052	-0.0431	0.0486
rs1427829	A	-0.0083	0.0014	0.0147	0.0102
rs143148393	T	-0.0211	0.0039	-0.0054	0.0351
rs1434630	T	-0.0137	0.0020	-0.0133	0.0137
rs143743568	A	0.0118	0.0020	-0.0085	0.0151
rs143812851	A	-0.0113	0.0019	0.0070	0.0141
rs1440930	C	0.0081	0.0014	0.0120	0.0104
rs144336753	A	0.0388	0.0053	-0.0411	0.0425
rs1445591	A	0.0085	0.0015	0.0045	0.0123
rs145018899	A	0.0095	0.0015	0.0085	0.0117
rs1464297	T	-0.0109	0.0015	-0.0156	0.0106
rs1467737	T	-0.0084	0.0014	0.0146	0.0107
rs1485300	C	0.0098	0.0015	-0.0100	0.0111
rs1490612	T	0.0193	0.0026	0.0000	0.0190
rs150252215	A	0.0204	0.0035	-0.0102	0.0289
rs150537577	A	-0.0151	0.0026	0.0165	0.0203
rs1505676	C	0.0084	0.0015	-0.0204	0.0104
rs151381	T	-0.0087	0.0014	0.0285	0.0105
rs1518890	T	-0.0090	0.0016	0.0015	0.0117
rs1527878	A	-0.0105	0.0016	0.0203	0.0126
rs1529597	A	0.0223	0.0038	-0.0332	0.0281
rs1538389	T	-0.0109	0.0018	0.0152	0.0133
rs1542354	A	-0.0084	0.0014	-0.0091	0.0106
rs1544	A	-0.0096	0.0016	0.0091	0.0115
rs1554798	A	-0.0080	0.0014	0.0173	0.0106
rs1564347	T	0.0088	0.0015	-0.0250	0.0108
rs1569092	A	0.0160	0.0020	-0.0086	0.0149
rs1593022	T	-0.0099	0.0017	0.0084	0.0126
rs1603460	T	0.0090	0.0014	-0.0024	0.0105
rs162445	A	0.0152	0.0026	0.0111	0.0170
rs163229	C	-0.0260	0.0047	0.0044	0.0344
rs1637770	T	0.0191	0.0028	-0.0091	0.0210
rs164938	T	-0.0088	0.0014	0.0037	0.0106
rs165633	A	-0.0116	0.0016	0.0053	0.0127
rs1656614	C	0.0122	0.0015	-0.0142	0.0118
rs1671269	T	-0.0100	0.0016	0.0035	0.0112
rs1671770	A	0.0125	0.0018	0.0119	0.0132
rs16851779	T	-0.0153	0.0027	0.0295	0.0218
rs16871807	T	0.0090	0.0015	-0.0080	0.0109
rs1689510	C	0.0194	0.0015	-0.0077	0.0115
rs16901689	T	0.0111	0.0019	-0.0294	0.0138
rs1693584	T	-0.0087	0.0015	0.0074	0.0112
rs16958559	T	0.0096	0.0017	0.0234	0.0142
rs16966271	T	0.0092	0.0016	-0.0020	0.0116

rs16975275	A	-0.0102	0.0017	-0.0006	0.0129
rs17048801	A	-0.0106	0.0014	0.0322	0.0112
rs17048855	A	0.0108	0.0015	0.0007	0.0108
rs17069646	T	0.0085	0.0015	0.0149	0.0109
rs17110109	T	-0.0093	0.0014	0.0140	0.0107
rs17113730	A	-0.0170	0.0027	0.0296	0.0200
rs17131123	A	-0.0243	0.0039	-0.0059	0.0404
rs17133297	A	-0.0146	0.0023	0.0121	0.0155
rs17144467	A	0.0098	0.0015	-0.0116	0.0110
rs17148998	A	0.0101	0.0017	-0.0109	0.0136
rs171697	C	0.0133	0.0015	-0.0096	0.0111
rs17170519	C	0.0132	0.0023	0.0045	0.0158
rs1717204	A	-0.0117	0.0018	-0.0088	0.0142
rs1718188	A	0.0096	0.0014	-0.0203	0.0105
rs17186106	T	0.0108	0.0018	-0.0191	0.0145
rs17190418	T	-0.0213	0.0031	0.0071	0.0276
rs17205908	T	-0.0092	0.0015	0.0139	0.0120
rs17248751	A	-0.0136	0.0017	0.0340	0.0141
rs1728118	A	0.0089	0.0016	-0.0146	0.0113
rs1729412	T	-0.0107	0.0014	0.0097	0.0103
rs173003	A	-0.0082	0.0014	-0.0048	0.0107
rs1734370	A	0.0099	0.0016	-0.0036	0.0112
rs1738050	C	-0.0093	0.0014	0.0014	0.0106
rs17411339	A	0.0140	0.0014	-0.0117	0.0110
rs17428076	C	0.0137	0.0016	0.0020	0.0123
rs17440885	A	0.0160	0.0029	0.0044	0.0246
rs1747714	T	0.0106	0.0014	0.0251	0.0103
rs1747817	T	-0.0092	0.0016	0.0387	0.0126
rs17489649	A	0.0127	0.0015	0.0000	0.0123
rs17502934	T	-0.0167	0.0020	0.0147	0.0146
rs175325	A	-0.0100	0.0014	-0.0049	0.0105
rs17536059	C	-0.0108	0.0019	0.0103	0.0143
rs17551064	A	0.0141	0.0019	0.0153	0.0149
rs17563464	A	-0.0147	0.0017	-0.0008	0.0159
rs17565975	A	-0.0106	0.0014	-0.0254	0.0103
rs17568389	A	0.0127	0.0014	-0.0038	0.0104
rs17574007	A	-0.0129	0.0021	0.0125	0.0177
rs1758747	A	0.0092	0.0015	-0.0155	0.0111
rs17598675	T	-0.0116	0.0014	0.0152	0.0105
rs17604349	A	0.0127	0.0018	-0.0100	0.0136
rs17609255	T	-0.0087	0.0014	0.0198	0.0104
rs176218	T	0.0200	0.0018	-0.0211	0.0124
rs17622379	T	-0.0113	0.0018	0.0002	0.0128
rs17650634	T	-0.0104	0.0019	0.0264	0.0150
rs17667540	A	-0.0087	0.0015	0.0066	0.0109
rs17669337	T	-0.0110	0.0014	-0.0050	0.0106

rs17680712	T	-0.0092	0.0014	0.0106	0.0104
rs17686649	T	-0.0098	0.0017	0.0403	0.0124
rs17732878	T	-0.0095	0.0017	-0.0216	0.0128
rs17742342	A	-0.0119	0.0018	0.0146	0.0135
rs17747544	A	-0.0090	0.0014	0.0079	0.0105
rs1779549	A	0.0081	0.0014	-0.0046	0.0108
rs178183	T	0.0145	0.0016	-0.0105	0.0118
rs17882802	A	0.0084	0.0014	-0.0141	0.0111
rs17883331	A	-0.0108	0.0018	0.0421	0.0141
rs1792602	A	-0.0097	0.0014	-0.0014	0.0109
rs181214	T	-0.0104	0.0017	0.0150	0.0144
rs182355396	A	-0.0525	0.0084	-0.0035	0.0774
rs182902112	A	-0.0300	0.0053	0.0217	0.0363
rs1835339	T	0.0084	0.0014	0.0170	0.0105
rs183869217	C	0.0174	0.0030	-0.0113	0.0236
rs1841023	A	-0.0092	0.0015	-0.0054	0.0117
rs1842713	A	-0.0117	0.0017	-0.0037	0.0120
rs1843815	A	0.0077	0.0014	-0.0202	0.0102
rs1861786	A	-0.0081	0.0014	0.0027	0.0105
rs1865955	T	0.0122	0.0019	-0.0126	0.0128
rs1866823	A	0.0107	0.0014	-0.0176	0.0107
rs187580	T	-0.0104	0.0017	0.0286	0.0136
rs187951956	A	-0.0315	0.0058	0.0060	0.0418
rs1880088	A	-0.0097	0.0016	0.0264	0.0112
rs1880692	A	0.0086	0.0014	-0.0012	0.0105
rs188251563	A	0.0428	0.0070	-0.1369	0.0654
rs1890132	T	-0.0089	0.0015	0.0106	0.0115
rs1898111	A	0.0101	0.0018	-0.0131	0.0128
rs190102446	T	-0.0210	0.0037	-0.0608	0.0299
rs1905616	A	0.0083	0.0015	-0.0220	0.0108
rs1910005	T	-0.0090	0.0015	-0.0041	0.0113
rs1918394	T	0.0119	0.0019	-0.0214	0.0136
rs192436652	T	-0.0319	0.0045	-0.0647	0.0430
rs1933264	T	0.0100	0.0016	-0.0009	0.0118
rs1952183	A	-0.0089	0.0014	0.0005	0.0106
rs1955250	A	-0.0140	0.0025	0.0331	0.0194
rs1963381	A	0.0099	0.0016	-0.0100	0.0117
rs1980129	A	0.0085	0.0014	-0.0009	0.0102
rs198262	T	-0.0223	0.0035	0.0036	0.0294
rs1991585	T	-0.0101	0.0015	-0.0039	0.0109
rs1995181	A	-0.0078	0.0014	-0.0156	0.0103
rs2007655	T	0.0086	0.0014	0.0064	0.0101
rs2011603	A	-0.0091	0.0016	0.0425	0.0121
rs2014830	T	0.0110	0.0015	-0.0259	0.0113
rs201495	T	-0.0112	0.0019	0.0275	0.0112
rs2023016	C	0.0107	0.0017	-0.0137	0.0120

rs2024568	T	-0.0097	0.0016	0.0079	0.0116
rs2029401	A	-0.0091	0.0014	0.0183	0.0107
rs2034631	T	0.0089	0.0015	-0.0083	0.0106
rs2039204	A	0.0082	0.0014	0.0031	0.0108
rs2043187	A	0.0107	0.0015	-0.0089	0.0120
rs2052285	A	0.0112	0.0014	-0.0152	0.0117
rs2055940	A	0.0082	0.0015	-0.0027	0.0120
rs2077235	T	0.0097	0.0017	0.0055	0.0119
rs2081652	A	0.0123	0.0015	0.0157	0.0112
rs2082317	T	-0.0078	0.0014	-0.0123	0.0105
rs2088913	A	0.0081	0.0014	0.0038	0.0103
rs2092248	T	-0.0083	0.0015	0.0125	0.0113
rs2098526	A	-0.0241	0.0043	0.0511	0.0277
rs2100249	T	-0.0089	0.0015	0.0139	0.0108
rs2126069	T	0.0080	0.0014	-0.0094	0.0105
rs2131167	A	0.0080	0.0014	-0.0229	0.0109
rs214626	A	0.0101	0.0018	-0.0096	0.0126
rs2160514	A	-0.0094	0.0014	0.0024	0.0105
rs2179152	T	-0.0131	0.0015	-0.0079	0.0108
rs2183271	T	0.0084	0.0015	-0.0049	0.0110
rs2199409	T	0.0103	0.0017	-0.0155	0.0130
rs2212430	T	-0.0100	0.0016	0.0125	0.0113
rs2216144	T	0.0098	0.0014	-0.0139	0.0105
rs2220926	T	-0.0095	0.0014	-0.0015	0.0103
rs2250660	C	0.0080	0.0014	-0.0103	0.0106
rs2252098	T	-0.0077	0.0014	-0.0106	0.0105
rs2254681	A	0.0101	0.0016	0.0046	0.0117
rs2256965	A	0.0106	0.0014	-0.0038	0.0111
rs2276209	A	0.0085	0.0015	-0.0171	0.0109
rs2283076	A	0.0115	0.0017	-0.0071	0.0127
rs2287838	A	-0.0104	0.0014	-0.0037	0.0105
rs2290601	T	-0.0149	0.0017	0.0055	0.0119
rs2297293	C	0.0099	0.0015	0.0022	0.0110
rs2299156	T	0.0099	0.0018	-0.0009	0.0128
rs2314338	T	0.0093	0.0016	-0.0110	0.0137
rs2321157	A	-0.0081	0.0014	-0.0110	0.0103
rs232496	T	0.0090	0.0015	0.0273	0.0105
rs2332179	A	0.0121	0.0020	-0.0123	0.0141
rs2336721	T	0.0095	0.0015	-0.0327	0.0109
rs2358628	A	-0.0091	0.0015	-0.0025	0.0110
rs236318	C	-0.0102	0.0017	-0.0032	0.0116
rs2364544	A	-0.0105	0.0014	0.0036	0.0107
rs2365376	A	0.0092	0.0015	0.0070	0.0108
rs2368831	T	-0.0092	0.0014	0.0041	0.0113
rs2373124	A	-0.0096	0.0016	0.0154	0.0132
rs2406253	A	0.0135	0.0018	0.0075	0.0145

rs2414072	A	-0.0083	0.0014	-0.0078	0.0104
rs2416214	A	0.0079	0.0014	0.0016	0.0104
rs2416759	A	-0.0093	0.0015	0.0075	0.0114
rs2416845	T	0.0263	0.0048	-0.0002	0.0341
rs2431023	A	0.0093	0.0014	-0.0046	0.0103
rs2434672	A	0.0091	0.0014	-0.0044	0.0105
rs2436760	T	0.0232	0.0042	0.0072	0.0306
rs2447097	T	0.0096	0.0014	0.0147	0.0109
rs2458370	T	-0.0086	0.0015	0.0134	0.0105
rs2469226	A	0.0096	0.0017	-0.0140	0.0128
rs2470966	C	-0.0103	0.0015	0.0113	0.0114
rs2478208	C	-0.0103	0.0014	-0.0031	0.0105
rs2496482	T	0.0121	0.0015	-0.0287	0.0115
rs2517086	C	-0.0088	0.0014	-0.0093	0.0112
rs2521602	A	-0.0266	0.0049	0.0104	0.0356
rs2522545	T	-0.0083	0.0014	0.0055	0.0105
rs2526398	C	-0.0222	0.0015	0.0150	0.0120
rs2529069	T	-0.0106	0.0016	0.0149	0.0113
rs252991	A	0.0095	0.0015	-0.0049	0.0107
rs2542673	A	0.0088	0.0015	0.0133	0.0113
rs2545795	A	0.0125	0.0014	0.0060	0.0104
rs255053	A	-0.0111	0.0018	0.0007	0.0127
rs2554835	A	0.0088	0.0014	-0.0026	0.0111
rs2568955	T	-0.0165	0.0016	0.0041	0.0123
rs2569041	T	0.0077	0.0014	-0.0058	0.0106
rs2570497	T	-0.0121	0.0015	0.0071	0.0106
rs2588959	T	-0.0080	0.0014	0.0027	0.0101
rs2657283	T	0.0085	0.0014	-0.0119	0.0108
rs2665668	A	-0.0094	0.0015	0.0231	0.0116
rs2702576	A	-0.0090	0.0014	0.0131	0.0103
rs2718277	T	0.0142	0.0026	-0.0176	0.0210
rs2718791	T	-0.0081	0.0015	-0.0095	0.0112
rs27220	A	-0.0114	0.0015	0.0072	0.0105
rs2725370	T	-0.0141	0.0015	0.0221	0.0111
rs2736752	T	0.0095	0.0017	0.0098	0.0140
rs2740795	A	0.0098	0.0016	-0.0079	0.0113
rs2761438	A	0.0098	0.0015	-0.0182	0.0109
rs2764684	T	0.0143	0.0019	-0.0185	0.0154
rs2805064	C	0.0093	0.0016	-0.0196	0.0124
rs2838006	T	0.0132	0.0015	-0.0175	0.0112
rs28482086	A	0.0086	0.0015	-0.0119	0.0107
rs28505285	C	0.0106	0.0018	0.0219	0.0144
rs28512462	C	0.0112	0.0014	-0.0095	0.0104
rs28513882	A	-0.0110	0.0018	0.0184	0.0150
rs2852349	T	-0.0093	0.0014	-0.0014	0.0102
rs28587776	T	0.0088	0.0015	0.0104	0.0114

rs28669886	A	-0.0084	0.0015	0.0167	0.0109
rs2870281	A	-0.0083	0.0014	-0.0090	0.0104
rs28735993	A	0.0162	0.0022	-0.0270	0.0177
rs2885198	A	0.0088	0.0014	-0.0210	0.0106
rs2898191	A	0.0093	0.0015	0.0073	0.0109
rs2910823	T	0.0115	0.0014	0.0081	0.0106
rs2916490	A	-0.0092	0.0015	0.0037	0.0111
rs2923424	A	0.0120	0.0014	0.0149	0.0105
rs2926702	T	-0.0124	0.0021	-0.0201	0.0136
rs2929032	A	-0.0081	0.0014	0.0070	0.0104
rs2929860	T	0.0110	0.0018	0.0268	0.0147
rs2942884	A	-0.0090	0.0014	0.0093	0.0105
rs2954114	A	-0.0081	0.0014	0.0040	0.0110
rs2958182	A	0.0082	0.0015	-0.0131	0.0112
rs2962378	A	0.0101	0.0017	-0.0133	0.0120
rs2964199	T	-0.0099	0.0015	0.0117	0.0110
rs2964255	A	0.0088	0.0015	0.0010	0.0111
rs2976397	T	0.0081	0.0014	0.0146	0.0104
rs2977464	T	0.0102	0.0018	-0.0046	0.0128
rs29792	A	-0.0093	0.0015	0.0075	0.0115
rs2980813	A	0.0088	0.0014	0.0189	0.0104
rs2989476	C	0.0078	0.0014	-0.0116	0.0104
rs2989751	A	-0.0092	0.0017	0.0127	0.0118
rs2992037	A	0.0149	0.0015	0.0178	0.0121
rs2994326	T	-0.0100	0.0018	0.0005	0.0135
rs2998299	T	-0.0136	0.0017	-0.0066	0.0134
rs3026996	A	0.0138	0.0016	-0.0077	0.0134
rs303752	A	-0.0110	0.0014	0.0236	0.0114
rs3111251	T	-0.0090	0.0014	0.0038	0.0108
rs312927	A	-0.0147	0.0022	-0.0203	0.0188
rs312945	A	-0.0091	0.0015	-0.0173	0.0111
rs317050	T	0.0078	0.0014	-0.0144	0.0103
rs322614	A	-0.0087	0.0014	0.0195	0.0105
rs322744	T	-0.0090	0.0016	-0.0123	0.0114
rs324885	A	0.0142	0.0014	0.0074	0.0106
rs32940	T	-0.0096	0.0015	0.0267	0.0114
rs339057	A	-0.0081	0.0014	-0.0067	0.0106
rs34067381	T	-0.0104	0.0015	-0.0058	0.0107
rs34098770	A	-0.0160	0.0020	0.0130	0.0138
rs34106693	C	0.0159	0.0019	-0.0118	0.0165
rs34122915	C	0.0084	0.0014	0.0014	0.0120
rs341504	A	0.0099	0.0015	-0.0091	0.0108
rs34155847	A	0.0135	0.0016	-0.0264	0.0124
rs34262657	T	-0.0240	0.0038	-0.0080	0.0245
rs34286836	T	-0.0089	0.0014	0.0050	0.0103
rs34298584	A	0.0111	0.0019	-0.0180	0.0140

rs34305371	A	0.0314	0.0024	0.0100	0.0198
rs34309	A	0.0080	0.0014	0.0130	0.0105
rs34316274	A	0.0097	0.0017	0.0217	0.0121
rs34363861	A	0.0087	0.0014	-0.0184	0.0113
rs34394051	A	-0.0117	0.0020	0.0139	0.0136
rs34410	C	-0.0083	0.0014	0.0185	0.0106
rs34624793	T	0.0116	0.0021	0.0177	0.0181
rs34720381	T	-0.0180	0.0024	0.0172	0.0196
rs34748029	A	0.0185	0.0025	-0.0422	0.0241
rs347661	T	-0.0081	0.0014	-0.0020	0.0107
rs34780702	A	0.0111	0.0017	0.0026	0.0117
rs34807077	A	0.0128	0.0019	-0.0157	0.0137
rs34811474	A	0.0104	0.0017	0.0070	0.0164
rs34967082	A	-0.0080	0.0014	-0.0042	0.0105
rs35016816	T	0.0176	0.0023	-0.0101	0.0162
rs350281	T	-0.0117	0.0015	0.0166	0.0130
rs35104491	A	0.0124	0.0018	0.0071	0.0126
rs35111506	C	-0.0175	0.0030	0.0366	0.0232
rs35192107	T	-0.0091	0.0017	0.0044	0.0131
rs35319653	T	0.0119	0.0015	-0.0005	0.0113
rs35417702	T	-0.0152	0.0014	0.0157	0.0103
rs35493937	C	0.0124	0.0017	-0.0209	0.0123
rs35606437	A	0.0105	0.0016	-0.0059	0.0115
rs356999	A	-0.0101	0.0014	0.0129	0.0110
rs35745455	A	0.0080	0.0014	-0.0095	0.0105
rs35754740	T	0.0083	0.0014	-0.0132	0.0105
rs35811586	T	0.0217	0.0031	-0.0251	0.0297
rs35929923	A	-0.0114	0.0016	0.0257	0.0119
rs36085856	C	0.0201	0.0036	0.0010	0.0278
rs36120534	T	0.0100	0.0018	0.0149	0.0144
rs362307	T	-0.0226	0.0027	0.0286	0.0219
rs363096	T	-0.0143	0.0014	0.0251	0.0103
rs3735478	T	0.0108	0.0016	-0.0356	0.0122
rs3751331	A	-0.0097	0.0014	0.0089	0.0108
rs3766979	A	0.0085	0.0015	-0.0189	0.0107
rs3768480	C	0.0101	0.0014	-0.0284	0.0107
rs3781339	T	-0.0104	0.0018	0.0403	0.0138
rs3790609	T	0.0107	0.0019	-0.0013	0.0138
rs3809169	T	0.0140	0.0025	-0.0148	0.0180
rs3809634	A	-0.0109	0.0015	0.0162	0.0119
rs3812281	T	0.0113	0.0014	-0.0116	0.0104
rs3817923	A	-0.0121	0.0022	0.0304	0.0155
rs382196	T	-0.0082	0.0014	0.0121	0.0107
rs3847228	T	0.0083	0.0014	0.0089	0.0110
rs3848715	A	-0.0080	0.0014	0.0070	0.0102
rs3859523	T	0.0124	0.0018	-0.0159	0.0135

rs387027	A	-0.0083	0.0015	0.0188	0.0108
rs3895736	A	0.0144	0.0019	0.0279	0.0139
rs3897821	A	0.0154	0.0015	-0.0149	0.0107
rs3948495	T	-0.0089	0.0014	0.0104	0.0105
rs399821	A	-0.0078	0.0014	-0.0121	0.0104
rs39998	A	0.0090	0.0015	-0.0173	0.0120
rs401526	T	-0.0104	0.0014	0.0047	0.0103
rs401966	C	-0.0097	0.0014	-0.0087	0.0111
rs4127499	A	0.0087	0.0015	-0.0125	0.0117
rs41282553	A	0.0237	0.0040	-0.0331	0.0319
rs42210	C	-0.0098	0.0016	-0.0179	0.0112
rs42302	A	0.0086	0.0015	-0.0011	0.0106
rs4255791	A	-0.0084	0.0015	-0.0038	0.0127
rs4263475	A	0.0078	0.0014	0.0104	0.0113
rs4283754	A	-0.0084	0.0014	0.0007	0.0127
rs429150	T	0.0085	0.0014	-0.0078	0.0111
rs4298514	T	0.0104	0.0015	0.0003	0.0110
rs4320563	A	0.0109	0.0014	-0.0219	0.0103
rs4328757	T	0.0096	0.0014	0.0018	0.0108
rs4352658	T	-0.0190	0.0026	0.0238	0.0185
rs4358081	A	-0.0095	0.0014	0.0093	0.0102
rs4358358	A	0.0109	0.0015	-0.0215	0.0112
rs4369924	A	0.0125	0.0019	-0.0023	0.0136
rs4384309	A	0.0102	0.0014	-0.0040	0.0109
rs4396896	A	-0.0107	0.0014	-0.0069	0.0104
rs4423373	A	-0.0087	0.0014	0.0042	0.0108
rs4426420	T	-0.0140	0.0019	0.0108	0.0136
rs4434676	A	-0.0089	0.0014	0.0097	0.0106
rs4458044	C	0.0101	0.0016	0.0085	0.0139
rs4467547	T	0.0121	0.0014	-0.0016	0.0114
rs4469771	T	0.0101	0.0015	0.0004	0.0109
rs4480339	A	-0.0097	0.0015	0.0191	0.0118
rs4490539	A	0.0142	0.0015	-0.0030	0.0118
rs4497562	A	0.0112	0.0016	-0.0115	0.0117
rs4500930	T	-0.0109	0.0015	0.0382	0.0108
rs4500960	T	-0.0131	0.0014	0.0068	0.0103
rs4502401	T	0.0085	0.0015	-0.0125	0.0109
rs4652135	A	0.0103	0.0016	-0.0123	0.0113
rs4658019	T	0.0084	0.0014	-0.0040	0.0103
rs4663617	A	0.0104	0.0017	-0.0052	0.0123
rs4664983	T	-0.0103	0.0018	0.0063	0.0125
rs4673840	T	-0.0138	0.0019	0.0065	0.0137
rs4675248	A	-0.0094	0.0014	-0.0129	0.0105
rs4685405	T	-0.0111	0.0018	-0.0063	0.0122
rs4687735	T	-0.0200	0.0033	-0.0428	0.0290
rs4691601	A	-0.0115	0.0014	0.0121	0.0103

rs4705763	C	0.0089	0.0014	0.0140	0.0105
rs4712371	A	-0.0124	0.0021	0.0221	0.0143
rs4719460	T	-0.0088	0.0015	0.0007	0.0112
rs4719944	T	-0.0121	0.0014	-0.0055	0.0103
rs4724083	T	0.0083	0.0015	-0.0082	0.0109
rs4726070	A	0.0122	0.0014	-0.0117	0.0104
rs4730020	T	0.0090	0.0016	-0.0005	0.0130
rs4731413	A	0.0111	0.0017	0.0036	0.0142
rs4735297	A	-0.0086	0.0015	-0.0153	0.0108
rs4739235	A	0.0109	0.0019	-0.0091	0.0134
rs4741571	A	-0.0102	0.0015	-0.0125	0.0119
rs4757957	C	0.0133	0.0015	-0.0164	0.0112
rs4766424	C	0.0142	0.0021	-0.0364	0.0177
rs4778058	T	-0.0092	0.0014	0.0174	0.0111
rs4785187	A	-0.0100	0.0017	-0.0151	0.0118
rs4787028	T	0.0086	0.0015	0.0071	0.0112
rs4787457	A	0.0162	0.0015	-0.0148	0.0111
rs4788115	A	0.0111	0.0019	-0.0316	0.0182
rs479018	A	0.0109	0.0015	0.0089	0.0122
rs4793090	A	0.0084	0.0015	-0.0421	0.0106
rs4810894	A	-0.0085	0.0015	0.0189	0.0106
rs4812697	T	0.0171	0.0027	-0.0095	0.0204
rs481940	T	0.0100	0.0016	-0.0168	0.0125
rs482787	T	0.0100	0.0015	-0.0228	0.0112
rs4839155	T	0.0115	0.0017	-0.0106	0.0122
rs4846010	A	0.0099	0.0018	-0.0425	0.0145
rs4846724	A	0.0098	0.0014	-0.0108	0.0102
rs4848924	A	-0.0117	0.0015	-0.0102	0.0108
rs4850954	T	0.0077	0.0014	0.0161	0.0108
rs4851263	A	-0.0151	0.0023	0.0562	0.0176
rs4877516	A	-0.0101	0.0014	0.0178	0.0103
rs4881269	A	0.0100	0.0014	0.0193	0.0105
rs488476	C	-0.0113	0.0015	-0.0003	0.0114
rs4894658	C	0.0091	0.0016	0.0087	0.0113
rs4895650	T	0.0093	0.0014	0.0045	0.0111
rs4899012	C	-0.0103	0.0014	0.0170	0.0105
rs4904523	A	-0.0082	0.0014	0.0185	0.0103
rs4915735	A	0.0116	0.0020	0.0054	0.0155
rs4919624	A	0.0193	0.0018	-0.0250	0.0133
rs4925065	T	-0.0078	0.0014	-0.0119	0.0105
rs4925109	A	-0.0096	0.0015	0.0308	0.0111
rs4938815	T	0.0085	0.0015	-0.0020	0.0115
rs4941735	T	0.0092	0.0014	-0.0037	0.0105
rs4972748	T	0.0105	0.0018	0.0119	0.0126
rs4977885	A	-0.0084	0.0014	0.0319	0.0107
rs4981245	A	0.0080	0.0014	0.0015	0.0109

rs4984541	A	-0.0138	0.0017	-0.0010	0.0129
rs4984613	T	0.0174	0.0028	0.0010	0.0198
rs4984682	C	-0.0138	0.0017	-0.0049	0.0123
rs548897	A	0.0081	0.0014	-0.0199	0.0106
rs55675587	T	-0.0103	0.0018	0.0001	0.0128
rs55736314	C	-0.0154	0.0014	0.0105	0.0113
rs55826493	T	-0.0127	0.0021	0.0423	0.0157
rs55986781	T	0.0187	0.0026	-0.0410	0.0224
rs56085180	A	-0.0269	0.0037	0.0096	0.0257
rs56099375	T	0.0120	0.0017	-0.0059	0.0137
rs56171318	T	-0.0137	0.0020	-0.0086	0.0139
rs56174996	A	-0.0143	0.0020	0.0060	0.0151
rs56306882	A	0.0086	0.0016	-0.0147	0.0127
rs56319902	T	-0.0206	0.0017	0.0484	0.0147
rs56391344	A	0.0148	0.0016	-0.0267	0.0129
rs563954	A	-0.0081	0.0014	-0.0015	0.0106
rs56405138	A	0.0153	0.0022	-0.0026	0.0153
rs56408528	T	-0.0082	0.0014	0.0082	0.0104
rs567003	A	0.0094	0.0015	0.0197	0.0114
rs56794817	A	0.0122	0.0019	-0.0097	0.0136
rs57016874	T	0.0209	0.0037	0.0016	0.0316
rs57148205	A	0.0095	0.0016	-0.0140	0.0125
rs57204268	A	0.0114	0.0020	0.0050	0.0154
rs57349798	A	0.0093	0.0014	-0.0474	0.0110
rs57352738	A	-0.0152	0.0017	-0.0044	0.0138
rs57437407	A	-0.0145	0.0023	-0.0293	0.0201
rs575113	A	0.0133	0.0015	-0.0182	0.0115
rs5754581	T	-0.0082	0.0014	0.0106	0.0103
rs5754753	T	-0.0126	0.0016	0.0046	0.0116
rs5754762	A	0.0197	0.0030	-0.0141	0.0198
rs57661533	T	-0.0114	0.0021	-0.0105	0.0135
rs580652	T	-0.0147	0.0024	-0.0109	0.0151
rs585557	A	0.0128	0.0018	-0.0295	0.0136
rs58779949	A	0.0110	0.0019	-0.0262	0.0151
rs58859557	T	0.0187	0.0028	0.0074	0.0214
rs58921703	T	0.0109	0.0015	-0.0101	0.0110
rs58950082	T	0.0095	0.0017	-0.0069	0.0124
rs58996896	A	-0.0094	0.0016	-0.0009	0.0124
rs590013	T	0.0100	0.0015	-0.0054	0.0110
rs59300999	T	-0.0164	0.0028	0.0128	0.0165
rs59480703	C	-0.0123	0.0018	-0.0152	0.0126
rs59813324	T	0.0097	0.0018	-0.0057	0.0124
rs59967356	T	-0.0127	0.0021	0.0319	0.0199
rs60096640	A	0.0159	0.0023	0.0084	0.0140
rs6020560	T	-0.0083	0.0014	-0.0094	0.0102
rs6043521	T	-0.0083	0.0014	0.0109	0.0111

rs60589532	A	0.0168	0.0028	-0.0019	0.0207
rs6060308	A	0.0095	0.0016	-0.0205	0.0113
rs6065080	T	-0.0130	0.0015	0.0101	0.0105
rs6065784	C	0.0110	0.0015	0.0004	0.0113
rs60717745	C	-0.0118	0.0019	0.0248	0.0137
rs60726488	T	-0.0090	0.0016	0.0027	0.0124
rs6091570	A	0.0081	0.0015	-0.0043	0.0107
rs61104616	A	-0.0172	0.0014	-0.0022	0.0104
rs6123924	A	0.0129	0.0019	-0.0413	0.0132
rs613872	T	-0.0163	0.0019	0.0305	0.0143
rs61387839	A	0.0088	0.0015	-0.0181	0.0122
rs61527214	A	0.0110	0.0014	0.0054	0.0115
rs61739710	A	-0.0099	0.0016	-0.0132	0.0116
rs61748951	A	-0.0273	0.0048	-0.0520	0.0502
rs61755388	T	0.0125	0.0017	-0.0045	0.0135
rs61757207	A	0.0359	0.0063	0.0761	0.0476
rs61798586	A	-0.0112	0.0020	0.0360	0.0158
rs61853335	T	-0.0109	0.0018	-0.0061	0.0128
rs61958175	A	-0.0198	0.0034	0.0284	0.0329
rs61997667	T	-0.0144	0.0020	0.0046	0.0148
rs62007304	A	0.0264	0.0047	0.0143	0.0500
rs62018216	T	0.0113	0.0019	0.0173	0.0151
rs62051146	A	-0.0134	0.0022	0.0189	0.0156
rs62090515	A	-0.0090	0.0015	0.0085	0.0108
rs62092949	T	-0.0087	0.0014	-0.0085	0.0106
rs62103198	T	-0.0147	0.0025	0.0159	0.0225
rs62109862	A	0.0136	0.0020	0.0114	0.0144
rs62142891	A	0.0108	0.0016	0.0065	0.0127
rs62155350	A	0.0338	0.0053	-0.0429	0.0473
rs62155770	A	-0.0196	0.0032	0.0430	0.0284
rs62155873	T	-0.0151	0.0021	-0.0189	0.0171
rs62174974	A	-0.0105	0.0018	0.0204	0.0122
rs62177359	A	-0.0275	0.0038	0.0004	0.0311
rs62179650	A	0.0112	0.0016	0.0074	0.0114
rs62182994	T	0.0137	0.0015	-0.0043	0.0115
rs62190914	T	0.0089	0.0015	0.0075	0.0112
rs62194170	A	0.0111	0.0017	-0.0157	0.0130
rs622169	T	0.0086	0.0014	-0.0068	0.0124
rs62247449	C	0.0114	0.0014	-0.0186	0.0107
rs62252819	A	-0.0094	0.0017	-0.0160	0.0125
rs62256284	T	0.0111	0.0017	0.0023	0.0126
rs62260764	C	-0.0132	0.0016	-0.0167	0.0120
rs62262671	A	0.0213	0.0020	-0.0280	0.0171
rs62340636	T	0.0103	0.0014	-0.0033	0.0104
rs62370510	A	-0.0109	0.0019	-0.0151	0.0146
rs62379838	T	0.0121	0.0015	-0.0163	0.0114

rs62409395	T	0.0101	0.0018	-0.0259	0.0184
rs62420387	T	-0.0184	0.0027	0.0143	0.0224
rs62506074	T	0.0091	0.0015	-0.0067	0.0122
rs62506104	A	-0.0098	0.0018	0.0128	0.0126
rs628993	A	0.0128	0.0023	0.0103	0.0161
rs631287	A	0.0084	0.0014	-0.0281	0.0104
rs633279	A	-0.0089	0.0015	-0.0096	0.0113
rs635754	A	0.0119	0.0014	0.0052	0.0102
rs6428587	T	0.0084	0.0015	-0.0022	0.0105
rs6435326	A	-0.0091	0.0014	-0.0097	0.0105
rs6442126	A	-0.0121	0.0015	0.0306	0.0114
rs6445633	A	0.0088	0.0015	0.0107	0.0124
rs6449503	A	0.0185	0.0014	-0.0035	0.0105
rs6450476	A	-0.0116	0.0015	0.0097	0.0114
rs6452793	T	0.0175	0.0017	-0.0074	0.0121
rs6461536	A	0.0088	0.0015	0.0123	0.0113
rs6469654	C	-0.0103	0.0017	-0.0059	0.0116
rs6472208	T	-0.0091	0.0014	0.0036	0.0107
rs6480234	T	-0.0084	0.0014	0.0160	0.0104
rs648044	A	0.0083	0.0015	0.0074	0.0115
rs6490618	T	0.0088	0.0015	-0.0098	0.0109
rs6493265	T	-0.0122	0.0014	0.0129	0.0108
rs6493275	A	0.0131	0.0017	0.0074	0.0135
rs6503409	T	0.0087	0.0015	0.0235	0.0117
rs6534338	T	0.0103	0.0015	-0.0146	0.0116
rs6543810	T	0.0082	0.0015	0.0075	0.0105
rs6557171	T	-0.0155	0.0015	-0.0008	0.0107
rs6567288	A	-0.0079	0.0014	0.0018	0.0103
rs6569077	T	0.0118	0.0014	-0.0032	0.0106
rs6573552	T	-0.0090	0.0014	0.0040	0.0102
rs6573559	T	0.0105	0.0015	0.0023	0.0111
rs660001	A	-0.0164	0.0017	0.0062	0.0126
rs663251	T	0.0081	0.0015	-0.0023	0.0105
rs66482320	C	0.0124	0.0022	0.0079	0.0165
rs66568921	T	-0.0181	0.0015	0.0164	0.0108
rs66671632	T	-0.0133	0.0021	0.0067	0.0148
rs66721975	A	-0.0087	0.0015	0.0016	0.0113
rs6672986	C	0.0094	0.0014	-0.0021	0.0102
rs6689641	A	0.0078	0.0014	0.0034	0.0105
rs6690195	T	-0.0113	0.0014	0.0103	0.0108
rs6695132	T	0.0097	0.0017	-0.0139	0.0117
rs6696068	T	-0.0088	0.0014	0.0093	0.0109
rs6697584	T	-0.0126	0.0017	0.0215	0.0135
rs6704768	A	-0.0116	0.0014	0.0215	0.0106
rs6706275	T	0.0083	0.0015	-0.0090	0.0117
rs6711399	T	-0.0107	0.0019	0.0135	0.0123

rs6715321	T	-0.0097	0.0014	0.0100	0.0107
rs6715849	A	-0.0122	0.0014	-0.0039	0.0105
rs6720515	A	0.0103	0.0017	0.0042	0.0122
rs67224963	A	0.0113	0.0017	-0.0060	0.0126
rs6729612	T	0.0092	0.0015	-0.0017	0.0109
rs6731373	A	-0.0115	0.0015	-0.0326	0.0121
rs6736025	T	0.0079	0.0014	-0.0158	0.0102
rs6738860	A	-0.0088	0.0014	0.0066	0.0105
rs6743032	A	-0.0165	0.0024	-0.0377	0.0195
rs6744040	A	-0.0080	0.0014	0.0100	0.0103
rs67456868	A	0.0119	0.0018	-0.0099	0.0146
rs6752228	T	-0.0080	0.0014	0.0072	0.0102
rs6757087	T	-0.0085	0.0014	0.0005	0.0108
rs6760887	T	0.0155	0.0022	-0.0095	0.0129
rs67661275	A	0.0134	0.0025	-0.0307	0.0195
rs6774533	T	0.0090	0.0015	-0.0082	0.0112
rs67790232	A	0.0101	0.0017	0.0059	0.0142
rs6780414	T	0.0105	0.0018	0.0007	0.0132
rs6792805	A	-0.0090	0.0016	-0.0116	0.0112
rs6812533	T	0.0106	0.0016	0.0111	0.0114
rs68145588	T	-0.0127	0.0021	-0.0063	0.0166
rs6824567	T	0.0130	0.0016	-0.0166	0.0122
rs6853599	T	0.0108	0.0019	-0.0113	0.0154
rs6861925	C	-0.0090	0.0014	0.0085	0.0102
rs6871635	A	-0.0087	0.0014	-0.0095	0.0110
rs6881581	A	-0.0096	0.0015	0.0297	0.0113
rs6881733	T	-0.0086	0.0016	-0.0056	0.0114
rs6917154	T	0.0127	0.0021	0.0202	0.0175
rs6917204	T	0.0119	0.0017	-0.0330	0.0117
rs6918506	C	0.0113	0.0014	0.0137	0.0103
rs6924023	A	0.0107	0.0016	0.0011	0.0116
rs6932108	C	-0.0210	0.0027	0.0211	0.0215
rs6946136	A	0.0081	0.0014	0.0031	0.0108
rs6946362	T	0.0091	0.0015	-0.0134	0.0121
rs6951996	A	-0.0182	0.0032	0.0262	0.0229
rs6956283	T	0.0098	0.0016	-0.0052	0.0117
rs6969783	A	-0.0098	0.0014	0.0230	0.0103
rs6977237	T	0.0082	0.0014	-0.0023	0.0104
rs6989141	A	0.0100	0.0017	-0.0018	0.0145
rs6994287	A	0.0102	0.0014	0.0007	0.0106
rs7012546	T	0.0092	0.0014	-0.0141	0.0104
rs7016302	C	-0.0125	0.0019	0.0050	0.0146
rs702606	T	0.0118	0.0021	-0.0115	0.0148
rs7029718	A	0.0244	0.0014	-0.0012	0.0105
rs7030373	A	-0.0098	0.0017	0.0058	0.0120
rs7035315	A	-0.0090	0.0015	-0.0092	0.0107

rs7040995	C	0.0097	0.0014	-0.0275	0.0104
rs7041702	A	-0.0111	0.0016	0.0247	0.0122
rs7084508	T	-0.0085	0.0015	-0.0139	0.0115
rs710629	A	0.0082	0.0015	-0.0130	0.0106
rs7108020	A	0.0111	0.0015	-0.0134	0.0112
rs711793	T	0.0103	0.0015	-0.0021	0.0128
rs7127580	C	-0.0150	0.0023	0.0098	0.0191
rs713584	A	-0.0080	0.0014	0.0030	0.0102
rs71413877	A	0.0351	0.0036	-0.0293	0.0334
rs7146625	A	0.0091	0.0016	-0.0150	0.0123
rs7147473	A	-0.0103	0.0015	0.0207	0.0118
rs7158218	A	-0.0090	0.0015	-0.0132	0.0117
rs71646142	T	0.0104	0.0018	-0.0002	0.0136
rs716513	A	0.0084	0.0014	-0.0086	0.0104
rs7167688	T	-0.0094	0.0014	-0.0020	0.0103
rs7171405	A	-0.0101	0.0016	0.0117	0.0119
rs717996	T	-0.0122	0.0014	0.0210	0.0113
rs7182216	C	-0.0096	0.0017	-0.0191	0.0136
rs7190	A	-0.0095	0.0015	0.0054	0.0122
rs721579	T	-0.0110	0.0016	0.0224	0.0115
rs7218235	A	-0.0097	0.0017	0.0080	0.0137
rs7223311	C	-0.0079	0.0014	0.0090	0.0112
rs7226824	T	-0.0078	0.0014	-0.0042	0.0104
rs72482130	T	0.0141	0.0019	-0.0104	0.0138
rs72486027	T	-0.0104	0.0016	0.0044	0.0116
rs7254263	T	-0.0119	0.0016	0.0026	0.0113
rs7255223	A	0.0097	0.0016	-0.0006	0.0114
rs72622559	T	-0.0120	0.0017	0.0105	0.0123
rs72624911	T	0.0282	0.0034	-0.0660	0.0306
rs72636697	T	-0.0140	0.0020	0.0016	0.0142
rs72667460	T	0.0197	0.0031	-0.0450	0.0233
rs72671456	A	-0.0174	0.0020	0.0334	0.0160
rs72672052	A	-0.0101	0.0018	0.0095	0.0131
rs72673097	A	0.0257	0.0042	-0.0065	0.0446
rs72677177	A	0.0100	0.0014	0.0101	0.0108
rs72694479	T	0.0127	0.0020	-0.0018	0.0155
rs72709560	A	0.0085	0.0015	-0.0085	0.0123
rs72771860	T	-0.0211	0.0029	-0.0046	0.0278
rs72792395	T	-0.0145	0.0022	0.0043	0.0162
rs728054	A	-0.0128	0.0015	0.0135	0.0108
rs72819118	T	-0.0231	0.0024	-0.0188	0.0210
rs72824753	T	0.0133	0.0024	-0.0103	0.0167
rs72828517	T	-0.0168	0.0019	0.0090	0.0140
rs72829857	A	-0.0149	0.0016	0.0066	0.0120
rs72834698	A	0.0117	0.0020	0.0009	0.0176
rs72881110	A	0.0146	0.0027	0.0159	0.0196

rs72883760	A	0.0101	0.0018	-0.0324	0.0133
rs72896637	T	0.0162	0.0028	-0.0358	0.0267
rs72902523	T	-0.0103	0.0016	-0.0121	0.0115
rs72906124	T	0.0185	0.0030	-0.0447	0.0245
rs72917504	T	-0.0178	0.0030	-0.0259	0.0228
rs72919450	T	-0.0132	0.0023	0.0119	0.0169
rs72944064	T	-0.0098	0.0016	-0.0079	0.0126
rs72962169	T	-0.0174	0.0019	-0.0209	0.0138
rs72972965	A	0.0083	0.0015	0.0031	0.0114
rs72993796	T	0.0127	0.0022	0.0184	0.0191
rs73034295	A	0.0099	0.0018	-0.0158	0.0143
rs730384	A	0.0101	0.0014	-0.0081	0.0106
rs73039077	C	0.0092	0.0016	-0.0093	0.0131
rs73055556	A	0.0111	0.0020	-0.0038	0.0151
rs73082325	C	-0.0252	0.0044	0.1306	0.0434
rs73106136	T	-0.0147	0.0026	-0.0008	0.0209
rs73154546	A	0.0197	0.0036	-0.0032	0.0316
rs7317761	C	0.0109	0.0016	0.0180	0.0111
rs73191311	A	-0.0094	0.0015	-0.0197	0.0116
rs7321274	A	0.0114	0.0018	-0.0111	0.0127
rs73219806	A	0.0106	0.0019	0.0059	0.0149
rs7323027	A	0.0130	0.0015	-0.0121	0.0106
rs7326331	A	-0.0128	0.0016	-0.0010	0.0114
rs73344830	A	0.0170	0.0014	0.0012	0.0108
rs73405293	A	0.0113	0.0020	-0.0079	0.0154
rs73457936	A	0.0144	0.0025	0.0110	0.0216
rs73518807	C	-0.0128	0.0021	0.0191	0.0120
rs7356536	T	-0.0129	0.0021	-0.0012	0.0156
rs736281	T	0.0086	0.0014	0.0194	0.0108
rs73643713	T	-0.0165	0.0027	0.0213	0.0198
rs736471	T	0.0083	0.0014	0.0042	0.0112
rs73648455	T	-0.0183	0.0026	0.0016	0.0185
rs737945	C	-0.0115	0.0014	0.0024	0.0105
rs73874335	T	-0.0178	0.0029	0.0158	0.0188
rs73961845	A	-0.0114	0.0014	0.0045	0.0110
rs74091672	A	-0.0153	0.0022	0.0115	0.0175
rs7430651	T	-0.0111	0.0016	0.0119	0.0111
rs743316	T	0.0112	0.0017	0.0130	0.0136
rs74415461	T	0.0176	0.0026	-0.0249	0.0240
rs74453875	A	0.0214	0.0037	0.0007	0.0249
rs74462621	T	0.0125	0.0020	0.0044	0.0160
rs7449561	A	0.0102	0.0017	0.0199	0.0122
rs7451726	A	0.0109	0.0017	0.0014	0.0124
rs74545339	A	-0.0158	0.0022	-0.0287	0.0164
rs7460106	T	-0.0123	0.0017	0.0117	0.0134
rs74615093	A	0.0145	0.0026	-0.0284	0.0214

rs746839	C	0.0135	0.0015	0.0120	0.0115
rs74747621	T	0.0153	0.0022	-0.0287	0.0172
rs74787922	A	0.0182	0.0028	0.0202	0.0219
rs74944275	T	0.0231	0.0035	-0.0524	0.0314
rs74944857	T	-0.0089	0.0015	0.0126	0.0114
rs7495033	A	0.0095	0.0015	-0.0238	0.0111
rs75033012	C	-0.0258	0.0038	0.0045	0.0379
rs75177132	T	0.0298	0.0036	-0.0008	0.0425
rs75203411	T	0.0135	0.0019	-0.0058	0.0142
rs7526112	T	0.0124	0.0015	0.0028	0.0106
rs75308819	A	0.0287	0.0047	0.0006	0.0398
rs75434274	A	0.0151	0.0026	-0.0563	0.0215
rs75500877	T	0.0207	0.0033	-0.0139	0.0279
rs7552964	A	0.0148	0.0023	-0.0178	0.0174
rs7560871	A	-0.0193	0.0027	0.0050	0.0214
rs7561705	A	0.0078	0.0014	-0.0024	0.0106
rs7575637	A	0.0107	0.0014	0.0230	0.0103
rs75756843	A	0.0288	0.0049	0.0029	0.0382
rs7575938	A	0.0090	0.0015	-0.0048	0.0106
rs7590368	T	-0.0146	0.0016	0.0115	0.0119
rs7597126	T	-0.0094	0.0014	0.0010	0.0108
rs7597412	C	0.0088	0.0015	0.0107	0.0109
rs7603132	A	0.0137	0.0018	-0.0239	0.0128
rs76076331	T	0.0206	0.0021	-0.0319	0.0158
rs76077165	A	0.0165	0.0025	0.0112	0.0203
rs76167224	T	-0.0187	0.0033	-0.0233	0.0345
rs7617204	A	-0.0097	0.0014	-0.0141	0.0103
rs76235882	A	0.0241	0.0039	-0.0273	0.0308
rs76241605	A	0.0139	0.0022	-0.0054	0.0206
rs76246107	A	-0.0162	0.0026	-0.0266	0.0259
rs7625428	T	0.0098	0.0014	0.0060	0.0107
rs76267866	A	0.0104	0.0017	-0.0175	0.0130
rs7630133	A	0.0082	0.0015	-0.0033	0.0105
rs763553	A	0.0087	0.0014	0.0035	0.0104
rs7640424	T	0.0113	0.0015	-0.0074	0.0119
rs7650602	T	-0.0089	0.0014	0.0207	0.0108
rs76552497	T	-0.0120	0.0019	0.0290	0.0143
rs76577427	C	0.0203	0.0024	-0.0135	0.0221
rs7672622	A	-0.0088	0.0016	0.0167	0.0114
rs767943	A	-0.0122	0.0016	0.0202	0.0114
rs7683416	T	0.0139	0.0014	-0.0011	0.0104
rs76876592	A	0.0126	0.0020	0.0193	0.0151
rs76878669	C	0.0141	0.0017	-0.0014	0.0124
rs7692359	T	-0.0109	0.0017	0.0298	0.0129
rs76957677	T	-0.0297	0.0054	0.0244	0.0718
rs77025239	A	-0.0140	0.0019	0.0068	0.0142

rs7714719	C	0.0116	0.0020	0.0219	0.0147
rs7716161	C	0.0105	0.0019	0.0259	0.0140
rs77201694	A	0.0156	0.0022	-0.0048	0.0205
rs77370942	A	-0.0147	0.0027	0.0027	0.0235
rs77554090	T	-0.0186	0.0027	0.0373	0.0230
rs77609760	A	0.0236	0.0028	-0.0051	0.0237
rs7766240	T	-0.0134	0.0024	0.0160	0.0189
rs77702622	A	-0.0213	0.0029	-0.0030	0.0251
rs77702819	T	0.0187	0.0025	-0.0358	0.0218
rs7772172	A	0.0091	0.0014	-0.0189	0.0106
rs7775100	T	-0.0087	0.0014	-0.0165	0.0106
rs77826402	T	0.0151	0.0027	-0.0092	0.0198
rs7796103	C	-0.0077	0.0014	0.0011	0.0103
rs7799141	A	0.0099	0.0015	-0.0094	0.0111
rs77999825	A	0.0294	0.0039	-0.0171	0.0368
rs7803932	A	0.0127	0.0019	-0.0155	0.0150
rs780569	A	-0.0085	0.0016	0.0141	0.0114
rs78116078	C	0.0086	0.0016	-0.0021	0.0122
rs7816777	T	-0.0088	0.0015	0.0078	0.0112
rs78193153	A	0.0191	0.0029	-0.0206	0.0190
rs7823700	T	0.0132	0.0024	-0.0310	0.0196
rs78365243	T	0.0181	0.0033	0.0167	0.0298
rs78440611	A	-0.0147	0.0024	0.0196	0.0172
rs7849480	A	-0.0084	0.0014	0.0211	0.0105
rs7849487	T	-0.0167	0.0015	-0.0154	0.0110
rs7855503	C	0.0088	0.0015	0.0089	0.0109
rs78648104	T	-0.0141	0.0025	0.0156	0.0188
rs78702390	C	0.0167	0.0030	-0.0066	0.0263
rs78714229	T	0.0172	0.0030	0.0263	0.0236
rs7875078	A	-0.0078	0.0014	0.0258	0.0105
rs78918150	T	-0.0080	0.0015	0.0025	0.0107
rs7894722	T	0.0085	0.0014	0.0117	0.0110
rs7899270	A	-0.0084	0.0015	0.0088	0.0112
rs7905192	T	0.0084	0.0014	0.0363	0.0103
rs790647	A	-0.0145	0.0017	-0.0079	0.0118
rs7910403	T	0.0122	0.0018	0.0075	0.0141
rs7914674	T	-0.0139	0.0022	0.0115	0.0131
rs7920624	A	0.0120	0.0014	-0.0128	0.0102
rs7924036	T	0.0133	0.0014	0.0049	0.0101
rs79265434	A	-0.0197	0.0022	0.0136	0.0181
rs7928017	A	0.0096	0.0014	-0.0018	0.0104
rs7931563	T	-0.0109	0.0014	-0.0040	0.0106
rs795230	T	0.0081	0.0014	-0.0028	0.0104
rs7958371	A	-0.0085	0.0015	0.0061	0.0107
rs79585412	T	-0.0178	0.0031	-0.0453	0.0241
rs7965154	A	-0.0085	0.0015	0.0034	0.0110

rs7967550	A	-0.0086	0.0014	0.0120	0.0104
rs7972246	T	0.0110	0.0015	-0.0226	0.0108
rs79728014	A	-0.0134	0.0020	0.0040	0.0161
rs7974852	A	0.0117	0.0014	-0.0056	0.0104
rs7977614	A	-0.0134	0.0016	0.0255	0.0128
rs79798166	A	0.0172	0.0025	-0.0047	0.0215
rs79855925	T	-0.0194	0.0033	0.0143	0.0289
rs79994730	T	0.0240	0.0035	-0.0109	0.0310
rs79997166	A	0.0172	0.0030	-0.0260	0.0275
rs80037907	T	-0.0107	0.0019	-0.0092	0.0152
rs8008382	T	-0.0106	0.0015	-0.0005	0.0110
rs8009933	A	0.0088	0.0015	-0.0075	0.0108
rs8016504	A	-0.0087	0.0014	0.0083	0.0109
rs80223410	T	-0.0118	0.0020	0.0250	0.0202
rs8024	A	-0.0115	0.0015	0.0462	0.0111
rs80257979	T	-0.0252	0.0040	-0.0556	0.0324
rs8030487	A	0.0085	0.0015	-0.0265	0.0115
rs803619	T	-0.0173	0.0023	0.0269	0.0169
rs8046072	A	0.0101	0.0018	-0.0227	0.0128
rs8052523	T	-0.0084	0.0014	-0.0076	0.0112
rs8066044	A	0.0090	0.0016	-0.0142	0.0116
rs806816	A	0.0101	0.0016	-0.0040	0.0123
rs8097125	T	0.0086	0.0014	0.0033	0.0109
rs8103741	A	-0.0108	0.0019	-0.0022	0.0149
rs818415	T	-0.0110	0.0018	0.0029	0.0125
rs8192465	A	-0.0272	0.0049	-0.0187	0.0474
rs852771	T	0.0092	0.0015	0.0104	0.0115
rs853286	T	0.0124	0.0023	-0.0106	0.0148
rs854796	A	0.0090	0.0015	-0.0001	0.0110
rs868456	A	0.0089	0.0015	-0.0059	0.0115
rs870589	A	-0.0079	0.0014	0.0143	0.0104
rs884108	A	-0.0094	0.0017	-0.0059	0.0129
rs891793	C	-0.0099	0.0014	0.0020	0.0104
rs893522	A	0.0149	0.0025	-0.0183	0.0186
rs902820	A	-0.0096	0.0014	0.0025	0.0106
rs911149	T	0.0091	0.0017	-0.0109	0.0119
rs912883	T	0.0087	0.0015	0.0006	0.0109
rs913509	A	0.0096	0.0015	-0.0065	0.0109
rs925161	C	-0.0078	0.0014	-0.0109	0.0104
rs9267658	T	0.0138	0.0020	-0.0457	0.0185
rs9267677	T	0.0180	0.0024	0.0229	0.0168
rs9289300	T	-0.0164	0.0019	0.0233	0.0142
rs9291437	C	-0.0081	0.0014	0.0085	0.0104
rs9294770	T	0.0083	0.0014	-0.0089	0.0105
rs929511	T	-0.0160	0.0021	0.0041	0.0154
rs9300612	T	0.0084	0.0015	0.0076	0.0108

rs933738	A	-0.0110	0.0018	0.0030	0.0138
rs9349956	A	-0.0159	0.0018	-0.0252	0.0139
rs9359939	A	-0.0105	0.0016	-0.0185	0.0116
rs936496	A	-0.0079	0.0014	0.0048	0.0107
rs9371883	C	-0.0087	0.0015	0.0040	0.0108
rs9373363	A	-0.0111	0.0016	-0.0062	0.0120
rs9375188	T	0.0212	0.0014	0.0014	0.0104
rs9375403	C	0.0126	0.0016	-0.0154	0.0125
rs9386110	T	-0.0163	0.0023	0.0021	0.0170
rs9388490	T	0.0091	0.0014	-0.0333	0.0106
rs939400	T	-0.0095	0.0015	0.0068	0.0107
rs9411331	A	0.0145	0.0015	-0.0220	0.0109
rs9435340	A	0.0086	0.0015	-0.0137	0.0107
rs9442750	A	-0.0089	0.0016	-0.0001	0.0122
rs9446060	A	0.0077	0.0014	-0.0004	0.0103
rs9465509	A	-0.0084	0.0014	0.0044	0.0102
rs9490512	A	-0.0136	0.0014	0.0125	0.0104
rs9492774	C	0.0080	0.0015	-0.0197	0.0104
rs9513416	A	-0.0116	0.0019	-0.0063	0.0149
rs9513754	T	0.0096	0.0016	0.0086	0.0124
rs9527662	A	0.0094	0.0014	0.0038	0.0110
rs9527905	A	-0.0094	0.0014	-0.0126	0.0104
rs9529146	T	0.0127	0.0017	-0.0107	0.0116
rs9536462	A	0.0133	0.0020	-0.0133	0.0144
rs9540718	A	-0.0089	0.0014	0.0159	0.0104
rs9545395	T	0.0138	0.0021	-0.0039	0.0146
rs9556958	T	-0.0110	0.0014	-0.0014	0.0104
rs9563168	A	0.0101	0.0017	-0.0020	0.0128
rs9568798	T	-0.0093	0.0016	0.0064	0.0115
rs9597907	T	0.0152	0.0027	-0.0066	0.0176
rs9611597	A	-0.0107	0.0019	0.0076	0.0166
rs9616906	A	0.0122	0.0014	-0.0094	0.0109
rs9616947	T	-0.0134	0.0015	0.0275	0.0118
rs9633970	T	0.0091	0.0016	0.0000	0.0123
rs9649	T	0.0108	0.0019	-0.0012	0.0134
rs9655780	A	-0.0153	0.0019	-0.0271	0.0155
rs9666728	T	-0.0082	0.0014	-0.0017	0.0104
rs9683585	C	0.0084	0.0014	0.0079	0.0102
rs969512	A	-0.0106	0.0015	0.0086	0.0107
rs9771228	T	0.0096	0.0015	-0.0197	0.0106
rs977143	A	0.0085	0.0015	-0.0008	0.0110
rs978807	A	0.0139	0.0018	-0.0233	0.0131
rs981230	T	0.0100	0.0014	0.0136	0.0103
rs981883	A	0.0099	0.0014	-0.0187	0.0105
rs9820604	T	0.0115	0.0018	-0.0103	0.0128
rs9821664	A	0.0094	0.0017	-0.0057	0.0118

rs9830359	T	-0.0147	0.0022	0.0253	0.0159
rs9844755	A	0.0083	0.0015	-0.0028	0.0110
rs9849884	A	-0.0090	0.0014	0.0033	0.0109
rs9853928	T	-0.0127	0.0017	0.0273	0.0118
rs9858921	A	-0.0087	0.0014	-0.0018	0.0104
rs9859556	T	0.0290	0.0015	-0.0423	0.0115
rs9866123	A	0.0086	0.0014	-0.0178	0.0108
rs9870317	A	0.0086	0.0015	-0.0306	0.0109
rs9877225	T	0.0090	0.0016	-0.0093	0.0118
rs9882532	T	0.0124	0.0015	0.0019	0.0111
rs9886703	A	-0.0136	0.0019	0.0175	0.0137
rs9888796	T	0.0113	0.0016	-0.0086	0.0117
rs9916901	T	0.0092	0.0016	-0.0310	0.0111
rs9924031	C	0.0093	0.0015	0.0036	0.0115
rs9926649	T	0.0177	0.0031	-0.0302	0.0254
rs9927049	A	0.0099	0.0016	0.0109	0.0129
rs9927137	A	0.0082	0.0014	0.0052	0.0115
rs9927842	T	-0.0130	0.0020	-0.0067	0.0142
rs9929556	T	-0.0099	0.0014	0.0150	0.0105
rs9929762	A	0.0096	0.0014	0.0046	0.0105
rs9929993	T	-0.0102	0.0015	0.0000	0.0109
rs9933256	A	0.0119	0.0014	0.0003	0.0123
rs995698	A	-0.0099	0.0014	-0.0070	0.0106
rs9974899	T	-0.0093	0.0016	-0.0011	0.0119
rs9977825	T	-0.0092	0.0015	0.0307	0.0114

Supplementary Table 10: Genetic association estimates for the IVW ratio-method MR analysis estimating the effect of education on SBP. ea=effect allele; gx=education; gy=SBP; se=standard error

SNP	ea	gx	gx_se	gy	gy_se
rs10006235	T	-0.0093	0.0016	-0.0395	0.0497
rs10009513	A	0.0088	0.0014	0.0018	0.0442
rs10032941	T	-0.0080	0.0015	0.0894	0.0460
rs10041403	T	0.0151	0.0019	0.0401	0.0586
rs10042828	A	-0.0145	0.0022	0.1308	0.0682
rs10046069	A	0.0127	0.0022	0.0832	0.0700
rs10057590	A	0.0106	0.0014	-0.0603	0.0447
rs10060023	T	0.0110	0.0015	-0.0364	0.0467
rs10061420	A	0.0082	0.0014	-0.0057	0.0443
rs1006749	A	0.0079	0.0014	-0.0187	0.0442
rs10074178	A	0.0079	0.0014	-0.0822	0.0443
rs1007731	A	-0.0143	0.0022	0.1084	0.0691
rs10080647	A	0.0124	0.0020	-0.1050	0.0641
rs1008078	T	-0.0182	0.0014	0.0074	0.0452
rs1009470	T	0.0086	0.0014	-0.0915	0.0445
rs10098073	A	-0.0091	0.0014	0.1493	0.0444
rs10122669	A	-0.0079	0.0014	-0.0819	0.0448
rs10128888	A	-0.0083	0.0015	-0.0346	0.0486
rs10145520	T	-0.0122	0.0018	0.0325	0.0554
rs10166286	T	0.0151	0.0019	-0.1156	0.0586
rs10169002	A	-0.0080	0.0014	-0.0320	0.0444
rs10181071	A	0.0102	0.0019	-0.0164	0.0584
rs10189857	A	0.0158	0.0014	-0.0370	0.0446
rs10192369	A	-0.0078	0.0014	0.0122	0.0442
rs10192834	T	0.0083	0.0015	-0.0126	0.0464
rs10193498	A	0.0097	0.0016	0.1208	0.0522
rs10204051	T	-0.0101	0.0014	-0.0294	0.0453
rs10205057	T	0.0090	0.0014	-0.0305	0.0445
rs10208	T	0.0091	0.0015	0.0797	0.0478
rs10215082	A	-0.0149	0.0014	0.0204	0.0443
rs1024268	T	-0.0084	0.0014	-0.0620	0.0448
rs10251438	A	0.0078	0.0014	-0.0583	0.0445
rs10264573	A	0.0175	0.0032	-0.0713	0.1022
rs1035578	A	-0.0094	0.0014	-0.0513	0.0445
rs10402747	T	0.0077	0.0014	-0.1542	0.0444
rs10411759	A	0.0116	0.0020	-0.3387	0.0624
rs10444280	T	0.0100	0.0018	0.0137	0.0549
rs10460095	A	-0.0124	0.0014	-0.0277	0.0447
rs10480450	A	0.0158	0.0028	0.0624	0.0938
rs10496091	A	-0.0131	0.0016	0.1681	0.0492
rs10499535	A	0.0080	0.0014	-0.0400	0.0442

rs1050847	T	0.0092	0.0014	0.0610	0.0449
rs10509251	T	-0.0093	0.0017	0.0569	0.0532
rs10515007	T	0.0143	0.0019	-0.0922	0.0605
rs1051860	A	-0.0079	0.0014	-0.0216	0.0449
rs10519504	T	0.0119	0.0019	0.0685	0.0610
rs1054442	A	-0.0136	0.0015	0.0070	0.0457
rs1061801	A	-0.0110	0.0018	0.0654	0.0562
rs1066769	A	-0.0230	0.0041	0.1637	0.1200
rs10742591	A	-0.0084	0.0014	0.0245	0.0454
rs10750539	A	-0.0095	0.0015	-0.0672	0.0464
rs10752262	T	0.0102	0.0014	-0.0716	0.0446
rs1075228	A	-0.0081	0.0014	-0.0255	0.0448
rs10761202	T	0.0088	0.0014	-0.0086	0.0442
rs10761251	A	0.0113	0.0015	0.0800	0.0471
rs10772644	C	0.0142	0.0022	-0.1412	0.0715
rs10773002	A	0.0185	0.0016	-0.1224	0.0513
rs10773208	T	-0.0110	0.0016	-0.0001	0.0506
rs10782651	T	-0.0082	0.0014	-0.0246	0.0446
rs10783243	A	-0.0092	0.0014	0.0080	0.0443
rs10789285	T	0.0091	0.0016	-0.0654	0.0527
rs10793903	A	0.0126	0.0022	-0.0109	0.0719
rs10795831	T	-0.0099	0.0017	-0.0020	0.0546
rs10798888	T	-0.0140	0.0019	0.0737	0.0586
rs10799615	A	-0.0089	0.0016	-0.0256	0.0507
rs10805383	A	-0.0103	0.0014	0.0683	0.0443
rs10810099	A	-0.0146	0.0016	0.0260	0.0494
rs10810145	A	0.0078	0.0014	-0.1067	0.0449
rs10830858	T	0.0092	0.0014	-0.1799	0.0442
rs10831656	T	0.0090	0.0015	-0.0114	0.0481
rs10844179	A	0.0104	0.0017	-0.0852	0.0534
rs10845051	A	0.0083	0.0014	-0.0334	0.0444
rs10875121	C	0.0174	0.0019	0.1168	0.0596
rs10877283	T	0.0085	0.0014	-0.0001	0.0451
rs10879676	T	-0.0083	0.0014	0.0993	0.0449
rs10886012	C	0.0078	0.0014	0.0591	0.0448
rs10892807	T	0.0109	0.0014	-0.1401	0.0448
rs10899282	A	0.0140	0.0017	-0.1262	0.0539
rs10927053	A	-0.0125	0.0022	0.0073	0.0714
rs10928190	T	-0.0087	0.0014	-0.0018	0.0445
rs10931821	A	-0.0141	0.0014	0.0703	0.0443
rs10937240	T	-0.0123	0.0018	-0.0590	0.0564
rs10940540	A	0.0078	0.0014	0.0789	0.0445
rs10940921	T	0.0111	0.0014	0.0207	0.0450
rs10943588	A	0.0084	0.0015	-0.2603	0.0456
rs10947439	T	0.0090	0.0015	-0.0366	0.0472
rs10949263	A	-0.0091	0.0016	0.0543	0.0519

rs10951590	T	-0.0109	0.0015	0.0719	0.0470
rs10974256	A	-0.0093	0.0015	-0.0697	0.0468
rs1097784	T	-0.0083	0.0014	-0.0926	0.0443
rs10979613	T	-0.0117	0.0015	0.0375	0.0470
rs10983324	A	0.0084	0.0015	0.1482	0.0485
rs10984445	A	0.0115	0.0014	-0.0293	0.0445
rs10985402	T	0.0089	0.0016	0.0152	0.0517
rs10995639	T	0.0078	0.0014	-0.1465	0.0448
rs11003463	T	0.0091	0.0015	0.0430	0.0453
rs11021432	A	0.0103	0.0015	0.0266	0.0455
rs11023764	A	0.0091	0.0015	-0.0675	0.0472
rs11030084	T	0.0106	0.0018	-0.0168	0.0566
rs11030102	C	0.0101	0.0016	0.0157	0.0501
rs1106090	A	0.0094	0.0014	-0.0769	0.0462
rs11076962	T	0.0086	0.0016	0.0373	0.0489
rs1107871	A	-0.0083	0.0014	-0.0425	0.0445
rs11081529	T	0.0126	0.0015	-0.0574	0.0486
rs11082011	T	0.0194	0.0015	-0.0672	0.0471
rs11100237	A	-0.0101	0.0014	0.0863	0.0442
rs11115056	A	0.0187	0.0023	-0.2173	0.0746
rs11121177	A	0.0151	0.0018	-0.1586	0.0585
rs111226181	T	-0.0201	0.0018	0.1488	0.0580
rs111235962	T	0.0133	0.0024	0.1370	0.0741
rs11130380	T	0.0090	0.0014	0.0977	0.0451
rs111370527	T	0.0151	0.0027	0.0489	0.0814
rs11138947	T	0.0105	0.0016	-0.0895	0.0494
rs111530150	T	0.0269	0.0048	-0.1564	0.1882
rs11155813	T	-0.0129	0.0023	0.3294	0.0741
rs11157931	A	-0.0139	0.0014	0.1772	0.0452
rs11158800	A	-0.0091	0.0014	-0.0267	0.0443
rs1117152	T	0.0106	0.0015	-0.0607	0.0486
rs11174399	A	0.0111	0.0018	-0.0974	0.0547
rs111821073	T	0.0124	0.0019	-0.0615	0.0612
rs111852224	T	0.0156	0.0022	-0.0815	0.0675
rs11190955	T	-0.0083	0.0015	0.1176	0.0462
rs1120924	T	0.0108	0.0015	-0.0207	0.0465
rs11209894	A	0.0119	0.0021	0.0636	0.0642
rs11210400	A	0.0120	0.0014	0.0281	0.0445
rs11211123	A	0.0104	0.0017	-0.1296	0.0524
rs11213482	A	-0.0106	0.0019	0.0640	0.0582
rs112210983	A	0.0105	0.0017	-0.0031	0.0537
rs1123285	C	-0.0094	0.0015	0.0421	0.0475
rs112375785	A	0.0184	0.0021	-0.1935	0.0632
rs112379405	C	-0.0082	0.0014	-0.0325	0.0446
rs112512729	T	-0.0166	0.0027	0.1393	0.0879
rs11265191	T	0.0083	0.0015	0.0248	0.0467

rs112806496	C	-0.0160	0.0025	-0.0852	0.0768
rs1128956	T	-0.0140	0.0019	-0.1189	0.0577
rs113205706	A	-0.0135	0.0024	-0.1382	0.0746
rs113520408	A	0.0155	0.0016	-0.1223	0.0491
rs113552169	C	0.0294	0.0053	0.1768	0.1662
rs113588399	T	-0.0104	0.0017	0.0842	0.0542
rs113615161	T	-0.0123	0.0021	0.0547	0.0649
rs113720505	A	0.0164	0.0029	-0.0808	0.0917
rs113731629	T	0.0339	0.0061	-0.2119	0.1908
rs114192810	T	-0.0279	0.0051	0.1908	0.1567
rs114468556	A	0.0293	0.0036	-0.1341	0.1060
rs114593137	A	-0.0122	0.0017	0.1157	0.0548
rs1146079	C	-0.0087	0.0016	0.0946	0.0503
rs114810763	A	-0.0121	0.0020	0.0681	0.0589
rs114952970	T	0.0265	0.0040	0.0205	0.1313
rs114958262	T	-0.0220	0.0039	-0.0636	0.1214
rs115000530	A	-0.0250	0.0031	0.2215	0.0973
rs115017135	A	-0.0206	0.0038	0.2565	0.1196
rs11542663	A	0.0112	0.0015	-0.1018	0.0486
rs115438240	T	-0.0169	0.0030	-0.0608	0.1000
rs115693355	A	0.0309	0.0053	-0.2054	0.1635
rs115732722	A	-0.0415	0.0072	-0.4482	0.2584
rs11588857	A	0.0199	0.0017	-0.0519	0.0542
rs11591870	T	0.0098	0.0017	-0.0696	0.0541
rs11592299	A	0.0097	0.0018	-0.1175	0.0547
rs11596387	T	0.0258	0.0046	-0.0052	0.1510
rs11598765	A	-0.0106	0.0018	0.0630	0.0564
rs11599236	T	-0.0106	0.0014	-0.0839	0.0455
rs11613431	A	-0.0094	0.0016	-0.0867	0.0500
rs11620355	A	0.0182	0.0025	-0.1094	0.0793
rs11623285	T	-0.0135	0.0021	0.1113	0.0643
rs116386746	C	0.0249	0.0045	-0.0148	0.1433
rs11640569	A	0.0112	0.0020	-0.0580	0.0642
rs11644446	A	0.0117	0.0019	0.1033	0.0629
rs11647188	A	0.0079	0.0014	-0.0143	0.0452
rs11652522	A	-0.0154	0.0023	-0.2832	0.0738
rs11657342	A	0.0140	0.0019	-0.2108	0.0458
rs11657979	A	-0.0091	0.0017	0.0449	0.0522
rs11663602	A	-0.0130	0.0016	-0.0399	0.0496
rs11663678	T	-0.0121	0.0022	0.1341	0.0681
rs116656374	T	-0.0242	0.0038	-0.0390	0.1157
rs11675476	T	-0.0127	0.0014	0.1318	0.0442
rs1167827	A	0.0104	0.0014	-0.2630	0.0446
rs11678980	A	-0.0166	0.0014	0.1217	0.0458
rs11687736	A	-0.0105	0.0015	-0.1214	0.0474
rs11693885	A	-0.0091	0.0014	0.0204	0.0446

rs116967397	A	0.0315	0.0056	-0.2708	0.1686
rs117005905	T	0.0150	0.0022	-0.0204	0.0719
rs11703948	A	-0.0168	0.0024	0.1094	0.0726
rs1171040	A	-0.0113	0.0017	0.0728	0.0546
rs11716398	A	0.0159	0.0021	-0.1567	0.0660
rs11720093	T	0.0150	0.0026	0.0006	0.0807
rs11720985	T	0.0085	0.0015	-0.0300	0.0479
rs11724690	T	0.0093	0.0015	0.0033	0.0494
rs117273411	T	-0.0211	0.0034	0.1545	0.1111
rs11732160	A	0.0105	0.0016	-0.0739	0.0504
rs11732657	A	-0.0126	0.0016	-0.0588	0.0506
rs11733439	A	-0.0096	0.0017	0.1345	0.0548
rs11736863	A	0.0142	0.0018	-0.0198	0.0574
rs117398064	C	-0.0141	0.0025	-0.0412	0.0753
rs117468730	A	-0.0310	0.0050	-0.2872	0.1565
rs117623407	A	-0.0118	0.0020	0.2018	0.0624
rs11765387	T	0.0086	0.0015	0.0553	0.0471
rs117668569	T	0.0234	0.0042	0.0391	0.1346
rs117677995	A	-0.0180	0.0032	0.2530	0.1001
rs11772232	T	0.0177	0.0019	-0.0771	0.0609
rs11774212	T	0.0128	0.0014	-0.0260	0.0443
rs117799466	C	0.0106	0.0016	-0.0442	0.0483
rs11780023	T	0.0085	0.0014	0.1344	0.0442
rs11789013	T	0.0106	0.0016	-0.0170	0.0519
rs117895796	T	0.0362	0.0064	-0.1861	0.2169
rs118040169	A	-0.0248	0.0038	0.0451	0.1183
rs118093058	T	0.0146	0.0021	-0.1332	0.0640
rs118134876	T	-0.0223	0.0030	0.4038	0.0928
rs11845781	T	-0.0079	0.0014	-0.0347	0.0445
rs11871429	A	0.0128	0.0017	0.2242	0.0527
rs11876620	T	0.0143	0.0024	0.0733	0.0739
rs11894424	A	0.0209	0.0033	0.0340	0.1072
rs11917701	A	-0.0087	0.0014	0.0501	0.0453
rs11919835	C	0.0089	0.0015	-0.1669	0.0459
rs1196760	C	0.0139	0.0025	-0.2964	0.0760
rs12005151	A	0.0087	0.0015	-0.0459	0.0466
rs12028010	T	0.0164	0.0017	0.1085	0.0524
rs12028229	T	0.0099	0.0016	-0.0008	0.0505
rs12054166	C	0.0096	0.0016	-0.1526	0.0507
rs12076635	C	0.0207	0.0017	0.0879	0.0535
rs12113634	T	0.0088	0.0015	-0.0521	0.0463
rs12123293	T	0.0086	0.0015	0.0069	0.0460
rs12126231	A	0.0095	0.0014	-0.2249	0.0450
rs12127928	T	0.0108	0.0018	0.1129	0.0554
rs12145078	A	-0.0127	0.0017	0.0137	0.0552
rs12151248	T	-0.0149	0.0023	-0.0953	0.0688

rs12170452	A	0.0105	0.0014	-0.0316	0.0445
rs1220779	A	-0.0079	0.0014	0.0220	0.0442
rs12234369	A	0.0087	0.0015	-0.0205	0.0479
rs12238011	T	-0.0141	0.0026	0.0706	0.0803
rs12273435	A	-0.0132	0.0017	0.1307	0.0546
rs12285074	A	0.0161	0.0023	0.3027	0.0772
rs12304188	A	0.0111	0.0019	-0.0315	0.0617
rs12325727	A	0.0088	0.0014	-0.1377	0.0443
rs12332731	A	0.0135	0.0018	0.0566	0.0554
rs12342546	A	-0.0128	0.0017	0.0201	0.0522
rs12359372	T	-0.0092	0.0015	0.0698	0.0473
rs12375949	T	-0.0138	0.0014	0.0686	0.0445
rs12405889	T	-0.0098	0.0014	0.0328	0.0440
rs12431682	T	-0.0093	0.0015	0.0124	0.0459
rs12438177	A	0.0093	0.0015	-0.0783	0.0459
rs12453682	T	0.0102	0.0015	0.0471	0.0483
rs1245829	A	-0.0099	0.0014	0.2447	0.0447
rs12467175	T	-0.0081	0.0014	0.0752	0.0442
rs12468040	T	0.0137	0.0014	-0.1166	0.0455
rs12473986	T	-0.0085	0.0015	0.1137	0.0487
rs12477385	T	0.0109	0.0017	-0.0171	0.0528
rs12478156	T	0.0108	0.0015	0.0611	0.0463
rs12503522	T	-0.0113	0.0016	0.0315	0.0489
rs12506221	T	-0.0130	0.0014	0.0966	0.0448
rs12516990	C	-0.0099	0.0017	0.0613	0.0547
rs12519073	T	-0.0107	0.0017	0.0375	0.0530
rs12524795	T	0.0102	0.0014	-0.0163	0.0444
rs12568153	T	-0.0091	0.0015	0.1598	0.0488
rs12571549	A	0.0151	0.0020	-0.0086	0.0644
rs12574281	A	-0.0080	0.0015	-0.0456	0.0456
rs12591647	T	-0.0152	0.0018	0.0515	0.0578
rs12601380	A	-0.0082	0.0014	0.1563	0.0447
rs12602286	T	0.0160	0.0021	0.0653	0.0668
rs12613500	C	0.0098	0.0014	0.0076	0.0448
rs12614263	A	0.0083	0.0014	-0.0323	0.0443
rs12638072	A	-0.0107	0.0015	-0.1098	0.0491
rs12643771	T	0.0130	0.0015	-0.0068	0.0479
rs12646216	T	0.0084	0.0014	-0.1530	0.0454
rs12646523	T	-0.0132	0.0016	0.0686	0.0508
rs12659776	T	0.0085	0.0014	-0.1029	0.0444
rs12670376	A	0.0092	0.0014	-0.0962	0.0444
rs1267062	C	0.0113	0.0018	0.0835	0.0578
rs12682775	T	-0.0119	0.0017	0.1087	0.0537
rs12694681	T	0.0102	0.0015	-0.1316	0.0476
rs12709186	A	-0.0091	0.0015	0.0050	0.0468
rs12712269	T	-0.0107	0.0014	0.1305	0.0455

rs12746551	C	0.0248	0.0041	0.0941	0.1251
rs12750688	C	0.0116	0.0016	-0.0707	0.0496
rs12761729	T	-0.0098	0.0016	-0.0036	0.0509
rs12761761	T	0.0153	0.0017	-0.0578	0.0508
rs12764593	C	0.0202	0.0029	-0.1161	0.0952
rs12765185	A	-0.0088	0.0016	0.0837	0.0498
rs12774577	T	0.0142	0.0020	-0.1128	0.0630
rs12789313	T	0.0092	0.0014	-0.1019	0.0441
rs12790196	T	-0.0101	0.0015	-0.2739	0.0470
rs12810587	T	0.0094	0.0015	-0.0637	0.0476
rs12875339	A	-0.0108	0.0015	-0.0828	0.0463
rs12888615	T	0.0120	0.0018	0.1015	0.0552
rs12891042	T	0.0092	0.0014	-0.0625	0.0453
rs12891191	C	0.0080	0.0014	-0.0685	0.0443
rs12912465	T	-0.0104	0.0016	0.0435	0.0508
rs12952191	T	-0.0081	0.0014	0.1622	0.0442
rs12953422	A	-0.0080	0.0014	0.0330	0.0449
rs12957463	A	0.0146	0.0018	-0.0001	0.0550
rs12962845	C	-0.0123	0.0021	-0.0356	0.0651
rs12967010	T	0.0114	0.0017	0.0300	0.0534
rs12970264	A	-0.0085	0.0014	0.0413	0.0445
rs12981405	T	-0.0112	0.0019	-0.0259	0.0594
rs13009915	T	0.0212	0.0037	-0.0801	0.1148
rs13010288	T	0.0198	0.0021	-0.0054	0.0647
rs13010566	A	-0.0091	0.0014	0.1162	0.0451
rs13015496	C	0.0119	0.0018	-0.1061	0.0568
rs13016316	A	-0.0138	0.0024	-0.2364	0.0763
rs1301838	T	-0.0096	0.0015	0.0598	0.0470
rs13018640	T	-0.0215	0.0014	0.0231	0.0450
rs13026611	A	-0.0084	0.0015	0.1297	0.0463
rs13029602	T	-0.0092	0.0014	0.0780	0.0453
rs13034349	T	-0.0082	0.0014	0.0469	0.0451
rs13050131	A	-0.0081	0.0015	-0.0386	0.0474
rs13085461	C	0.0103	0.0014	-0.1156	0.0446
rs13099165	T	-0.0118	0.0017	0.0368	0.0548
rs13107325	T	-0.0239	0.0027	-0.6251	0.0841
rs13117856	A	0.0084	0.0015	-0.0593	0.0489
rs13130765	C	-0.0091	0.0014	0.0644	0.0442
rs13133213	A	0.0096	0.0014	-0.0596	0.0441
rs13145650	T	-0.0173	0.0025	-0.0322	0.0786
rs13154429	C	-0.0136	0.0023	0.0872	0.0704
rs13163062	T	0.0115	0.0014	-0.0055	0.0447
rs13163845	T	-0.0149	0.0020	0.1133	0.0630
rs13168136	A	-0.0092	0.0017	0.1000	0.0525
rs13169187	A	-0.0079	0.0014	0.1781	0.0443
rs13177031	A	-0.0085	0.0015	0.0255	0.0461

rs13190235	T	-0.0138	0.0024	0.1305	0.0760
rs13197257	T	0.0109	0.0016	-0.0353	0.0500
rs1320139	C	-0.0149	0.0014	0.0513	0.0446
rs13212041	T	0.0101	0.0018	-0.0382	0.0546
rs13240401	T	0.0176	0.0017	-0.0056	0.0530
rs13246220	T	-0.0093	0.0014	-0.0518	0.0453
rs13261773	C	0.0122	0.0019	0.0346	0.0619
rs13266287	A	-0.0085	0.0015	0.0308	0.0472
rs13281564	A	0.0088	0.0014	-0.0218	0.0443
rs13284516	C	-0.0082	0.0014	0.0070	0.0443
rs1329044	T	0.0134	0.0019	-0.0356	0.0579
rs1329125	T	-0.0097	0.0015	0.0761	0.0472
rs13296345	T	0.0090	0.0015	-0.1369	0.0460
rs13318986	A	0.0124	0.0015	0.0393	0.0466
rs13327482	A	-0.0121	0.0018	-0.0052	0.0584
rs1334297	A	0.0257	0.0016	0.0467	0.0503
rs1335482	T	0.0096	0.0014	0.0275	0.0443
rs13361043	T	-0.0158	0.0028	-0.0030	0.0892
rs13381557	A	-0.0087	0.0014	0.1282	0.0445
rs13388333	A	0.0085	0.0015	-0.1161	0.0457
rs13397529	C	-0.0097	0.0017	0.0281	0.0522
rs13398860	A	0.0086	0.0015	-0.0060	0.0463
rs13402497	A	-0.0080	0.0014	0.0778	0.0442
rs13422673	T	-0.0110	0.0014	0.0927	0.0443
rs13425585	C	-0.0089	0.0014	0.0025	0.0443
rs1368250	T	0.0250	0.0045	0.0328	0.1276
rs137079	T	0.0128	0.0020	0.0188	0.0632
rs137858393	A	0.0193	0.0029	0.2457	0.0886
rs138096147	A	0.0234	0.0042	0.0837	0.1312
rs138484388	T	-0.0306	0.0051	-0.2900	0.1504
rs1391513	T	-0.0095	0.0015	0.0262	0.0485
rs139244147	A	-0.0193	0.0030	0.1445	0.0973
rs1392816	T	0.0082	0.0014	0.0162	0.0456
rs139980871	T	0.0180	0.0029	-0.1954	0.0888
rs1404549	A	-0.0089	0.0015	-0.0131	0.0474
rs1405876	T	0.0109	0.0015	0.0327	0.0461
rs140711597	C	0.0366	0.0055	-0.4791	0.1548
rs1408284	C	-0.0127	0.0020	-0.0562	0.0646
rs1408430	C	0.0096	0.0016	0.0129	0.0492
rs141729694	T	0.0195	0.0026	0.2035	0.0855
rs142014757	A	0.0102	0.0018	0.0216	0.0552
rs1426619	T	0.0096	0.0014	0.0120	0.0447
rs1427298	T	0.0086	0.0014	-0.0731	0.0451
rs142747148	A	0.0329	0.0052	0.1229	0.1674
rs1427829	A	-0.0083	0.0014	-0.1141	0.0444
rs143148393	T	-0.0211	0.0039	-0.0203	0.1316

rs1434630	T	-0.0137	0.0020	0.0530	0.0616
rs143743568	A	0.0118	0.0020	-0.0589	0.0616
rs143812851	A	-0.0113	0.0019	0.1158	0.0583
rs1440930	C	0.0081	0.0014	0.0094	0.0446
rs144336753	A	0.0388	0.0053	-0.3430	0.1594
rs1445591	A	0.0085	0.0015	-0.0256	0.0486
rs1464297	T	-0.0109	0.0015	-0.0096	0.0466
rs1467737	T	-0.0084	0.0014	-0.0034	0.0446
rs1485300	C	0.0098	0.0015	0.0626	0.0472
rs1490612	T	0.0193	0.0026	-0.1830	0.0840
rs150252215	A	0.0204	0.0035	-0.3593	0.1116
rs150421637	A	0.0349	0.0061	-0.3692	0.1823
rs150537577	A	-0.0151	0.0026	0.1759	0.0848
rs1505676	C	0.0084	0.0015	0.1290	0.0456
rs151381	T	-0.0087	0.0014	-0.0257	0.0441
rs1518890	T	-0.0090	0.0016	0.1485	0.0503
rs1527878	A	-0.0105	0.0016	0.1536	0.0517
rs1529597	A	0.0223	0.0038	0.0154	0.1304
rs1538389	T	-0.0109	0.0018	-0.0022	0.0559
rs1542354	A	-0.0084	0.0014	-0.0718	0.0446
rs1544	A	-0.0096	0.0016	0.0688	0.0501
rs1554798	A	-0.0080	0.0014	-0.0072	0.0454
rs1564347	T	0.0088	0.0015	0.0413	0.0466
rs1569092	A	0.0160	0.0020	-0.0940	0.0608
rs1593022	T	-0.0099	0.0017	-0.0322	0.0519
rs1603460	T	0.0090	0.0014	-0.1323	0.0448
rs162445	A	0.0152	0.0026	0.0697	0.0832
rs163229	C	-0.0260	0.0047	0.0712	0.1483
rs1637770	T	0.0191	0.0028	-0.1866	0.0919
rs164938	T	-0.0088	0.0014	0.0787	0.0453
rs165633	A	-0.0116	0.0016	-0.0467	0.0531
rs1656614	C	0.0122	0.0015	-0.1916	0.0482
rs1671269	T	-0.0100	0.0016	-0.0102	0.0523
rs1671770	A	0.0125	0.0018	-0.0571	0.0589
rs16851779	T	-0.0153	0.0027	-0.0166	0.0804
rs16871807	T	0.0090	0.0015	-0.0100	0.0467
rs1689510	C	0.0194	0.0015	-0.1921	0.0466
rs16901689	T	0.0111	0.0019	-0.1317	0.0597
rs1693584	T	-0.0087	0.0015	0.1117	0.0479
rs16958559	T	0.0096	0.0017	-0.1098	0.0528
rs16966271	T	0.0092	0.0016	-0.0971	0.0494
rs16975275	A	-0.0102	0.0017	-0.0081	0.0523
rs17048801	A	-0.0106	0.0014	-0.0080	0.0461
rs17048855	A	0.0108	0.0015	-0.0095	0.0463
rs17069646	T	0.0085	0.0015	-0.0231	0.0478
rs17110109	T	-0.0093	0.0014	0.0493	0.0453

rs17113730	A	-0.0170	0.0027	-0.0737	0.0869
rs17131123	A	-0.0243	0.0039	0.0299	0.1123
rs17133297	A	-0.0146	0.0023	0.0811	0.0761
rs17144467	A	0.0098	0.0015	-0.0951	0.0476
rs17148998	A	0.0101	0.0017	-0.1533	0.0545
rs171697	C	0.0133	0.0015	0.1485	0.0470
rs17170519	C	0.0132	0.0023	0.0051	0.0719
rs1717204	A	-0.0117	0.0018	0.0545	0.0567
rs1718188	A	0.0096	0.0014	0.1476	0.0446
rs17186106	T	0.0108	0.0018	-0.1258	0.0580
rs17190418	T	-0.0213	0.0031	0.0034	0.0938
rs17205908	T	-0.0092	0.0015	-0.0103	0.0465
rs17248751	A	-0.0136	0.0017	0.0791	0.0528
rs1728118	A	0.0089	0.0016	-0.0617	0.0496
rs1729412	T	-0.0107	0.0014	0.1045	0.0448
rs173003	A	-0.0082	0.0014	-0.0291	0.0443
rs1734370	A	0.0099	0.0016	-0.0226	0.0486
rs1738050	C	-0.0093	0.0014	-0.0241	0.0454
rs17411339	A	0.0140	0.0014	-0.0735	0.0459
rs17428076	C	0.0137	0.0016	-0.0841	0.0515
rs17440885	A	0.0160	0.0029	0.0289	0.0907
rs1747714	T	0.0106	0.0014	0.0123	0.0444
rs1747817	T	-0.0092	0.0016	-0.0563	0.0514
rs17489649	A	0.0127	0.0015	-0.0135	0.0468
rs17502934	T	-0.0167	0.0020	0.0277	0.0631
rs175325	A	-0.0100	0.0014	-0.0084	0.0454
rs17536059	C	-0.0108	0.0019	0.1281	0.0578
rs17551064	A	0.0141	0.0019	-0.0641	0.0596
rs17563464	A	-0.0147	0.0017	0.1319	0.0538
rs17565975	A	-0.0106	0.0014	-0.1532	0.0448
rs17568389	A	0.0127	0.0014	-0.0617	0.0443
rs17574007	A	-0.0129	0.0021	0.0481	0.0685
rs1758747	A	0.0092	0.0015	-0.0602	0.0479
rs17598675	T	-0.0116	0.0014	0.0829	0.0445
rs17604349	A	0.0127	0.0018	-0.1064	0.0581
rs17609255	T	-0.0087	0.0014	0.1060	0.0448
rs176218	T	0.0200	0.0018	0.0685	0.0566
rs17622379	T	-0.0113	0.0018	0.0490	0.0562
rs17650634	T	-0.0104	0.0019	-0.0544	0.0587
rs17667540	A	-0.0087	0.0015	0.0816	0.0481
rs17669337	T	-0.0110	0.0014	-0.0272	0.0447
rs17680712	T	-0.0092	0.0014	0.0783	0.0446
rs17686649	T	-0.0098	0.0017	-0.0879	0.0537
rs17732878	T	-0.0095	0.0017	0.1779	0.0539
rs17742342	A	-0.0119	0.0018	0.2674	0.0553
rs17747544	A	-0.0090	0.0014	0.1002	0.0442

rs1779549	A	0.0081	0.0014	-0.0334	0.0449
rs178183	T	0.0145	0.0016	-0.0815	0.0511
rs17882802	A	0.0084	0.0014	0.0440	0.0444
rs17883331	A	-0.0108	0.0018	0.1992	0.0556
rs1792602	A	-0.0097	0.0014	0.0117	0.0450
rs181214	T	-0.0104	0.0017	0.1669	0.0543
rs182355396	A	-0.0525	0.0084	-0.2252	0.2589
rs182902112	A	-0.0300	0.0053	0.0453	0.1663
rs1835339	T	0.0084	0.0014	0.0346	0.0455
rs183869217	C	0.0174	0.0030	0.0262	0.0876
rs1841023	A	-0.0092	0.0015	0.0085	0.0478
rs1842713	A	-0.0117	0.0017	0.0034	0.0540
rs1843815	A	0.0077	0.0014	0.0595	0.0443
rs1861786	A	-0.0081	0.0014	0.0264	0.0454
rs186456786	A	0.0455	0.0079	-0.2867	0.2313
rs1865955	T	0.0122	0.0019	0.0053	0.0582
rs1866823	A	0.0107	0.0014	-0.0744	0.0448
rs187580	T	-0.0104	0.0017	0.0553	0.0518
rs187951956	A	-0.0315	0.0058	0.1045	0.1952
rs1880088	A	-0.0097	0.0016	-0.0196	0.0509
rs1880692	A	0.0086	0.0014	-0.0584	0.0444
rs188251563	A	0.0428	0.0070	0.2065	0.2256
rs1890132	T	-0.0089	0.0015	-0.0717	0.0484
rs1898111	A	0.0101	0.0018	-0.0572	0.0581
rs190102446	T	-0.0210	0.0037	-0.1061	0.1211
rs1905616	A	0.0083	0.0015	0.1076	0.0470
rs1910005	T	-0.0090	0.0015	-0.0481	0.0486
rs1918394	T	0.0119	0.0019	0.0513	0.0596
rs191903670	A	0.0335	0.0061	-0.1809	0.2023
rs192436652	T	-0.0319	0.0045	0.0699	0.1355
rs1933264	T	0.0100	0.0016	0.1101	0.0513
rs1952183	A	-0.0089	0.0014	-0.0619	0.0442
rs1955250	A	-0.0140	0.0025	0.0678	0.0806
rs1963381	A	0.0099	0.0016	-0.1165	0.0517
rs1980129	A	0.0085	0.0014	-0.0041	0.0442
rs198262	T	-0.0223	0.0035	0.1961	0.1097
rs1991585	T	-0.0101	0.0015	0.1295	0.0485
rs1995181	A	-0.0078	0.0014	-0.1169	0.0443
rs2007655	T	0.0086	0.0014	-0.0387	0.0442
rs2011603	A	-0.0091	0.0016	0.3345	0.0502
rs2014830	T	0.0110	0.0015	-0.2347	0.0484
rs2023016	C	0.0107	0.0017	-0.0468	0.0538
rs2024568	T	-0.0097	0.0016	-0.0695	0.0515
rs2029401	A	-0.0091	0.0014	0.0047	0.0450
rs2034631	T	0.0089	0.0015	0.0853	0.0465
rs2039204	A	0.0082	0.0014	-0.1387	0.0443

rs2043187	A	0.0107	0.0015	0.0044	0.0489
rs2052285	A	0.0112	0.0014	-0.0030	0.0453
rs2055940	A	0.0082	0.0015	-0.0353	0.0472
rs2077235	T	0.0097	0.0017	0.0124	0.0522
rs2081652	A	0.0123	0.0015	-0.0670	0.0466
rs2082317	T	-0.0078	0.0014	0.0101	0.0451
rs2088913	A	0.0081	0.0014	0.0224	0.0447
rs2092248	T	-0.0083	0.0015	-0.0471	0.0484
rs2098526	A	-0.0241	0.0043	0.0717	0.1352
rs2100249	T	-0.0089	0.0015	0.0301	0.0468
rs2126069	T	0.0080	0.0014	0.0502	0.0445
rs2131167	A	0.0080	0.0014	-0.0110	0.0445
rs214626	A	0.0101	0.0018	0.0448	0.0571
rs2160514	A	-0.0094	0.0014	-0.0090	0.0452
rs2179152	T	-0.0131	0.0015	-0.1236	0.0455
rs2183271	T	0.0084	0.0015	-0.1625	0.0461
rs2199409	T	0.0103	0.0017	-0.0017	0.0552
rs2212430	T	-0.0100	0.0016	0.0134	0.0484
rs2216144	T	0.0098	0.0014	-0.0167	0.0444
rs2220926	T	-0.0095	0.0014	-0.0636	0.0448
rs2250660	C	0.0080	0.0014	0.0227	0.0442
rs2252098	T	-0.0077	0.0014	-0.0695	0.0443
rs2254681	A	0.0101	0.0016	0.0864	0.0505
rs2256965	A	0.0106	0.0014	-0.1265	0.0449
rs2276209	A	0.0085	0.0015	0.0562	0.0488
rs2283076	A	0.0115	0.0017	-0.0077	0.0527
rs2287838	A	-0.0104	0.0014	-0.0413	0.0445
rs2290601	T	-0.0149	0.0017	0.0510	0.0525
rs2297293	C	0.0099	0.0015	-0.0733	0.0481
rs2299156	T	0.0099	0.0018	-0.0999	0.0584
rs2314338	T	0.0093	0.0016	-0.0152	0.0498
rs2321157	A	-0.0081	0.0014	-0.0933	0.0445
rs232496	T	0.0090	0.0015	0.0862	0.0462
rs2332179	A	0.0121	0.0020	0.1331	0.0652
rs2336721	T	0.0095	0.0015	-0.1737	0.0469
rs2358628	A	-0.0091	0.0015	-0.0249	0.0478
rs236318	C	-0.0102	0.0017	0.0095	0.0522
rs2364544	A	-0.0105	0.0014	-0.0021	0.0454
rs2365376	A	0.0092	0.0015	-0.0685	0.0471
rs2368831	T	-0.0092	0.0014	0.1108	0.0451
rs2373124	A	-0.0096	0.0016	-0.1531	0.0520
rs2406253	A	0.0135	0.0018	-0.1352	0.0556
rs2414072	A	-0.0083	0.0014	-0.2248	0.0445
rs2416214	A	0.0079	0.0014	0.0060	0.0447
rs2416759	A	-0.0093	0.0015	-0.1499	0.0483
rs2416845	T	0.0263	0.0048	-0.1896	0.1529

rs2431023	A	0.0093	0.0014	0.0408	0.0447
rs2434672	A	0.0091	0.0014	-0.0018	0.0448
rs2436760	T	0.0232	0.0042	-0.0837	0.1271
rs2447097	T	0.0096	0.0014	0.0345	0.0446
rs2458370	T	-0.0086	0.0015	0.1403	0.0463
rs2469226	A	0.0096	0.0017	0.0369	0.0531
rs2470966	C	-0.0103	0.0015	0.0683	0.0479
rs2478208	C	-0.0103	0.0014	0.0086	0.0443
rs2496482	T	0.0121	0.0015	-0.0429	0.0452
rs2517086	C	-0.0088	0.0014	0.0061	0.0450
rs2521602	A	-0.0266	0.0049	-0.1953	0.1509
rs2522545	T	-0.0083	0.0014	0.1148	0.0450
rs2526398	C	-0.0222	0.0015	0.0856	0.0462
rs2529069	T	-0.0106	0.0016	0.0820	0.0509
rs252991	A	0.0095	0.0015	-0.0703	0.0458
rs2542673	A	0.0088	0.0015	0.0134	0.0472
rs2545795	A	0.0125	0.0014	0.0245	0.0446
rs255053	A	-0.0111	0.0018	-0.0681	0.0565
rs2554835	A	0.0088	0.0014	-0.0193	0.0453
rs2568955	T	-0.0165	0.0016	0.0351	0.0534
rs2569041	T	0.0077	0.0014	-0.0984	0.0451
rs2570497	T	-0.0121	0.0015	-0.0756	0.0462
rs2588959	T	-0.0080	0.0014	0.0466	0.0442
rs2657283	T	0.0085	0.0014	-0.1156	0.0449
rs2665668	A	-0.0094	0.0015	-0.0426	0.0466
rs2702576	A	-0.0090	0.0014	0.1262	0.0456
rs2718277	T	0.0142	0.0026	-0.1554	0.0782
rs2718791	T	-0.0081	0.0015	-0.0372	0.0462
rs27220	A	-0.0114	0.0015	0.0181	0.0460
rs2725370	T	-0.0141	0.0015	0.1240	0.0482
rs2736752	T	0.0095	0.0017	-0.1088	0.0542
rs2740795	A	0.0098	0.0016	0.1492	0.0503
rs2761438	A	0.0098	0.0015	-0.0167	0.0455
rs2764684	T	0.0143	0.0019	-0.0428	0.0577
rs2805064	C	0.0093	0.0016	0.0227	0.0493
rs2838006	T	0.0132	0.0015	-0.0361	0.0463
rs28482086	A	0.0086	0.0015	-0.0731	0.0467
rs28505285	C	0.0106	0.0018	-0.0099	0.0573
rs28512462	C	0.0112	0.0014	-0.2510	0.0458
rs28513882	A	-0.0110	0.0018	-0.0768	0.0570
rs2852349	T	-0.0093	0.0014	-0.0042	0.0444
rs28587776	T	0.0088	0.0015	-0.1027	0.0460
rs28669886	A	-0.0084	0.0015	-0.0345	0.0463
rs2870281	A	-0.0083	0.0014	0.0529	0.0445
rs28735993	A	0.0162	0.0022	-0.0615	0.0690
rs2885198	A	0.0088	0.0014	-0.0557	0.0446

rs2898191	A	0.0093	0.0015	-0.1006	0.0476
rs2910823	T	0.0115	0.0014	-0.0744	0.0449
rs2916490	A	-0.0092	0.0015	0.1374	0.0487
rs2923424	A	0.0120	0.0014	-0.1555	0.0452
rs2926702	T	-0.0124	0.0021	0.1792	0.0667
rs2929032	A	-0.0081	0.0014	0.0505	0.0443
rs2929860	T	0.0110	0.0018	0.0285	0.0556
rs2942884	A	-0.0090	0.0014	0.1015	0.0444
rs2954114	A	-0.0081	0.0014	0.0047	0.0444
rs2958182	A	0.0082	0.0015	0.0301	0.0470
rs2962378	A	0.0101	0.0017	-0.0780	0.0536
rs2964199	T	-0.0099	0.0015	-0.0175	0.0479
rs2964255	A	0.0088	0.0015	0.1047	0.0479
rs2976397	T	0.0081	0.0014	0.0158	0.0448
rs2977464	T	0.0102	0.0018	-0.1001	0.0567
rs29792	A	-0.0093	0.0015	0.1476	0.0482
rs2980813	A	0.0088	0.0014	-0.0822	0.0445
rs2989476	C	0.0078	0.0014	0.0127	0.0446
rs2989751	A	-0.0092	0.0017	-0.0121	0.0521
rs2992037	A	0.0149	0.0015	0.1665	0.0478
rs2994326	T	-0.0100	0.0018	-0.0758	0.0572
rs2998299	T	-0.0136	0.0017	-0.0206	0.0542
rs3026996	A	0.0138	0.0016	0.1277	0.0515
rs303752	A	-0.0110	0.0014	0.1068	0.0455
rs3111251	T	-0.0090	0.0014	-0.0350	0.0447
rs312927	A	-0.0147	0.0022	-0.0902	0.0688
rs312945	A	-0.0091	0.0015	-0.0811	0.0483
rs317050	T	0.0078	0.0014	-0.0294	0.0442
rs322614	A	-0.0087	0.0014	0.0037	0.0454
rs322744	T	-0.0090	0.0016	0.0415	0.0502
rs324885	A	0.0142	0.0014	-0.0298	0.0443
rs32940	T	-0.0096	0.0015	0.0790	0.0483
rs339057	A	-0.0081	0.0014	0.0940	0.0447
rs34067381	T	-0.0104	0.0015	-0.0154	0.0463
rs34098770	A	-0.0160	0.0020	0.1059	0.0615
rs34106693	C	0.0159	0.0019	-0.0557	0.0599
rs34122915	C	0.0084	0.0014	-0.0580	0.0457
rs341504	A	0.0099	0.0015	0.0477	0.0462
rs34155847	A	0.0135	0.0016	-0.1774	0.0516
rs34262657	T	-0.0240	0.0038	-0.1359	0.1274
rs34286836	T	-0.0089	0.0014	-0.1135	0.0446
rs34298584	A	0.0111	0.0019	-0.0748	0.0590
rs34305371	A	0.0314	0.0024	-0.1364	0.0730
rs34309	A	0.0080	0.0014	-0.0102	0.0457
rs34316274	A	0.0097	0.0017	0.1355	0.0517
rs34363861	A	0.0087	0.0014	-0.2093	0.0453

rs34394051	A	-0.0117	0.0020	0.1590	0.0606
rs34410	C	-0.0083	0.0014	0.0875	0.0443
rs34624793	T	0.0116	0.0021	-0.0045	0.0641
rs34720381	T	-0.0180	0.0024	0.2131	0.0764
rs34748029	A	0.0185	0.0025	-0.0060	0.0768
rs347661	T	-0.0081	0.0014	0.1000	0.0450
rs34780702	A	0.0111	0.0017	-0.0300	0.0518
rs34807077	A	0.0128	0.0019	-0.0460	0.0598
rs34811474	A	0.0104	0.0017	-0.2049	0.0523
rs34967082	A	-0.0080	0.0014	0.1451	0.0449
rs35016816	T	0.0176	0.0023	0.1088	0.0741
rs350281	T	-0.0117	0.0015	0.0945	0.0471
rs35104491	A	0.0124	0.0018	0.0190	0.0564
rs35111506	C	-0.0175	0.0030	0.1860	0.0954
rs35192107	T	-0.0091	0.0017	0.0509	0.0518
rs35319653	T	0.0119	0.0015	-0.1378	0.0465
rs35417702	T	-0.0152	0.0014	0.0479	0.0442
rs35493937	C	0.0124	0.0017	0.0426	0.0530
rs35606437	A	0.0105	0.0016	-0.0005	0.0501
rs356999	A	-0.0101	0.0014	-0.0168	0.0456
rs35745455	A	0.0080	0.0014	-0.0311	0.0441
rs35751693	T	-0.0246	0.0040	0.4313	0.1167
rs35754740	T	0.0083	0.0014	-0.0872	0.0449
rs35811586	T	0.0217	0.0031	0.0811	0.0951
rs35929923	A	-0.0114	0.0016	0.0499	0.0508
rs36085856	C	0.0201	0.0036	-0.1558	0.0882
rs36120534	T	0.0100	0.0018	-0.0322	0.0567
rs362307	T	-0.0226	0.0027	0.2642	0.0843
rs363096	T	-0.0143	0.0014	0.1230	0.0447
rs3735478	T	0.0108	0.0016	-0.1233	0.0486
rs3751331	A	-0.0097	0.0014	-0.0574	0.0457
rs3766979	A	0.0085	0.0015	-0.0932	0.0471
rs3768480	C	0.0101	0.0014	-0.1056	0.0443
rs3781339	T	-0.0104	0.0018	-0.0337	0.0559
rs3790609	T	0.0107	0.0019	0.1439	0.0576
rs3809169	T	0.0140	0.0025	0.1836	0.0775
rs3809634	A	-0.0109	0.0015	0.0637	0.0480
rs3812281	T	0.0113	0.0014	-0.0564	0.0451
rs3817923	A	-0.0121	0.0022	0.0926	0.0690
rs382196	T	-0.0082	0.0014	-0.0421	0.0455
rs3847228	T	0.0083	0.0014	0.0049	0.0451
rs3848715	A	-0.0080	0.0014	0.0596	0.0443
rs3859523	T	0.0124	0.0018	-0.0850	0.0575
rs387027	A	-0.0083	0.0015	0.1790	0.0464
rs3895736	A	0.0144	0.0019	0.1848	0.0578
rs3897821	A	0.0154	0.0015	-0.0647	0.0468

rs3948495	T	-0.0089	0.0014	0.0485	0.0453
rs399821	A	-0.0078	0.0014	0.0034	0.0446
rs39998	A	0.0090	0.0015	0.0424	0.0489
rs401526	T	-0.0104	0.0014	-0.0131	0.0443
rs401966	C	-0.0097	0.0014	0.0389	0.0456
rs4127499	A	0.0087	0.0015	0.0270	0.0460
rs41282553	A	0.0237	0.0040	-0.0504	0.1250
rs42210	C	-0.0098	0.0016	-0.0054	0.0490
rs42302	A	0.0086	0.0015	-0.0562	0.0467
rs4255791	A	-0.0084	0.0015	0.0733	0.0469
rs4263475	A	0.0078	0.0014	-0.0777	0.0454
rs4283754	A	-0.0084	0.0014	-0.0218	0.0468
rs429150	T	0.0085	0.0014	-0.2606	0.0444
rs4298514	T	0.0104	0.0015	-0.0023	0.0483
rs4320563	A	0.0109	0.0014	-0.0416	0.0443
rs4328757	T	0.0096	0.0014	-0.0402	0.0454
rs4352658	T	-0.0190	0.0026	0.0531	0.0809
rs4358081	A	-0.0095	0.0014	-0.1201	0.0442
rs4358358	A	0.0109	0.0015	-0.0341	0.0480
rs4369924	A	0.0125	0.0019	-0.0931	0.0606
rs4384309	A	0.0102	0.0014	0.0009	0.0446
rs4396896	A	-0.0107	0.0014	0.0005	0.0453
rs4423373	A	-0.0087	0.0014	0.0315	0.0445
rs4426420	T	-0.0140	0.0019	0.0897	0.0606
rs4434676	A	-0.0089	0.0014	-0.0454	0.0446
rs4458044	C	0.0101	0.0016	0.1886	0.0514
rs4467547	T	0.0121	0.0014	0.0247	0.0449
rs4469771	T	0.0101	0.0015	-0.0132	0.0472
rs4480339	A	-0.0097	0.0015	-0.0546	0.0477
rs4490539	A	0.0142	0.0015	0.0726	0.0480
rs4497562	A	0.0112	0.0016	-0.0503	0.0508
rs4500930	T	-0.0109	0.0015	0.0721	0.0464
rs4500960	T	-0.0131	0.0014	0.0409	0.0443
rs4502401	T	0.0085	0.0015	-0.0434	0.0468
rs4652135	A	0.0103	0.0016	0.0711	0.0497
rs4658019	T	0.0084	0.0014	-0.1452	0.0443
rs4663617	A	0.0104	0.0017	-0.1114	0.0529
rs4664983	T	-0.0103	0.0018	0.1239	0.0558
rs4673840	T	-0.0138	0.0019	0.1415	0.0602
rs4675248	A	-0.0094	0.0014	-0.0281	0.0451
rs4685405	T	-0.0111	0.0018	0.0367	0.0587
rs4687735	T	-0.0200	0.0033	0.0345	0.1013
rs4691601	A	-0.0115	0.0014	-0.0028	0.0443
rs4705763	C	0.0089	0.0014	-0.1175	0.0443
rs4712371	A	-0.0124	0.0021	0.0376	0.0678
rs4719460	T	-0.0088	0.0015	0.0872	0.0467

rs4719944	T	-0.0121	0.0014	0.0248	0.0445
rs4724083	T	0.0083	0.0015	-0.0377	0.0479
rs4726070	A	0.0122	0.0014	-0.0637	0.0452
rs4730020	T	0.0090	0.0016	-0.0630	0.0500
rs4731413	A	0.0111	0.0017	-0.0913	0.0543
rs4735297	A	-0.0086	0.0015	-0.1124	0.0475
rs4739235	A	0.0109	0.0019	-0.0171	0.0596
rs4741571	A	-0.0102	0.0015	-0.0956	0.0477
rs4757957	C	0.0133	0.0015	0.1122	0.0474
rs4766424	C	0.0142	0.0021	-0.0325	0.0662
rs4778058	T	-0.0092	0.0014	-0.0370	0.0449
rs4785187	A	-0.0100	0.0017	-0.0842	0.0532
rs4787028	T	0.0086	0.0015	-0.0766	0.0467
rs4787457	A	0.0162	0.0015	-0.1380	0.0456
rs4788115	A	0.0111	0.0019	0.0085	0.0592
rs479018	A	0.0109	0.0015	-0.0189	0.0476
rs4793090	A	0.0084	0.0015	0.0353	0.0466
rs4810894	A	-0.0085	0.0015	0.0976	0.0459
rs4812697	T	0.0171	0.0027	0.1107	0.0849
rs481940	T	0.0100	0.0016	-0.0488	0.0500
rs482787	T	0.0100	0.0015	-0.0519	0.0471
rs4839155	T	0.0115	0.0017	-0.0093	0.0519
rs4846010	A	0.0099	0.0018	-0.1588	0.0554
rs4846724	A	0.0098	0.0014	-0.0470	0.0444
rs4848924	A	-0.0117	0.0015	0.0286	0.0492
rs4850954	T	0.0077	0.0014	-0.0052	0.0446
rs4851263	A	-0.0151	0.0023	0.0596	0.0725
rs4877516	A	-0.0101	0.0014	0.0003	0.0445
rs4881269	A	0.0100	0.0014	-0.0072	0.0456
rs488476	C	-0.0113	0.0015	0.0945	0.0455
rs4894658	C	0.0091	0.0016	-0.0806	0.0506
rs4895650	T	0.0093	0.0014	-0.0792	0.0449
rs4899012	C	-0.0103	0.0014	-0.0950	0.0453
rs4904523	A	-0.0082	0.0014	-0.0018	0.0442
rs4915735	A	0.0116	0.0020	-0.0579	0.0632
rs4919624	A	0.0193	0.0018	-0.1195	0.0546
rs4925065	T	-0.0078	0.0014	0.2212	0.0443
rs4925109	A	-0.0096	0.0015	0.1350	0.0483
rs4938815	T	0.0085	0.0015	-0.1291	0.0483
rs4941735	T	0.0092	0.0014	-0.1394	0.0447
rs4972748	T	0.0105	0.0018	-0.1418	0.0574
rs4977885	A	-0.0084	0.0014	-0.0047	0.0451
rs4981245	A	0.0080	0.0014	-0.1122	0.0447
rs4984541	A	-0.0138	0.0017	0.0941	0.0528
rs4984613	T	0.0174	0.0028	0.0119	0.0916
rs4984682	C	-0.0138	0.0017	-0.0256	0.0532

rs548897	A	0.0081	0.0014	-0.0712	0.0445
rs55675587	T	-0.0103	0.0018	0.0258	0.0563
rs55736314	C	-0.0154	0.0014	0.0776	0.0452
rs55826493	T	-0.0127	0.0021	-0.0440	0.0666
rs55986781	T	0.0187	0.0026	0.1182	0.0780
rs56085180	A	-0.0269	0.0037	0.1163	0.1151
rs56099375	T	0.0120	0.0017	-0.0108	0.0517
rs56171318	T	-0.0137	0.0020	0.0954	0.0633
rs56174996	A	-0.0143	0.0020	-0.0248	0.0649
rs56306882	A	0.0086	0.0016	-0.1570	0.0498
rs56319902	T	-0.0206	0.0017	-0.2734	0.0528
rs56391344	A	0.0148	0.0016	-0.0948	0.0508
rs563954	A	-0.0081	0.0014	0.0216	0.0443
rs56405138	A	0.0153	0.0022	0.0689	0.0676
rs56408528	T	-0.0082	0.0014	-0.0077	0.0443
rs567003	A	0.0094	0.0015	0.0430	0.0465
rs56794817	A	0.0122	0.0019	-0.1145	0.0608
rs57016874	T	0.0209	0.0037	-0.1524	0.1130
rs57148205	A	0.0095	0.0016	-0.1135	0.0523
rs57204268	A	0.0114	0.0020	-0.1395	0.0614
rs57349798	A	0.0093	0.0014	-0.0172	0.0455
rs57352738	A	-0.0152	0.0017	0.0922	0.0545
rs57437407	A	-0.0145	0.0023	-0.0849	0.0703
rs575113	A	0.0133	0.0015	0.0688	0.0488
rs5754581	T	-0.0082	0.0014	0.1115	0.0446
rs5754753	T	-0.0126	0.0016	-0.0103	0.0490
rs5754762	A	0.0197	0.0030	-0.1614	0.0978
rs57661533	T	-0.0114	0.0021	0.0176	0.0660
rs580652	T	-0.0147	0.0024	0.0377	0.0745
rs585557	A	0.0128	0.0018	-0.2410	0.0553
rs58779949	A	0.0110	0.0019	-0.0743	0.0573
rs58859557	T	0.0187	0.0028	0.1457	0.0864
rs58921703	T	0.0109	0.0015	0.0851	0.0466
rs58950082	T	0.0095	0.0017	-0.0722	0.0547
rs58996896	A	-0.0094	0.0016	-0.0404	0.0499
rs590013	T	0.0100	0.0015	0.0456	0.0473
rs59300999	T	-0.0164	0.0028	-0.0979	0.0890
rs59480703	C	-0.0123	0.0018	0.1603	0.0556
rs59813324	T	0.0097	0.0018	-0.0260	0.0556
rs59967356	T	-0.0127	0.0021	0.0778	0.0657
rs60096640	A	0.0159	0.0023	-0.2028	0.0729
rs6020560	T	-0.0083	0.0014	-0.0675	0.0443
rs6043521	T	-0.0083	0.0014	0.0538	0.0457
rs60589532	A	0.0168	0.0028	-0.1437	0.0933
rs6060308	A	0.0095	0.0016	0.0899	0.0505
rs6065080	T	-0.0130	0.0015	-0.0393	0.0463

rs6065784	C	0.0110	0.0015	0.0062	0.0489
rs60717745	C	-0.0118	0.0019	-0.0562	0.0612
rs60726488	T	-0.0090	0.0016	-0.0428	0.0503
rs6091570	A	0.0081	0.0015	-0.0257	0.0468
rs61104616	A	-0.0172	0.0014	-0.0867	0.0442
rs6123924	A	0.0129	0.0019	-0.0087	0.0614
rs613872	T	-0.0163	0.0019	-0.1163	0.0583
rs61387839	A	0.0088	0.0015	-0.0244	0.0476
rs61527214	A	0.0110	0.0014	-0.0485	0.0451
rs61739710	A	-0.0099	0.0016	-0.1245	0.0491
rs61748951	A	-0.0273	0.0048	0.2476	0.1474
rs61755388	T	0.0125	0.0017	-0.0475	0.0536
rs61757207	A	0.0359	0.0063	0.0887	0.1829
rs61798586	A	-0.0112	0.0020	-0.0076	0.0620
rs61853335	T	-0.0109	0.0018	0.0021	0.0581
rs61958175	A	-0.0198	0.0034	0.0525	0.1039
rs61997667	T	-0.0144	0.0020	0.0861	0.0613
rs62007304	A	0.0264	0.0047	-0.1417	0.1352
rs62018216	T	0.0113	0.0019	0.0309	0.0614
rs62051146	A	-0.0134	0.0022	-0.3401	0.0693
rs62090515	A	-0.0090	0.0015	0.0593	0.0461
rs62092949	T	-0.0087	0.0014	-0.0058	0.0445
rs62103198	T	-0.0147	0.0025	0.0375	0.0775
rs62109862	A	0.0136	0.0020	-0.0401	0.0642
rs62142891	A	0.0108	0.0016	-0.0566	0.0503
rs62155350	A	0.0338	0.0053	0.1493	0.1572
rs62155770	A	-0.0196	0.0032	0.0842	0.0951
rs62155873	T	-0.0151	0.0021	0.1928	0.0664
rs62174974	A	-0.0105	0.0018	0.1616	0.0556
rs62177359	A	-0.0275	0.0038	0.1214	0.1222
rs62179650	A	0.0112	0.0016	0.0011	0.0479
rs62182994	T	0.0137	0.0015	-0.0259	0.0474
rs62190914	T	0.0089	0.0015	-0.1062	0.0460
rs62194170	A	0.0111	0.0017	-0.1683	0.0552
rs622169	T	0.0086	0.0014	-0.0385	0.0462
rs62247449	C	0.0114	0.0014	0.0209	0.0446
rs62252819	A	-0.0094	0.0017	-0.0659	0.0521
rs62256284	T	0.0111	0.0017	0.1543	0.0542
rs62260764	C	-0.0132	0.0016	-0.1516	0.0501
rs62262671	A	0.0213	0.0020	-0.2061	0.0638
rs62340636	T	0.0103	0.0014	0.0248	0.0459
rs62370510	A	-0.0109	0.0019	0.0809	0.0578
rs62379838	T	0.0121	0.0015	0.1173	0.0481
rs62409395	T	0.0101	0.0018	-0.0551	0.0548
rs62420387	T	-0.0184	0.0027	0.3290	0.0836
rs62506074	T	0.0091	0.0015	-0.0737	0.0469

rs62506104	A	-0.0098	0.0018	0.1261	0.0567
rs628993	A	0.0128	0.0023	-0.0147	0.0753
rs631287	A	0.0084	0.0014	0.1939	0.0445
rs633279	A	-0.0089	0.0015	0.0533	0.0486
rs635754	A	0.0119	0.0014	0.0571	0.0450
rs6428587	T	0.0084	0.0015	-0.0559	0.0459
rs6435326	A	-0.0091	0.0014	0.1079	0.0444
rs6442126	A	-0.0121	0.0015	0.0687	0.0468
rs6445633	A	0.0088	0.0015	0.0351	0.0477
rs6449503	A	0.0185	0.0014	-0.0326	0.0445
rs6450476	A	-0.0116	0.0015	-0.1081	0.0486
rs6452793	T	0.0175	0.0017	-0.1754	0.0534
rs6461536	A	0.0088	0.0015	-0.0952	0.0489
rs6469654	C	-0.0103	0.0017	0.1858	0.0547
rs6472208	T	-0.0091	0.0014	0.0117	0.0451
rs6480234	T	-0.0084	0.0014	0.1402	0.0443
rs648044	A	0.0083	0.0015	0.0109	0.0463
rs6490618	T	0.0088	0.0015	-0.0278	0.0472
rs6493265	T	-0.0122	0.0014	0.0724	0.0452
rs6493275	A	0.0131	0.0017	-0.0215	0.0541
rs6503409	T	0.0087	0.0015	0.0500	0.0464
rs6534338	T	0.0103	0.0015	-0.0199	0.0485
rs6543810	T	0.0082	0.0015	-0.0251	0.0454
rs6557171	T	-0.0155	0.0015	0.0818	0.0466
rs6567288	A	-0.0079	0.0014	0.1406	0.0448
rs6569077	T	0.0118	0.0014	-0.0673	0.0473
rs6573552	T	-0.0090	0.0014	0.0486	0.0442
rs6573559	T	0.0105	0.0015	0.0043	0.0476
rs660001	A	-0.0164	0.0017	0.1957	0.0546
rs663251	T	0.0081	0.0015	-0.1603	0.0465
rs66482320	C	0.0124	0.0022	-0.0232	0.0705
rs66568921	T	-0.0181	0.0015	-0.0472	0.0465
rs66671632	T	-0.0133	0.0021	-0.1108	0.0665
rs66721975	A	-0.0087	0.0015	0.2263	0.0492
rs6672986	C	0.0094	0.0014	-0.0822	0.0444
rs6689641	A	0.0078	0.0014	-0.0711	0.0443
rs6690195	T	-0.0113	0.0014	0.0825	0.0443
rs6695132	T	0.0097	0.0017	-0.0470	0.0536
rs6696068	T	-0.0088	0.0014	0.1698	0.0453
rs6697584	T	-0.0126	0.0017	0.0539	0.0532
rs6704768	A	-0.0116	0.0014	-0.0445	0.0445
rs6706275	T	0.0083	0.0015	-0.0660	0.0468
rs6711399	T	-0.0107	0.0019	-0.0744	0.0583
rs6715321	T	-0.0097	0.0014	0.0222	0.0446
rs6715849	A	-0.0122	0.0014	0.0497	0.0446
rs6720515	A	0.0103	0.0017	-0.0073	0.0533

rs67224963	A	0.0113	0.0017	-0.1065	0.0551
rs6729612	T	0.0092	0.0015	0.0360	0.0476
rs6731373	A	-0.0115	0.0015	0.2127	0.0460
rs6736025	T	0.0079	0.0014	-0.0281	0.0447
rs6738860	A	-0.0088	0.0014	0.0397	0.0444
rs6743032	A	-0.0165	0.0024	-0.0926	0.0750
rs6744040	A	-0.0080	0.0014	-0.0169	0.0445
rs67456868	A	0.0119	0.0018	-0.0335	0.0568
rs6752228	T	-0.0080	0.0014	0.1231	0.0443
rs6757087	T	-0.0085	0.0014	0.0443	0.0450
rs67661275	A	0.0134	0.0025	0.1133	0.0780
rs6774533	T	0.0090	0.0015	-0.0767	0.0493
rs67790232	A	0.0101	0.0017	0.0570	0.0547
rs6780414	T	0.0105	0.0018	0.0470	0.0575
rs6792805	A	-0.0090	0.0016	-0.0601	0.0499
rs6812533	T	0.0106	0.0016	0.0686	0.0512
rs68145588	T	-0.0127	0.0021	0.0142	0.0625
rs6824567	T	0.0130	0.0016	-0.1792	0.0490
rs6853599	T	0.0108	0.0019	-0.1579	0.0576
rs6861925	C	-0.0090	0.0014	0.0301	0.0444
rs6871635	A	-0.0087	0.0014	0.1806	0.0449
rs6881581	A	-0.0096	0.0015	0.1074	0.0467
rs6881733	T	-0.0086	0.0016	-0.0823	0.0491
rs6917154	T	0.0127	0.0021	-0.0727	0.0662
rs6917204	T	0.0119	0.0017	-0.0491	0.0557
rs6918506	C	0.0113	0.0014	0.1136	0.0443
rs6924023	A	0.0107	0.0016	0.0321	0.0521
rs6932108	C	-0.0210	0.0027	-0.0901	0.0792
rs6946136	A	0.0081	0.0014	-0.0249	0.0452
rs6946362	T	0.0091	0.0015	-0.0112	0.0487
rs6951996	A	-0.0182	0.0032	0.1361	0.1022
rs6956283	T	0.0098	0.0016	0.0804	0.0500
rs6969783	A	-0.0098	0.0014	0.0598	0.0445
rs6977237	T	0.0082	0.0014	0.0983	0.0443
rs6989141	A	0.0100	0.0017	-0.0785	0.0553
rs6994287	A	0.0102	0.0014	-0.0428	0.0448
rs7012546	T	0.0092	0.0014	0.0065	0.0449
rs7016302	C	-0.0125	0.0019	0.0316	0.0589
rs702606	T	0.0118	0.0021	-0.1376	0.0648
rs7029718	A	0.0244	0.0014	0.0054	0.0448
rs7030373	A	-0.0098	0.0017	0.0598	0.0548
rs7035315	A	-0.0090	0.0015	0.1421	0.0460
rs7040995	C	0.0097	0.0014	-0.0667	0.0447
rs7041702	A	-0.0111	0.0016	-0.0265	0.0503
rs7084508	T	-0.0085	0.0015	0.1212	0.0464
rs710629	A	0.0082	0.0015	-0.1144	0.0465

rs7108020	A	0.0111	0.0015	0.0116	0.0461
rs711793	T	0.0103	0.0015	0.0842	0.0474
rs7127580	C	-0.0150	0.0023	0.0852	0.0705
rs713584	A	-0.0080	0.0014	0.0217	0.0448
rs71413877	A	0.0351	0.0036	-0.1507	0.1134
rs7146625	A	0.0091	0.0016	0.0647	0.0504
rs7147473	A	-0.0103	0.0015	-0.0656	0.0472
rs7158218	A	-0.0090	0.0015	0.0454	0.0483
rs71646142	T	0.0104	0.0018	-0.0279	0.0563
rs716513	A	0.0084	0.0014	-0.0389	0.0444
rs7167688	T	-0.0094	0.0014	0.0252	0.0443
rs7171405	A	-0.0101	0.0016	0.1533	0.0521
rs717996	T	-0.0122	0.0014	0.1049	0.0453
rs7182216	C	-0.0096	0.0017	-0.0982	0.0547
rs7190	A	-0.0095	0.0015	-0.1158	0.0462
rs721579	T	-0.0110	0.0016	-0.0302	0.0506
rs7218235	A	-0.0097	0.0017	0.0535	0.0546
rs7223311	C	-0.0079	0.0014	0.0280	0.0445
rs7226824	T	-0.0078	0.0014	0.0484	0.0443
rs72482130	T	0.0141	0.0019	-0.1144	0.0618
rs7254263	T	-0.0119	0.0016	0.1165	0.0494
rs7255223	A	0.0097	0.0016	-0.1611	0.0502
rs72622559	T	-0.0120	0.0017	0.0836	0.0567
rs72624911	T	0.0282	0.0034	-0.4305	0.1068
rs72636697	T	-0.0140	0.0020	0.0993	0.0652
rs72667460	T	0.0197	0.0031	-0.0523	0.0966
rs72671456	A	-0.0174	0.0020	0.0509	0.0646
rs72672052	A	-0.0101	0.0018	0.1032	0.0587
rs72673097	A	0.0257	0.0042	0.3710	0.1213
rs72677177	A	0.0100	0.0014	0.0298	0.0451
rs72694479	T	0.0127	0.0020	0.0107	0.0632
rs72709560	A	0.0085	0.0015	-0.0272	0.0472
rs72771860	T	-0.0211	0.0029	-0.0516	0.0924
rs72792395	T	-0.0145	0.0022	0.0301	0.0701
rs728054	A	-0.0128	0.0015	0.1003	0.0463
rs72819118	T	-0.0231	0.0024	0.0329	0.0752
rs72824753	T	0.0133	0.0024	0.0771	0.0724
rs72828517	T	-0.0168	0.0019	0.1766	0.0575
rs72829857	A	-0.0149	0.0016	0.0756	0.0524
rs72834698	A	0.0117	0.0020	-0.1791	0.0633
rs72881110	A	0.0146	0.0027	0.0048	0.0836
rs72883760	A	0.0101	0.0018	-0.1216	0.0555
rs72896637	T	0.0162	0.0028	-0.2336	0.0840
rs72902523	T	-0.0103	0.0016	-0.0099	0.0513
rs72906124	T	0.0185	0.0030	-0.0710	0.0956
rs72917504	T	-0.0178	0.0030	-0.2355	0.0938

rs72919450	T	-0.0132	0.0023	0.1270	0.0734
rs72944064	T	-0.0098	0.0016	0.1244	0.0506
rs72962169	T	-0.0174	0.0019	0.0667	0.0604
rs72972965	A	0.0083	0.0015	-0.1212	0.0462
rs72993796	T	0.0127	0.0022	-0.0831	0.0687
rs73034295	A	0.0099	0.0018	-0.0929	0.0567
rs730384	A	0.0101	0.0014	-0.0717	0.0448
rs73039077	C	0.0092	0.0016	-0.0356	0.0506
rs73055556	A	0.0111	0.0020	-0.0230	0.0638
rs73082325	C	-0.0252	0.0044	0.4356	0.1301
rs73106136	T	-0.0147	0.0026	-0.2982	0.0823
rs73154546	A	0.0197	0.0036	-0.2548	0.1120
rs7317761	C	0.0109	0.0016	-0.0111	0.0504
rs73191311	A	-0.0094	0.0015	0.0898	0.0469
rs7321274	A	0.0114	0.0018	0.0058	0.0550
rs73219806	A	0.0106	0.0019	0.0698	0.0606
rs7323027	A	0.0130	0.0015	0.0154	0.0471
rs7326331	A	-0.0128	0.0016	0.0372	0.0496
rs73344830	A	0.0170	0.0014	-0.1032	0.0448
rs73405293	A	0.0113	0.0020	0.0968	0.0623
rs73457936	A	0.0144	0.0025	0.0387	0.0778
rs7356536	T	-0.0129	0.0021	0.1913	0.0642
rs73581580	A	-0.0181	0.0022	-0.0355	0.0669
rs736281	T	0.0086	0.0014	-0.1172	0.0452
rs73643713	T	-0.0165	0.0027	0.0499	0.0849
rs736471	T	0.0083	0.0014	-0.0578	0.0446
rs73648455	T	-0.0183	0.0026	0.0022	0.0840
rs737945	C	-0.0115	0.0014	0.0870	0.0445
rs73874335	T	-0.0178	0.0029	0.1351	0.0970
rs73961845	A	-0.0114	0.0014	0.0141	0.0454
rs74091672	A	-0.0153	0.0022	0.0257	0.0677
rs7430651	T	-0.0111	0.0016	0.0177	0.0483
rs743316	T	0.0112	0.0017	0.1110	0.0543
rs74415461	T	0.0176	0.0026	-0.0280	0.0783
rs74453875	A	0.0214	0.0037	-0.0958	0.1195
rs74462621	T	0.0125	0.0020	-0.0452	0.0647
rs7449561	A	0.0102	0.0017	0.0139	0.0528
rs7451726	A	0.0109	0.0017	-0.0638	0.0547
rs74545339	A	-0.0158	0.0022	-0.0475	0.0703
rs7460106	T	-0.0123	0.0017	0.1675	0.0529
rs74615093	A	0.0145	0.0026	0.0582	0.0810
rs746839	C	0.0135	0.0015	-0.0711	0.0463
rs74747621	T	0.0153	0.0022	-0.1544	0.0703
rs74787922	A	0.0182	0.0028	0.0079	0.0877
rs74944275	T	0.0231	0.0035	0.1961	0.1050
rs74944857	T	-0.0089	0.0015	0.0714	0.0469

rs7495033	A	0.0095	0.0015	-0.3547	0.0489
rs75033012	C	-0.0258	0.0038	-0.2226	0.1127
rs75177132	T	0.0298	0.0036	-0.1252	0.1020
rs75203411	T	0.0135	0.0019	-0.0764	0.0590
rs7526112	T	0.0124	0.0015	0.0260	0.0459
rs75308819	A	0.0287	0.0047	0.1488	0.1494
rs75434274	A	0.0151	0.0026	-0.2109	0.0790
rs75500877	T	0.0207	0.0033	-0.2212	0.1020
rs7552964	A	0.0148	0.0023	-0.1681	0.0749
rs7560871	A	-0.0193	0.0027	0.1493	0.0866
rs7561705	A	0.0078	0.0014	0.0234	0.0443
rs7575637	A	0.0107	0.0014	-0.1676	0.0443
rs75756843	A	0.0288	0.0049	-0.0513	0.1665
rs7575938	A	0.0090	0.0015	0.0499	0.0467
rs7590368	T	-0.0146	0.0016	0.0811	0.0503
rs7597126	T	-0.0094	0.0014	0.0506	0.0443
rs7597412	C	0.0088	0.0015	-0.0321	0.0460
rs7603132	A	0.0137	0.0018	-0.1234	0.0556
rs76076331	T	0.0206	0.0021	-0.1192	0.0675
rs76077165	A	0.0165	0.0025	-0.2660	0.0799
rs76167224	T	-0.0187	0.0033	0.3284	0.0997
rs7617204	A	-0.0097	0.0014	0.1134	0.0443
rs76235882	A	0.0241	0.0039	-0.0173	0.1315
rs76241605	A	0.0139	0.0022	-0.1209	0.0690
rs76246107	A	-0.0162	0.0026	0.0092	0.0834
rs7625428	T	0.0098	0.0014	0.0803	0.0452
rs76267866	A	0.0104	0.0017	0.0159	0.0546
rs7630133	A	0.0082	0.0015	-0.0754	0.0464
rs763553	A	0.0087	0.0014	-0.0924	0.0445
rs7640424	T	0.0113	0.0015	0.0910	0.0477
rs7650602	T	-0.0089	0.0014	-0.0383	0.0446
rs76552497	T	-0.0120	0.0019	0.1559	0.0590
rs76577427	C	0.0203	0.0024	-0.0850	0.0722
rs7672622	A	-0.0088	0.0016	0.1055	0.0512
rs767943	A	-0.0122	0.0016	0.1687	0.0505
rs7683416	T	0.0139	0.0014	-0.1420	0.0445
rs76876592	A	0.0126	0.0020	0.0314	0.0648
rs76878669	C	0.0141	0.0017	-0.0414	0.0524
rs7692359	T	-0.0109	0.0017	0.0476	0.0538
rs76957677	T	-0.0297	0.0054	0.3804	0.1577
rs77025239	A	-0.0140	0.0019	0.0126	0.0618
rs7714719	C	0.0116	0.0020	-0.0845	0.0630
rs7716161	C	0.0105	0.0019	0.1367	0.0581
rs77201694	A	0.0156	0.0022	0.0167	0.0684
rs77370942	A	-0.0147	0.0027	-0.0610	0.0830
rs77554090	T	-0.0186	0.0027	0.0448	0.0841

rs77609760	A	0.0236	0.0028	-0.1124	0.0880
rs7766240	T	-0.0134	0.0024	0.0535	0.0746
rs77702622	A	-0.0213	0.0029	-0.0274	0.0890
rs77702819	T	0.0187	0.0025	-0.1398	0.0760
rs77719387	A	-0.0403	0.0058	0.7345	0.1775
rs7772172	A	0.0091	0.0014	0.0013	0.0450
rs7775100	T	-0.0087	0.0014	0.0873	0.0452
rs77826402	T	0.0151	0.0027	0.0683	0.0858
rs7796103	C	-0.0077	0.0014	-0.0284	0.0447
rs7799141	A	0.0099	0.0015	-0.0093	0.0474
rs77999825	A	0.0294	0.0039	-0.3624	0.1206
rs7803932	A	0.0127	0.0019	-0.0470	0.0591
rs780569	A	-0.0085	0.0016	0.0032	0.0489
rs78116078	C	0.0086	0.0016	0.0114	0.0488
rs7816777	T	-0.0088	0.0015	0.0473	0.0465
rs78193153	A	0.0191	0.0029	-0.2361	0.0913
rs7823700	T	0.0132	0.0024	-0.0325	0.0745
rs78365243	T	0.0181	0.0033	-0.0634	0.1010
rs78440611	A	-0.0147	0.0024	-0.0103	0.0761
rs7849480	A	-0.0084	0.0014	0.1135	0.0447
rs7849487	T	-0.0167	0.0015	-0.0176	0.0467
rs7855503	C	0.0088	0.0015	0.0300	0.0461
rs78648104	T	-0.0141	0.0025	-0.2874	0.0787
rs78702390	C	0.0167	0.0030	-0.1621	0.0929
rs78714229	T	0.0172	0.0030	0.1572	0.0947
rs7875078	A	-0.0078	0.0014	0.0303	0.0446
rs78918150	T	-0.0080	0.0015	0.0430	0.0456
rs7894722	T	0.0085	0.0014	-0.0025	0.0455
rs7899270	A	-0.0084	0.0015	-0.0422	0.0465
rs7905192	T	0.0084	0.0014	0.0859	0.0444
rs790647	A	-0.0145	0.0017	0.0758	0.0524
rs7910403	T	0.0122	0.0018	-0.1157	0.0554
rs7914674	T	-0.0139	0.0022	-0.0073	0.0549
rs7920624	A	0.0120	0.0014	-0.0910	0.0445
rs7924036	T	0.0133	0.0014	-0.1268	0.0442
rs79265434	A	-0.0197	0.0022	0.3693	0.0691
rs7928017	A	0.0096	0.0014	-0.0834	0.0445
rs7931563	T	-0.0109	0.0014	0.0410	0.0457
rs795230	T	0.0081	0.0014	0.1249	0.0447
rs7958371	A	-0.0085	0.0015	0.0506	0.0463
rs79585412	T	-0.0178	0.0031	0.2009	0.0945
rs7965154	A	-0.0085	0.0015	0.0832	0.0477
rs7967550	A	-0.0086	0.0014	-0.0129	0.0452
rs7972246	T	0.0110	0.0015	-0.2111	0.0467
rs79728014	A	-0.0134	0.0020	0.0659	0.0620
rs7974852	A	0.0117	0.0014	-0.1255	0.0442

rs7977614	A	-0.0134	0.0016	0.0936	0.0485
rs79798166	A	0.0172	0.0025	-0.0345	0.0800
rs79855925	T	-0.0194	0.0033	-0.0210	0.1082
rs79994730	T	0.0240	0.0035	-0.0523	0.1017
rs79997166	A	0.0172	0.0030	-0.2574	0.0926
rs80037907	T	-0.0107	0.0019	0.1139	0.0599
rs8008382	T	-0.0106	0.0015	-0.0225	0.0484
rs8009933	A	0.0088	0.0015	-0.0532	0.0477
rs8016504	A	-0.0087	0.0014	0.0150	0.0447
rs80223410	T	-0.0118	0.0020	0.0153	0.0641
rs8024	A	-0.0115	0.0015	0.1466	0.0466
rs80257979	T	-0.0252	0.0040	0.3318	0.1316
rs8030487	A	0.0085	0.0015	-0.1296	0.0478
rs803619	T	-0.0173	0.0023	0.1388	0.0743
rs8046072	A	0.0101	0.0018	-0.2254	0.0556
rs8052523	T	-0.0084	0.0014	-0.0496	0.0452
rs8066044	A	0.0090	0.0016	-0.2723	0.0501
rs806816	A	0.0101	0.0016	-0.2070	0.0511
rs8097125	T	0.0086	0.0014	-0.0636	0.0454
rs8103741	A	-0.0108	0.0019	0.0676	0.0576
rs818415	T	-0.0110	0.0018	0.0449	0.0566
rs8192465	A	-0.0272	0.0049	0.0320	0.1496
rs852771	T	0.0092	0.0015	-0.0804	0.0480
rs853286	T	0.0124	0.0023	-0.0205	0.0736
rs854796	A	0.0090	0.0015	0.0410	0.0476
rs868456	A	0.0089	0.0015	0.0084	0.0485
rs870589	A	-0.0079	0.0014	0.0490	0.0444
rs884108	A	-0.0094	0.0017	0.0101	0.0525
rs891793	C	-0.0099	0.0014	0.0584	0.0444
rs893522	A	0.0149	0.0025	-0.1039	0.0764
rs902820	A	-0.0096	0.0014	-0.0380	0.0448
rs911149	T	0.0091	0.0017	0.0304	0.0522
rs912883	T	0.0087	0.0015	0.0670	0.0475
rs913509	A	0.0096	0.0015	-0.0025	0.0469
rs925161	C	-0.0078	0.0014	0.0074	0.0443
rs9267658	T	0.0138	0.0020	-0.3224	0.0623
rs9267677	T	0.0180	0.0024	-0.0001	0.0758
rs9289300	T	-0.0164	0.0019	0.1380	0.0609
rs9291437	C	-0.0081	0.0014	0.0740	0.0455
rs9294770	T	0.0083	0.0014	0.0086	0.0459
rs929511	T	-0.0160	0.0021	-0.0372	0.0670
rs9300612	T	0.0084	0.0015	-0.0079	0.0483
rs933738	A	-0.0110	0.0018	0.3409	0.0588
rs9349956	A	-0.0159	0.0018	0.0147	0.0571
rs9359939	A	-0.0105	0.0016	0.0533	0.0523
rs936496	A	-0.0079	0.0014	-0.0423	0.0455

rs9371883	C	-0.0087	0.0015	0.0107	0.0461
rs9373363	A	-0.0111	0.0016	0.2332	0.0511
rs9375188	T	0.0212	0.0014	-0.1275	0.0443
rs9375403	C	0.0126	0.0016	-0.1313	0.0522
rs9386110	T	-0.0163	0.0023	0.0043	0.0713
rs9388490	T	0.0091	0.0014	0.0311	0.0446
rs939400	T	-0.0095	0.0015	0.0745	0.0461
rs9411331	A	0.0145	0.0015	-0.0490	0.0474
rs9435340	A	0.0086	0.0015	-0.0389	0.0467
rs9442750	A	-0.0089	0.0016	-0.0236	0.0502
rs9446060	A	0.0077	0.0014	0.0530	0.0456
rs9465509	A	-0.0084	0.0014	-0.0116	0.0442
rs9490512	A	-0.0136	0.0014	0.1525	0.0445
rs9492774	C	0.0080	0.0015	-0.0334	0.0459
rs9513416	A	-0.0116	0.0019	0.0626	0.0603
rs9513754	T	0.0096	0.0016	-0.0690	0.0489
rs9527662	A	0.0094	0.0014	0.0442	0.0466
rs9527905	A	-0.0094	0.0014	0.0940	0.0451
rs9529146	T	0.0127	0.0017	-0.0959	0.0528
rs9536462	A	0.0133	0.0020	-0.0822	0.0636
rs9540718	A	-0.0089	0.0014	0.0953	0.0444
rs9545395	T	0.0138	0.0021	0.0189	0.0663
rs9556958	T	-0.0110	0.0014	0.1348	0.0445
rs9563168	A	0.0101	0.0017	-0.1964	0.0546
rs9568798	T	-0.0093	0.0016	0.0421	0.0491
rs9597907	T	0.0152	0.0027	-0.0906	0.0872
rs9611597	A	-0.0107	0.0019	0.1415	0.0610
rs9616906	A	0.0122	0.0014	0.0272	0.0446
rs9616947	T	-0.0134	0.0015	0.0289	0.0456
rs9633970	T	0.0091	0.0016	-0.0251	0.0531
rs9649	T	0.0108	0.0019	-0.0675	0.0590
rs9655780	A	-0.0153	0.0019	0.0544	0.0593
rs9666728	T	-0.0082	0.0014	0.1334	0.0443
rs9683585	C	0.0084	0.0014	0.0636	0.0445
rs969512	A	-0.0106	0.0015	-0.0385	0.0469
rs9771228	T	0.0096	0.0015	0.0263	0.0462
rs977143	A	0.0085	0.0015	-0.1333	0.0459
rs978807	A	0.0139	0.0018	0.0005	0.0559
rs981230	T	0.0100	0.0014	0.0123	0.0443
rs981883	A	0.0099	0.0014	-0.1676	0.0451
rs9820604	T	0.0115	0.0018	-0.0636	0.0585
rs9821664	A	0.0094	0.0017	0.0067	0.0552
rs9830359	T	-0.0147	0.0022	-0.1921	0.0701
rs9844755	A	0.0083	0.0015	-0.0452	0.0470
rs9849884	A	-0.0090	0.0014	-0.1602	0.0449
rs9853928	T	-0.0127	0.0017	0.0596	0.0550

rs9858921	A	-0.0087	0.0014	0.1422	0.0446
rs9859556	T	0.0290	0.0015	-0.2416	0.0480
rs9866123	A	0.0086	0.0014	0.0268	0.0446
rs9870317	A	0.0086	0.0015	0.0057	0.0477
rs9877225	T	0.0090	0.0016	-0.0917	0.0517
rs9882532	T	0.0124	0.0015	-0.1023	0.0462
rs9886703	A	-0.0136	0.0019	-0.1061	0.0591
rs9888796	T	0.0113	0.0016	0.0796	0.0507
rs9916901	T	0.0092	0.0016	0.0039	0.0504
rs9924031	C	0.0093	0.0015	-0.1213	0.0455
rs9926649	T	0.0177	0.0031	0.3493	0.1024
rs9927049	A	0.0099	0.0016	0.1250	0.0508
rs9927137	A	0.0082	0.0014	-0.1498	0.0446
rs9927842	T	-0.0130	0.0020	0.0506	0.0609
rs9929556	T	-0.0099	0.0014	-0.0599	0.0448
rs9929762	A	0.0096	0.0014	-0.0378	0.0447
rs9929993	T	-0.0102	0.0015	0.1178	0.0464
rs9933256	A	0.0119	0.0014	-0.0576	0.0444
rs995698	A	-0.0099	0.0014	0.0732	0.0445
rs9974899	T	-0.0093	0.0016	0.0045	0.0514
rs9977825	T	-0.0092	0.0015	0.0773	0.0461

Supplementary Table 11: Genetic association estimates for the IVW ratio-method MR analysis estimating the effect of education on smoking. ea=effect allele; gx=education; gy=smoking; se=standard error

SNP	ea	gx	gx_se	gy	gy_se
rs10006235	T	-0.0093	0.0016	0.003612	0.00156
rs10009513	A	0.0088	0.0014	0.001024	0.001385
rs10032941	T	-0.008	0.0015	0.003399	0.001442
rs10041403	T	0.0151	0.0019	-0.00723	0.001837
rs10042828	A	-0.0145	0.0022	0.003836	0.002141
rs10046069	A	0.0127	0.0022	-0.00139	0.002166
rs10057590	A	0.0106	0.0014	-0.00351	0.001402
rs10060023	T	0.011	0.0015	-0.00349	0.001465
rs10061420	A	0.0082	0.0014	0.000716	0.001388
rs1006749	A	0.0079	0.0014	-0.00184	0.001386
rs10074178	A	0.0079	0.0014	0.002318	0.001388
rs1007731	A	-0.0143	0.0022	-0.00096	0.002167
rs10080647	A	0.0124	0.002	-0.0051	0.001997
rs1008078	T	-0.0182	0.0014	0.009224	0.00142
rs1009470	T	0.0086	0.0014	-4.06E-05	0.001395
rs10098073	A	-0.0091	0.0014	-0.00028	0.001395
rs10122669	A	-0.0079	0.0014	-0.00053	0.001407
rs10128888	A	-0.0083	0.0015	0.004269	0.00153
rs10145520	T	-0.0122	0.0018	0.007569	0.001742
rs10166286	T	0.0151	0.0019	-0.00131	0.001837
rs10169002	A	-0.008	0.0014	0.000395	0.001394
rs10181071	A	0.0102	0.0019	-0.0047	0.001831
rs10189857	A	0.0158	0.0014	-0.00331	0.001399
rs10192369	A	-0.0078	0.0014	0.001407	0.001387
rs10192834	T	0.0083	0.0015	-0.00247	0.001457
rs10193498	A	0.0097	0.0016	-0.00852	0.001634
rs10204051	T	-0.0101	0.0014	0.006349	0.001421
rs10205057	T	0.009	0.0014	-0.00465	0.001395
rs10208	T	0.0091	0.0015	-0.00028	0.001494
rs10215082	A	-0.0149	0.0014	0.003141	0.00139
rs1024268	T	-0.0084	0.0014	0.003369	0.001408
rs10251438	A	0.0078	0.0014	0.000107	0.001396
rs10264573	A	0.0175	0.0032	0.000271	0.003213
rs1035578	A	-0.0094	0.0014	0.005859	0.001397
rs10402747	T	0.0077	0.0014	-0.00496	0.001392
rs10411759	A	0.0116	0.002	-0.00474	0.001953
rs10444280	T	0.01	0.0018	-0.00614	0.001724
rs10460095	A	-0.0124	0.0014	0.003876	0.001405
rs10480450	A	0.0158	0.0028	-0.00398	0.002924
rs10496091	A	-0.0131	0.0016	0.005344	0.001542
rs10499535	A	0.008	0.0014	-0.00329	0.001386

rs1050847	T	0.0092	0.0014	-0.00797	0.001405
rs10509251	T	-0.0093	0.0017	0.001709	0.001664
rs10515007	T	0.0143	0.0019	0.000155	0.001893
rs1051860	A	-0.0079	0.0014	0.00429	0.001408
rs10519504	T	0.0119	0.0019	-0.00122	0.00191
rs1054442	A	-0.0136	0.0015	0.003692	0.001431
rs1061801	A	-0.011	0.0018	0.009375	0.001761
rs1066769	A	-0.023	0.0041	0.003756	0.003769
rs10742591	A	-0.0084	0.0014	0.005674	0.001426
rs10750539	A	-0.0095	0.0015	0.004313	0.001457
rs10752262	T	0.0102	0.0014	-0.0027	0.001402
rs1075228	A	-0.0081	0.0014	0.002384	0.001405
rs10761202	T	0.0088	0.0014	0.002485	0.001385
rs10761251	A	0.0113	0.0015	-0.00089	0.001473
rs10772644	C	0.0142	0.0022	-0.00487	0.002248
rs10773002	A	0.0185	0.0016	-0.00056	0.001606
rs10773208	T	-0.011	0.0016	0.000947	0.00159
rs10782651	T	-0.0082	0.0014	0.00446	0.001396
rs10783243	A	-0.0092	0.0014	0.004655	0.001388
rs10789285	T	0.0091	0.0016	6.71E-05	0.001653
rs10793903	A	0.0126	0.0022	-0.00621	0.002247
rs10795831	T	-0.0099	0.0017	0.005112	0.001711
rs10798888	T	-0.014	0.0019	0.009939	0.001838
rs10799615	A	-0.0089	0.0016	-0.00093	0.001591
rs10805383	A	-0.0103	0.0014	0.00564	0.001389
rs10810099	A	-0.0146	0.0016	0.003675	0.001551
rs10810145	A	0.0078	0.0014	-0.0046	0.001411
rs10830858	T	0.0092	0.0014	-0.00175	0.001387
rs10831656	T	0.009	0.0015	-0.00388	0.001509
rs10844179	A	0.0104	0.0017	-0.00231	0.001678
rs10845051	A	0.0083	0.0014	-0.00351	0.001394
rs10875121	C	0.0174	0.0019	-0.00465	0.001875
rs10877283	T	0.0085	0.0014	-0.00101	0.001414
rs10879676	T	-0.0083	0.0014	0.002396	0.001408
rs10886012	C	0.0078	0.0014	-0.00319	0.001407
rs10892807	T	0.0109	0.0014	-0.00261	0.001404
rs10899282	A	0.014	0.0017	-0.00338	0.001679
rs10927053	A	-0.0125	0.0022	0.011176	0.002248
rs10928190	T	-0.0087	0.0014	0.002985	0.001398
rs10931821	A	-0.0141	0.0014	0.004123	0.001392
rs10937240	T	-0.0123	0.0018	-0.0004	0.001761
rs10940540	A	0.0078	0.0014	-0.00161	0.001398
rs10940921	T	0.0111	0.0014	-0.00338	0.00141
rs10943588	A	0.0084	0.0015	-0.00123	0.001428
rs10947439	T	0.009	0.0015	-0.00354	0.001476
rs10949263	A	-0.0091	0.0016	0.005058	0.001625

rs10951590	T	-0.0109	0.0015	0.004047	0.001477
rs10974256	A	-0.0093	0.0015	0.002515	0.00147
rs1097784	T	-0.0083	0.0014	0.000959	0.00139
rs10979613	T	-0.0117	0.0015	0.005306	0.001439
rs10983324	A	0.0084	0.0015	-0.00264	0.001521
rs10984445	A	0.0115	0.0014	0.000246	0.001393
rs10985402	T	0.0089	0.0016	-0.00116	0.001612
rs10995639	T	0.0078	0.0014	-0.00171	0.001406
rs11003463	T	0.0091	0.0015	0.002952	0.001422
rs11021432	A	0.0103	0.0015	-0.0009	0.001429
rs11023764	A	0.0091	0.0015	-0.00056	0.001477
rs11030084	T	0.0106	0.0018	-0.01189	0.00178
rs11030102	C	0.0101	0.0016	-0.01092	0.001577
rs1106090	A	0.0094	0.0014	-0.00029	0.001441
rs11076962	T	0.0086	0.0016	-0.00342	0.001539
rs1107871	A	-0.0083	0.0014	0.001577	0.001396
rs11081529	T	0.0126	0.0015	0.003811	0.001526
rs11082011	T	0.0194	0.0015	-0.00799	0.001476
rs11100237	A	-0.0101	0.0014	-6.04E-05	0.001386
rs11115056	A	0.0187	0.0023	0.000382	0.002336
rs11121177	A	0.0151	0.0018	-0.00819	0.001825
rs111226181	T	-0.0201	0.0018	2.93E-05	0.001818
rs111235962	T	0.0133	0.0024	-0.00354	0.002337
rs11130380	T	0.009	0.0014	-0.0051	0.001417
rs111370527	T	0.0151	0.0027	0.003834	0.002574
rs11138947	T	0.0105	0.0016	-0.00207	0.00155
rs111530150	T	0.0269	0.0048	-0.00436	0.005881
rs11155813	T	-0.0129	0.0023	0.001397	0.002313
rs11157931	A	-0.0139	0.0014	0.001246	0.001419
rs11158800	A	-0.0091	0.0014	0.005767	0.00139
rs1117152	T	0.0106	0.0015	-0.00296	0.001522
rs11174399	A	0.0111	0.0018	-2.43E-05	0.001719
rs111821073	T	0.0124	0.0019	-0.00374	0.001921
rs111852224	T	0.0156	0.0022	-0.00342	0.002115
rs11190955	T	-0.0083	0.0015	0.004499	0.00145
rs1120924	T	0.0108	0.0015	-0.00385	0.001462
rs11209894	A	0.0119	0.0021	-0.0005	0.002012
rs11210400	A	0.012	0.0014	-0.00216	0.001397
rs11211123	A	0.0104	0.0017	-0.00578	0.001647
rs11213482	A	-0.0106	0.0019	0.003447	0.001833
rs112210983	A	0.0105	0.0017	-0.00277	0.001678
rs1123285	C	-0.0094	0.0015	0.002702	0.00149
rs112375785	A	0.0184	0.0021	-0.00456	0.001992
rs112379405	C	-0.0082	0.0014	0.002314	0.001398
rs112512729	T	-0.0166	0.0027	0.008879	0.002772
rs11265191	T	0.0083	0.0015	-5.83E-05	0.001466

rs112806496	C	-0.016	0.0025	0.001189	0.002422
rs1128956	T	-0.014	0.0019	0.005527	0.00181
rs113205706	A	-0.0135	0.0024	0.003597	0.002356
rs113520408	A	0.0155	0.0016	-0.00046	0.001544
rs113552169	C	0.0294	0.0053	-0.00549	0.005253
rs113588399	T	-0.0104	0.0017	-0.00108	0.001707
rs113615161	T	-0.0123	0.0021	0.006323	0.002039
rs113720505	A	0.0164	0.0029	-0.00271	0.002864
rs113731629	T	0.0339	0.0061	-0.00323	0.006051
rs114192810	T	-0.0279	0.0051	-0.00797	0.004952
rs114468556	A	0.0293	0.0036	-0.00199	0.00334
rs114593137	A	-0.0122	0.0017	0.004189	0.001714
rs1146079	C	-0.0087	0.0016	0.002488	0.00158
rs114810763	A	-0.0121	0.002	0.004972	0.001852
rs114952970	T	0.0265	0.004	-0.01845	0.00415
rs114958262	T	-0.022	0.0039	-0.0015	0.003841
rs115000530	A	-0.025	0.0031	0.007924	0.003055
rs115017135	A	-0.0206	0.0038	0.000839	0.003793
rs11542663	A	0.0112	0.0015	-0.00082	0.001524
rs115438240	T	-0.0169	0.003	0.007791	0.003128
rs115693355	A	0.0309	0.0053	-0.00587	0.005189
rs11588857	A	0.0199	0.0017	-0.00727	0.001702
rs11591870	T	0.0098	0.0017	-0.00286	0.001698
rs11592299	A	0.0097	0.0018	0.006635	0.001714
rs11596387	T	0.0258	0.0046	0.002738	0.004671
rs11598765	A	-0.0106	0.0018	0.003528	0.001763
rs11599236	T	-0.0106	0.0014	0.009375	0.00142
rs11613431	A	-0.0094	0.0016	-0.00061	0.001573
rs11620355	A	0.0182	0.0025	-0.00537	0.002478
rs11623285	T	-0.0135	0.0021	0.001923	0.00201
rs116386746	C	0.0249	0.0045	-0.02055	0.004535
rs11640569	A	0.0112	0.002	-0.0027	0.001995
rs11644446	A	0.0117	0.0019	-0.00421	0.001964
rs11647188	A	0.0079	0.0014	-0.0014	0.001418
rs11652522	A	-0.0154	0.0023	0.000877	0.002307
rs11657342	A	0.014	0.0019	-0.00362	0.001437
rs11657979	A	-0.0091	0.0017	0.006409	0.00164
rs11663602	A	-0.013	0.0016	0.006874	0.001554
rs11663678	T	-0.0121	0.0022	0.005762	0.002138
rs116656374	T	-0.0242	0.0038	-0.00036	0.003629
rs11675476	T	-0.0127	0.0014	-0.00054	0.001386
rs1167827	A	0.0104	0.0014	-0.00643	0.001398
rs11678980	A	-0.0166	0.0014	0.006583	0.001435
rs11687736	A	-0.0105	0.0015	0.00588	0.001485
rs11693885	A	-0.0091	0.0014	0.009369	0.0014
rs116967397	A	0.0315	0.0056	-0.00947	0.005348

rs117005905	T	0.015	0.0022	-0.00048	0.002242
rs11703948	A	-0.0168	0.0024	-0.00233	0.002296
rs1171040	A	-0.0113	0.0017	0.004085	0.001716
rs11716398	A	0.0159	0.0021	0.001227	0.002069
rs11720093	T	0.015	0.0026	-0.0006	0.002529
rs11720985	T	0.0085	0.0015	1.24E-05	0.001503
rs11724690	T	0.0093	0.0015	-0.00021	0.001546
rs117273411	T	-0.0211	0.0034	0.00464	0.003469
rs11732160	A	0.0105	0.0016	-0.00477	0.001575
rs11732657	A	-0.0126	0.0016	0.003212	0.001588
rs11733439	A	-0.0096	0.0017	0.003434	0.001718
rs11736863	A	0.0142	0.0018	-0.00137	0.001799
rs117398064	C	-0.0141	0.0025	0.006766	0.002372
rs117468730	A	-0.031	0.005	-0.00111	0.004938
rs117623407	A	-0.0118	0.002	0.00138	0.001956
rs11765387	T	0.0086	0.0015	-0.00495	0.001476
rs117668569	T	0.0234	0.0042	-0.00899	0.004277
rs117677995	A	-0.018	0.0032	0.00175	0.003169
rs11772232	T	0.0177	0.0019	-0.00769	0.001907
rs11774212	T	0.0128	0.0014	-0.00311	0.001391
rs117799466	C	0.0106	0.0016	-0.0044	0.001519
rs11780023	T	0.0085	0.0014	-0.00397	0.001388
rs11789013	T	0.0106	0.0016	-0.00334	0.001627
rs117895796	T	0.0362	0.0064	-0.01214	0.006719
rs118040169	A	-0.0248	0.0038	-0.00068	0.003701
rs118093058	T	0.0146	0.0021	0.005401	0.002011
rs118134876	T	-0.0223	0.003	0.008583	0.002921
rs11845781	T	-0.0079	0.0014	0.004206	0.001395
rs11871429	A	0.0128	0.0017	-0.00184	0.001656
rs11876620	T	0.0143	0.0024	-0.0018	0.002306
rs11894424	A	0.0209	0.0033	-0.00332	0.003329
rs11917701	A	-0.0087	0.0014	0.001999	0.00142
rs11919835	C	0.0089	0.0015	-0.00335	0.00144
rs1196760	C	0.0139	0.0025	-0.0025	0.002408
rs12005151	A	0.0087	0.0015	-0.00161	0.001458
rs12028010	T	0.0164	0.0017	0.004008	0.001649
rs12028229	T	0.0099	0.0016	-0.00098	0.001587
rs12054166	C	0.0096	0.0016	-0.00305	0.001587
rs12076635	C	0.0207	0.0017	-0.00904	0.001672
rs12113634	T	0.0088	0.0015	-0.00273	0.00145
rs12123293	T	0.0086	0.0015	-0.00328	0.001444
rs12126231	A	0.0095	0.0014	-0.00273	0.001413
rs12127928	T	0.0108	0.0018	-0.00257	0.001743
rs12145078	A	-0.0127	0.0017	0.00498	0.001724
rs12151248	T	-0.0149	0.0023	0.003731	0.002172
rs12170452	A	0.0105	0.0014	-0.0017	0.001394

rs1220779	A	-0.0079	0.0014	-0.00154	0.001388
rs12234369	A	0.0087	0.0015	0.000685	0.001503
rs12238011	T	-0.0141	0.0026	0.007181	0.002513
rs12273435	A	-0.0132	0.0017	0.007328	0.001716
rs12285074	A	0.0161	0.0023	-0.00576	0.002399
rs12304188	A	0.0111	0.0019	-0.00271	0.001928
rs12325727	A	0.0088	0.0014	-0.00436	0.00139
rs12332731	A	0.0135	0.0018	-0.0044	0.001738
rs12342546	A	-0.0128	0.0017	0.000295	0.001636
rs12359372	T	-0.0092	0.0015	0.002665	0.001485
rs12375949	T	-0.0138	0.0014	0.004996	0.001395
rs12405889	T	-0.0098	0.0014	-2.57E-05	0.001381
rs12431682	T	-0.0093	0.0015	0.001169	0.001439
rs12438177	A	0.0093	0.0015	-0.00077	0.001437
rs12453682	T	0.0102	0.0015	-0.00775	0.001514
rs1245829	A	-0.0099	0.0014	0.003304	0.001402
rs12467175	T	-0.0081	0.0014	0.001371	0.001385
rs12468040	T	0.0137	0.0014	-0.00403	0.001425
rs12473986	T	-0.0085	0.0015	0.004909	0.001532
rs12477385	T	0.0109	0.0017	-0.00726	0.001662
rs12478156	T	0.0108	0.0015	-0.00051	0.001446
rs12503522	T	-0.0113	0.0016	0.005673	0.001539
rs12506221	T	-0.013	0.0014	0.005333	0.001405
rs12516990	C	-0.0099	0.0017	0.00222	0.001717
rs12519073	T	-0.0107	0.0017	0.004531	0.001662
rs12524795	T	0.0102	0.0014	-0.00169	0.001394
rs12568153	T	-0.0091	0.0015	-0.00543	0.001533
rs12571549	A	0.0151	0.002	0.000598	0.002011
rs12574281	A	-0.008	0.0015	-0.00017	0.001431
rs12591647	T	-0.0152	0.0018	0.002082	0.00181
rs12601380	A	-0.0082	0.0014	0.003541	0.001403
rs12602286	T	0.016	0.0021	-0.00344	0.002085
rs12613500	C	0.0098	0.0014	-0.00038	0.001403
rs12614263	A	0.0083	0.0014	-0.00175	0.00139
rs12638072	A	-0.0107	0.0015	0.00273	0.001535
rs12643771	T	0.013	0.0015	-0.00232	0.001503
rs12646216	T	0.0084	0.0014	-0.003	0.001415
rs12646523	T	-0.0132	0.0016	0.000257	0.001594
rs12659776	T	0.0085	0.0014	-0.00681	0.001391
rs12670376	A	0.0092	0.0014	-0.00104	0.001393
rs1267062	C	0.0113	0.0018	-0.00051	0.001815
rs12682775	T	-0.0119	0.0017	0.004864	0.001679
rs12694681	T	0.0102	0.0015	-0.00211	0.001495
rs12709186	A	-0.0091	0.0015	0.004399	0.001469
rs12712269	T	-0.0107	0.0014	0.001221	0.001425
rs12746551	C	0.0248	0.0041	-0.00678	0.003946

rs12750688	C	0.0116	0.0016	0.000445	0.001562
rs12761729	T	-0.0098	0.0016	0.003291	0.001595
rs12761761	T	0.0153	0.0017	-0.00255	0.001601
rs12764593	C	0.0202	0.0029	-0.01168	0.002979
rs12765185	A	-0.0088	0.0016	0.004926	0.001564
rs12774577	T	0.0142	0.002	-0.01201	0.001975
rs12789313	T	0.0092	0.0014	0.000273	0.001385
rs12790196	T	-0.0101	0.0015	0.004456	0.001475
rs12810587	T	0.0094	0.0015	0.003002	0.001494
rs12875339	A	-0.0108	0.0015	0.0051	0.001449
rs12888615	T	0.012	0.0018	-0.00069	0.001736
rs12891042	T	0.0092	0.0014	-5.41E-05	0.001421
rs12891191	C	0.008	0.0014	-0.00141	0.001388
rs12912465	T	-0.0104	0.0016	0.001191	0.001593
rs12952191	T	-0.0081	0.0014	0.002278	0.001387
rs12953422	A	-0.008	0.0014	0.001686	0.001408
rs12957463	A	0.0146	0.0018	-0.00373	0.001724
rs12962845	C	-0.0123	0.0021	0.000471	0.002044
rs12967010	T	0.0114	0.0017	-0.00405	0.001676
rs12970264	A	-0.0085	0.0014	0.006134	0.001395
rs12981405	T	-0.0112	0.0019	0.004598	0.001869
rs13009915	T	0.0212	0.0037	0.001172	0.003592
rs13010288	T	0.0198	0.0021	-0.00682	0.00204
rs13010566	A	-0.0091	0.0014	-9.84E-05	0.001386
rs13015496	C	0.0119	0.0018	-0.00798	0.001776
rs13016316	A	-0.0138	0.0024	0.006373	0.002398
rs1301838	T	-0.0096	0.0015	0.000489	0.001476
rs13018640	T	-0.0215	0.0014	0.003504	0.001414
rs13026611	A	-0.0084	0.0015	0.000687	0.001453
rs13029602	T	-0.0092	0.0014	-0.00231	0.001422
rs13034349	T	-0.0082	0.0014	0.004117	0.001418
rs13050131	A	-0.0081	0.0015	0.004402	0.001482
rs13085461	C	0.0103	0.0014	-0.00351	0.001397
rs13099165	T	-0.0118	0.0017	0.001804	0.001721
rs13107325	T	-0.0239	0.0027	-0.00381	0.002634
rs13117856	A	0.0084	0.0015	-0.00206	0.001529
rs13130765	C	-0.0091	0.0014	0.000931	0.001386
rs13133213	A	0.0096	0.0014	-0.00298	0.001383
rs13145650	T	-0.0173	0.0025	0.007803	0.002478
rs13154429	C	-0.0136	0.0023	0.003417	0.002205
rs13163062	T	0.0115	0.0014	-0.00393	0.001403
rs13163845	T	-0.0149	0.002	0.008783	0.001984
rs13168136	A	-0.0092	0.0017	0.001769	0.001642
rs13169187	A	-0.0079	0.0014	0.002575	0.00139
rs13177031	A	-0.0085	0.0015	0.001195	0.001444
rs13190235	T	-0.0138	0.0024	0.000241	0.002374

rs13197257	T	0.0109	0.0016	-0.00362	0.001568
rs1320139	C	-0.0149	0.0014	0.000935	0.001402
rs13212041	T	0.0101	0.0018	-0.00046	0.001722
rs13240401	T	0.0176	0.0017	-0.00167	0.00166
rs13246220	T	-0.0093	0.0014	-6.92E-05	0.001421
rs13261773	C	0.0122	0.0019	-0.00183	0.001921
rs13266287	A	-0.0085	0.0015	0.005165	0.001477
rs13281564	A	0.0088	0.0014	-0.00227	0.001389
rs13284516	C	-0.0082	0.0014	0.002902	0.00139
rs1329044	T	0.0134	0.0019	-0.00351	0.001826
rs1329125	T	-0.0097	0.0015	0.004598	0.001484
rs13296345	T	0.009	0.0015	-0.00251	0.001442
rs13318986	A	0.0124	0.0015	-0.00232	0.001465
rs13327482	A	-0.0121	0.0018	0.003373	0.001833
rs1334297	A	0.0257	0.0016	-0.00702	0.001574
rs1335482	T	0.0096	0.0014	-0.00388	0.001387
rs13361043	T	-0.0158	0.0028	0.00267	0.002792
rs13381557	A	-0.0087	0.0014	0.001006	0.001394
rs13388333	A	0.0085	0.0015	0.000131	0.001436
rs13397529	C	-0.0097	0.0017	0.004073	0.001639
rs13398860	A	0.0086	0.0015	-0.00596	0.00145
rs13402497	A	-0.008	0.0014	0.000429	0.001389
rs13422673	T	-0.011	0.0014	0.001457	0.00139
rs13425585	C	-0.0089	0.0014	0.00095	0.00139
rs1368250	T	0.025	0.0045	-0.00436	0.004027
rs137079	T	0.0128	0.002	-0.00546	0.001976
rs137858393	A	0.0193	0.0029	0.000973	0.002788
rs138096147	A	0.0234	0.0042	-0.00461	0.004155
rs138484388	T	-0.0306	0.0051	0.00023	0.004772
rs1391513	T	-0.0095	0.0015	0.002156	0.001518
rs139244147	A	-0.0193	0.003	0.004224	0.003052
rs1392816	T	0.0082	0.0014	-0.00984	0.001433
rs139980871	T	0.018	0.0029	0.003086	0.002786
rs1404549	A	-0.0089	0.0015	0.000394	0.001487
rs1405876	T	0.0109	0.0015	0.004789	0.001448
rs140711597	C	0.0366	0.0055	-0.00985	0.004909
rs1408284	C	-0.0127	0.002	0.002743	0.002025
rs1408430	C	0.0096	0.0016	-0.00227	0.001549
rs141729694	T	0.0195	0.0026	0.001601	0.002656
rs142014757	A	0.0102	0.0018	-0.00644	0.001736
rs1426619	T	0.0096	0.0014	-0.00333	0.001402
rs1427298	T	0.0086	0.0014	-0.00216	0.001414
rs142747148	A	0.0329	0.0052	-0.00427	0.005315
rs1427829	A	-0.0083	0.0014	0.005488	0.001391
rs143148393	T	-0.0211	0.0039	0.001786	0.004117
rs1434630	T	-0.0137	0.002	0.003822	0.001929

rs143743568	A	0.0118	0.002	-0.00364	0.00193
rs143812851	A	-0.0113	0.0019	-0.00072	0.001835
rs1440930	C	0.0081	0.0014	-0.00102	0.0014
rs144336753	A	0.0388	0.0053	-0.01928	0.005054
rs1445591	A	0.0085	0.0015	-3.56E-05	0.001528
rs1464297	T	-0.0109	0.0015	-0.00023	0.001463
rs1467737	T	-0.0084	0.0014	0.002573	0.0014
rs1485300	C	0.0098	0.0015	0.002036	0.001483
rs1490612	T	0.0193	0.0026	0.003371	0.002616
rs150252215	A	0.0204	0.0035	0.001639	0.003485
rs150421637	A	0.0349	0.0061	-0.00994	0.005704
rs150537577	A	-0.0151	0.0026	-0.00134	0.00264
rs1505676	C	0.0084	0.0015	-0.00219	0.00143
rs151381	T	-0.0087	0.0014	-0.00017	0.001384
rs1518890	T	-0.009	0.0016	0.000156	0.001582
rs1527878	A	-0.0105	0.0016	0.002319	0.001621
rs1529597	A	0.0223	0.0038	-0.00708	0.00406
rs1538389	T	-0.0109	0.0018	0.004964	0.001762
rs1542354	A	-0.0084	0.0014	0.002296	0.001397
rs1544	A	-0.0096	0.0016	0.003682	0.001569
rs1554798	A	-0.008	0.0014	-0.00155	0.001423
rs1564347	T	0.0088	0.0015	-0.00309	0.001459
rs1569092	A	0.016	0.002	-0.00611	0.001909
rs1593022	T	-0.0099	0.0017	0.002296	0.001635
rs1603460	T	0.009	0.0014	-0.00085	0.001405
rs162445	A	0.0152	0.0026	-0.00444	0.002597
rs163229	C	-0.026	0.0047	0.005898	0.004688
rs1637770	T	0.0191	0.0028	-0.00448	0.002877
rs164938	T	-0.0088	0.0014	0.002011	0.00142
rs165633	A	-0.0116	0.0016	-0.00035	0.001668
rs1656614	C	0.0122	0.0015	-0.00742	0.001509
rs1671269	T	-0.01	0.0016	0.004326	0.00161
rs1671770	A	0.0125	0.0018	-0.003	0.001836
rs16851779	T	-0.0153	0.0027	0.001034	0.002529
rs16871807	T	0.009	0.0015	-0.00597	0.001468
rs1689510	C	0.0194	0.0015	-0.00671	0.001465
rs16901689	T	0.0111	0.0019	-0.00519	0.001866
rs1693584	T	-0.0087	0.0015	0.002155	0.0015
rs16958559	T	0.0096	0.0017	-0.006	0.001653
rs16966271	T	0.0092	0.0016	-0.00143	0.00155
rs16975275	A	-0.0102	0.0017	0.004024	0.001645
rs17048801	A	-0.0106	0.0014	0.003562	0.001446
rs17048855	A	0.0108	0.0015	-0.00113	0.001456
rs17069646	T	0.0085	0.0015	-0.00477	0.001498
rs17110109	T	-0.0093	0.0014	0.003841	0.001421
rs17113730	A	-0.017	0.0027	0.001996	0.002714

rs17131123	A	-0.0243	0.0039	0.008006	0.003584
rs17133297	A	-0.0146	0.0023	0.010347	0.002394
rs17144467	A	0.0098	0.0015	0.001374	0.001493
rs17148998	A	0.0101	0.0017	-0.00337	0.001711
rs171697	C	0.0133	0.0015	-0.00272	0.001474
rs17170519	C	0.0132	0.0023	-0.00497	0.002258
rs1717204	A	-0.0117	0.0018	0.004896	0.001779
rs1718188	A	0.0096	0.0014	-0.00362	0.001398
rs17186106	T	0.0108	0.0018	-0.00259	0.001824
rs17190418	T	-0.0213	0.0031	0.002936	0.002956
rs17205908	T	-0.0092	0.0015	0.002319	0.001462
rs17248751	A	-0.0136	0.0017	0.003136	0.001658
rs1728118	A	0.0089	0.0016	-0.0017	0.00156
rs1729412	T	-0.0107	0.0014	0.001043	0.001406
rs173003	A	-0.0082	0.0014	0.005276	0.001387
rs1734370	A	0.0099	0.0016	-0.00089	0.001524
rs1738050	C	-0.0093	0.0014	0.003826	0.001425
rs17411339	A	0.014	0.0014	-0.00607	0.001404
rs17428076	C	0.0137	0.0016	-0.00055	0.001618
rs17440885	A	0.016	0.0029	-0.00582	0.002861
rs1747714	T	0.0106	0.0014	-0.00226	0.001394
rs1747817	T	-0.0092	0.0016	0.006445	0.001616
rs17489649	A	0.0127	0.0015	-0.00494	0.00147
rs17502934	T	-0.0167	0.002	0.005555	0.00198
rs175325	A	-0.01	0.0014	0.001375	0.00142
rs17536059	C	-0.0108	0.0019	0.001278	0.001814
rs17551064	A	0.0141	0.0019	-0.00539	0.00188
rs17563464	A	-0.0147	0.0017	0.001902	0.00169
rs17565975	A	-0.0106	0.0014	0.002435	0.001404
rs17568389	A	0.0127	0.0014	-0.00089	0.001389
rs17574007	A	-0.0129	0.0021	0.006144	0.002167
rs1758747	A	0.0092	0.0015	-0.00062	0.001505
rs17598675	T	-0.0116	0.0014	0.001379	0.001397
rs17604349	A	0.0127	0.0018	0.005656	0.001809
rs17609255	T	-0.0087	0.0014	0.007083	0.001406
rs176218	T	0.02	0.0018	-0.00377	0.001769
rs17622379	T	-0.0113	0.0018	-0.0009	0.00177
rs17650634	T	-0.0104	0.0019	-0.00028	0.001848
rs17667540	A	-0.0087	0.0015	0.003018	0.001509
rs17669337	T	-0.011	0.0014	0.000603	0.001404
rs17680712	T	-0.0092	0.0014	0.001955	0.001399
rs17686649	T	-0.0098	0.0017	-0.00098	0.00169
rs17732878	T	-0.0095	0.0017	0.0036	0.001693
rs17742342	A	-0.0119	0.0018	0.004126	0.001729
rs17747544	A	-0.009	0.0014	-6.73E-05	0.001386
rs1779549	A	0.0081	0.0014	-0.00181	0.001394

rs178183	T	0.0145	0.0016	-0.00412	0.001601
rs17882802	A	0.0084	0.0014	-0.00252	0.001393
rs17883331	A	-0.0108	0.0018	0.006166	0.001749
rs1792602	A	-0.0097	0.0014	0.001856	0.001415
rs181214	T	-0.0104	0.0017	0.00383	0.001704
rs182902112	A	-0.03	0.0053	0.007629	0.005269
rs1835339	T	0.0084	0.0014	-0.00175	0.001429
rs183869217	C	0.0174	0.003	-0.008	0.00274
rs1841023	A	-0.0092	0.0015	0.002409	0.0015
rs1842713	A	-0.0117	0.0017	0.003741	0.001692
rs1843815	A	0.0077	0.0014	-0.00543	0.001391
rs1861786	A	-0.0081	0.0014	-0.00111	0.001426
rs186456786	A	0.0455	0.0079	-0.01175	0.007261
rs1865955	T	0.0122	0.0019	-0.00648	0.001825
rs1866823	A	0.0107	0.0014	-0.00673	0.001407
rs187580	T	-0.0104	0.0017	0.00012	0.00163
rs187951956	A	-0.0315	0.0058	-0.00237	0.006203
rs1880088	A	-0.0097	0.0016	0.000488	0.001589
rs1880692	A	0.0086	0.0014	-0.00176	0.001394
rs1890132	T	-0.0089	0.0015	0.003494	0.001517
rs1898111	A	0.0101	0.0018	-0.00963	0.00182
rs190102446	T	-0.021	0.0037	0.001361	0.003785
rs1905616	A	0.0083	0.0015	-0.00242	0.001472
rs1910005	T	-0.009	0.0015	0.000944	0.001527
rs1918394	T	0.0119	0.0019	-0.00489	0.001864
rs191903670	A	0.0335	0.0061	-0.01651	0.006528
rs192436652	T	-0.0319	0.0045	0.011409	0.004267
rs1933264	T	0.01	0.0016	-0.00587	0.001607
rs1952183	A	-0.0089	0.0014	0.003695	0.001387
rs1955250	A	-0.014	0.0025	0.001905	0.002479
rs1963381	A	0.0099	0.0016	0.000372	0.001621
rs1980129	A	0.0085	0.0014	-0.00096	0.001386
rs198262	T	-0.0223	0.0035	0.000709	0.003462
rs1991585	T	-0.0101	0.0015	0.000275	0.001519
rs1995181	A	-0.0078	0.0014	0.003715	0.001389
rs2007655	T	0.0086	0.0014	-0.00165	0.001388
rs2011603	A	-0.0091	0.0016	-0.00115	0.001574
rs2014830	T	0.011	0.0015	-0.00326	0.001514
rs2023016	C	0.0107	0.0017	-0.00269	0.001693
rs2024568	T	-0.0097	0.0016	0.003589	0.001613
rs2029401	A	-0.0091	0.0014	-0.00111	0.001409
rs2034631	T	0.0089	0.0015	-0.00362	0.001456
rs2039204	A	0.0082	0.0014	-0.0009	0.001388
rs2043187	A	0.0107	0.0015	-0.00459	0.001497
rs2052285	A	0.0112	0.0014	-0.00296	0.001419
rs2055940	A	0.0082	0.0015	-0.00118	0.001479

rs2077235	T	0.0097	0.0017	-0.0024	0.001631
rs2081652	A	0.0123	0.0015	-0.00095	0.001463
rs2082317	T	-0.0078	0.0014	0.000948	0.001412
rs2088913	A	0.0081	0.0014	-0.00141	0.001403
rs2092248	T	-0.0083	0.0015	0.002157	0.001516
rs2098526	A	-0.0241	0.0043	0.00506	0.004228
rs2100249	T	-0.0089	0.0015	0.002597	0.001468
rs2126069	T	0.008	0.0014	0.002547	0.001396
rs2131167	A	0.008	0.0014	-0.00757	0.001397
rs214626	A	0.0101	0.0018	-0.00338	0.001794
rs2160514	A	-0.0094	0.0014	0.006993	0.001417
rs2179152	T	-0.0131	0.0015	0.009051	0.001431
rs2183271	T	0.0084	0.0015	-0.00641	0.001444
rs2199409	T	0.0103	0.0017	-0.00414	0.001728
rs2212430	T	-0.01	0.0016	0.001983	0.00152
rs2216144	T	0.0098	0.0014	-0.00124	0.001393
rs2220926	T	-0.0095	0.0014	0.002712	0.001404
rs2250660	C	0.008	0.0014	-0.00102	0.001387
rs2252098	T	-0.0077	0.0014	0.003147	0.001388
rs2254681	A	0.0101	0.0016	0.000419	0.001586
rs2256965	A	0.0106	0.0014	-0.00139	0.001408
rs2276209	A	0.0085	0.0015	-0.00257	0.001531
rs2283076	A	0.0115	0.0017	-0.00535	0.001654
rs2287838	A	-0.0104	0.0014	0.001274	0.001396
rs2290601	T	-0.0149	0.0017	0.00429	0.001641
rs2297293	C	0.0099	0.0015	0.000651	0.001508
rs2299156	T	0.0099	0.0018	-0.00125	0.00182
rs2314338	T	0.0093	0.0016	-0.0046	0.001561
rs2321157	A	-0.0081	0.0014	0.000598	0.001392
rs232496	T	0.009	0.0015	0.00134	0.001448
rs2332179	A	0.0121	0.002	-0.00162	0.002025
rs2336721	T	0.0095	0.0015	-0.00623	0.001471
rs2358628	A	-0.0091	0.0015	0.002357	0.001499
rs236318	C	-0.0102	0.0017	0.003596	0.001639
rs2364544	A	-0.0105	0.0014	0.000136	0.001424
rs2365376	A	0.0092	0.0015	-0.00271	0.001476
rs2368831	T	-0.0092	0.0014	0.003296	0.001414
rs2373124	A	-0.0096	0.0016	0.000905	0.001634
rs2406253	A	0.0135	0.0018	-0.00108	0.001744
rs2414072	A	-0.0083	0.0014	0.004721	0.001394
rs2416214	A	0.0079	0.0014	-0.00142	0.0014
rs2416759	A	-0.0093	0.0015	0.004877	0.001515
rs2416845	T	0.0263	0.0048	-0.00547	0.004746
rs2431023	A	0.0093	0.0014	0.000422	0.001402
rs2434672	A	0.0091	0.0014	-0.00362	0.001409
rs2436760	T	0.0232	0.0042	-0.01307	0.004001

rs2447097	T	0.0096	0.0014	-0.00489	0.0014
rs2458370	T	-0.0086	0.0015	0.002584	0.001451
rs2469226	A	0.0096	0.0017	-0.0052	0.001665
rs2470966	C	-0.0103	0.0015	0.005383	0.001509
rs2478208	C	-0.0103	0.0014	0.003096	0.00139
rs2496482	T	0.0121	0.0015	-0.00413	0.001415
rs2517086	C	-0.0088	0.0014	0.003945	0.001409
rs2521602	A	-0.0266	0.0049	0.015276	0.004685
rs2522545	T	-0.0083	0.0014	-0.0017	0.001411
rs2526398	C	-0.0222	0.0015	0.007324	0.001451
rs2529069	T	-0.0106	0.0016	0.003994	0.001575
rs252991	A	0.0095	0.0015	-0.00346	0.001434
rs2542673	A	0.0088	0.0015	0.000529	0.001482
rs2545795	A	0.0125	0.0014	-0.00445	0.001396
rs255053	A	-0.0111	0.0018	-0.00043	0.00177
rs2554835	A	0.0088	0.0014	-0.00136	0.00142
rs2568955	T	-0.0165	0.0016	0.003917	0.001683
rs2569041	T	0.0077	0.0014	-0.00093	0.001417
rs2570497	T	-0.0121	0.0015	0.006187	0.001447
rs2588959	T	-0.008	0.0014	0.008291	0.001388
rs2657283	T	0.0085	0.0014	-0.00318	0.001407
rs2665668	A	-0.0094	0.0015	0.003801	0.001458
rs2702576	A	-0.009	0.0014	-0.00032	0.00143
rs2718277	T	0.0142	0.0026	-0.00819	0.002463
rs2718791	T	-0.0081	0.0015	0.005771	0.001447
rs27220	A	-0.0114	0.0015	0.002454	0.001436
rs2725370	T	-0.0141	0.0015	0.004252	0.001512
rs2736752	T	0.0095	0.0017	0.000446	0.001694
rs2740795	A	0.0098	0.0016	-0.00679	0.00158
rs2761438	A	0.0098	0.0015	-0.00345	0.001428
rs2764684	T	0.0143	0.0019	-0.00339	0.001816
rs2805064	C	0.0093	0.0016	-0.00151	0.001549
rs2838006	T	0.0132	0.0015	-0.00564	0.001453
rs28482086	A	0.0086	0.0015	-0.00252	0.001464
rs28505285	C	0.0106	0.0018	-0.00681	0.001803
rs28512462	C	0.0112	0.0014	-0.00777	0.001436
rs28513882	A	-0.011	0.0018	-6.98E-05	0.001794
rs2852349	T	-0.0093	0.0014	0.003356	0.001394
rs28587776	T	0.0088	0.0015	-0.00225	0.001445
rs28669886	A	-0.0084	0.0015	0.000164	0.001449
rs2870281	A	-0.0083	0.0014	-0.0002	0.001396
rs28735993	A	0.0162	0.0022	-0.00022	0.002176
rs2885198	A	0.0088	0.0014	0.000974	0.001399
rs2898191	A	0.0093	0.0015	0.001154	0.001494
rs2910823	T	0.0115	0.0014	-0.00142	0.001407
rs2916490	A	-0.0092	0.0015	0.001199	0.00153

rs2923424	A	0.012	0.0014	-0.00544	0.001419
rs2926702	T	-0.0124	0.0021	-0.00345	0.002091
rs2929032	A	-0.0081	0.0014	0.000303	0.00139
rs2929860	T	0.011	0.0018	-0.00516	0.001747
rs2942884	A	-0.009	0.0014	0.002273	0.001394
rs2954114	A	-0.0081	0.0014	0.003703	0.001392
rs2958182	A	0.0082	0.0015	-0.00333	0.001474
rs2962378	A	0.0101	0.0017	0.002195	0.001681
rs2964199	T	-0.0099	0.0015	-0.0004	0.001492
rs2964255	A	0.0088	0.0015	0.00199	0.0015
rs2976397	T	0.0081	0.0014	-0.00043	0.001402
rs2977464	T	0.0102	0.0018	-0.00109	0.00178
rs29792	A	-0.0093	0.0015	-0.00015	0.001513
rs2980813	A	0.0088	0.0014	-0.00333	0.001397
rs2989476	C	0.0078	0.0014	-0.0017	0.001398
rs2989751	A	-0.0092	0.0017	0.002195	0.00163
rs2992037	A	0.0149	0.0015	-0.00657	0.001503
rs2994326	T	-0.01	0.0018	0.001685	0.001793
rs2998299	T	-0.0136	0.0017	0.001446	0.001694
rs3026996	A	0.0138	0.0016	-0.00357	0.001615
rs303752	A	-0.011	0.0014	-0.00117	0.00143
rs3111251	T	-0.009	0.0014	0.001669	0.001402
rs312927	A	-0.0147	0.0022	0.00353	0.002165
rs312945	A	-0.0091	0.0015	0.002025	0.001512
rs317050	T	0.0078	0.0014	-0.00843	0.001387
rs322614	A	-0.0087	0.0014	0.003155	0.001425
rs322744	T	-0.009	0.0016	0.00072	0.001574
rs324885	A	0.0142	0.0014	-0.00468	0.00139
rs32940	T	-0.0096	0.0015	0.004292	0.001517
rs339057	A	-0.0081	0.0014	0.00036	0.001404
rs34067381	T	-0.0104	0.0015	-0.00064	0.001452
rs34098770	A	-0.016	0.002	0.001499	0.001933
rs34106693	C	0.0159	0.0019	-0.00349	0.001883
rs34122915	C	0.0084	0.0014	-0.00174	0.001435
rs341504	A	0.0099	0.0015	0.000367	0.001447
rs34155847	A	0.0135	0.0016	-0.00411	0.001623
rs34262657	T	-0.024	0.0038	0.000515	0.003965
rs34286836	T	-0.0089	0.0014	0.002778	0.001399
rs34298584	A	0.0111	0.0019	-0.00463	0.001847
rs34305371	A	0.0314	0.0024	-0.00446	0.002293
rs34309	A	0.008	0.0014	-0.00105	0.001433
rs34316274	A	0.0097	0.0017	-0.00071	0.001619
rs34363861	A	0.0087	0.0014	-0.00289	0.001423
rs34394051	A	-0.0117	0.002	0.003291	0.001901
rs34410	C	-0.0083	0.0014	0.000931	0.001391
rs34624793	T	0.0116	0.0021	-0.00473	0.002016

rs34720381	T	-0.018	0.0024	0.006627	0.002406
rs34748029	A	0.0185	0.0025	-0.00444	0.002416
rs347661	T	-0.0081	0.0014	0.002259	0.001411
rs34780702	A	0.0111	0.0017	-0.0048	0.001622
rs34807077	A	0.0128	0.0019	-0.00613	0.001878
rs34811474	A	0.0104	0.0017	-0.00485	0.001644
rs34967082	A	-0.008	0.0014	0.001858	0.001409
rs35016816	T	0.0176	0.0023	-0.00038	0.002323
rs350281	T	-0.0117	0.0015	0.004729	0.001482
rs35104491	A	0.0124	0.0018	-0.0008	0.001774
rs35111506	C	-0.0175	0.003	0.00578	0.00299
rs35192107	T	-0.0091	0.0017	0.003766	0.00163
rs35319653	T	0.0119	0.0015	-0.00729	0.001462
rs35417702	T	-0.0152	0.0014	-0.00385	0.001387
rs35493937	C	0.0124	0.0017	0.004117	0.001662
rs35606437	A	0.0105	0.0016	-0.00202	0.001571
rs356999	A	-0.0101	0.0014	0.003502	0.00143
rs35745455	A	0.008	0.0014	0.00125	0.001384
rs35751693	T	-0.0246	0.004	0.004688	0.003702
rs35754740	T	0.0083	0.0014	-0.00523	0.001407
rs35811586	T	0.0217	0.0031	-0.00253	0.003004
rs35929923	A	-0.0114	0.0016	0.002826	0.001596
rs36085856	C	0.0201	0.0036	-0.00356	0.002738
rs36120534	T	0.01	0.0018	-9.18E-05	0.00178
rs362307	T	-0.0226	0.0027	0.007062	0.002655
rs363096	T	-0.0143	0.0014	0.004495	0.001403
rs3735478	T	0.0108	0.0016	-0.00608	0.001526
rs3751331	A	-0.0097	0.0014	0.004593	0.001433
rs3766979	A	0.0085	0.0015	0.003276	0.001477
rs3768480	C	0.0101	0.0014	-0.00333	0.001392
rs3781339	T	-0.0104	0.0018	0.003446	0.001751
rs3790609	T	0.0107	0.0019	-0.00657	0.00181
rs3809169	T	0.014	0.0025	0.002099	0.002439
rs3809634	A	-0.0109	0.0015	0.003207	0.001508
rs3812281	T	0.0113	0.0014	-0.00415	0.001414
rs3817923	A	-0.0121	0.0022	0.001573	0.00218
rs382196	T	-0.0082	0.0014	0.003295	0.00143
rs3847228	T	0.0083	0.0014	-0.00152	0.001415
rs3848715	A	-0.008	0.0014	-0.00092	0.001388
rs3859523	T	0.0124	0.0018	-0.00382	0.001809
rs387027	A	-0.0083	0.0015	0.003498	0.001454
rs3895736	A	0.0144	0.0019	-0.00277	0.001819
rs3897821	A	0.0154	0.0015	-0.00054	0.001467
rs3948495	T	-0.0089	0.0014	0.005169	0.001411
rs399821	A	-0.0078	0.0014	0.001247	0.001401
rs39998	A	0.009	0.0015	0.001737	0.00153

rs401526	T	-0.0104	0.0014	0.00138	0.001389
rs401966	C	-0.0097	0.0014	0.003969	0.00143
rs4127499	A	0.0087	0.0015	-0.00193	0.001445
rs41282553	A	0.0237	0.004	-0.00338	0.003928
rs42210	C	-0.0098	0.0016	0.00602	0.001538
rs42302	A	0.0086	0.0015	-0.00357	0.001464
rs4255791	A	-0.0084	0.0015	0.003447	0.001475
rs4263475	A	0.0078	0.0014	-0.00246	0.001425
rs4283754	A	-0.0084	0.0014	0.000308	0.001469
rs429150	T	0.0085	0.0014	-0.00248	0.001389
rs4298514	T	0.0104	0.0015	-0.00107	0.001517
rs4320563	A	0.0109	0.0014	-0.00256	0.001388
rs4328757	T	0.0096	0.0014	-0.00177	0.001422
rs4352658	T	-0.019	0.0026	0.011661	0.002542
rs4358081	A	-0.0095	0.0014	0.005913	0.001385
rs4358358	A	0.0109	0.0015	-0.00066	0.001507
rs4369924	A	0.0125	0.0019	-0.00127	0.001892
rs4384309	A	0.0102	0.0014	-0.00432	0.0014
rs4396896	A	-0.0107	0.0014	0.002952	0.00142
rs4423373	A	-0.0087	0.0014	0.00106	0.001392
rs4426420	T	-0.014	0.0019	0.003034	0.001894
rs4434676	A	-0.0089	0.0014	-0.00039	0.001397
rs4458044	C	0.0101	0.0016	0.002028	0.001611
rs4467547	T	0.0121	0.0014	-0.00497	0.001411
rs4469771	T	0.0101	0.0015	0.0014	0.001479
rs4480339	A	-0.0097	0.0015	0.003338	0.001496
rs4490539	A	0.0142	0.0015	-0.00504	0.001505
rs4497562	A	0.0112	0.0016	-0.00249	0.001587
rs4500930	T	-0.0109	0.0015	0.001663	0.001456
rs4500960	T	-0.0131	0.0014	0.007163	0.001388
rs4502401	T	0.0085	0.0015	-0.00333	0.001473
rs4652135	A	0.0103	0.0016	-0.00356	0.001556
rs4658019	T	0.0084	0.0014	0.000182	0.00139
rs4663617	A	0.0104	0.0017	-0.00072	0.001654
rs4664983	T	-0.0103	0.0018	-0.00101	0.001744
rs4673840	T	-0.0138	0.0019	-9.24E-05	0.001885
rs4675248	A	-0.0094	0.0014	0.009221	0.001417
rs4685405	T	-0.0111	0.0018	0.006954	0.00183
rs4687735	T	-0.02	0.0033	0.010081	0.003194
rs4691601	A	-0.0115	0.0014	0.001769	0.00139
rs4705763	C	0.0089	0.0014	-0.00346	0.001389
rs4712371	A	-0.0124	0.0021	0.004953	0.002127
rs4719460	T	-0.0088	0.0015	0.000189	0.001461
rs4719944	T	-0.0121	0.0014	0.005313	0.001395
rs4724083	T	0.0083	0.0015	-0.00113	0.001501
rs4726070	A	0.0122	0.0014	-0.00141	0.001418

rs4730020	T	0.009	0.0016	-0.002	0.00157
rs4731413	A	0.0111	0.0017	-0.0015	0.001705
rs4735297	A	-0.0086	0.0015	0.008012	0.00149
rs4739235	A	0.0109	0.0019	-0.00616	0.001868
rs4741571	A	-0.0102	0.0015	0.002544	0.001496
rs4757957	C	0.0133	0.0015	-0.00269	0.001489
rs4766424	C	0.0142	0.0021	-0.0045	0.002078
rs4778058	T	-0.0092	0.0014	0.001367	0.00141
rs4785187	A	-0.01	0.0017	0.008344	0.001667
rs4787028	T	0.0086	0.0015	0.000499	0.001465
rs4787457	A	0.0162	0.0015	-0.00246	0.001431
rs4788115	A	0.0111	0.0019	-0.00259	0.001864
rs479018	A	0.0109	0.0015	-0.00362	0.001495
rs4793090	A	0.0084	0.0015	-0.00473	0.001461
rs4810894	A	-0.0085	0.0015	-0.0006	0.00144
rs4812697	T	0.0171	0.0027	-0.00238	0.00266
rs481940	T	0.01	0.0016	-0.00161	0.001571
rs482787	T	0.01	0.0015	-0.00251	0.001481
rs4839155	T	0.0115	0.0017	-0.00439	0.001635
rs4846010	A	0.0099	0.0018	0.000396	0.001737
rs4846724	A	0.0098	0.0014	-0.00187	0.001393
rs4848924	A	-0.0117	0.0015	0.002394	0.001541
rs4850954	T	0.0077	0.0014	-0.00021	0.001398
rs4851263	A	-0.0151	0.0023	0.002084	0.002272
rs4877516	A	-0.0101	0.0014	0.00853	0.001396
rs4881269	A	0.01	0.0014	-0.00051	0.001431
rs488476	C	-0.0113	0.0015	0.005229	0.001424
rs4894658	C	0.0091	0.0016	-0.00549	0.001585
rs4895650	T	0.0093	0.0014	-0.00478	0.001407
rs4899012	C	-0.0103	0.0014	0.002855	0.001419
rs4904523	A	-0.0082	0.0014	0.002084	0.001388
rs4915735	A	0.0116	0.002	-0.00032	0.001986
rs4919624	A	0.0193	0.0018	-0.00712	0.001719
rs4925065	T	-0.0078	0.0014	0.000162	0.001389
rs4925109	A	-0.0096	0.0015	0.002089	0.001511
rs4938815	T	0.0085	0.0015	-0.0016	0.001516
rs4941735	T	0.0092	0.0014	-0.00315	0.001402
rs4972748	T	0.0105	0.0018	-0.00526	0.001795
rs4977885	A	-0.0084	0.0014	0.004335	0.001416
rs4981245	A	0.008	0.0014	6.51E-05	0.001402
rs4984541	A	-0.0138	0.0017	0.004448	0.001658
rs4984613	T	0.0174	0.0028	0.001602	0.002858
rs4984682	C	-0.0138	0.0017	0.008582	0.001663
rs548897	A	0.0081	0.0014	-0.00337	0.001397
rs55675587	T	-0.0103	0.0018	0.003789	0.001774
rs55736314	C	-0.0154	0.0014	0.005302	0.001416

rs55826493	T	-0.0127	0.0021	0.002858	0.002091
rs55986781	T	0.0187	0.0026	-0.00836	0.002455
rs56085180	A	-0.0269	0.0037	0.000656	0.003622
rs56099375	T	0.012	0.0017	-0.00665	0.001625
rs56171318	T	-0.0137	0.002	0.002248	0.00198
rs56174996	A	-0.0143	0.002	0.005625	0.002032
rs56306882	A	0.0086	0.0016	-0.00576	0.001566
rs56319902	T	-0.0206	0.0017	-0.00577	0.001661
rs56391344	A	0.0148	0.0016	-0.00725	0.001595
rs563954	A	-0.0081	0.0014	0.005409	0.00139
rs56405138	A	0.0153	0.0022	-0.00832	0.002125
rs56408528	T	-0.0082	0.0014	-0.00198	0.001389
rs567003	A	0.0094	0.0015	-0.00108	0.00146
rs56794817	A	0.0122	0.0019	-0.00095	0.001912
rs57016874	T	0.0209	0.0037	-0.00379	0.003532
rs57148205	A	0.0095	0.0016	-0.0023	0.001637
rs57204268	A	0.0114	0.002	-0.00283	0.001931
rs57349798	A	0.0093	0.0014	-0.0076	0.001423
rs57352738	A	-0.0152	0.0017	0.007648	0.001711
rs57437407	A	-0.0145	0.0023	0.005304	0.002211
rs575113	A	0.0133	0.0015	-0.00147	0.001533
rs5754581	T	-0.0082	0.0014	-0.00148	0.001398
rs5754753	T	-0.0126	0.0016	0.000462	0.001532
rs5754762	A	0.0197	0.003	-0.011	0.003061
rs57661533	T	-0.0114	0.0021	0.002373	0.002056
rs580652	T	-0.0147	0.0024	0.001236	0.002331
rs585557	A	0.0128	0.0018	-0.0037	0.001738
rs58779949	A	0.011	0.0019	-0.00601	0.001812
rs58859557	T	0.0187	0.0028	-0.00941	0.002716
rs58921703	T	0.0109	0.0015	-0.00291	0.001461
rs58950082	T	0.0095	0.0017	-0.00262	0.001712
rs58996896	A	-0.0094	0.0016	0.007186	0.001567
rs590013	T	0.01	0.0015	-0.00422	0.001482
rs59300999	T	-0.0164	0.0028	0.006788	0.002774
rs59480703	C	-0.0123	0.0018	0.005694	0.00175
rs59813324	T	0.0097	0.0018	-0.00186	0.001739
rs59967356	T	-0.0127	0.0021	0.00186	0.002071
rs60096640	A	0.0159	0.0023	-0.00634	0.002288
rs6020560	T	-0.0083	0.0014	0.004347	0.001391
rs6043521	T	-0.0083	0.0014	-0.00016	0.001432
rs60589532	A	0.0168	0.0028	-0.01353	0.002929
rs6060308	A	0.0095	0.0016	-0.00625	0.00158
rs6065080	T	-0.013	0.0015	-0.00095	0.001451
rs6065784	C	0.011	0.0015	-0.00336	0.001529
rs60717745	C	-0.0118	0.0019	0.000625	0.001923
rs60726488	T	-0.009	0.0016	-0.00176	0.001584

rs6091570	A	0.0081	0.0015	-0.00033	0.001468
rs61104616	A	-0.0172	0.0014	0.002495	0.001386
rs6123924	A	0.0129	0.0019	-0.00066	0.001926
rs613872	T	-0.0163	0.0019	0.005204	0.001829
rs61387839	A	0.0088	0.0015	0.0018	0.001499
rs61527214	A	0.011	0.0014	-0.00199	0.001416
rs61739710	A	-0.0099	0.0016	0.000629	0.001546
rs61748951	A	-0.0273	0.0048	0.01185	0.004661
rs61755388	T	0.0125	0.0017	-0.00036	0.001684
rs61757207	A	0.0359	0.0063	-0.01124	0.005729
rs61798586	A	-0.0112	0.002	-0.00119	0.00195
rs61853335	T	-0.0109	0.0018	0.008161	0.001822
rs61958175	A	-0.0198	0.0034	0.015883	0.003248
rs61997667	T	-0.0144	0.002	0.005451	0.001925
rs62007304	A	0.0264	0.0047	-0.01018	0.004274
rs62018216	T	0.0113	0.0019	-0.00414	0.001922
rs62051146	A	-0.0134	0.0022	0.003659	0.002176
rs62090515	A	-0.009	0.0015	0.000622	0.001446
rs62092949	T	-0.0087	0.0014	-0.00152	0.001392
rs62103198	T	-0.0147	0.0025	0.007861	0.002426
rs62109862	A	0.0136	0.002	-0.00151	0.002005
rs62142891	A	0.0108	0.0016	-0.00376	0.001576
rs62155350	A	0.0338	0.0053	-0.00158	0.00498
rs62155770	A	-0.0196	0.0032	0.005872	0.003001
rs62155873	T	-0.0151	0.0021	0.016875	0.002092
rs62174974	A	-0.0105	0.0018	0.002205	0.001742
rs62177359	A	-0.0275	0.0038	0.020162	0.00382
rs62179650	A	0.0112	0.0016	-0.00798	0.001504
rs62182994	T	0.0137	0.0015	-0.00577	0.001489
rs62190914	T	0.0089	0.0015	0.002666	0.001445
rs62194170	A	0.0111	0.0017	-0.0037	0.001732
rs622169	T	0.0086	0.0014	0.000759	0.001452
rs62247449	C	0.0114	0.0014	-0.00113	0.001401
rs62252819	A	-0.0094	0.0017	0.003213	0.001633
rs62256284	T	0.0111	0.0017	0.001444	0.0017
rs62260764	C	-0.0132	0.0016	0.005694	0.001573
rs62262671	A	0.0213	0.002	-0.01043	0.002009
rs62340636	T	0.0103	0.0014	-0.00234	0.00144
rs62370510	A	-0.0109	0.0019	-0.00037	0.001817
rs62379838	T	0.0121	0.0015	0.000361	0.001509
rs62409395	T	0.0101	0.0018	-0.00276	0.001722
rs62420387	T	-0.0184	0.0027	0.003624	0.002616
rs62506074	T	0.0091	0.0015	-0.00138	0.001469
rs62506104	A	-0.0098	0.0018	0.001131	0.001775
rs628993	A	0.0128	0.0023	-0.00294	0.002354
rs631287	A	0.0084	0.0014	-0.00897	0.001394

rs633279	A	-0.0089	0.0015	-0.00221	0.001523
rs635754	A	0.0119	0.0014	-0.00071	0.00141
rs6428587	T	0.0084	0.0015	-0.00333	0.001442
rs6435326	A	-0.0091	0.0014	0.002253	0.00139
rs6442126	A	-0.0121	0.0015	0.006837	0.001464
rs6445633	A	0.0088	0.0015	0.000865	0.001498
rs6449503	A	0.0185	0.0014	-0.00611	0.001395
rs6450476	A	-0.0116	0.0015	-0.00268	0.001524
rs6452793	T	0.0175	0.0017	-0.00454	0.001662
rs6461536	A	0.0088	0.0015	-0.00106	0.001535
rs6469654	C	-0.0103	0.0017	0.004153	0.001701
rs6472208	T	-0.0091	0.0014	-0.00351	0.001413
rs6480234	T	-0.0084	0.0014	0.003113	0.00139
rs648044	A	0.0083	0.0015	-0.00467	0.001455
rs6490618	T	0.0088	0.0015	-0.00116	0.00148
rs6493265	T	-0.0122	0.0014	0.009167	0.001417
rs6493275	A	0.0131	0.0017	-0.01624	0.001698
rs6503409	T	0.0087	0.0015	-0.00014	0.001453
rs6534338	T	0.0103	0.0015	-0.00701	0.001525
rs6543810	T	0.0082	0.0015	0.001302	0.001426
rs6557171	T	-0.0155	0.0015	0.005658	0.001464
rs6567288	A	-0.0079	0.0014	0.002612	0.001404
rs6569077	T	0.0118	0.0014	-0.00302	0.001483
rs6573552	T	-0.009	0.0014	0.000667	0.001387
rs6573559	T	0.0105	0.0015	0.002118	0.001495
rs660001	A	-0.0164	0.0017	0.001337	0.001699
rs663251	T	0.0081	0.0015	-0.00324	0.001461
rs66482320	C	0.0124	0.0022	-0.00167	0.002212
rs66568921	T	-0.0181	0.0015	0.007452	0.001458
rs66671632	T	-0.0133	0.0021	0.00392	0.002082
rs66721975	A	-0.0087	0.0015	0.000749	0.001544
rs6672986	C	0.0094	0.0014	-0.00405	0.001392
rs6689641	A	0.0078	0.0014	-0.00356	0.00139
rs6690195	T	-0.0113	0.0014	0.007175	0.00139
rs6695132	T	0.0097	0.0017	-0.00202	0.001682
rs6696068	T	-0.0088	0.0014	-0.00083	0.001424
rs6697584	T	-0.0126	0.0017	0.002318	0.00168
rs6704768	A	-0.0116	0.0014	-0.00194	0.001398
rs6706275	T	0.0083	0.0015	0.00143	0.001471
rs6711399	T	-0.0107	0.0019	0.000568	0.001824
rs6715321	T	-0.0097	0.0014	0.002996	0.0014
rs6715849	A	-0.0122	0.0014	0.002142	0.001398
rs6720515	A	0.0103	0.0017	-0.00118	0.001673
rs67224963	A	0.0113	0.0017	-0.00076	0.001728
rs6729612	T	0.0092	0.0015	0.002053	0.001485
rs6731373	A	-0.0115	0.0015	-0.00464	0.001443

rs6736025	T	0.0079	0.0014	-0.00267	0.001397
rs6738860	A	-0.0088	0.0014	0.00776	0.001395
rs6743032	A	-0.0165	0.0024	0.002839	0.002358
rs6744040	A	-0.008	0.0014	0.003935	0.001395
rs67456868	A	0.0119	0.0018	-0.00064	0.001783
rs6752228	T	-0.008	0.0014	0.004266	0.001389
rs6757087	T	-0.0085	0.0014	0.003863	0.001412
rs67661275	A	0.0134	0.0025	0.000738	0.002458
rs6774533	T	0.009	0.0015	-0.00117	0.001545
rs67790232	A	0.0101	0.0017	-0.0044	0.00172
rs6780414	T	0.0105	0.0018	0.000504	0.001794
rs6792805	A	-0.009	0.0016	0.003869	0.001561
rs6812533	T	0.0106	0.0016	-0.00499	0.001602
rs68145588	T	-0.0127	0.0021	0.002519	0.001964
rs6824567	T	0.013	0.0016	-0.00907	0.001541
rs6853599	T	0.0108	0.0019	-0.00463	0.001814
rs6861925	C	-0.009	0.0014	0.004547	0.001395
rs6871635	A	-0.0087	0.0014	0.00777	0.001409
rs6881581	A	-0.0096	0.0015	0.001345	0.001468
rs6881733	T	-0.0086	0.0016	-0.0007	0.001543
rs6917154	T	0.0127	0.0021	-0.00486	0.002079
rs6917204	T	0.0119	0.0017	-0.00307	0.001739
rs6918506	C	0.0113	0.0014	-0.00583	0.001389
rs6924023	A	0.0107	0.0016	-0.0043	0.001627
rs6932108	C	-0.021	0.0027	0.006206	0.002493
rs6946136	A	0.0081	0.0014	-0.00113	0.001417
rs6946362	T	0.0091	0.0015	-0.00278	0.001528
rs6951996	A	-0.0182	0.0032	0.0044	0.003171
rs6956283	T	0.0098	0.0016	-0.00367	0.001564
rs6969783	A	-0.0098	0.0014	0.010167	0.001396
rs6977237	T	0.0082	0.0014	-0.00168	0.001389
rs6989141	A	0.01	0.0017	-0.00319	0.001742
rs6994287	A	0.0102	0.0014	-0.00094	0.001406
rs7012546	T	0.0092	0.0014	-8.64E-05	0.001406
rs7016302	C	-0.0125	0.0019	-0.00064	0.001849
rs702606	T	0.0118	0.0021	-0.00219	0.002029
rs7029718	A	0.0244	0.0014	-0.00359	0.001407
rs7030373	A	-0.0098	0.0017	0.002187	0.00172
rs7035315	A	-0.009	0.0015	0.001277	0.001442
rs7040995	C	0.0097	0.0014	-0.0023	0.001401
rs7041702	A	-0.0111	0.0016	0.003211	0.001574
rs7084508	T	-0.0085	0.0015	0.003102	0.001456
rs710629	A	0.0082	0.0015	0.003499	0.001459
rs7108020	A	0.0111	0.0015	-0.00336	0.001449
rs711793	T	0.0103	0.0015	0.000836	0.001491
rs7127580	C	-0.015	0.0023	0.002757	0.002214

rs713584	A	-0.008	0.0014	0.001336	0.001405
rs71413877	A	0.0351	0.0036	-0.00524	0.003575
rs7146625	A	0.0091	0.0016	-0.00109	0.001577
rs7147473	A	-0.0103	0.0015	0.003689	0.001482
rs7158218	A	-0.009	0.0015	0.001212	0.001521
rs71646142	T	0.0104	0.0018	-0.0015	0.00177
rs716513	A	0.0084	0.0014	-0.00501	0.001392
rs7167688	T	-0.0094	0.0014	0.000246	0.00139
rs7171405	A	-0.0101	0.0016	0.002002	0.001632
rs717996	T	-0.0122	0.0014	0.006343	0.001421
rs7182216	C	-0.0096	0.0017	-0.00145	0.001656
rs7190	A	-0.0095	0.0015	0.004287	0.001447
rs721579	T	-0.011	0.0016	-0.00158	0.001586
rs7218235	A	-0.0097	0.0017	0.006354	0.001714
rs7223311	C	-0.0079	0.0014	0.000104	0.001395
rs7226824	T	-0.0078	0.0014	0.001536	0.001388
rs72482130	T	0.0141	0.0019	-0.00821	0.001942
rs7254263	T	-0.0119	0.0016	2.09E-05	0.001535
rs7255223	A	0.0097	0.0016	-0.00243	0.001572
rs72622559	T	-0.012	0.0017	0.003695	0.001784
rs72624911	T	0.0282	0.0034	-0.00551	0.003332
rs72636697	T	-0.014	0.002	0.007118	0.002048
rs72667460	T	0.0197	0.0031	-0.00653	0.003037
rs72671456	A	-0.0174	0.002	0.002443	0.002029
rs72672052	A	-0.0101	0.0018	0.002664	0.001838
rs72673097	A	0.0257	0.0042	0.002756	0.003835
rs72677177	A	0.01	0.0014	-0.0046	0.001415
rs72694479	T	0.0127	0.002	0.000632	0.001967
rs72709560	A	0.0085	0.0015	-0.00412	0.001477
rs72771860	T	-0.0211	0.0029	0.003233	0.002902
rs72792395	T	-0.0145	0.0022	0.001619	0.002193
rs728054	A	-0.0128	0.0015	-0.0003	0.001451
rs72819118	T	-0.0231	0.0024	0.000632	0.002371
rs72824753	T	0.0133	0.0024	-0.00289	0.00227
rs72828517	T	-0.0168	0.0019	-0.00032	0.00181
rs72829857	A	-0.0149	0.0016	0.004388	0.001639
rs72834698	A	0.0117	0.002	-0.00641	0.001987
rs72881110	A	0.0146	0.0027	-0.00229	0.002617
rs72883760	A	0.0101	0.0018	-0.00589	0.001741
rs72896637	T	0.0162	0.0028	-0.00387	0.002633
rs72902523	T	-0.0103	0.0016	-0.00173	0.001606
rs72906124	T	0.0185	0.003	-0.0043	0.003014
rs72917504	T	-0.0178	0.003	0.002912	0.002936
rs72919450	T	-0.0132	0.0023	7.82E-06	0.002293
rs72944064	T	-0.0098	0.0016	0.001077	0.001592
rs72962169	T	-0.0174	0.0019	0.007546	0.001905

rs72972965	A	0.0083	0.0015	-0.00226	0.00145
rs72993796	T	0.0127	0.0022	-0.00158	0.00216
rs73034295	A	0.0099	0.0018	-0.004	0.001783
rs730384	A	0.0101	0.0014	-0.00167	0.001405
rs73039077	C	0.0092	0.0016	-0.00613	0.001587
rs73055556	A	0.0111	0.002	-0.00555	0.002004
rs73082325	C	-0.0252	0.0044	0.007511	0.00409
rs73106136	T	-0.0147	0.0026	0.000864	0.002578
rs73154546	A	0.0197	0.0036	0.003429	0.003508
rs7317761	C	0.0109	0.0016	-0.00802	0.001581
rs73191311	A	-0.0094	0.0015	-0.00184	0.001474
rs7321274	A	0.0114	0.0018	-0.00356	0.001727
rs73219806	A	0.0106	0.0019	-0.00718	0.0019
rs7323027	A	0.013	0.0015	-0.0052	0.001453
rs7326331	A	-0.0128	0.0016	0.001651	0.001557
rs73344830	A	0.017	0.0014	-0.00494	0.001407
rs73405293	A	0.0113	0.002	-0.00607	0.001958
rs73457936	A	0.0144	0.0025	-0.00479	0.002454
rs7356536	T	-0.0129	0.0021	0.000415	0.002014
rs73581580	A	-0.0181	0.0022	0.00779	0.002112
rs736281	T	0.0086	0.0014	0.000472	0.001418
rs73643713	T	-0.0165	0.0027	0.000964	0.002666
rs736471	T	0.0083	0.0014	0.000482	0.0014
rs73648455	T	-0.0183	0.0026	0.006131	0.002591
rs737945	C	-0.0115	0.0014	-0.00081	0.001394
rs73874335	T	-0.0178	0.0029	0.003291	0.003027
rs73961845	A	-0.0114	0.0014	0.000626	0.001427
rs74091672	A	-0.0153	0.0022	0.00475	0.002134
rs7430651	T	-0.0111	0.0016	0.001454	0.001518
rs743316	T	0.0112	0.0017	0.000818	0.001706
rs74415461	T	0.0176	0.0026	-0.01029	0.002484
rs74453875	A	0.0214	0.0037	-0.00241	0.003722
rs74462621	T	0.0125	0.002	-0.0075	0.002029
rs7449561	A	0.0102	0.0017	-0.00478	0.001652
rs7451726	A	0.0109	0.0017	-0.0002	0.001707
rs74545339	A	-0.0158	0.0022	0.001394	0.00221
rs7460106	T	-0.0123	0.0017	-0.0013	0.001656
rs74615093	A	0.0145	0.0026	-0.0007	0.002551
rs746839	C	0.0135	0.0015	-0.00141	0.001454
rs74747621	T	0.0153	0.0022	-0.0019	0.002215
rs74787922	A	0.0182	0.0028	-0.00615	0.00273
rs74944275	T	0.0231	0.0035	-0.00514	0.003339
rs74944857	T	-0.0089	0.0015	-0.00158	0.001474
rs7495033	A	0.0095	0.0015	-0.00456	0.001529
rs75033012	C	-0.0258	0.0038	-0.00206	0.003566
rs75177132	T	0.0298	0.0036	-0.01546	0.003254

rs75203411	T	0.0135	0.0019	-0.0017	0.001853
rs7526112	T	0.0124	0.0015	0.000882	0.00144
rs75308819	A	0.0287	0.0047	0.002466	0.004644
rs75434274	A	0.0151	0.0026	-0.0043	0.002473
rs75500877	T	0.0207	0.0033	0.008457	0.00324
rs7552964	A	0.0148	0.0023	-0.0046	0.00234
rs7560871	A	-0.0193	0.0027	0.003504	0.002717
rs7561705	A	0.0078	0.0014	0.002862	0.001388
rs7575637	A	0.0107	0.0014	-0.00383	0.001389
rs75756843	A	0.0288	0.0049	-0.00992	0.005127
rs7575938	A	0.009	0.0015	-0.00265	0.001467
rs7590368	T	-0.0146	0.0016	-5.10E-05	0.00158
rs7597126	T	-0.0094	0.0014	-0.00299	0.001391
rs7597412	C	0.0088	0.0015	-0.00149	0.001442
rs7603132	A	0.0137	0.0018	-0.00404	0.001744
rs76076331	T	0.0206	0.0021	-0.00479	0.002117
rs76077165	A	0.0165	0.0025	-0.00774	0.002516
rs76167224	T	-0.0187	0.0033	0.003298	0.003134
rs7617204	A	-0.0097	0.0014	-0.00164	0.001387
rs76235882	A	0.0241	0.0039	-0.0095	0.004136
rs76241605	A	0.0139	0.0022	-0.0021	0.002167
rs76246107	A	-0.0162	0.0026	-0.00679	0.002618
rs7625428	T	0.0098	0.0014	-0.00268	0.001416
rs76267866	A	0.0104	0.0017	0.000711	0.001712
rs7630133	A	0.0082	0.0015	-0.00101	0.001457
rs763553	A	0.0087	0.0014	0.000991	0.001396
rs7640424	T	0.0113	0.0015	-0.00845	0.0015
rs7650602	T	-0.0089	0.0014	0.001206	0.001401
rs76552497	T	-0.012	0.0019	0.00602	0.001849
rs76577427	C	0.0203	0.0024	-0.00266	0.002257
rs7672622	A	-0.0088	0.0016	0.001225	0.0016
rs767943	A	-0.0122	0.0016	0.004641	0.001581
rs7683416	T	0.0139	0.0014	0.003507	0.001395
rs76876592	A	0.0126	0.002	-0.0005	0.002027
rs76878669	C	0.0141	0.0017	-0.00012	0.001645
rs7692359	T	-0.0109	0.0017	0.001135	0.001685
rs76957677	T	-0.0297	0.0054	0.011942	0.004998
rs77025239	A	-0.014	0.0019	0.001923	0.001931
rs7714719	C	0.0116	0.002	-0.0056	0.001985
rs7716161	C	0.0105	0.0019	-0.00167	0.001826
rs77201694	A	0.0156	0.0022	-0.00541	0.002149
rs77370942	A	-0.0147	0.0027	0.005794	0.002607
rs77554090	T	-0.0186	0.0027	0.00593	0.00264
rs77609760	A	0.0236	0.0028	-0.00273	0.002778
rs7766240	T	-0.0134	0.0024	0.005069	0.002358
rs77702622	A	-0.0213	0.0029	0.003886	0.00281

rs77702819	T	0.0187	0.0025	-0.00336	0.002407
rs77719387	A	-0.0403	0.0058	0.007003	0.005594
rs7772172	A	0.0091	0.0014	-2.63E-05	0.001413
rs7775100	T	-0.0087	0.0014	0.002445	0.001421
rs77826402	T	0.0151	0.0027	-0.00396	0.002701
rs7796103	C	-0.0077	0.0014	0.002003	0.001402
rs7799141	A	0.0099	0.0015	-0.00828	0.00149
rs77999825	A	0.0294	0.0039	-0.00092	0.00376
rs7803932	A	0.0127	0.0019	-0.00021	0.001855
rs780569	A	-0.0085	0.0016	-0.00033	0.001539
rs78116078	C	0.0086	0.0016	-0.0047	0.001534
rs7816777	T	-0.0088	0.0015	0.000304	0.001458
rs78193153	A	0.0191	0.0029	-0.00644	0.002873
rs7823700	T	0.0132	0.0024	-0.00197	0.002334
rs78365243	T	0.0181	0.0033	-0.0069	0.00321
rs78440611	A	-0.0147	0.0024	0.000135	0.002389
rs7849480	A	-0.0084	0.0014	0.00398	0.001403
rs7849487	T	-0.0167	0.0015	0.002538	0.001465
rs7855503	C	0.0088	0.0015	0.00104	0.001449
rs78648104	T	-0.0141	0.0025	0.008235	0.002475
rs78702390	C	0.0167	0.003	-0.00341	0.002955
rs78714229	T	0.0172	0.003	-0.00741	0.003004
rs7875078	A	-0.0078	0.0014	0.005057	0.001396
rs78918150	T	-0.008	0.0015	-4.61E-05	0.001431
rs7894722	T	0.0085	0.0014	-0.00564	0.00143
rs7899270	A	-0.0084	0.0015	-0.00015	0.001457
rs7905192	T	0.0084	0.0014	-0.00448	0.001394
rs790647	A	-0.0145	0.0017	0.005332	0.001644
rs7910403	T	0.0122	0.0018	-0.00488	0.001739
rs7914674	T	-0.0139	0.0022	0.007585	0.001718
rs7920624	A	0.012	0.0014	-0.00144	0.001395
rs7924036	T	0.0133	0.0014	-0.007	0.001386
rs79265434	A	-0.0197	0.0022	0.000486	0.002154
rs7928017	A	0.0096	0.0014	-0.00516	0.001399
rs7931563	T	-0.0109	0.0014	0.00372	0.001435
rs795230	T	0.0081	0.0014	0.001822	0.001402
rs7958371	A	-0.0085	0.0015	0.002871	0.001453
rs79585412	T	-0.0178	0.0031	0.005597	0.002993
rs7965154	A	-0.0085	0.0015	0.002559	0.001498
rs7967550	A	-0.0086	0.0014	-8.59E-05	0.00142
rs7972246	T	0.011	0.0015	-0.00411	0.001461
rs79728014	A	-0.0134	0.002	0.00708	0.001949
rs7974852	A	0.0117	0.0014	-0.00273	0.001386
rs7977614	A	-0.0134	0.0016	0.005783	0.001522
rs79798166	A	0.0172	0.0025	0.00034	0.002506
rs79855925	T	-0.0194	0.0033	0.004365	0.003345

rs79994730	T	0.024	0.0035	-0.01536	0.003212
rs79997166	A	0.0172	0.003	-0.00361	0.002913
rs80037907	T	-0.0107	0.0019	0.002256	0.001887
rs8008382	T	-0.0106	0.0015	-0.00016	0.001517
rs8009933	A	0.0088	0.0015	-0.00335	0.00149
rs8016504	A	-0.0087	0.0014	0.006423	0.001402
rs80223410	T	-0.0118	0.002	0.001208	0.00201
rs8024	A	-0.0115	0.0015	0.004813	0.001464
rs80257979	T	-0.0252	0.004	0.005916	0.00409
rs8030487	A	0.0085	0.0015	-0.00311	0.0015
rs803619	T	-0.0173	0.0023	0.004223	0.002311
rs8046072	A	0.0101	0.0018	-0.00329	0.001748
rs8052523	T	-0.0084	0.0014	0.001289	0.001415
rs8066044	A	0.009	0.0016	-0.00281	0.001571
rs806816	A	0.0101	0.0016	-0.00335	0.001605
rs8097125	T	0.0086	0.0014	-0.0029	0.001426
rs8103741	A	-0.0108	0.0019	0.001437	0.001816
rs818415	T	-0.011	0.0018	0.001821	0.001765
rs8192465	A	-0.0272	0.0049	-0.00285	0.004684
rs852771	T	0.0092	0.0015	-0.00039	0.001509
rs853286	T	0.0124	0.0023	-0.00348	0.002292
rs854796	A	0.009	0.0015	-6.56E-05	0.001496
rs868456	A	0.0089	0.0015	-0.00078	0.001524
rs870589	A	-0.0079	0.0014	0.004464	0.001392
rs884108	A	-0.0094	0.0017	0.000558	0.001645
rs891793	C	-0.0099	0.0014	-0.00089	0.001393
rs893522	A	0.0149	0.0025	-0.00101	0.002398
rs902820	A	-0.0096	0.0014	0.002419	0.001406
rs911149	T	0.0091	0.0017	0.002308	0.001636
rs912883	T	0.0087	0.0015	-0.00515	0.001487
rs913509	A	0.0096	0.0015	-0.00509	0.00147
rs925161	C	-0.0078	0.0014	0.003529	0.001388
rs9267658	T	0.0138	0.002	-0.00154	0.00196
rs9267677	T	0.018	0.0024	-0.00848	0.002348
rs9289300	T	-0.0164	0.0019	-0.00111	0.001901
rs9291437	C	-0.0081	0.0014	0.004139	0.001428
rs9294770	T	0.0083	0.0014	-0.00153	0.001435
rs929511	T	-0.016	0.0021	0.003354	0.002113
rs9300612	T	0.0084	0.0015	-0.00201	0.001514
rs933738	A	-0.011	0.0018	0.009068	0.001834
rs9349956	A	-0.0159	0.0018	0.003763	0.001786
rs9359939	A	-0.0105	0.0016	0.003897	0.001644
rs936496	A	-0.0079	0.0014	0.003381	0.001428
rs9371883	C	-0.0087	0.0015	-0.00011	0.001449
rs9373363	A	-0.0111	0.0016	0.004196	0.001607
rs9375188	T	0.0212	0.0014	-0.0005	0.001391

rs9375403	C	0.0126	0.0016	0.004135	0.001634
rs9386110	T	-0.0163	0.0023	0.002533	0.002239
rs9388490	T	0.0091	0.0014	-0.00454	0.001398
rs939400	T	-0.0095	0.0015	0.004514	0.001444
rs9411331	A	0.0145	0.0015	-0.00744	0.001488
rs9435340	A	0.0086	0.0015	-0.00835	0.001464
rs9442750	A	-0.0089	0.0016	0.003758	0.001578
rs9446060	A	0.0077	0.0014	-0.00211	0.001397
rs9465509	A	-0.0084	0.0014	0.001633	0.001387
rs9490512	A	-0.0136	0.0014	0.003482	0.001398
rs9492774	C	0.008	0.0015	0.002482	0.00144
rs9513416	A	-0.0116	0.0019	-0.00021	0.001902
rs9513754	T	0.0096	0.0016	-0.00544	0.00153
rs9527662	A	0.0094	0.0014	-0.0051	0.001435
rs9527905	A	-0.0094	0.0014	0.000543	0.001415
rs9529146	T	0.0127	0.0017	-0.00116	0.001656
rs9536462	A	0.0133	0.002	-0.00436	0.001986
rs9540718	A	-0.0089	0.0014	0.004755	0.00139
rs9545395	T	0.0138	0.0021	-0.00098	0.002076
rs9556958	T	-0.011	0.0014	0.001103	0.001396
rs9563168	A	0.0101	0.0017	-0.00275	0.001704
rs9568798	T	-0.0093	0.0016	0.000521	0.00154
rs9597907	T	0.0152	0.0027	-0.00346	0.002714
rs9611597	A	-0.0107	0.0019	-0.00283	0.001905
rs9616906	A	0.0122	0.0014	0.001616	0.001396
rs9616947	T	-0.0134	0.0015	0.002045	0.001433
rs9633970	T	0.0091	0.0016	-0.00087	0.001665
rs9649	T	0.0108	0.0019	-0.00572	0.001848
rs9655780	A	-0.0153	0.0019	0.002639	0.001869
rs9666728	T	-0.0082	0.0014	0.002435	0.001389
rs9683585	C	0.0084	0.0014	-0.00179	0.001397
rs969512	A	-0.0106	0.0015	0.009665	0.001469
rs9771228	T	0.0096	0.0015	-0.01105	0.001446
rs977143	A	0.0085	0.0015	0.002145	0.001438
rs978807	A	0.0139	0.0018	-0.00083	0.001757
rs981230	T	0.01	0.0014	-0.00168	0.00139
rs981883	A	0.0099	0.0014	-0.00139	0.001415
rs9820604	T	0.0115	0.0018	-0.00017	0.001835
rs9821664	A	0.0094	0.0017	-0.00172	0.001724
rs9830359	T	-0.0147	0.0022	0.008127	0.002189
rs9844755	A	0.0083	0.0015	-0.004	0.001473
rs9849884	A	-0.009	0.0014	0.002691	0.001407
rs9853928	T	-0.0127	0.0017	0.002272	0.001724
rs9858921	A	-0.0087	0.0014	0.001379	0.001398
rs9859556	T	0.029	0.0015	-0.00663	0.001501
rs9866123	A	0.0086	0.0014	-0.00311	0.0014

rs9870317	A	0.0086	0.0015	-0.00052	0.001492
rs9877225	T	0.009	0.0016	-0.00411	0.00162
rs9882532	T	0.0124	0.0015	-0.00818	0.001451
rs9886703	A	-0.0136	0.0019	-0.00095	0.001862
rs9888796	T	0.0113	0.0016	-0.00046	0.001588
rs9916901	T	0.0092	0.0016	-0.00696	0.001579
rs9924031	C	0.0093	0.0015	-0.00424	0.001429
rs9926649	T	0.0177	0.0031	0.000196	0.003206
rs9927049	A	0.0099	0.0016	0.002227	0.001601
rs9927137	A	0.0082	0.0014	-0.00426	0.001391
rs9927842	T	-0.013	0.002	0.003949	0.001912
rs9929556	T	-0.0099	0.0014	-0.00028	0.001404
rs9929762	A	0.0096	0.0014	-0.00189	0.0014
rs9929993	T	-0.0102	0.0015	0.000551	0.001455
rs9933256	A	0.0119	0.0014	-0.00529	0.001392
rs995698	A	-0.0099	0.0014	0.002889	0.001395
rs9974899	T	-0.0093	0.0016	0.000785	0.001611
rs9977825	T	-0.0092	0.0015	0.006324	0.001449

Supplementary Table 12: Genetic association estimates for the IVW ratio-method MR analysis estimating the effect of education on stroke. ea=effect allele; gx=education; gy=stroke; se=standard error

SNP	ea	gx	gx_se	gy	gy_se
rs10006235	T	-0.0093	0.0016	0.0003	0.0086
rs10009513	A	0.0088	0.0014	-0.0064	0.0080
rs10021733	T	-0.0164	0.0027	-0.0238	0.0131
rs10032941	T	-0.0080	0.0015	0.0135	0.0081
rs10041403	T	0.0151	0.0019	-0.0167	0.0122
rs10042828	A	-0.0145	0.0022	0.0025	0.0146
rs10046069	A	0.0127	0.0022	-0.0157	0.0117
rs10057590	A	0.0106	0.0014	-0.0169	0.0080
rs10060023	T	0.0110	0.0015	-0.0144	0.0085
rs10061420	A	0.0082	0.0014	0.0026	0.0080
rs1006749	A	0.0079	0.0014	0.0137	0.0084
rs10074178	A	0.0079	0.0014	0.0001	0.0078
rs1007731	A	-0.0143	0.0022	-0.0170	0.0156
rs10080647	A	0.0124	0.0020	-0.0049	0.0107
rs1008078	T	-0.0182	0.0014	0.0103	0.0083
rs1009470	T	0.0086	0.0014	0.0120	0.0079
rs10098073	A	-0.0091	0.0014	-0.0059	0.0082
rs10122669	A	-0.0079	0.0014	0.0110	0.0080
rs10128888	A	-0.0083	0.0015	0.0105	0.0087
rs10145520	T	-0.0122	0.0018	0.0095	0.0098
rs10166286	T	0.0151	0.0019	0.0069	0.0098
rs10169002	A	-0.0080	0.0014	0.0027	0.0081
rs10181071	A	0.0102	0.0019	-0.0154	0.0110
rs10189857	A	0.0158	0.0014	-0.0086	0.0084
rs10192369	A	-0.0078	0.0014	0.0048	0.0079
rs10192834	T	0.0083	0.0015	0.0053	0.0084
rs10193498	A	0.0097	0.0016	-0.0114	0.0086
rs10204051	T	-0.0101	0.0014	0.0029	0.0085
rs10205057	T	0.0090	0.0014	-0.0148	0.0078
rs10208	T	0.0091	0.0015	0.0056	0.0087
rs10215082	A	-0.0149	0.0014	0.0074	0.0086
rs1024268	T	-0.0084	0.0014	0.0014	0.0081
rs10251438	A	0.0078	0.0014	-0.0104	0.0079
rs10264573	A	0.0175	0.0032	0.0219	0.0191
rs1035578	A	-0.0094	0.0014	0.0023	0.0080
rs10402747	T	0.0077	0.0014	0.0010	0.0086
rs10411759	A	0.0116	0.0020	-0.0025	0.0103
rs10444280	T	0.0100	0.0018	-0.0097	0.0095
rs10460095	A	-0.0124	0.0014	0.0066	0.0082
rs10480450	A	0.0158	0.0028	-0.0070	0.0137
rs10496091	A	-0.0131	0.0016	-0.0134	0.0091

rs10499535	A	0.0080	0.0014	-0.0096	0.0080
rs1050847	T	0.0092	0.0014	-0.0083	0.0091
rs10509251	T	-0.0093	0.0017	0.0103	0.0092
rs10515007	T	0.0143	0.0019	0.0094	0.0103
rs1051860	A	-0.0079	0.0014	-0.0032	0.0080
rs10519504	T	0.0119	0.0019	0.0096	0.0107
rs1054442	A	-0.0136	0.0015	0.0114	0.0081
rs1061801	A	-0.0110	0.0018	-0.0025	0.0119
rs1066769	A	-0.0230	0.0041	-0.0104	0.0195
rs10742591	A	-0.0084	0.0014	-0.0013	0.0082
rs10748841	A	-0.0111	0.0020	-0.0042	0.0088
rs10750539	A	-0.0095	0.0015	-0.0028	0.0088
rs10752262	T	0.0102	0.0014	-0.0008	0.0082
rs1075228	A	-0.0081	0.0014	0.0134	0.0092
rs10761202	T	0.0088	0.0014	-0.0028	0.0078
rs10761251	A	0.0113	0.0015	0.0065	0.0081
rs10772644	C	0.0142	0.0022	0.0064	0.0129
rs10773002	A	0.0185	0.0016	-0.0150	0.0088
rs10773208	T	-0.0110	0.0016	0.0016	0.0093
rs10782651	T	-0.0082	0.0014	0.0058	0.0078
rs10783243	A	-0.0092	0.0014	-0.0078	0.0078
rs10789285	T	0.0091	0.0016	0.0026	0.0088
rs10793903	A	0.0126	0.0022	-0.0072	0.0120
rs10795831	T	-0.0099	0.0017	-0.0051	0.0090
rs10798888	T	-0.0140	0.0019	-0.0107	0.0101
rs10799615	A	-0.0089	0.0016	-0.0016	0.0086
rs10805383	A	-0.0103	0.0014	0.0172	0.0081
rs10810099	A	-0.0146	0.0016	0.0094	0.0087
rs10810145	A	0.0078	0.0014	-0.0119	0.0085
rs10830858	T	0.0092	0.0014	-0.0008	0.0081
rs10831656	T	0.0090	0.0015	-0.0073	0.0084
rs10844179	A	0.0104	0.0017	-0.0021	0.0092
rs10845051	A	0.0083	0.0014	0.0022	0.0086
rs10875121	C	0.0174	0.0019	-0.0007	0.0111
rs10877283	T	0.0085	0.0014	-0.0001	0.0080
rs10879676	T	-0.0083	0.0014	-0.0070	0.0082
rs10886012	C	0.0078	0.0014	0.0018	0.0079
rs10892807	T	0.0109	0.0014	-0.0068	0.0083
rs10899282	A	0.0140	0.0017	-0.0168	0.0096
rs10927053	A	-0.0125	0.0022	-0.0083	0.0120
rs10928190	T	-0.0087	0.0014	0.0084	0.0081
rs10931821	A	-0.0141	0.0014	0.0063	0.0079
rs10937240	T	-0.0123	0.0018	-0.0047	0.0092
rs10940540	A	0.0078	0.0014	0.0005	0.0082
rs10940921	T	0.0111	0.0014	0.0111	0.0081
rs10943588	A	0.0084	0.0015	-0.0027	0.0082

rs10947439	T	0.0090	0.0015	-0.0059	0.0085
rs10949263	A	-0.0091	0.0016	0.0079	0.0090
rs10951590	T	-0.0109	0.0015	0.0079	0.0085
rs10974256	A	-0.0093	0.0015	0.0001	0.0084
rs1097784	T	-0.0083	0.0014	0.0011	0.0082
rs10979613	T	-0.0117	0.0015	-0.0043	0.0080
rs10983324	A	0.0084	0.0015	-0.0055	0.0084
rs10984445	A	0.0115	0.0014	-0.0075	0.0080
rs10985402	T	0.0089	0.0016	0.0033	0.0092
rs10995639	T	0.0078	0.0014	0.0035	0.0080
rs10996167	C	0.0113	0.0020	-0.0024	0.0090
rs11003463	T	0.0091	0.0015	-0.0181	0.0099
rs11021432	A	0.0103	0.0015	0.0066	0.0086
rs11023764	A	0.0091	0.0015	-0.0072	0.0091
rs11030084	T	0.0106	0.0018	-0.0120	0.0099
rs11030102	C	0.0101	0.0016	-0.0094	0.0102
rs1106090	A	0.0094	0.0014	-0.0035	0.0081
rs11076962	T	0.0086	0.0016	-0.0099	0.0087
rs1107871	A	-0.0083	0.0014	-0.0060	0.0080
rs11081529	T	0.0126	0.0015	0.0003	0.0088
rs11082011	T	0.0194	0.0015	0.0049	0.0085
rs11100237	A	-0.0101	0.0014	0.0038	0.0079
rs11115056	A	0.0187	0.0023	0.0076	0.0152
rs11121177	A	0.0151	0.0018	-0.0293	0.0105
rs111226181	T	-0.0201	0.0018	0.0319	0.0112
rs111235962	T	0.0133	0.0024	0.0015	0.0140
rs11130380	T	0.0090	0.0014	0.0124	0.0086
rs111370527	T	0.0151	0.0027	-0.0061	0.0185
rs11138947	T	0.0105	0.0016	-0.0050	0.0084
rs111530150	T	0.0269	0.0048	-0.0007	0.0318
rs11155813	T	-0.0129	0.0023	0.0076	0.0109
rs11157931	A	-0.0139	0.0014	-0.0005	0.0084
rs11158800	A	-0.0091	0.0014	-0.0005	0.0079
rs1117152	T	0.0106	0.0015	-0.0096	0.0085
rs11174399	A	0.0111	0.0018	-0.0083	0.0099
rs111821073	T	0.0124	0.0019	-0.0041	0.0114
rs111852224	T	0.0156	0.0022	-0.0132	0.0144
rs11190955	T	-0.0083	0.0015	-0.0069	0.0086
rs11191237	T	0.0132	0.0024	0.0073	0.0119
rs1120924	T	0.0108	0.0015	-0.0043	0.0087
rs11209894	A	0.0119	0.0021	0.0406	0.0115
rs11210228	T	-0.0105	0.0019	-0.0016	0.0087
rs11210400	A	0.0120	0.0014	-0.0045	0.0083
rs11211123	A	0.0104	0.0017	-0.0168	0.0091
rs11213482	A	-0.0106	0.0019	-0.0159	0.0132
rs112210983	A	0.0105	0.0017	0.0089	0.0104

rs1123285	C	-0.0094	0.0015	0.0124	0.0085
rs112375785	A	0.0184	0.0021	-0.0046	0.0148
rs112379405	C	-0.0082	0.0014	-0.0151	0.0084
rs112512729	T	-0.0166	0.0027	0.0079	0.0174
rs112603734	A	-0.0131	0.0022	0.0131	0.0106
rs11265191	T	0.0083	0.0015	-0.0014	0.0082
rs112806496	C	-0.0160	0.0025	0.0036	0.0167
rs1128956	T	-0.0140	0.0019	-0.0084	0.0109
rs113205706	A	-0.0135	0.0024	0.0182	0.0156
rs113520408	A	0.0155	0.0016	-0.0111	0.0100
rs113588399	T	-0.0104	0.0017	-0.0075	0.0106
rs113615161	T	-0.0123	0.0021	0.0088	0.0115
rs113720505	A	0.0164	0.0029	0.0140	0.0214
rs114192810	T	-0.0279	0.0051	0.0445	0.0340
rs114468556	A	0.0293	0.0036	-0.0170	0.0263
rs114593137	A	-0.0122	0.0017	0.0083	0.0105
rs1146079	C	-0.0087	0.0016	-0.0032	0.0092
rs114810763	A	-0.0121	0.0020	-0.0156	0.0147
rs114952970	T	0.0265	0.0040	0.0243	0.0274
rs114958262	T	-0.0220	0.0039	-0.0242	0.0285
rs115000530	A	-0.0250	0.0031	-0.0002	0.0205
rs115017135	A	-0.0206	0.0038	0.0255	0.0271
rs11542663	A	0.0112	0.0015	0.0085	0.0084
rs115438240	T	-0.0169	0.0030	0.0250	0.0193
rs115693355	A	0.0309	0.0053	-0.0045	0.0364
rs11588857	A	0.0199	0.0017	0.0141	0.0096
rs11591870	T	0.0098	0.0017	-0.0044	0.0115
rs11592299	A	0.0097	0.0018	-0.0063	0.0115
rs11596387	T	0.0258	0.0046	0.0450	0.0345
rs11598765	A	-0.0106	0.0018	0.0062	0.0099
rs11599236	T	-0.0106	0.0014	-0.0113	0.0081
rs11613431	A	-0.0094	0.0016	-0.0030	0.0099
rs11620355	A	0.0182	0.0025	-0.0127	0.0160
rs11623285	T	-0.0135	0.0021	0.0114	0.0129
rs116386746	C	0.0249	0.0045	-0.0321	0.0316
rs11640569	A	0.0112	0.0020	-0.0053	0.0128
rs11644446	A	0.0117	0.0019	-0.0329	0.0120
rs11647188	A	0.0079	0.0014	-0.0029	0.0083
rs11652522	A	-0.0154	0.0023	0.0092	0.0136
rs11657342	A	0.0140	0.0019	-0.0005	0.0105
rs11657979	A	-0.0091	0.0017	0.0037	0.0096
rs11663602	A	-0.0130	0.0016	0.0105	0.0094
rs11663678	T	-0.0121	0.0022	0.0273	0.0132
rs116656374	T	-0.0242	0.0038	-0.0157	0.0289
rs11675476	T	-0.0127	0.0014	0.0130	0.0079
rs1167827	A	0.0104	0.0014	-0.0328	0.0093

rs11678980	A	-0.0166	0.0014	0.0024	0.0082
rs11687736	A	-0.0105	0.0015	0.0127	0.0086
rs11693885	A	-0.0091	0.0014	0.0046	0.0081
rs116967397	A	0.0315	0.0056	0.0075	0.0397
rs117005905	T	0.0150	0.0022	-0.0219	0.0126
rs11703948	A	-0.0168	0.0024	-0.0041	0.0134
rs1171040	A	-0.0113	0.0017	0.0079	0.0094
rs11716398	A	0.0159	0.0021	-0.0173	0.0103
rs11720093	T	0.0150	0.0026	-0.0132	0.0138
rs11720985	T	0.0085	0.0015	0.0103	0.0094
rs11724690	T	0.0093	0.0015	0.0018	0.0095
rs117273411	T	-0.0211	0.0034	0.0329	0.0249
rs11732160	A	0.0105	0.0016	-0.0054	0.0091
rs11732657	A	-0.0126	0.0016	-0.0081	0.0091
rs11733439	A	-0.0096	0.0017	0.0167	0.0096
rs11736863	A	0.0142	0.0018	-0.0064	0.0107
rs117398064	C	-0.0141	0.0025	-0.0122	0.0174
rs117468730	A	-0.0310	0.0050	-0.0494	0.0338
rs117623407	A	-0.0118	0.0020	0.0011	0.0136
rs11765387	T	0.0086	0.0015	-0.0074	0.0083
rs117668569	T	0.0234	0.0042	-0.0348	0.0277
rs117677995	A	-0.0180	0.0032	0.0191	0.0253
rs11772232	T	0.0177	0.0019	-0.0217	0.0114
rs11774212	T	0.0128	0.0014	0.0007	0.0085
rs117799466	C	0.0106	0.0016	-0.0043	0.0101
rs11780023	T	0.0085	0.0014	0.0144	0.0084
rs11789013	T	0.0106	0.0016	-0.0009	0.0095
rs118040169	A	-0.0248	0.0038	0.0166	0.0258
rs118093058	T	0.0146	0.0021	0.0284	0.0123
rs118134876	T	-0.0223	0.0030	0.0270	0.0207
rs11845781	T	-0.0079	0.0014	0.0099	0.0080
rs11871429	A	0.0128	0.0017	-0.0178	0.0091
rs11876620	T	0.0143	0.0024	-0.0043	0.0149
rs11894424	A	0.0209	0.0033	0.0036	0.0137
rs11917701	A	-0.0087	0.0014	0.0085	0.0081
rs11919835	C	0.0089	0.0015	-0.0079	0.0085
rs1196760	C	0.0139	0.0025	-0.0084	0.0161
rs12005151	A	0.0087	0.0015	-0.0125	0.0085
rs12028010	T	0.0164	0.0017	0.0028	0.0093
rs12028229	T	0.0099	0.0016	-0.0069	0.0117
rs12054166	C	0.0096	0.0016	0.0153	0.0095
rs12076635	C	0.0207	0.0017	-0.0041	0.0091
rs12113634	T	0.0088	0.0015	0.0064	0.0083
rs12123293	T	0.0086	0.0015	-0.0015	0.0083
rs12126231	A	0.0095	0.0014	-0.0183	0.0082
rs12127928	T	0.0108	0.0018	0.0002	0.0109

rs12145078	A	-0.0127	0.0017	0.0168	0.0101
rs12151248	T	-0.0149	0.0023	-0.0235	0.0150
rs12170452	A	0.0105	0.0014	-0.0138	0.0085
rs1220779	A	-0.0079	0.0014	-0.0015	0.0083
rs12234369	A	0.0087	0.0015	-0.0088	0.0089
rs12238011	T	-0.0141	0.0026	0.0104	0.0136
rs12273435	A	-0.0132	0.0017	0.0081	0.0114
rs12285074	A	0.0161	0.0023	0.0194	0.0107
rs12304188	A	0.0111	0.0019	-0.0199	0.0100
rs12325727	A	0.0088	0.0014	-0.0152	0.0081
rs12332731	A	0.0135	0.0018	0.0020	0.0106
rs12342546	A	-0.0128	0.0017	-0.0047	0.0095
rs12359372	T	-0.0092	0.0015	0.0149	0.0086
rs12375949	T	-0.0138	0.0014	0.0023	0.0081
rs12405889	T	-0.0098	0.0014	-0.0075	0.0078
rs12431682	T	-0.0093	0.0015	0.0017	0.0082
rs12438177	A	0.0093	0.0015	0.0028	0.0081
rs12453682	T	0.0102	0.0015	0.0062	0.0083
rs1245829	A	-0.0099	0.0014	0.0032	0.0088
rs12467175	T	-0.0081	0.0014	-0.0070	0.0080
rs12468040	T	0.0137	0.0014	0.0015	0.0082
rs12473986	T	-0.0085	0.0015	0.0057	0.0085
rs12477385	T	0.0109	0.0017	-0.0275	0.0096
rs12478156	T	0.0108	0.0015	-0.0042	0.0083
rs12503522	T	-0.0113	0.0016	0.0100	0.0086
rs12506221	T	-0.0130	0.0014	-0.0026	0.0079
rs12516990	C	-0.0099	0.0017	-0.0012	0.0105
rs12519073	T	-0.0107	0.0017	0.0149	0.0098
rs12524795	T	0.0102	0.0014	-0.0071	0.0081
rs12568153	T	-0.0091	0.0015	0.0030	0.0088
rs12571549	A	0.0151	0.0020	-0.0006	0.0102
rs12574281	A	-0.0080	0.0015	0.0029	0.0090
rs12591647	T	-0.0152	0.0018	0.0149	0.0096
rs12601380	A	-0.0082	0.0014	-0.0102	0.0082
rs12602286	T	0.0160	0.0021	-0.0160	0.0110
rs12613500	C	0.0098	0.0014	-0.0062	0.0081
rs12614263	A	0.0083	0.0014	-0.0026	0.0080
rs12620796	A	0.0121	0.0019	-0.0075	0.0086
rs12638072	A	-0.0107	0.0015	0.0040	0.0092
rs12643771	T	0.0130	0.0015	-0.0131	0.0090
rs12646216	T	0.0084	0.0014	-0.0161	0.0083
rs12646523	T	-0.0132	0.0016	0.0024	0.0090
rs12659776	T	0.0085	0.0014	-0.0079	0.0079
rs12670376	A	0.0092	0.0014	-0.0152	0.0079
rs1267062	C	0.0113	0.0018	-0.0029	0.0111
rs12682775	T	-0.0119	0.0017	0.0230	0.0094

rs12694681	T	0.0102	0.0015	-0.0092	0.0085
rs12709186	A	-0.0091	0.0015	-0.0037	0.0090
rs12712269	T	-0.0107	0.0014	0.0062	0.0081
rs12746551	C	0.0248	0.0041	0.0161	0.0227
rs12750688	C	0.0116	0.0016	-0.0077	0.0100
rs12761729	T	-0.0098	0.0016	0.0168	0.0105
rs12761761	T	0.0153	0.0017	0.0033	0.0103
rs12764593	C	0.0202	0.0029	-0.0426	0.0198
rs12765185	A	-0.0088	0.0016	-0.0102	0.0096
rs12774577	T	0.0142	0.0020	-0.0556	0.0119
rs12789313	T	0.0092	0.0014	-0.0097	0.0082
rs12790196	T	-0.0101	0.0015	0.0129	0.0089
rs12810587	T	0.0094	0.0015	-0.0012	0.0087
rs12875339	A	-0.0108	0.0015	0.0003	0.0086
rs12888615	T	0.0120	0.0018	-0.0170	0.0113
rs12891042	T	0.0092	0.0014	0.0027	0.0080
rs12891191	C	0.0080	0.0014	-0.0059	0.0084
rs12912465	T	-0.0104	0.0016	0.0027	0.0106
rs12952191	T	-0.0081	0.0014	0.0102	0.0081
rs12953422	A	-0.0080	0.0014	0.0102	0.0086
rs12957463	A	0.0146	0.0018	-0.0073	0.0095
rs12962845	C	-0.0123	0.0021	0.0130	0.0115
rs12967010	T	0.0114	0.0017	-0.0041	0.0101
rs12970264	A	-0.0085	0.0014	-0.0254	0.0092
rs12981405	T	-0.0112	0.0019	-0.0055	0.0102
rs13009915	T	0.0212	0.0037	-0.0085	0.0259
rs13010288	T	0.0198	0.0021	-0.0297	0.0129
rs13010566	A	-0.0091	0.0014	-0.0069	0.0083
rs13015496	C	0.0119	0.0018	-0.0007	0.0096
rs13016316	A	-0.0138	0.0024	-0.0099	0.0124
rs1301838	T	-0.0096	0.0015	-0.0003	0.0089
rs13018640	T	-0.0215	0.0014	0.0054	0.0081
rs13026611	A	-0.0084	0.0015	0.0066	0.0085
rs13029602	T	-0.0092	0.0014	-0.0020	0.0083
rs13034349	T	-0.0082	0.0014	0.0177	0.0084
rs13050131	A	-0.0081	0.0015	0.0009	0.0088
rs13085461	C	0.0103	0.0014	-0.0058	0.0079
rs13099165	T	-0.0118	0.0017	0.0142	0.0105
rs13107325	T	-0.0239	0.0027	-0.0005	0.0193
rs13117856	A	0.0084	0.0015	-0.0017	0.0091
rs13130765	C	-0.0091	0.0014	0.0155	0.0088
rs13133213	A	0.0096	0.0014	-0.0201	0.0079
rs13145650	T	-0.0173	0.0025	0.0013	0.0134
rs13154429	C	-0.0136	0.0023	0.0123	0.0142
rs13163062	T	0.0115	0.0014	-0.0079	0.0080
rs13163845	T	-0.0149	0.0020	-0.0104	0.0112

rs13168136	A	-0.0092	0.0017	0.0104	0.0089
rs13169187	A	-0.0079	0.0014	0.0024	0.0080
rs13177031	A	-0.0085	0.0015	0.0012	0.0084
rs13190235	T	-0.0138	0.0024	0.0048	0.0119
rs13197257	T	0.0109	0.0016	-0.0109	0.0093
rs1320139	C	-0.0149	0.0014	0.0082	0.0080
rs13212041	T	0.0101	0.0018	0.0049	0.0094
rs13240401	T	0.0176	0.0017	-0.0142	0.0101
rs13246220	T	-0.0093	0.0014	-0.0079	0.0082
rs13261773	C	0.0122	0.0019	0.0058	0.0108
rs13266287	A	-0.0085	0.0015	0.0159	0.0084
rs13281564	A	0.0088	0.0014	0.0015	0.0083
rs13284516	C	-0.0082	0.0014	-0.0054	0.0087
rs1329044	T	0.0134	0.0019	-0.0026	0.0105
rs1329125	T	-0.0097	0.0015	-0.0042	0.0092
rs13296345	T	0.0090	0.0015	-0.0202	0.0081
rs13327482	A	-0.0121	0.0018	0.0148	0.0115
rs1334297	A	0.0257	0.0016	-0.0066	0.0092
rs1335482	T	0.0096	0.0014	-0.0221	0.0089
rs13361043	T	-0.0158	0.0028	0.0062	0.0127
rs13381557	A	-0.0087	0.0014	-0.0010	0.0080
rs13388333	A	0.0085	0.0015	0.0040	0.0087
rs13397529	C	-0.0097	0.0017	0.0085	0.0102
rs13398860	A	0.0086	0.0015	0.0028	0.0085
rs13402497	A	-0.0080	0.0014	0.0075	0.0077
rs13422673	T	-0.0110	0.0014	-0.0042	0.0083
rs13425585	C	-0.0089	0.0014	0.0134	0.0080
rs1368250	T	0.0250	0.0045	0.0055	0.0367
rs137079	T	0.0128	0.0020	0.0039	0.0119
rs137858393	A	0.0193	0.0029	-0.0163	0.0194
rs138096147	A	0.0234	0.0042	0.0273	0.0320
rs1391513	T	-0.0095	0.0015	0.0127	0.0087
rs139244147	A	-0.0193	0.0030	0.0006	0.0166
rs1392816	T	0.0082	0.0014	-0.0117	0.0085
rs139980871	T	0.0180	0.0029	-0.0069	0.0192
rs1404549	A	-0.0089	0.0015	0.0047	0.0089
rs1405876	T	0.0109	0.0015	0.0059	0.0087
rs140711597	C	0.0366	0.0055	-0.0578	0.0418
rs1408284	C	-0.0127	0.0020	0.0334	0.0131
rs1408430	C	0.0096	0.0016	0.0089	0.0088
rs141586924	A	-0.0090	0.0015	-0.0016	0.0098
rs141729694	T	0.0195	0.0026	0.0160	0.0151
rs142014757	A	0.0102	0.0018	-0.0143	0.0131
rs1426619	T	0.0096	0.0014	0.0041	0.0079
rs1427298	T	0.0086	0.0014	-0.0096	0.0083
rs142747148	A	0.0329	0.0052	-0.0206	0.0371

rs1427829	A	-0.0083	0.0014	0.0170	0.0080
rs143148393	T	-0.0211	0.0039	-0.0213	0.0271
rs1434630	T	-0.0137	0.0020	-0.0147	0.0108
rs143743568	A	0.0118	0.0020	-0.0001	0.0116
rs143812851	A	-0.0113	0.0019	0.0061	0.0118
rs1440930	C	0.0081	0.0014	-0.0024	0.0084
rs144336753	A	0.0388	0.0053	0.0087	0.0357
rs1445591	A	0.0085	0.0015	-0.0039	0.0098
rs145018899	A	0.0095	0.0015	-0.0006	0.0092
rs1464297	T	-0.0109	0.0015	0.0013	0.0084
rs1467737	T	-0.0084	0.0014	0.0022	0.0082
rs1485300	C	0.0098	0.0015	-0.0103	0.0086
rs1490612	T	0.0193	0.0026	-0.0228	0.0169
rs150252215	A	0.0204	0.0035	-0.0201	0.0224
rs150537577	A	-0.0151	0.0026	-0.0209	0.0140
rs1505676	C	0.0084	0.0015	-0.0041	0.0082
rs151381	T	-0.0087	0.0014	0.0057	0.0080
rs1518890	T	-0.0090	0.0016	-0.0080	0.0090
rs1527878	A	-0.0105	0.0016	0.0020	0.0098
rs1529597	A	0.0223	0.0038	-0.0330	0.0236
rs1538389	T	-0.0109	0.0018	0.0053	0.0103
rs1542354	A	-0.0084	0.0014	0.0018	0.0082
rs1544	A	-0.0096	0.0016	0.0090	0.0090
rs1554798	A	-0.0080	0.0014	0.0134	0.0081
rs1564347	T	0.0088	0.0015	0.0024	0.0084
rs1569092	A	0.0160	0.0020	-0.0013	0.0111
rs1593022	T	-0.0099	0.0017	0.0041	0.0099
rs1603460	T	0.0090	0.0014	-0.0081	0.0081
rs162445	A	0.0152	0.0026	0.0236	0.0121
rs163229	C	-0.0260	0.0047	0.0090	0.0311
rs1637770	T	0.0191	0.0028	0.0306	0.0174
rs164938	T	-0.0088	0.0014	0.0107	0.0081
rs165633	A	-0.0116	0.0016	0.0074	0.0101
rs1656614	C	0.0122	0.0015	-0.0105	0.0094
rs1671269	T	-0.0100	0.0016	-0.0110	0.0088
rs1671770	A	0.0125	0.0018	-0.0051	0.0104
rs16851779	T	-0.0153	0.0027	0.0001	0.0146
rs16871807	T	0.0090	0.0015	0.0052	0.0083
rs1689510	C	0.0194	0.0015	-0.0187	0.0088
rs16901689	T	0.0111	0.0019	-0.0045	0.0107
rs1693584	T	-0.0087	0.0015	0.0180	0.0088
rs16958559	T	0.0096	0.0017	0.0092	0.0106
rs16966271	T	0.0092	0.0016	0.0034	0.0087
rs16975275	A	-0.0102	0.0017	0.0049	0.0095
rs17048801	A	-0.0106	0.0014	0.0099	0.0085
rs17048855	A	0.0108	0.0015	-0.0128	0.0083

rs17069646	T	0.0085	0.0015	0.0032	0.0082
rs17110109	T	-0.0093	0.0014	0.0262	0.0081
rs17113730	A	-0.0170	0.0027	0.0041	0.0165
rs17131123	A	-0.0243	0.0039	0.0484	0.0289
rs17133297	A	-0.0146	0.0023	0.0075	0.0117
rs17144467	A	0.0098	0.0015	-0.0060	0.0084
rs17148998	A	0.0101	0.0017	-0.0060	0.0108
rs171697	C	0.0133	0.0015	-0.0200	0.0093
rs17170519	C	0.0132	0.0023	-0.0074	0.0114
rs1717204	A	-0.0117	0.0018	0.0093	0.0112
rs1718188	A	0.0096	0.0014	0.0011	0.0079
rs17186106	T	0.0108	0.0018	-0.0281	0.0109
rs17190418	T	-0.0213	0.0031	0.0199	0.0210
rs17205908	T	-0.0092	0.0015	-0.0072	0.0097
rs17248751	A	-0.0136	0.0017	0.0152	0.0111
rs1728118	A	0.0089	0.0016	-0.0013	0.0085
rs1729412	T	-0.0107	0.0014	-0.0010	0.0080
rs173003	A	-0.0082	0.0014	-0.0022	0.0083
rs1734370	A	0.0099	0.0016	-0.0036	0.0088
rs1738050	C	-0.0093	0.0014	0.0066	0.0084
rs17411339	A	0.0140	0.0014	-0.0101	0.0088
rs17428076	C	0.0137	0.0016	-0.0089	0.0099
rs17440885	A	0.0160	0.0029	0.0177	0.0187
rs1747714	T	0.0106	0.0014	-0.0143	0.0084
rs1747817	T	-0.0092	0.0016	0.0171	0.0100
rs17489649	A	0.0127	0.0015	-0.0146	0.0093
rs17502934	T	-0.0167	0.0020	0.0061	0.0120
rs175325	A	-0.0100	0.0014	0.0071	0.0084
rs17536059	C	-0.0108	0.0019	0.0029	0.0114
rs17551064	A	0.0141	0.0019	0.0025	0.0111
rs17563464	A	-0.0147	0.0017	0.0295	0.0119
rs17565975	A	-0.0106	0.0014	-0.0042	0.0080
rs17568389	A	0.0127	0.0014	-0.0004	0.0081
rs17574007	A	-0.0129	0.0021	0.0114	0.0137
rs1758747	A	0.0092	0.0015	-0.0072	0.0085
rs17598675	T	-0.0116	0.0014	0.0048	0.0085
rs17604349	A	0.0127	0.0018	-0.0036	0.0111
rs17609255	T	-0.0087	0.0014	0.0125	0.0081
rs176218	T	0.0200	0.0018	-0.0010	0.0094
rs17622379	T	-0.0113	0.0018	0.0094	0.0105
rs17650634	T	-0.0104	0.0019	0.0088	0.0117
rs17667540	A	-0.0087	0.0015	-0.0025	0.0081
rs17669337	T	-0.0110	0.0014	-0.0085	0.0080
rs17680712	T	-0.0092	0.0014	0.0050	0.0078
rs17686649	T	-0.0098	0.0017	0.0164	0.0102
rs17732878	T	-0.0095	0.0017	-0.0069	0.0103

rs17742342	A	-0.0119	0.0018	0.0006	0.0105
rs17747544	A	-0.0090	0.0014	-0.0008	0.0082
rs1779549	A	0.0081	0.0014	-0.0148	0.0080
rs178183	T	0.0145	0.0016	-0.0018	0.0091
rs17882802	A	0.0084	0.0014	-0.0153	0.0086
rs17883331	A	-0.0108	0.0018	0.0074	0.0112
rs1792602	A	-0.0097	0.0014	0.0082	0.0082
rs181214	T	-0.0104	0.0017	0.0087	0.0113
rs182902112	A	-0.0300	0.0053	-0.0179	0.0357
rs1835339	T	0.0084	0.0014	0.0105	0.0082
rs183869217	C	0.0174	0.0030	0.0077	0.0189
rs1841023	A	-0.0092	0.0015	-0.0013	0.0092
rs1842713	A	-0.0117	0.0017	-0.0313	0.0093
rs1843815	A	0.0077	0.0014	-0.0146	0.0080
rs1861786	A	-0.0081	0.0014	0.0046	0.0081
rs1865955	T	0.0122	0.0019	-0.0076	0.0096
rs1866823	A	0.0107	0.0014	0.0009	0.0079
rs187580	T	-0.0104	0.0017	-0.0028	0.0106
rs187951956	A	-0.0315	0.0058	0.0283	0.0418
rs1880088	A	-0.0097	0.0016	0.0184	0.0089
rs1880692	A	0.0086	0.0014	0.0039	0.0081
rs1890132	T	-0.0089	0.0015	0.0114	0.0098
rs1898111	A	0.0101	0.0018	-0.0051	0.0098
rs190102446	T	-0.0210	0.0037	0.0167	0.0241
rs1905616	A	0.0083	0.0015	-0.0035	0.0086
rs1910005	T	-0.0090	0.0015	-0.0046	0.0090
rs1918394	T	0.0119	0.0019	-0.0080	0.0103
rs192436652	T	-0.0319	0.0045	0.0266	0.0318
rs1933264	T	0.0100	0.0016	-0.0090	0.0090
rs1952183	A	-0.0089	0.0014	-0.0017	0.0082
rs1955250	A	-0.0140	0.0025	0.0105	0.0133
rs1963381	A	0.0099	0.0016	0.0147	0.0090
rs1980129	A	0.0085	0.0014	0.0016	0.0079
rs198262	T	-0.0223	0.0035	0.0039	0.0227
rs1991585	T	-0.0101	0.0015	0.0065	0.0087
rs1995181	A	-0.0078	0.0014	-0.0036	0.0077
rs2007655	T	0.0086	0.0014	-0.0031	0.0078
rs2011603	A	-0.0091	0.0016	0.0262	0.0089
rs2014830	T	0.0110	0.0015	-0.0106	0.0088
rs201495	T	-0.0112	0.0019	0.0003	0.0106
rs2023016	C	0.0107	0.0017	-0.0169	0.0093
rs2024568	T	-0.0097	0.0016	0.0280	0.0089
rs2029401	A	-0.0091	0.0014	0.0089	0.0082
rs2034631	T	0.0089	0.0015	-0.0152	0.0082
rs2039204	A	0.0082	0.0014	-0.0088	0.0079
rs2043187	A	0.0107	0.0015	-0.0108	0.0090

rs2052285	A	0.0112	0.0014	0.0017	0.0084
rs2055940	A	0.0082	0.0015	-0.0161	0.0089
rs2077235	T	0.0097	0.0017	0.0013	0.0090
rs2081652	A	0.0123	0.0015	-0.0041	0.0087
rs2082317	T	-0.0078	0.0014	0.0087	0.0081
rs2088913	A	0.0081	0.0014	-0.0044	0.0079
rs2092248	T	-0.0083	0.0015	0.0010	0.0087
rs2098526	A	-0.0241	0.0043	0.0110	0.0203
rs2100249	T	-0.0089	0.0015	0.0034	0.0082
rs2126069	T	0.0080	0.0014	0.0061	0.0082
rs2131167	A	0.0080	0.0014	-0.0025	0.0081
rs214626	A	0.0101	0.0018	0.0028	0.0096
rs2160514	A	-0.0094	0.0014	0.0027	0.0081
rs2179152	T	-0.0131	0.0015	0.0039	0.0087
rs2183271	T	0.0084	0.0015	0.0144	0.0088
rs2199409	T	0.0103	0.0017	0.0129	0.0100
rs2212430	T	-0.0100	0.0016	0.0003	0.0087
rs2216144	T	0.0098	0.0014	-0.0103	0.0078
rs2220926	T	-0.0095	0.0014	0.0062	0.0078
rs2250660	C	0.0080	0.0014	-0.0123	0.0081
rs2252098	T	-0.0077	0.0014	0.0003	0.0086
rs2254681	A	0.0101	0.0016	0.0054	0.0094
rs2256965	A	0.0106	0.0014	-0.0060	0.0084
rs2276209	A	0.0085	0.0015	0.0159	0.0083
rs2283076	A	0.0115	0.0017	0.0079	0.0103
rs2287838	A	-0.0104	0.0014	-0.0024	0.0081
rs2290601	T	-0.0149	0.0017	0.0103	0.0088
rs2297293	C	0.0099	0.0015	-0.0006	0.0088
rs2299156	T	0.0099	0.0018	0.0041	0.0098
rs2314338	T	0.0093	0.0016	-0.0063	0.0093
rs2321157	A	-0.0081	0.0014	0.0087	0.0079
rs232496	T	0.0090	0.0015	-0.0015	0.0082
rs2332179	A	0.0121	0.0020	0.0105	0.0117
rs2336721	T	0.0095	0.0015	-0.0040	0.0088
rs2358628	A	-0.0091	0.0015	0.0135	0.0085
rs236318	C	-0.0102	0.0017	-0.0082	0.0092
rs2364544	A	-0.0105	0.0014	-0.0040	0.0083
rs2365376	A	0.0092	0.0015	0.0065	0.0084
rs2368831	T	-0.0092	0.0014	-0.0026	0.0096
rs2373124	A	-0.0096	0.0016	0.0138	0.0096
rs2406253	A	0.0135	0.0018	-0.0115	0.0108
rs2414072	A	-0.0083	0.0014	0.0164	0.0078
rs2416214	A	0.0079	0.0014	0.0030	0.0082
rs2416759	A	-0.0093	0.0015	-0.0134	0.0085
rs2416845	T	0.0263	0.0048	-0.0177	0.0247
rs2431023	A	0.0093	0.0014	-0.0121	0.0080

rs2434672	A	0.0091	0.0014	0.0040	0.0082
rs2436760	T	0.0232	0.0042	-0.0124	0.0186
rs2447097	T	0.0096	0.0014	-0.0011	0.0080
rs2458370	T	-0.0086	0.0015	0.0136	0.0080
rs2469226	A	0.0096	0.0017	-0.0061	0.0100
rs2470966	C	-0.0103	0.0015	0.0157	0.0098
rs2478208	C	-0.0103	0.0014	-0.0018	0.0079
rs2496482	T	0.0121	0.0015	-0.0137	0.0092
rs2517086	C	-0.0088	0.0014	0.0011	0.0085
rs2521602	A	-0.0266	0.0049	-0.0114	0.0218
rs2522545	T	-0.0083	0.0014	-0.0002	0.0081
rs2526398	C	-0.0222	0.0015	0.0172	0.0090
rs2529069	T	-0.0106	0.0016	0.0111	0.0087
rs252991	A	0.0095	0.0015	-0.0068	0.0081
rs2542673	A	0.0088	0.0015	0.0083	0.0083
rs2545795	A	0.0125	0.0014	0.0004	0.0082
rs255053	A	-0.0111	0.0018	-0.0069	0.0098
rs2554835	A	0.0088	0.0014	-0.0080	0.0085
rs2568955	T	-0.0165	0.0016	0.0067	0.0098
rs2569041	T	0.0077	0.0014	-0.0009	0.0085
rs2570497	T	-0.0121	0.0015	0.0102	0.0081
rs2588959	T	-0.0080	0.0014	0.0066	0.0078
rs2657283	T	0.0085	0.0014	-0.0067	0.0085
rs2665668	A	-0.0094	0.0015	-0.0046	0.0087
rs2702576	A	-0.0090	0.0014	0.0172	0.0086
rs2718277	T	0.0142	0.0026	-0.0030	0.0147
rs2718791	T	-0.0081	0.0015	-0.0097	0.0087
rs27220	A	-0.0114	0.0015	-0.0080	0.0082
rs2725370	T	-0.0141	0.0015	-0.0069	0.0092
rs2736752	T	0.0095	0.0017	-0.0125	0.0110
rs2740795	A	0.0098	0.0016	0.0183	0.0089
rs2761438	A	0.0098	0.0015	-0.0082	0.0083
rs2764684	T	0.0143	0.0019	0.0105	0.0118
rs2805064	C	0.0093	0.0016	-0.0084	0.0095
rs2838006	T	0.0132	0.0015	-0.0087	0.0084
rs28482086	A	0.0086	0.0015	-0.0004	0.0086
rs28505285	C	0.0106	0.0018	0.0071	0.0115
rs28512462	C	0.0112	0.0014	0.0023	0.0082
rs28513882	A	-0.0110	0.0018	-0.0004	0.0116
rs2852349	T	-0.0093	0.0014	-0.0009	0.0081
rs28587776	T	0.0088	0.0015	-0.0097	0.0086
rs28669886	A	-0.0084	0.0015	0.0132	0.0086
rs2870281	A	-0.0083	0.0014	0.0064	0.0080
rs28735993	A	0.0162	0.0022	0.0107	0.0127
rs2885198	A	0.0088	0.0014	-0.0138	0.0082
rs2898191	A	0.0093	0.0015	-0.0002	0.0088

rs2910823	T	0.0115	0.0014	0.0121	0.0085
rs2916490	A	-0.0092	0.0015	0.0087	0.0090
rs2923424	A	0.0120	0.0014	-0.0013	0.0080
rs2926702	T	-0.0124	0.0021	0.0148	0.0108
rs2929032	A	-0.0081	0.0014	-0.0042	0.0078
rs2929860	T	0.0110	0.0018	0.0001	0.0115
rs2942884	A	-0.0090	0.0014	-0.0020	0.0079
rs2954114	A	-0.0081	0.0014	0.0004	0.0081
rs2958182	A	0.0082	0.0015	0.0016	0.0087
rs2962378	A	0.0101	0.0017	-0.0014	0.0092
rs2964199	T	-0.0099	0.0015	0.0061	0.0085
rs2964255	A	0.0088	0.0015	-0.0161	0.0085
rs2976397	T	0.0081	0.0014	0.0109	0.0084
rs2977464	T	0.0102	0.0018	0.0016	0.0095
rs29792	A	-0.0093	0.0015	-0.0257	0.0090
rs2980813	A	0.0088	0.0014	-0.0104	0.0081
rs2989476	C	0.0078	0.0014	-0.0188	0.0080
rs2989751	A	-0.0092	0.0017	0.0014	0.0089
rs2992037	A	0.0149	0.0015	-0.0038	0.0091
rs2994326	T	-0.0100	0.0018	0.0013	0.0108
rs2998299	T	-0.0136	0.0017	-0.0022	0.0096
rs3026996	A	0.0138	0.0016	0.0031	0.0106
rs303752	A	-0.0110	0.0014	0.0291	0.0089
rs3111251	T	-0.0090	0.0014	-0.0021	0.0080
rs312927	A	-0.0147	0.0022	0.0194	0.0136
rs312945	A	-0.0091	0.0015	-0.0081	0.0087
rs317050	T	0.0078	0.0014	-0.0164	0.0079
rs322614	A	-0.0087	0.0014	0.0066	0.0080
rs322744	T	-0.0090	0.0016	-0.0001	0.0087
rs324885	A	0.0142	0.0014	-0.0079	0.0081
rs32940	T	-0.0096	0.0015	-0.0022	0.0088
rs339057	A	-0.0081	0.0014	0.0111	0.0081
rs34067381	T	-0.0104	0.0015	0.0310	0.0090
rs34098770	A	-0.0160	0.0020	0.0024	0.0106
rs34106693	C	0.0159	0.0019	-0.0124	0.0122
rs34122915	C	0.0084	0.0014	-0.0034	0.0093
rs341504	A	0.0099	0.0015	-0.0116	0.0082
rs34155847	A	0.0135	0.0016	-0.0091	0.0094
rs34262657	T	-0.0240	0.0038	-0.0276	0.0255
rs34286836	T	-0.0089	0.0014	-0.0136	0.0080
rs34298584	A	0.0111	0.0019	-0.0003	0.0115
rs34305371	A	0.0314	0.0024	-0.0241	0.0164
rs34309	A	0.0080	0.0014	-0.0022	0.0083
rs34316274	A	0.0097	0.0017	-0.0052	0.0091
rs34363861	A	0.0087	0.0014	-0.0031	0.0083
rs34394051	A	-0.0117	0.0020	0.0113	0.0103

rs34410	C	-0.0083	0.0014	0.0077	0.0080
rs34624793	T	0.0116	0.0021	-0.0145	0.0144
rs34720381	T	-0.0180	0.0024	0.0377	0.0141
rs34748029	A	0.0185	0.0025	0.0138	0.0176
rs347661	T	-0.0081	0.0014	0.0017	0.0084
rs34780702	A	0.0111	0.0017	-0.0002	0.0092
rs34807077	A	0.0128	0.0019	0.0018	0.0099
rs34811474	A	0.0104	0.0017	-0.0161	0.0128
rs34967082	A	-0.0080	0.0014	-0.0064	0.0084
rs35016816	T	0.0176	0.0023	0.0041	0.0128
rs350281	T	-0.0117	0.0015	-0.0102	0.0112
rs35104491	A	0.0124	0.0018	0.0105	0.0096
rs35111506	C	-0.0175	0.0030	-0.0021	0.0192
rs35192107	T	-0.0091	0.0017	0.0225	0.0098
rs35319653	T	0.0119	0.0015	0.0028	0.0086
rs35417702	T	-0.0152	0.0014	0.0030	0.0078
rs35493937	C	0.0124	0.0017	-0.0016	0.0094
rs35606437	A	0.0105	0.0016	-0.0011	0.0090
rs356999	A	-0.0101	0.0014	-0.0049	0.0085
rs35745455	A	0.0080	0.0014	-0.0031	0.0084
rs35751693	T	-0.0246	0.0040	-0.0001	0.0308
rs35754740	T	0.0083	0.0014	0.0053	0.0080
rs35811586	T	0.0217	0.0031	-0.0050	0.0225
rs35929923	A	-0.0114	0.0016	0.0110	0.0097
rs36085856	C	0.0201	0.0036	-0.0112	0.0183
rs36120534	T	0.0100	0.0018	0.0000	0.0114
rs362307	T	-0.0226	0.0027	0.0607	0.0176
rs363096	T	-0.0143	0.0014	0.0140	0.0079
rs3735478	T	0.0108	0.0016	0.0053	0.0096
rs3751331	A	-0.0097	0.0014	-0.0109	0.0084
rs3766979	A	0.0085	0.0015	-0.0051	0.0084
rs3768480	C	0.0101	0.0014	-0.0093	0.0085
rs3781339	T	-0.0104	0.0018	0.0254	0.0099
rs3790609	T	0.0107	0.0019	-0.0051	0.0105
rs3809169	T	0.0140	0.0025	-0.0153	0.0145
rs3809634	A	-0.0109	0.0015	0.0030	0.0091
rs3812281	T	0.0113	0.0014	-0.0035	0.0081
rs3817923	A	-0.0121	0.0022	0.0149	0.0131
rs382196	T	-0.0082	0.0014	-0.0053	0.0083
rs3847228	T	0.0083	0.0014	0.0089	0.0086
rs3848715	A	-0.0080	0.0014	0.0054	0.0084
rs3859523	T	0.0124	0.0018	0.0031	0.0108
rs387027	A	-0.0083	0.0015	0.0114	0.0085
rs3895736	A	0.0144	0.0019	-0.0115	0.0115
rs3897821	A	0.0154	0.0015	0.0000	0.0085
rs3948495	T	-0.0089	0.0014	0.0062	0.0083

rs399821	A	-0.0078	0.0014	-0.0046	0.0078
rs39998	A	0.0090	0.0015	-0.0033	0.0094
rs401526	T	-0.0104	0.0014	0.0060	0.0081
rs401966	C	-0.0097	0.0014	0.0076	0.0088
rs4127499	A	0.0087	0.0015	-0.0037	0.0091
rs41282553	A	0.0237	0.0040	-0.0155	0.0256
rs42210	C	-0.0098	0.0016	-0.0039	0.0088
rs42302	A	0.0086	0.0015	-0.0085	0.0084
rs4255791	A	-0.0084	0.0015	-0.0014	0.0092
rs4263475	A	0.0078	0.0014	-0.0089	0.0086
rs4283754	A	-0.0084	0.0014	-0.0006	0.0098
rs429150	T	0.0085	0.0014	-0.0005	0.0085
rs4298514	T	0.0104	0.0015	-0.0108	0.0084
rs4320563	A	0.0109	0.0014	-0.0158	0.0078
rs4328757	T	0.0096	0.0014	0.0037	0.0082
rs4352658	T	-0.0190	0.0026	-0.0091	0.0141
rs4358081	A	-0.0095	0.0014	-0.0047	0.0082
rs4358358	A	0.0109	0.0015	-0.0048	0.0090
rs4369924	A	0.0125	0.0019	-0.0239	0.0102
rs4384309	A	0.0102	0.0014	-0.0084	0.0083
rs4396896	A	-0.0107	0.0014	0.0019	0.0079
rs4423373	A	-0.0087	0.0014	0.0037	0.0086
rs4426420	T	-0.0140	0.0019	0.0005	0.0111
rs4434676	A	-0.0089	0.0014	0.0246	0.0085
rs4458044	C	0.0101	0.0016	-0.0178	0.0110
rs4467547	T	0.0121	0.0014	-0.0187	0.0091
rs4469771	T	0.0101	0.0015	-0.0082	0.0083
rs4480339	A	-0.0097	0.0015	0.0017	0.0094
rs4490539	A	0.0142	0.0015	-0.0021	0.0092
rs4497562	A	0.0112	0.0016	-0.0125	0.0088
rs4500930	T	-0.0109	0.0015	0.0114	0.0084
rs4500960	T	-0.0131	0.0014	-0.0180	0.0079
rs4502401	T	0.0085	0.0015	-0.0027	0.0086
rs4652135	A	0.0103	0.0016	-0.0046	0.0087
rs4658019	T	0.0084	0.0014	0.0021	0.0081
rs4663617	A	0.0104	0.0017	0.0024	0.0098
rs4664983	T	-0.0103	0.0018	0.0037	0.0093
rs4673840	T	-0.0138	0.0019	0.0008	0.0105
rs4675248	A	-0.0094	0.0014	-0.0031	0.0082
rs4685405	T	-0.0111	0.0018	0.0010	0.0098
rs4687735	T	-0.0200	0.0033	0.0532	0.0226
rs4691601	A	-0.0115	0.0014	0.0000	0.0080
rs4705763	C	0.0089	0.0014	0.0011	0.0079
rs4712371	A	-0.0124	0.0021	0.0158	0.0116
rs4719460	T	-0.0088	0.0015	0.0167	0.0083
rs4719944	T	-0.0121	0.0014	-0.0011	0.0079

rs4724083	T	0.0083	0.0015	0.0015	0.0087
rs4726070	A	0.0122	0.0014	0.0011	0.0081
rs4730020	T	0.0090	0.0016	-0.0127	0.0101
rs4731413	A	0.0111	0.0017	-0.0090	0.0111
rs4735297	A	-0.0086	0.0015	-0.0011	0.0082
rs4739235	A	0.0109	0.0019	-0.0206	0.0101
rs4741571	A	-0.0102	0.0015	0.0016	0.0093
rs4757957	C	0.0133	0.0015	0.0013	0.0087
rs4766424	C	0.0142	0.0021	-0.0109	0.0135
rs4778058	T	-0.0092	0.0014	0.0051	0.0082
rs4785187	A	-0.0100	0.0017	0.0033	0.0092
rs4787028	T	0.0086	0.0015	-0.0031	0.0083
rs4787457	A	0.0162	0.0015	0.0053	0.0085
rs4788115	A	0.0111	0.0019	-0.0144	0.0114
rs479018	A	0.0109	0.0015	0.0024	0.0099
rs4793090	A	0.0084	0.0015	0.0039	0.0081
rs4810894	A	-0.0085	0.0015	-0.0065	0.0082
rs4812697	T	0.0171	0.0027	-0.0353	0.0179
rs481940	T	0.0100	0.0016	0.0112	0.0094
rs482787	T	0.0100	0.0015	0.0108	0.0083
rs4839155	T	0.0115	0.0017	0.0133	0.0096
rs4846010	A	0.0099	0.0018	-0.0147	0.0121
rs4846724	A	0.0098	0.0014	-0.0013	0.0079
rs4848924	A	-0.0117	0.0015	0.0084	0.0085
rs4850954	T	0.0077	0.0014	-0.0005	0.0082
rs4851263	A	-0.0151	0.0023	0.0077	0.0119
rs4877516	A	-0.0101	0.0014	0.0102	0.0079
rs4881269	A	0.0100	0.0014	-0.0063	0.0081
rs488476	C	-0.0113	0.0015	0.0065	0.0085
rs4894658	C	0.0091	0.0016	0.0032	0.0088
rs4895650	T	0.0093	0.0014	0.0083	0.0088
rs4899012	C	-0.0103	0.0014	0.0087	0.0084
rs4904523	A	-0.0082	0.0014	0.0082	0.0079
rs4915735	A	0.0116	0.0020	-0.0047	0.0120
rs4919624	A	0.0193	0.0018	-0.0043	0.0100
rs4925065	T	-0.0078	0.0014	0.0014	0.0081
rs4925109	A	-0.0096	0.0015	0.0213	0.0086
rs4938815	T	0.0085	0.0015	0.0061	0.0088
rs4941735	T	0.0092	0.0014	0.0052	0.0083
rs4972748	T	0.0105	0.0018	-0.0115	0.0096
rs4977885	A	-0.0084	0.0014	0.0134	0.0085
rs4981245	A	0.0080	0.0014	-0.0045	0.0085
rs4984541	A	-0.0138	0.0017	-0.0005	0.0093
rs4984613	T	0.0174	0.0028	-0.0208	0.0147
rs4984682	C	-0.0138	0.0017	-0.0034	0.0091
rs548897	A	0.0081	0.0014	-0.0100	0.0083

rs55675587	T	-0.0103	0.0018	0.0071	0.0098
rs55736314	C	-0.0154	0.0014	0.0183	0.0090
rs55826493	T	-0.0127	0.0021	-0.0179	0.0135
rs55986781	T	0.0187	0.0026	-0.0291	0.0166
rs56085180	A	-0.0269	0.0037	-0.0020	0.0236
rs56099375	T	0.0120	0.0017	0.0042	0.0108
rs56171318	T	-0.0137	0.0020	-0.0105	0.0121
rs56174996	A	-0.0143	0.0020	0.0001	0.0131
rs56306882	A	0.0086	0.0016	0.0065	0.0097
rs56319902	T	-0.0206	0.0017	0.0211	0.0125
rs56391344	A	0.0148	0.0016	-0.0135	0.0103
rs563954	A	-0.0081	0.0014	0.0097	0.0086
rs56405138	A	0.0153	0.0022	-0.0070	0.0127
rs56408528	T	-0.0082	0.0014	0.0095	0.0080
rs567003	A	0.0094	0.0015	-0.0048	0.0090
rs56794817	A	0.0122	0.0019	-0.0089	0.0101
rs57016874	T	0.0209	0.0037	-0.0085	0.0211
rs57148205	A	0.0095	0.0016	-0.0160	0.0097
rs57204268	A	0.0114	0.0020	-0.0010	0.0125
rs57349798	A	0.0093	0.0014	-0.0171	0.0087
rs57352738	A	-0.0152	0.0017	-0.0072	0.0103
rs57437407	A	-0.0145	0.0023	-0.0229	0.0137
rs575113	A	0.0133	0.0015	-0.0151	0.0087
rs5754581	T	-0.0082	0.0014	-0.0041	0.0080
rs5754753	T	-0.0126	0.0016	-0.0033	0.0099
rs5754762	A	0.0197	0.0030	-0.0291	0.0139
rs57661533	T	-0.0114	0.0021	-0.0002	0.0103
rs580652	T	-0.0147	0.0024	0.0080	0.0111
rs585557	A	0.0128	0.0018	-0.0294	0.0095
rs58779949	A	0.0110	0.0019	-0.0208	0.0112
rs58859557	T	0.0187	0.0028	-0.0561	0.0150
rs58921703	T	0.0109	0.0015	-0.0155	0.0083
rs58950082	T	0.0095	0.0017	-0.0093	0.0094
rs58996896	A	-0.0094	0.0016	0.0059	0.0094
rs590013	T	0.0100	0.0015	-0.0085	0.0086
rs59300999	T	-0.0164	0.0028	0.0065	0.0121
rs59480703	C	-0.0123	0.0018	-0.0160	0.0096
rs59813324	T	0.0097	0.0018	-0.0060	0.0095
rs59967356	T	-0.0127	0.0021	-0.0050	0.0144
rs60096640	A	0.0159	0.0023	-0.0006	0.0108
rs6020560	T	-0.0083	0.0014	-0.0078	0.0080
rs6043521	T	-0.0083	0.0014	-0.0057	0.0084
rs60589532	A	0.0168	0.0028	0.0086	0.0152
rs6060308	A	0.0095	0.0016	-0.0208	0.0092
rs6065080	T	-0.0130	0.0015	0.0001	0.0082
rs6065784	C	0.0110	0.0015	-0.0042	0.0092

rs60717745	C	-0.0118	0.0019	-0.0092	0.0105
rs60726488	T	-0.0090	0.0016	0.0021	0.0093
rs6091570	A	0.0081	0.0015	-0.0106	0.0082
rs61104616	A	-0.0172	0.0014	0.0047	0.0080
rs6123924	A	0.0129	0.0019	-0.0163	0.0102
rs613872	T	-0.0163	0.0019	0.0168	0.0121
rs61387839	A	0.0088	0.0015	-0.0028	0.0095
rs61527214	A	0.0110	0.0014	0.0000	0.0085
rs61739710	A	-0.0099	0.0016	0.0027	0.0091
rs61748951	A	-0.0273	0.0048	0.0547	0.0362
rs61755388	T	0.0125	0.0017	0.0202	0.0107
rs61798586	A	-0.0112	0.0020	-0.0017	0.0115
rs61853335	T	-0.0109	0.0018	0.0190	0.0100
rs61958175	A	-0.0198	0.0034	0.0378	0.0251
rs61997667	T	-0.0144	0.0020	0.0071	0.0118
rs62007304	A	0.0264	0.0047	-0.0032	0.0368
rs62018216	T	0.0113	0.0019	-0.0066	0.0121
rs62051146	A	-0.0134	0.0022	0.0071	0.0122
rs62090515	A	-0.0090	0.0015	0.0231	0.0085
rs62092949	T	-0.0087	0.0014	-0.0033	0.0081
rs62103198	T	-0.0147	0.0025	-0.0212	0.0186
rs62109862	A	0.0136	0.0020	-0.0236	0.0110
rs62142891	A	0.0108	0.0016	-0.0138	0.0100
rs62155350	A	0.0338	0.0053	0.0803	0.0407
rs62155770	A	-0.0196	0.0032	-0.0111	0.0213
rs62155873	T	-0.0151	0.0021	-0.0017	0.0136
rs62174974	A	-0.0105	0.0018	0.0237	0.0095
rs62177359	A	-0.0275	0.0038	0.0216	0.0291
rs62179650	A	0.0112	0.0016	0.0109	0.0094
rs62182994	T	0.0137	0.0015	-0.0092	0.0097
rs62190914	T	0.0089	0.0015	0.0091	0.0085
rs62194170	A	0.0111	0.0017	0.0000	0.0095
rs622169	T	0.0086	0.0014	-0.0158	0.0097
rs62247449	C	0.0114	0.0014	0.0045	0.0078
rs62252819	A	-0.0094	0.0017	-0.0043	0.0103
rs62256284	T	0.0111	0.0017	0.0001	0.0098
rs62260764	C	-0.0132	0.0016	-0.0054	0.0102
rs62262671	A	0.0213	0.0020	0.0020	0.0138
rs62340636	T	0.0103	0.0014	-0.0105	0.0080
rs62370510	A	-0.0109	0.0019	0.0277	0.0115
rs62379838	T	0.0121	0.0015	-0.0005	0.0095
rs62409395	T	0.0101	0.0018	-0.0072	0.0137
rs62420387	T	-0.0184	0.0027	0.0002	0.0174
rs62506074	T	0.0091	0.0015	-0.0024	0.0091
rs62506104	A	-0.0098	0.0018	0.0008	0.0105
rs628993	A	0.0128	0.0023	0.0276	0.0122

rs631287	A	0.0084	0.0014	0.0045	0.0078
rs633279	A	-0.0089	0.0015	0.0134	0.0086
rs635754	A	0.0119	0.0014	-0.0044	0.0078
rs6428587	T	0.0084	0.0015	-0.0027	0.0081
rs6435326	A	-0.0091	0.0014	-0.0170	0.0081
rs6442126	A	-0.0121	0.0015	0.0090	0.0093
rs6445633	A	0.0088	0.0015	-0.0021	0.0096
rs6449503	A	0.0185	0.0014	-0.0038	0.0088
rs6450476	A	-0.0116	0.0015	0.0162	0.0089
rs6452793	T	0.0175	0.0017	-0.0147	0.0100
rs6461536	A	0.0088	0.0015	-0.0169	0.0087
rs6469654	C	-0.0103	0.0017	0.0051	0.0091
rs6472208	T	-0.0091	0.0014	0.0164	0.0087
rs6480234	T	-0.0084	0.0014	0.0182	0.0081
rs648044	A	0.0083	0.0015	0.0043	0.0089
rs6490618	T	0.0088	0.0015	-0.0096	0.0083
rs6493265	T	-0.0122	0.0014	0.0179	0.0085
rs6493275	A	0.0131	0.0017	0.0070	0.0102
rs6503409	T	0.0087	0.0015	-0.0035	0.0089
rs6534338	T	0.0103	0.0015	-0.0051	0.0086
rs6543810	T	0.0082	0.0015	-0.0012	0.0085
rs6557171	T	-0.0155	0.0015	0.0040	0.0083
rs6567288	A	-0.0079	0.0014	0.0277	0.0081
rs6569077	T	0.0118	0.0014	-0.0049	0.0082
rs6573552	T	-0.0090	0.0014	0.0079	0.0080
rs6573559	T	0.0105	0.0015	0.0055	0.0083
rs660001	A	-0.0164	0.0017	0.0200	0.0096
rs663251	T	0.0081	0.0015	-0.0070	0.0080
rs66482320	C	0.0124	0.0022	-0.0113	0.0121
rs66568921	T	-0.0181	0.0015	0.0109	0.0082
rs66671632	T	-0.0133	0.0021	-0.0137	0.0116
rs66721975	A	-0.0087	0.0015	0.0036	0.0091
rs6672986	C	0.0094	0.0014	-0.0081	0.0080
rs6689641	A	0.0078	0.0014	-0.0034	0.0081
rs6690195	T	-0.0113	0.0014	-0.0080	0.0087
rs6695132	T	0.0097	0.0017	-0.0019	0.0092
rs6696068	T	-0.0088	0.0014	0.0047	0.0081
rs6697584	T	-0.0126	0.0017	-0.0001	0.0100
rs6704768	A	-0.0116	0.0014	0.0099	0.0077
rs6706275	T	0.0083	0.0015	-0.0058	0.0094
rs6711399	T	-0.0107	0.0019	-0.0083	0.0095
rs6715321	T	-0.0097	0.0014	0.0156	0.0082
rs6715849	A	-0.0122	0.0014	0.0114	0.0079
rs6720515	A	0.0103	0.0017	-0.0004	0.0090
rs67224963	A	0.0113	0.0017	-0.0214	0.0095
rs6729612	T	0.0092	0.0015	0.0149	0.0086

rs6731373	A	-0.0115	0.0015	-0.0018	0.0091
rs6736025	T	0.0079	0.0014	0.0037	0.0079
rs6738860	A	-0.0088	0.0014	-0.0145	0.0085
rs6743032	A	-0.0165	0.0024	-0.0119	0.0144
rs6744040	A	-0.0080	0.0014	-0.0155	0.0079
rs67456868	A	0.0119	0.0018	-0.0184	0.0112
rs6752228	T	-0.0080	0.0014	-0.0037	0.0079
rs6757087	T	-0.0085	0.0014	-0.0008	0.0084
rs6760887	T	0.0155	0.0022	0.0005	0.0102
rs67661275	A	0.0134	0.0025	-0.0320	0.0173
rs6774533	T	0.0090	0.0015	-0.0018	0.0086
rs67790232	A	0.0101	0.0017	-0.0088	0.0101
rs6780414	T	0.0105	0.0018	-0.0043	0.0102
rs6792805	A	-0.0090	0.0016	0.0043	0.0088
rs6812533	T	0.0106	0.0016	0.0112	0.0090
rs68145588	T	-0.0127	0.0021	-0.0146	0.0120
rs6824567	T	0.0130	0.0016	-0.0063	0.0095
rs6853599	T	0.0108	0.0019	-0.0346	0.0120
rs6861925	C	-0.0090	0.0014	0.0043	0.0079
rs6871635	A	-0.0087	0.0014	0.0073	0.0081
rs6881581	A	-0.0096	0.0015	0.0094	0.0086
rs6881733	T	-0.0086	0.0016	0.0073	0.0084
rs6917154	T	0.0127	0.0021	-0.0031	0.0140
rs6917204	T	0.0119	0.0017	-0.0125	0.0092
rs6918506	C	0.0113	0.0014	0.0057	0.0080
rs6924023	A	0.0107	0.0016	-0.0018	0.0093
rs6932108	C	-0.0210	0.0027	0.0220	0.0184
rs6946136	A	0.0081	0.0014	-0.0047	0.0084
rs6946362	T	0.0091	0.0015	-0.0004	0.0090
rs6951996	A	-0.0182	0.0032	-0.0013	0.0177
rs6956283	T	0.0098	0.0016	0.0090	0.0092
rs6969783	A	-0.0098	0.0014	0.0093	0.0079
rs6977237	T	0.0082	0.0014	0.0139	0.0079
rs6989141	A	0.0100	0.0017	-0.0375	0.0109
rs6994287	A	0.0102	0.0014	0.0002	0.0080
rs7012546	T	0.0092	0.0014	-0.0114	0.0082
rs7016302	C	-0.0125	0.0019	-0.0178	0.0113
rs702606	T	0.0118	0.0021	0.0122	0.0118
rs7029718	A	0.0244	0.0014	-0.0136	0.0081
rs7030373	A	-0.0098	0.0017	0.0089	0.0089
rs7035315	A	-0.0090	0.0015	-0.0032	0.0081
rs7040995	C	0.0097	0.0014	0.0117	0.0079
rs7041702	A	-0.0111	0.0016	0.0028	0.0092
rs7084508	T	-0.0085	0.0015	0.0005	0.0088
rs710629	A	0.0082	0.0015	0.0053	0.0084
rs7108020	A	0.0111	0.0015	0.0001	0.0090

rs711793	T	0.0103	0.0015	0.0001	0.0096
rs7127580	C	-0.0150	0.0023	0.0025	0.0135
rs713584	A	-0.0080	0.0014	0.0006	0.0083
rs71413877	A	0.0351	0.0036	-0.0397	0.0254
rs7146625	A	0.0091	0.0016	-0.0046	0.0090
rs7147473	A	-0.0103	0.0015	0.0047	0.0093
rs7158218	A	-0.0090	0.0015	0.0207	0.0092
rs71646142	T	0.0104	0.0018	0.0087	0.0113
rs716513	A	0.0084	0.0014	0.0100	0.0082
rs7167688	T	-0.0094	0.0014	0.0005	0.0081
rs7171405	A	-0.0101	0.0016	0.0002	0.0092
rs717996	T	-0.0122	0.0014	-0.0012	0.0084
rs7182216	C	-0.0096	0.0017	-0.0032	0.0105
rs7190	A	-0.0095	0.0015	0.0132	0.0093
rs721579	T	-0.0110	0.0016	-0.0094	0.0089
rs7218235	A	-0.0097	0.0017	0.0078	0.0105
rs7223311	C	-0.0079	0.0014	0.0153	0.0080
rs7226824	T	-0.0078	0.0014	0.0009	0.0079
rs72482130	T	0.0141	0.0019	-0.0165	0.0113
rs72486027	T	-0.0104	0.0016	0.0213	0.0096
rs7254263	T	-0.0119	0.0016	0.0095	0.0089
rs7255223	A	0.0097	0.0016	-0.0112	0.0089
rs72622559	T	-0.0120	0.0017	0.0133	0.0093
rs72624911	T	0.0282	0.0034	-0.0212	0.0259
rs72636697	T	-0.0140	0.0020	0.0063	0.0112
rs72667460	T	0.0197	0.0031	-0.0343	0.0208
rs72671456	A	-0.0174	0.0020	0.0336	0.0127
rs72672052	A	-0.0101	0.0018	-0.0008	0.0102
rs72673097	A	0.0257	0.0042	-0.0738	0.0324
rs72677177	A	0.0100	0.0014	0.0047	0.0086
rs72694479	T	0.0127	0.0020	-0.0090	0.0127
rs72709560	A	0.0085	0.0015	-0.0123	0.0092
rs72771860	T	-0.0211	0.0029	-0.0064	0.0218
rs72792395	T	-0.0145	0.0022	0.0041	0.0135
rs728054	A	-0.0128	0.0015	0.0241	0.0087
rs72819118	T	-0.0231	0.0024	0.0255	0.0159
rs72824753	T	0.0133	0.0024	-0.0227	0.0145
rs72828517	T	-0.0168	0.0019	0.0071	0.0108
rs72829857	A	-0.0149	0.0016	-0.0050	0.0096
rs72834698	A	0.0117	0.0020	-0.0127	0.0133
rs72881110	A	0.0146	0.0027	-0.0276	0.0157
rs72883760	A	0.0101	0.0018	0.0069	0.0105
rs72896637	T	0.0162	0.0028	-0.0073	0.0191
rs72902523	T	-0.0103	0.0016	-0.0076	0.0089
rs72906124	T	0.0185	0.0030	-0.0342	0.0190
rs72917504	T	-0.0178	0.0030	0.0162	0.0192

rs72919450	T	-0.0132	0.0023	0.0232	0.0125
rs72944064	T	-0.0098	0.0016	0.0085	0.0098
rs72962169	T	-0.0174	0.0019	0.0079	0.0116
rs72972965	A	0.0083	0.0015	0.0118	0.0087
rs72993796	T	0.0127	0.0022	-0.0091	0.0148
rs73034295	A	0.0099	0.0018	-0.0166	0.0115
rs730384	A	0.0101	0.0014	-0.0032	0.0082
rs73039077	C	0.0092	0.0016	-0.0033	0.0101
rs73055556	A	0.0111	0.0020	-0.0400	0.0108
rs73082325	C	-0.0252	0.0044	0.0017	0.0331
rs73106136	T	-0.0147	0.0026	-0.0138	0.0166
rs73154546	A	0.0197	0.0036	-0.0312	0.0248
rs7317761	C	0.0109	0.0016	-0.0012	0.0088
rs73191311	A	-0.0094	0.0015	-0.0017	0.0086
rs7321274	A	0.0114	0.0018	0.0024	0.0100
rs73219806	A	0.0106	0.0019	-0.0222	0.0101
rs7323027	A	0.0130	0.0015	0.0027	0.0083
rs7326331	A	-0.0128	0.0016	0.0228	0.0090
rs73344830	A	0.0170	0.0014	0.0025	0.0082
rs73405293	A	0.0113	0.0020	0.0180	0.0112
rs73457936	A	0.0144	0.0025	0.0085	0.0160
rs73518807	C	-0.0128	0.0021	-0.0068	0.0091
rs7356536	T	-0.0129	0.0021	0.0142	0.0130
rs73581580	A	-0.0181	0.0022	0.0271	0.0169
rs736281	T	0.0086	0.0014	0.0013	0.0084
rs73643713	T	-0.0165	0.0027	-0.0224	0.0144
rs736471	T	0.0083	0.0014	0.0066	0.0083
rs73648455	T	-0.0183	0.0026	0.0060	0.0140
rs737945	C	-0.0115	0.0014	0.0137	0.0081
rs73874335	T	-0.0178	0.0029	-0.0042	0.0146
rs73961845	A	-0.0114	0.0014	0.0045	0.0085
rs74091672	A	-0.0153	0.0022	0.0014	0.0147
rs7430651	T	-0.0111	0.0016	0.0159	0.0090
rs743316	T	0.0112	0.0017	-0.0005	0.0109
rs74415461	T	0.0176	0.0026	0.0009	0.0168
rs74453875	A	0.0214	0.0037	0.0329	0.0197
rs74462621	T	0.0125	0.0020	0.0132	0.0127
rs7449561	A	0.0102	0.0017	0.0081	0.0093
rs7451726	A	0.0109	0.0017	0.0030	0.0093
rs74545339	A	-0.0158	0.0022	-0.0127	0.0132
rs7460106	T	-0.0123	0.0017	-0.0218	0.0098
rs74615093	A	0.0145	0.0026	-0.0316	0.0151
rs746839	C	0.0135	0.0015	-0.0076	0.0091
rs74747621	T	0.0153	0.0022	-0.0283	0.0137
rs74787922	A	0.0182	0.0028	0.0055	0.0176
rs74944275	T	0.0231	0.0035	0.0307	0.0233

rs74944857	T	-0.0089	0.0015	-0.0092	0.0096
rs7495033	A	0.0095	0.0015	-0.0084	0.0087
rs75033012	C	-0.0258	0.0038	-0.0025	0.0292
rs75177132	T	0.0298	0.0036	-0.0217	0.0288
rs75203411	T	0.0135	0.0019	-0.0008	0.0107
rs7526112	T	0.0124	0.0015	0.0012	0.0084
rs75308819	A	0.0287	0.0047	0.0221	0.0353
rs75434274	A	0.0151	0.0026	-0.0014	0.0184
rs75500877	T	0.0207	0.0033	-0.0043	0.0205
rs7552964	A	0.0148	0.0023	-0.0095	0.0149
rs7560871	A	-0.0193	0.0027	0.0261	0.0167
rs7561705	A	0.0078	0.0014	0.0120	0.0080
rs7575637	A	0.0107	0.0014	-0.0073	0.0078
rs75756843	A	0.0288	0.0049	0.0884	0.0336
rs7575938	A	0.0090	0.0015	-0.0072	0.0085
rs7590368	T	-0.0146	0.0016	0.0057	0.0094
rs7597126	T	-0.0094	0.0014	0.0001	0.0086
rs7597412	C	0.0088	0.0015	-0.0180	0.0089
rs7603132	A	0.0137	0.0018	0.0039	0.0099
rs76076331	T	0.0206	0.0021	-0.0229	0.0128
rs76077165	A	0.0165	0.0025	-0.0073	0.0163
rs76167224	T	-0.0187	0.0033	0.0690	0.0262
rs7617204	A	-0.0097	0.0014	0.0015	0.0081
rs76235882	A	0.0241	0.0039	-0.0104	0.0253
rs76241605	A	0.0139	0.0022	0.0064	0.0151
rs76246107	A	-0.0162	0.0026	0.0060	0.0197
rs7625428	T	0.0098	0.0014	0.0095	0.0084
rs76267866	A	0.0104	0.0017	0.0027	0.0106
rs7630133	A	0.0082	0.0015	-0.0029	0.0088
rs763553	A	0.0087	0.0014	0.0154	0.0083
rs7640424	T	0.0113	0.0015	-0.0127	0.0097
rs7650602	T	-0.0089	0.0014	-0.0071	0.0082
rs76552497	T	-0.0120	0.0019	0.0157	0.0107
rs76577427	C	0.0203	0.0024	-0.0182	0.0160
rs7672622	A	-0.0088	0.0016	0.0015	0.0088
rs767943	A	-0.0122	0.0016	0.0052	0.0092
rs7683416	T	0.0139	0.0014	0.0031	0.0080
rs76876592	A	0.0126	0.0020	-0.0075	0.0123
rs76878669	C	0.0141	0.0017	-0.0022	0.0092
rs7692359	T	-0.0109	0.0017	0.0044	0.0105
rs76957677	T	-0.0297	0.0054	0.0526	0.0442
rs77025239	A	-0.0140	0.0019	-0.0070	0.0102
rs7714719	C	0.0116	0.0020	-0.0025	0.0114
rs7716161	C	0.0105	0.0019	0.0155	0.0112
rs77201694	A	0.0156	0.0022	-0.0156	0.0154
rs77370942	A	-0.0147	0.0027	0.0090	0.0176

rs77554090	T	-0.0186	0.0027	-0.0061	0.0173
rs77609760	A	0.0236	0.0028	-0.0027	0.0185
rs7766240	T	-0.0134	0.0024	0.0007	0.0135
rs77702622	A	-0.0213	0.0029	0.0030	0.0198
rs77702819	T	0.0187	0.0025	-0.0077	0.0158
rs7772172	A	0.0091	0.0014	-0.0011	0.0090
rs7775100	T	-0.0087	0.0014	0.0133	0.0079
rs77826402	T	0.0151	0.0027	0.0275	0.0154
rs7796103	C	-0.0077	0.0014	0.0192	0.0084
rs7799141	A	0.0099	0.0015	0.0066	0.0085
rs77999825	A	0.0294	0.0039	-0.0102	0.0286
rs7803932	A	0.0127	0.0019	-0.0169	0.0117
rs780569	A	-0.0085	0.0016	0.0085	0.0087
rs78116078	C	0.0086	0.0016	0.0059	0.0099
rs7816777	T	-0.0088	0.0015	0.0017	0.0087
rs78193153	A	0.0191	0.0029	0.0037	0.0129
rs7823700	T	0.0132	0.0024	-0.0069	0.0169
rs78365243	T	0.0181	0.0033	-0.0199	0.0212
rs78440611	A	-0.0147	0.0024	0.0039	0.0153
rs7849480	A	-0.0084	0.0014	0.0203	0.0080
rs7849487	T	-0.0167	0.0015	-0.0203	0.0081
rs7855503	C	0.0088	0.0015	0.0117	0.0083
rs78648104	T	-0.0141	0.0025	0.0584	0.0142
rs78702390	C	0.0167	0.0030	-0.0433	0.0183
rs78714229	T	0.0172	0.0030	-0.0076	0.0192
rs7875078	A	-0.0078	0.0014	0.0204	0.0081
rs78918150	T	-0.0080	0.0015	0.0098	0.0088
rs7894722	T	0.0085	0.0014	-0.0062	0.0084
rs7899270	A	-0.0084	0.0015	-0.0014	0.0088
rs7905192	T	0.0084	0.0014	0.0011	0.0083
rs790647	A	-0.0145	0.0017	0.0117	0.0091
rs7910403	T	0.0122	0.0018	-0.0015	0.0112
rs7914674	T	-0.0139	0.0022	-0.0185	0.0099
rs7920624	A	0.0120	0.0014	-0.0232	0.0080
rs7924036	T	0.0133	0.0014	-0.0064	0.0080
rs79265434	A	-0.0197	0.0022	0.0390	0.0132
rs7928017	A	0.0096	0.0014	-0.0160	0.0081
rs7931563	T	-0.0109	0.0014	0.0018	0.0081
rs795230	T	0.0081	0.0014	0.0031	0.0081
rs7958371	A	-0.0085	0.0015	-0.0047	0.0084
rs79585412	T	-0.0178	0.0031	0.0036	0.0175
rs7965154	A	-0.0085	0.0015	0.0028	0.0085
rs7967550	A	-0.0086	0.0014	0.0068	0.0085
rs7972246	T	0.0110	0.0015	-0.0020	0.0085
rs79728014	A	-0.0134	0.0020	0.0051	0.0126
rs7974852	A	0.0117	0.0014	0.0100	0.0079

rs7977614	A	-0.0134	0.0016	-0.0133	0.0096
rs79798166	A	0.0172	0.0025	0.0082	0.0165
rs79855925	T	-0.0194	0.0033	-0.0114	0.0226
rs79994730	T	0.0240	0.0035	0.0617	0.0244
rs79997166	A	0.0172	0.0030	0.0364	0.0208
rs80037907	T	-0.0107	0.0019	0.0186	0.0118
rs8008382	T	-0.0106	0.0015	0.0069	0.0085
rs8009933	A	0.0088	0.0015	-0.0068	0.0083
rs8016504	A	-0.0087	0.0014	-0.0032	0.0077
rs80223410	T	-0.0118	0.0020	0.0273	0.0143
rs8024	A	-0.0115	0.0015	0.0254	0.0089
rs80257979	T	-0.0252	0.0040	-0.0009	0.0260
rs8030487	A	0.0085	0.0015	-0.0218	0.0095
rs803619	T	-0.0173	0.0023	0.0057	0.0132
rs8046072	A	0.0101	0.0018	0.0006	0.0104
rs8052523	T	-0.0084	0.0014	0.0068	0.0085
rs8066044	A	0.0090	0.0016	-0.0233	0.0090
rs806816	A	0.0101	0.0016	0.0173	0.0100
rs8097125	T	0.0086	0.0014	0.0140	0.0086
rs8103741	A	-0.0108	0.0019	0.0019	0.0105
rs818415	T	-0.0110	0.0018	0.0178	0.0097
rs8192465	A	-0.0272	0.0049	0.0545	0.0373
rs852771	T	0.0092	0.0015	-0.0039	0.0091
rs853286	T	0.0124	0.0023	-0.0111	0.0114
rs854796	A	0.0090	0.0015	-0.0046	0.0086
rs868456	A	0.0089	0.0015	0.0056	0.0089
rs870589	A	-0.0079	0.0014	0.0002	0.0080
rs884108	A	-0.0094	0.0017	-0.0140	0.0097
rs891793	C	-0.0099	0.0014	0.0082	0.0080
rs893522	A	0.0149	0.0025	-0.0153	0.0151
rs902820	A	-0.0096	0.0014	-0.0013	0.0080
rs911149	T	0.0091	0.0017	0.0000	0.0099
rs912883	T	0.0087	0.0015	0.0136	0.0083
rs913509	A	0.0096	0.0015	0.0027	0.0085
rs925161	C	-0.0078	0.0014	0.0081	0.0081
rs9267658	T	0.0138	0.0020	-0.0209	0.0136
rs9267677	T	0.0180	0.0024	0.0240	0.0147
rs9289300	T	-0.0164	0.0019	-0.0026	0.0115
rs9291437	C	-0.0081	0.0014	-0.0035	0.0080
rs9294770	T	0.0083	0.0014	0.0035	0.0083
rs929511	T	-0.0160	0.0021	0.0139	0.0121
rs9300612	T	0.0084	0.0015	0.0042	0.0083
rs933738	A	-0.0110	0.0018	-0.0045	0.0104
rs9349956	A	-0.0159	0.0018	0.0158	0.0116
rs9359939	A	-0.0105	0.0016	-0.0101	0.0091
rs936496	A	-0.0079	0.0014	0.0134	0.0085

rs9371883	C	-0.0087	0.0015	0.0066	0.0086
rs9373363	A	-0.0111	0.0016	-0.0104	0.0087
rs9375188	T	0.0212	0.0014	0.0015	0.0079
rs9375403	C	0.0126	0.0016	-0.0049	0.0099
rs9386110	T	-0.0163	0.0023	0.0059	0.0124
rs9388490	T	0.0091	0.0014	0.0163	0.0086
rs939400	T	-0.0095	0.0015	0.0114	0.0083
rs9411331	A	0.0145	0.0015	-0.0240	0.0083
rs9435340	A	0.0086	0.0015	-0.0002	0.0082
rs9442750	A	-0.0089	0.0016	-0.0079	0.0097
rs9446060	A	0.0077	0.0014	-0.0099	0.0082
rs9465509	A	-0.0084	0.0014	-0.0009	0.0080
rs9490512	A	-0.0136	0.0014	0.0000	0.0079
rs9492774	C	0.0080	0.0015	-0.0028	0.0079
rs9513416	A	-0.0116	0.0019	-0.0203	0.0122
rs9513754	T	0.0096	0.0016	0.0079	0.0091
rs9527662	A	0.0094	0.0014	-0.0017	0.0091
rs9527905	A	-0.0094	0.0014	0.0134	0.0080
rs9529146	T	0.0127	0.0017	0.0024	0.0090
rs9536462	A	0.0133	0.0020	0.0088	0.0106
rs9540718	A	-0.0089	0.0014	-0.0105	0.0080
rs9545395	T	0.0138	0.0021	-0.0204	0.0107
rs9556958	T	-0.0110	0.0014	0.0109	0.0075
rs9563168	A	0.0101	0.0017	-0.0208	0.0100
rs9568798	T	-0.0093	0.0016	-0.0068	0.0091
rs9597907	T	0.0152	0.0027	-0.0127	0.0158
rs9611597	A	-0.0107	0.0019	0.0011	0.0128
rs9616906	A	0.0122	0.0014	-0.0177	0.0086
rs9616947	T	-0.0134	0.0015	0.0116	0.0087
rs9633970	T	0.0091	0.0016	-0.0146	0.0093
rs9649	T	0.0108	0.0019	-0.0175	0.0122
rs9655780	A	-0.0153	0.0019	-0.0130	0.0119
rs9666728	T	-0.0082	0.0014	0.0162	0.0079
rs9683585	C	0.0084	0.0014	0.0073	0.0078
rs969512	A	-0.0106	0.0015	0.0051	0.0085
rs9771228	T	0.0096	0.0015	-0.0075	0.0086
rs977143	A	0.0085	0.0015	-0.0067	0.0088
rs978807	A	0.0139	0.0018	-0.0070	0.0095
rs981230	T	0.0100	0.0014	-0.0087	0.0084
rs981883	A	0.0099	0.0014	-0.0232	0.0080
rs9820604	T	0.0115	0.0018	-0.0056	0.0098
rs9821664	A	0.0094	0.0017	-0.0073	0.0092
rs9830359	T	-0.0147	0.0022	0.0128	0.0122
rs9844755	A	0.0083	0.0015	-0.0032	0.0085
rs9849884	A	-0.0090	0.0014	-0.0011	0.0084
rs9853928	T	-0.0127	0.0017	0.0062	0.0089

rs9858921	A	-0.0087	0.0014	0.0075	0.0078
rs9859556	T	0.0290	0.0015	-0.0071	0.0094
rs9866123	A	0.0086	0.0014	-0.0034	0.0081
rs9870317	A	0.0086	0.0015	0.0093	0.0082
rs9877225	T	0.0090	0.0016	-0.0128	0.0093
rs9882532	T	0.0124	0.0015	0.0005	0.0087
rs9886703	A	-0.0136	0.0019	-0.0060	0.0099
rs9888796	T	0.0113	0.0016	0.0008	0.0095
rs9916901	T	0.0092	0.0016	-0.0071	0.0085
rs9924031	C	0.0093	0.0015	-0.0112	0.0091
rs9926649	T	0.0177	0.0031	-0.0197	0.0171
rs9927049	A	0.0099	0.0016	0.0165	0.0099
rs9927137	A	0.0082	0.0014	0.0033	0.0087
rs9927842	T	-0.0130	0.0020	0.0065	0.0107
rs9929556	T	-0.0099	0.0014	0.0020	0.0081
rs9929762	A	0.0096	0.0014	-0.0053	0.0079
rs9929993	T	-0.0102	0.0015	0.0093	0.0081
rs9933256	A	0.0119	0.0014	-0.0138	0.0092
rs995698	A	-0.0099	0.0014	-0.0107	0.0086
rs9974899	T	-0.0093	0.0016	-0.0068	0.0098
rs9977825	T	-0.0092	0.0015	0.0178	0.0090

Supplementary Table 13: Genetic association estimates for the two-sample regression-based MVMR analysis estimating the effect of SBP on CAD, adjusted for education. ea=effect allele; gx=SBP; gy=CAD; gz=education; se=standard error

SNP	ea	gx	gx_se	gy	gy_se	gz	gz_se
rs10024506	G	0.3036	0.0521	0.0131	0.0102	0.0004	0.0020
rs10048404	C	0.2683	0.0458	0.0192	0.0100	-0.0055	0.0018
rs1010064	A	0.4007	0.0571	0.0401	0.0114	-0.0015	0.0022
rs10195405	A	0.2918	0.0486	0.0166	0.0104	-0.0035	0.0019
rs10212058	A	0.3278	0.0558	0.0355	0.0122	-0.0003	0.0022
rs1051447	C	0.2715	0.0489	0.0065	0.0099	0.0003	0.0019
rs10775143	C	0.2586	0.0453	-0.0066	0.0096	0.0075	0.0017
rs1077795	A	0.2939	0.0503	0.0193	0.0111	0.0000	0.0019
rs10817007	T	-0.4373	0.0661	-0.0231	0.0131	0.0045	0.0026
rs10883543	G	-0.6091	0.0705	0.0147	0.0168	0.0000	0.0027
rs10977670	A	0.3011	0.0463	-0.0118	0.0097	0.0012	0.0018
rs10995311	C	0.3440	0.0446	-0.0113	0.0096	0.0059	0.0017
rs11070245	T	-0.2798	0.0444	0.0050	0.0093	-0.0052	0.0017
rs11145807	A	0.2734	0.0453	0.0081	0.0107	0.0017	0.0018
rs11146456	A	-0.4230	0.0695	-0.0022	0.0190	0.0026	0.0027
rs11187838	G	0.4660	0.0446	-0.0242	0.0093	0.0028	0.0017
rs11191580	T	0.9647	0.0829	0.0732	0.0142	0.0022	0.0031
rs113044050	C	0.3637	0.0641	0.0232	0.0130	-0.0046	0.0024
rs113523959	G	0.3852	0.0627	-0.0031	0.0162	-0.0046	0.0025
rs113605702	G	0.4460	0.0798	0.0097	0.0171	-0.0134	0.0031
rs115262049	A	0.4875	0.0780	0.0321	0.0200	-0.0032	0.0030
rs11605215	G	-0.3191	0.0559	-0.0157	0.0117	-0.0014	0.0021
rs11616710	C	-0.5056	0.0778	-0.0179	0.0196	0.0028	0.0029
rs11641308	T	-0.3272	0.0470	-0.0311	0.0104	0.0008	0.0018
rs11667829	G	-0.2636	0.0472	-0.0359	0.0108	0.0046	0.0018
rs116734066	C	0.4365	0.0764	-0.0227	0.0164	0.0024	0.0030
rs11697820	C	0.2499	0.0451	0.0068	0.0096	-0.0020	0.0017
rs11719057	G	0.2714	0.0467	0.0253	0.0100	-0.0014	0.0018
rs11721038	T	0.4978	0.0841	0.0464	0.0154	-0.0024	0.0032
rs1173771	A	-0.6369	0.0450	-0.0119	0.0093	0.0000	0.0017
rs11774829	T	0.4089	0.0739	0.0209	0.0175	0.0029	0.0028
rs11795365	G	-0.3642	0.0642	0.0185	0.0129	-0.0015	0.0025
rs11821781	A	-0.3392	0.0507	-0.0038	0.0109	0.0026	0.0020
rs11874	G	-0.6251	0.0642	-0.0497	0.0156	0.0068	0.0025
rs12035750	T	-0.3499	0.0454	-0.0138	0.0101	0.0085	0.0018
rs12055028	G	-0.3149	0.0479	0.0012	0.0098	0.0000	0.0018
rs12147852	G	-0.3294	0.0516	-0.0149	0.0110	-0.0012	0.0020
rs12258967	C	0.6150	0.0484	0.0257	0.0112	0.0011	0.0019
rs1229984	T	-1.0787	0.1483	-0.0333	0.0186	0.0229	0.0057
rs1250258	C	0.3174	0.0501	0.0328	0.0108	0.0021	0.0019
rs12509892	T	-0.3709	0.0443	-0.0397	0.0094	0.0037	0.0017

rs12543884	A	-0.3423	0.0579	-0.0105	0.0111	-0.0027	0.0022
rs12544711	G	0.2944	0.0449	0.0011	0.0093	-0.0002	0.0017
rs12567136	C	0.7688	0.0595	0.0232	0.0128	0.0015	0.0023
rs12608469	T	-0.5593	0.0935	0.0495	0.0212	-0.0057	0.0036
rs1262894	A	0.4733	0.0836	-0.0130	0.0191	-0.0053	0.0034
rs1263146	G	0.2574	0.0444	-0.0026	0.0095	-0.0045	0.0017
rs12643599	A	0.3161	0.0459	0.0512	0.0095	-0.0011	0.0018
rs12699561	G	-0.2481	0.0456	-0.0246	0.0098	0.0055	0.0018
rs12705390	G	-0.7711	0.0555	-0.0174	0.0110	0.0051	0.0021
rs12822344	C	-0.2893	0.0448	-0.0109	0.0095	0.0005	0.0017
rs12906962	T	-0.2770	0.0477	-0.0166	0.0102	-0.0041	0.0018
rs1290784	C	-0.3710	0.0446	-0.0114	0.0094	-0.0011	0.0017
rs13107325	C	0.6251	0.0841	0.0067	0.0223	0.0188	0.0034
rs13129779	C	0.2546	0.0446	0.0251	0.0094	-0.0004	0.0017
rs13219548	C	-0.4362	0.0445	-0.0066	0.0092	0.0009	0.0017
rs13436194	A	0.3628	0.0446	0.0206	0.0093	0.0039	0.0017
rs137923903	C	1.2912	0.1937	0.0361	0.0454	0.0046	0.0078
rs1397985	G	0.3098	0.0473	0.0075	0.0099	0.0015	0.0018
rs1422279	C	-0.3138	0.0456	-0.0060	0.0096	-0.0045	0.0018
rs1436138	A	0.3017	0.0463	0.0081	0.0098	0.0029	0.0018
rs145339349	G	-1.1270	0.1676	-0.0356	0.0429	-0.0010	0.0065
rs1458038	C	-0.7179	0.0487	-0.0461	0.0102	-0.0028	0.0019
rs149932962	G	-0.3024	0.0511	0.0241	0.0123	0.0014	0.0020
rs150857355	G	-0.8776	0.1531	-0.0783	0.0444	-0.0029	0.0060
rs151157965	G	-0.4568	0.0751	0.0079	0.0162	0.0059	0.0029
rs1530558	T	0.3654	0.0643	-0.0072	0.0152	-0.0035	0.0027
rs1542977	G	-0.2805	0.0443	-0.0295	0.0093	0.0065	0.0017
rs1543270	C	0.3593	0.0445	-0.0226	0.0094	-0.0028	0.0017
rs1543927	T	0.4427	0.0502	0.0205	0.0101	-0.0087	0.0019
rs167479	G	0.5274	0.0442	0.0146	0.0135	0.0009	0.0020
rs17011002	C	-0.5018	0.0638	-0.0103	0.0130	0.0041	0.0025
rs17173238	A	-0.2918	0.0489	-0.0154	0.0113	-0.0018	0.0019
rs1757915	G	-0.3347	0.0461	-0.0185	0.0096	-0.0007	0.0018
rs17677603	A	-0.3326	0.0454	-0.0283	0.0096	-0.0027	0.0018
rs1779240	G	0.3653	0.0521	0.0183	0.0107	-0.0032	0.0020
rs1887320	G	-0.3471	0.0444	-0.0226	0.0091	0.0020	0.0017
rs2017199	G	0.3274	0.0485	-0.0030	0.0106	-0.0007	0.0019
rs2052692	A	0.2673	0.0475	-0.0084	0.0108	0.0064	0.0018
rs2062316	A	-0.3519	0.0445	-0.0107	0.0100	0.0030	0.0017
rs2111834	G	0.4380	0.0684	-0.0223	0.0163	-0.0107	0.0027
rs2129561	A	-0.3354	0.0451	-0.0166	0.0094	0.0004	0.0017
rs2177843	C	-0.4184	0.0628	0.0015	0.0125	0.0024	0.0024
rs2237306	C	0.3706	0.0642	0.0023	0.0126	-0.0205	0.0024
rs2249105	A	0.3532	0.0458	-0.0033	0.0097	-0.0039	0.0018
rs2285815	C	0.2952	0.0500	0.0184	0.0101	-0.0039	0.0019
rs2288276	G	-0.4101	0.0729	-0.0071	0.0136	-0.0017	0.0028

rs2301597	T	0.4335	0.0448	-0.0002	0.0094	-0.0052	0.0017
rs2303083	G	0.4692	0.0559	0.0158	0.0125	-0.0105	0.0022
rs2306363	G	0.4059	0.0546	0.0499	0.0117	-0.0052	0.0022
rs231708	G	0.2696	0.0476	0.0035	0.0100	0.0010	0.0018
rs2423514	A	0.3053	0.0443	0.0181	0.0095	0.0005	0.0017
rs2469133	G	-0.2652	0.0466	-0.0220	0.0098	-0.0036	0.0018
rs2469997	G	0.3808	0.0570	-0.0012	0.0127	0.0011	0.0022
rs2493296	C	-0.4144	0.0642	-0.0560	0.0147	-0.0003	0.0025
rs2499801	G	0.3601	0.0569	-0.0518	0.0123	-0.0012	0.0022
rs2606738	T	-0.3227	0.0470	-0.0130	0.0095	0.0065	0.0018
rs2610989	T	-0.3378	0.0503	-0.0239	0.0112	0.0077	0.0019
rs2613203	A	-0.3618	0.0569	-0.0335	0.0118	-0.0040	0.0022
rs263017	A	0.2394	0.0444	0.0017	0.0092	-0.0032	0.0017
rs2643826	C	-0.3654	0.0445	-0.0001	0.0096	0.0014	0.0017
rs2681492	T	0.7161	0.0589	-0.0735	0.0113	0.0021	0.0023
rs268263	T	-0.5684	0.0517	-0.0418	0.0109	-0.0050	0.0020
rs2764834	T	-0.3148	0.0539	-0.0042	0.0109	0.0011	0.0021
rs2782652	T	-0.2821	0.0443	-0.0223	0.0097	-0.0121	0.0017
rs2782980	T	-0.3734	0.0492	-0.0149	0.0106	-0.0027	0.0019
rs2867114	C	0.4161	0.0759	0.0416	0.0126	-0.0053	0.0025
rs2897515	T	0.2629	0.0445	0.0118	0.0093	0.0031	0.0017
rs2978456	T	-0.2517	0.0443	0.0026	0.0101	0.0066	0.0018
rs3101442	T	-0.3048	0.0443	-0.0138	0.0094	0.0017	0.0017
rs3211995	G	0.4331	0.0607	0.0062	0.0141	-0.0090	0.0024
rs34991912	T	0.2671	0.0449	0.0465	0.0094	-0.0003	0.0017
rs35021474	C	0.4695	0.0455	-0.0006	0.0104	-0.0067	0.0018
rs35443	G	0.3896	0.0454	-0.0015	0.0096	0.0009	0.0018
rs35726503	A	0.3459	0.0447	-0.0004	0.0098	-0.0041	0.0017
rs35807464	G	-0.5299	0.0889	-0.0220	0.0184	-0.0087	0.0032
rs3786516	T	-0.3211	0.0538	0.0149	0.0110	-0.0023	0.0020
rs3790604	C	-0.7446	0.0846	-0.0138	0.0161	0.0033	0.0032
rs3826537	A	-0.2872	0.0447	0.0037	0.0100	0.0018	0.0017
rs3827750	C	-0.5341	0.0705	-0.0395	0.0131	-0.0052	0.0027
rs3887266	C	-0.3884	0.0694	-0.0179	0.0143	-0.0007	0.0027
rs4141663	C	0.2757	0.0449	0.0094	0.0093	0.0018	0.0017
rs42038	C	0.3773	0.0484	0.0313	0.0109	0.0011	0.0019
rs4246616	C	-0.2718	0.0445	-0.0007	0.0098	-0.0041	0.0017
rs434578	C	0.3810	0.0634	0.0369	0.0141	-0.0012	0.0025
rs4480845	T	0.4017	0.0463	-0.0173	0.0098	-0.0006	0.0018
rs4648815	G	0.2905	0.0447	0.0050	0.0112	-0.0074	0.0018
rs4666493	G	-0.3577	0.0448	-0.0130	0.0094	-0.0007	0.0017
rs4690775	A	0.2629	0.0451	0.0203	0.0095	0.0031	0.0017
rs4937515	G	0.3455	0.0453	0.0075	0.0103	-0.0046	0.0017
rs55924432	C	-0.2770	0.0451	-0.0213	0.0100	-0.0023	0.0018
rs56352102	C	-0.5273	0.0570	-0.0295	0.0120	-0.0004	0.0022
rs57139556	A	0.5824	0.0857	0.0457	0.0157	-0.0026	0.0033

rs57301765	G	-0.5131	0.0608	-0.0697	0.0113	0.0052	0.0023
rs597808	A	0.4025	0.0443	0.0612	0.0106	-0.0024	0.0017
rs604723	T	-0.6293	0.0497	-0.0327	0.0101	0.0049	0.0019
rs6062625	T	-0.3285	0.0512	-0.0201	0.0118	0.0035	0.0020
rs61148001	C	0.3262	0.0546	0.0086	0.0108	0.0000	0.0021
rs62162674	G	0.2691	0.0445	0.0115	0.0096	0.0006	0.0017
rs62229372	C	-0.3966	0.0672	-0.0043	0.0168	0.0037	0.0026
rs6453400	G	0.2515	0.0445	0.0053	0.0096	-0.0035	0.0017
rs6461992	A	-0.7171	0.0853	0.0089	0.0172	0.0014	0.0032
rs6704991	A	-0.2749	0.0461	-0.0128	0.0099	0.0019	0.0018
rs6708660	T	0.2771	0.0452	-0.0143	0.0097	-0.0002	0.0017
rs6775384	G	-0.2988	0.0441	-0.0294	0.0094	0.0249	0.0017
rs6787069	C	-0.3038	0.0496	0.0119	0.0104	0.0085	0.0019
rs6815273	G	0.2850	0.0450	0.0124	0.0101	0.0002	0.0017
rs6963105	A	-0.2766	0.0446	-0.0167	0.0111	0.0092	0.0018
rs7019061	C	0.2794	0.0489	0.0130	0.0100	-0.0031	0.0019
rs7070797	G	0.5992	0.0635	0.0129	0.0154	-0.0046	0.0025
rs7107356	A	-0.4367	0.0441	-0.0063	0.0093	0.0055	0.0017
rs7123754	T	0.3299	0.0472	0.0164	0.0098	-0.0032	0.0018
rs7125196	T	0.4486	0.0693	0.0172	0.0127	0.0106	0.0026
rs71313932	G	-0.2825	0.0491	-0.0359	0.0105	-0.0010	0.0019
rs7200432	G	0.3351	0.0484	0.0109	0.0101	0.0025	0.0019
rs72683923	T	1.0062	0.1588	0.0390	0.0368	-0.0110	0.0062
rs72792829	C	0.3598	0.0534	0.0131	0.0104	-0.0010	0.0021
rs72812818	G	0.2708	0.0482	0.0109	0.0108	-0.0009	0.0018
rs72843959	C	0.2788	0.0471	-0.0239	0.0271	0.0031	0.0018
rs7302981	A	0.3398	0.0457	0.0003	0.0100	-0.0007	0.0018
rs73046792	G	0.4241	0.0595	0.0163	0.0163	0.0001	0.0024
rs73050466	G	0.8133	0.1316	-0.0128	0.0340	-0.0064	0.0048
rs7306710	T	-0.2759	0.0445	-0.0212	0.0098	0.0082	0.0017
rs73075659	A	0.3378	0.0467	0.0062	0.0106	0.0026	0.0018
rs7315688	G	0.2693	0.0484	0.0191	0.0102	-0.0024	0.0019
rs73563812	G	0.3946	0.0521	0.0202	0.0107	0.0065	0.0020
rs7439366	T	-0.2487	0.0443	0.0001	0.0102	0.0005	0.0017
rs7459106	A	0.3574	0.0562	0.0327	0.0115	-0.0045	0.0022
rs75271047	G	0.4691	0.0800	0.0607	0.0169	-0.0057	0.0031
rs7532726	A	0.2910	0.0500	0.0208	0.0107	-0.0002	0.0019
rs7553325	C	-0.2516	0.0443	0.0201	0.0097	0.0000	0.0017
rs75777337	T	-0.6027	0.0724	-0.0420	0.0159	0.0026	0.0027
rs75989961	T	-0.4855	0.0807	-0.0312	0.0216	0.0027	0.0032
rs77870048	C	-0.9363	0.0989	-0.0144	0.0304	-0.0063	0.0040
rs79349366	C	0.7946	0.1255	-0.0266	0.0351	0.0157	0.0048
rs7938342	T	-0.5398	0.0449	-0.0097	0.0104	0.0015	0.0018
rs7963801	T	-0.2501	0.0448	-0.0218	0.0102	0.0107	0.0017
rs8027450	C	-0.5420	0.0474	-0.0573	0.0109	0.0012	0.0018
rs8076588	C	0.2841	0.0443	0.0088	0.0095	0.0050	0.0017

rs861585	G	-0.2584	0.0445	-0.0075	0.0094	0.0067	0.0017
rs891511	G	0.3979	0.0479	0.0214	0.0109	-0.0042	0.0019
rs9294987	T	-0.2568	0.0445	0.0239	0.0101	-0.0012	0.0017
rs9373523	T	0.2556	0.0450	0.0009	0.0093	-0.0022	0.0017
rs940743	T	-0.2682	0.0444	-0.0333	0.0098	-0.0002	0.0017
rs9472040	A	-0.2797	0.0473	-0.0200	0.0096	-0.0006	0.0018
rs9773022	T	-0.2937	0.0443	0.0078	0.0110	-0.0008	0.0018
rs9907379	T	-0.4026	0.0542	-0.0358	0.0111	-0.0009	0.0021
rs9993149	T	0.2797	0.0442	0.0043	0.0092	-0.0053	0.0017

Supplementary Table 14: Genetic association estimates for the two-sample regression-based MVMR analysis estimating the effect of SBP on MI, adjusted for education. ea=effect allele; gx=SBP; gy=MI; gz=education; se=standard error

SNP	ea	gx	gx_se	gy	gy_se	gz	gz_se
rs10024506	G	0.3036	0.0521	0.0213	0.0114	0.0004	0.0020
rs10048404	C	0.2683	0.0458	0.0068	0.0111	-0.0055	0.0018
rs1010064	A	0.4007	0.0571	0.0406	0.0127	-0.0015	0.0022
rs10195405	A	0.2918	0.0486	0.0086	0.0111	-0.0035	0.0019
rs10212058	A	0.3278	0.0558	0.0274	0.0134	-0.0003	0.0022
rs1051447	C	0.2715	0.0489	0.0131	0.0109	0.0003	0.0019
rs10775143	C	0.2586	0.0453	-0.0001	0.0107	0.0075	0.0017
rs1077795	A	0.2939	0.0503	0.0086	0.0121	0.0000	0.0019
rs10817007	T	-0.4373	0.0661	-0.0317	0.0146	0.0045	0.0026
rs10883543	G	-0.6091	0.0705	0.0193	0.0183	0.0000	0.0027
rs10977670	A	0.3011	0.0463	-0.0025	0.0107	0.0012	0.0018
rs10995311	C	0.3440	0.0446	-0.0162	0.0105	0.0059	0.0017
rs11070245	T	-0.2798	0.0444	0.0075	0.0103	-0.0052	0.0017
rs11145807	A	0.2734	0.0453	0.0155	0.0120	0.0017	0.0018
rs11146456	A	-0.4230	0.0695	-0.0195	0.0212	0.0026	0.0027
rs11187838	G	0.4660	0.0446	-0.0190	0.0104	0.0028	0.0017
rs11191580	T	0.9647	0.0829	0.0729	0.0158	0.0022	0.0031
rs113044050	C	0.3637	0.0641	0.0130	0.0143	-0.0046	0.0024
rs113523959	G	0.3852	0.0627	0.0004	0.0180	-0.0046	0.0025
rs113605702	G	0.4460	0.0798	0.0049	0.0192	-0.0134	0.0031
rs115262049	A	0.4875	0.0780	0.0264	0.0218	-0.0032	0.0030
rs11605215	G	-0.3191	0.0559	0.0029	0.0131	-0.0014	0.0021
rs11616710	C	-0.5056	0.0778	-0.0098	0.0215	0.0028	0.0029
rs11641308	T	-0.3272	0.0470	-0.0149	0.0116	0.0008	0.0018
rs11667829	G	-0.2636	0.0472	-0.0314	0.0118	0.0046	0.0018
rs116734066	C	0.4365	0.0764	-0.0453	0.0178	0.0024	0.0030
rs11697820	C	0.2499	0.0451	0.0104	0.0106	-0.0020	0.0017
rs11719057	G	0.2714	0.0467	0.0101	0.0111	-0.0014	0.0018
rs11721038	T	0.4978	0.0841	0.0381	0.0171	-0.0024	0.0032
rs1173771	A	-0.6369	0.0450	-0.0048	0.0105	0.0000	0.0017
rs11774829	T	0.4089	0.0739	0.0116	0.0196	0.0029	0.0028
rs11795365	G	-0.3642	0.0642	0.0282	0.0145	-0.0015	0.0025
rs11821781	A	-0.3392	0.0507	-0.0104	0.0120	0.0026	0.0020
rs11874	G	-0.6251	0.0642	-0.0494	0.0171	0.0068	0.0025
rs12035750	T	-0.3499	0.0454	-0.0035	0.0112	0.0085	0.0018
rs12055028	G	-0.3149	0.0479	0.0049	0.0108	0.0000	0.0018
rs12147852	G	-0.3294	0.0516	-0.0116	0.0123	-0.0012	0.0020
rs12258967	C	0.6150	0.0484	0.0294	0.0122	0.0011	0.0019
rs1229984	T	-1.0787	0.1483	-0.0308	0.0208	0.0229	0.0057
rs1250258	C	0.3174	0.0501	0.0306	0.0116	0.0021	0.0019
rs12509892	T	-0.3709	0.0443	-0.0320	0.0103	0.0037	0.0017

rs12543884	A	-0.3423	0.0579	-0.0153	0.0124	-0.0027	0.0022
rs12544711	G	0.2944	0.0449	-0.0002	0.0104	-0.0002	0.0017
rs12567136	C	0.7688	0.0595	0.0182	0.0141	0.0015	0.0023
rs12608469	T	-0.5593	0.0935	0.0490	0.0239	-0.0057	0.0036
rs1262894	A	0.4733	0.0836	-0.0187	0.0214	-0.0053	0.0034
rs1263146	G	0.2574	0.0444	-0.0056	0.0102	-0.0045	0.0017
rs12643599	A	0.3161	0.0459	0.0531	0.0105	-0.0011	0.0018
rs12699561	G	-0.2481	0.0456	-0.0253	0.0110	0.0055	0.0018
rs12705390	G	-0.7711	0.0555	0.0028	0.0123	0.0051	0.0021
rs12822344	C	-0.2893	0.0448	-0.0098	0.0106	0.0005	0.0017
rs12906962	T	-0.2770	0.0477	-0.0240	0.0115	-0.0041	0.0018
rs1290784	C	-0.3710	0.0446	-0.0073	0.0104	-0.0011	0.0017
rs13107325	C	0.6251	0.0841	0.0015	0.0244	0.0188	0.0034
rs13129779	C	0.2546	0.0446	0.0168	0.0104	-0.0004	0.0017
rs13219548	C	-0.4362	0.0445	-0.0044	0.0102	0.0009	0.0017
rs13436194	A	0.3628	0.0446	0.0287	0.0102	0.0039	0.0017
rs137923903	C	1.2912	0.1937	0.0681	0.0487	0.0046	0.0078
rs1397985	G	0.3098	0.0473	0.0008	0.0110	0.0015	0.0018
rs1422279	C	-0.3138	0.0456	-0.0150	0.0106	-0.0045	0.0018
rs1436138	A	0.3017	0.0463	-0.0086	0.0110	0.0029	0.0018
rs145339349	G	-1.1270	0.1676	-0.0589	0.0480	-0.0010	0.0065
rs1458038	C	-0.7179	0.0487	-0.0467	0.0113	-0.0028	0.0019
rs149932962	G	-0.3024	0.0511	0.0287	0.0136	0.0014	0.0020
rs150857355	G	-0.8776	0.1531	-0.0799	0.0504	-0.0029	0.0060
rs151157965	G	-0.4568	0.0751	-0.0109	0.0181	0.0059	0.0029
rs1530558	T	0.3654	0.0643	-0.0206	0.0161	-0.0035	0.0027
rs1542977	G	-0.2805	0.0443	-0.0279	0.0104	0.0065	0.0017
rs1543270	C	0.3593	0.0445	-0.0139	0.0104	-0.0028	0.0017
rs1543927	T	0.4427	0.0502	0.0348	0.0113	-0.0087	0.0019
rs167479	G	0.5274	0.0442	0.0128	0.0158	0.0009	0.0020
rs17011002	C	-0.5018	0.0638	0.0023	0.0146	0.0041	0.0025
rs17173238	A	-0.2918	0.0489	-0.0091	0.0125	-0.0018	0.0019
rs1757915	G	-0.3347	0.0461	-0.0107	0.0106	-0.0007	0.0018
rs17677603	A	-0.3326	0.0454	-0.0240	0.0106	-0.0027	0.0018
rs1779240	G	0.3653	0.0521	0.0217	0.0118	-0.0032	0.0020
rs1887320	G	-0.3471	0.0444	-0.0115	0.0102	0.0020	0.0017
rs2017199	G	0.3274	0.0485	-0.0043	0.0117	-0.0007	0.0019
rs2052692	A	0.2673	0.0475	-0.0123	0.0121	0.0064	0.0018
rs2062316	A	-0.3519	0.0445	-0.0120	0.0110	0.0030	0.0017
rs2111834	G	0.4380	0.0684	-0.0009	0.0180	-0.0107	0.0027
rs2129561	A	-0.3354	0.0451	-0.0146	0.0107	0.0004	0.0017
rs2177843	C	-0.4184	0.0628	-0.0073	0.0140	0.0024	0.0024
rs2237306	C	0.3706	0.0642	0.0096	0.0143	-0.0205	0.0024
rs2249105	A	0.3532	0.0458	-0.0006	0.0105	-0.0039	0.0018
rs2285815	C	0.2952	0.0500	0.0230	0.0112	-0.0039	0.0019
rs2288276	G	-0.4101	0.0729	-0.0116	0.0151	-0.0017	0.0028

rs2301597	T	0.4335	0.0448	0.0010	0.0103	-0.0052	0.0017
rs2303083	G	0.4692	0.0559	0.0139	0.0138	-0.0105	0.0022
rs2306363	G	0.4059	0.0546	0.0421	0.0129	-0.0052	0.0022
rs231708	G	0.2696	0.0476	0.0109	0.0111	0.0010	0.0018
rs2423514	A	0.3053	0.0443	0.0251	0.0104	0.0005	0.0017
rs2469133	G	-0.2652	0.0466	-0.0373	0.0109	-0.0036	0.0018
rs2469997	G	0.3808	0.0570	-0.0035	0.0140	0.0011	0.0022
rs2493296	C	-0.4144	0.0642	-0.0596	0.0162	-0.0003	0.0025
rs2499801	G	0.3601	0.0569	-0.0526	0.0134	-0.0012	0.0022
rs2606738	T	-0.3227	0.0470	-0.0061	0.0105	0.0065	0.0018
rs2610989	T	-0.3378	0.0503	-0.0374	0.0125	0.0077	0.0019
rs2613203	A	-0.3618	0.0569	-0.0191	0.0131	-0.0040	0.0022
rs263017	A	0.2394	0.0444	0.0085	0.0102	-0.0032	0.0017
rs2643826	C	-0.3654	0.0445	-0.0006	0.0106	0.0014	0.0017
rs2681492	T	0.7161	0.0589	-0.0715	0.0125	0.0021	0.0023
rs268263	T	-0.5684	0.0517	-0.0408	0.0117	-0.0050	0.0020
rs2764834	T	-0.3148	0.0539	-0.0020	0.0120	0.0011	0.0021
rs2782652	T	-0.2821	0.0443	-0.0184	0.0108	-0.0121	0.0017
rs2782980	T	-0.3734	0.0492	-0.0218	0.0118	-0.0027	0.0019
rs2867114	C	0.4161	0.0759	0.0213	0.0139	-0.0053	0.0025
rs2897515	T	0.2629	0.0445	0.0124	0.0103	0.0031	0.0017
rs2978456	T	-0.2517	0.0443	0.0047	0.0113	0.0066	0.0018
rs3101442	T	-0.3048	0.0443	-0.0181	0.0105	0.0017	0.0017
rs3211995	G	0.4331	0.0607	-0.0074	0.0158	-0.0090	0.0024
rs34991912	T	0.2671	0.0449	0.0466	0.0104	-0.0003	0.0017
rs35021474	C	0.4695	0.0455	-0.0134	0.0115	-0.0067	0.0018
rs35443	G	0.3896	0.0454	0.0008	0.0107	0.0009	0.0018
rs35726503	A	0.3459	0.0447	-0.0022	0.0109	-0.0041	0.0017
rs35807464	G	-0.5299	0.0889	-0.0309	0.0208	-0.0087	0.0032
rs3786516	T	-0.3211	0.0538	0.0184	0.0121	-0.0023	0.0020
rs3790604	C	-0.7446	0.0846	-0.0184	0.0182	0.0033	0.0032
rs3826537	A	-0.2872	0.0447	0.0144	0.0111	0.0018	0.0017
rs3827750	C	-0.5341	0.0705	-0.0408	0.0145	-0.0052	0.0027
rs3887266	C	-0.3884	0.0694	-0.0282	0.0156	-0.0007	0.0027
rs4141663	C	0.2757	0.0449	0.0069	0.0104	0.0018	0.0017
rs42038	C	0.3773	0.0484	0.0234	0.0122	0.0011	0.0019
rs4246616	C	-0.2718	0.0445	-0.0056	0.0105	-0.0041	0.0017
rs434578	C	0.3810	0.0634	0.0292	0.0153	-0.0012	0.0025
rs4480845	T	0.4017	0.0463	-0.0164	0.0108	-0.0006	0.0018
rs4648815	G	0.2905	0.0447	0.0030	0.0123	-0.0074	0.0018
rs4666493	G	-0.3577	0.0448	-0.0107	0.0104	-0.0007	0.0017
rs4690775	A	0.2629	0.0451	0.0233	0.0105	0.0031	0.0017
rs4937515	G	0.3455	0.0453	-0.0018	0.0113	-0.0046	0.0017
rs55924432	C	-0.2770	0.0451	-0.0055	0.0112	-0.0023	0.0018
rs56352102	C	-0.5273	0.0570	-0.0187	0.0132	-0.0004	0.0022
rs57139556	A	0.5824	0.0857	0.0526	0.0174	-0.0026	0.0033

rs57301765	G	-0.5131	0.0608	-0.0412	0.0126	0.0052	0.0023
rs597808	A	0.4025	0.0443	0.0699	0.0118	-0.0024	0.0017
rs604723	T	-0.6293	0.0497	-0.0288	0.0112	0.0049	0.0019
rs6062625	T	-0.3285	0.0512	-0.0355	0.0132	0.0035	0.0020
rs61148001	C	0.3262	0.0546	0.0144	0.0120	0.0000	0.0021
rs62162674	G	0.2691	0.0445	0.0014	0.0103	0.0006	0.0017
rs62229372	C	-0.3966	0.0672	0.0046	0.0192	0.0037	0.0026
rs6453400	G	0.2515	0.0445	0.0149	0.0107	-0.0035	0.0017
rs6461992	A	-0.7171	0.0853	0.0095	0.0197	0.0014	0.0032
rs6704991	A	-0.2749	0.0461	0.0017	0.0107	0.0019	0.0018
rs6708660	T	0.2771	0.0452	-0.0090	0.0106	-0.0002	0.0017
rs6775384	G	-0.2988	0.0441	-0.0281	0.0104	0.0249	0.0017
rs6787069	C	-0.3038	0.0496	0.0187	0.0114	0.0085	0.0019
rs6815273	G	0.2850	0.0450	0.0001	0.0112	0.0002	0.0017
rs6963105	A	-0.2766	0.0446	-0.0123	0.0121	0.0092	0.0018
rs7019061	C	0.2794	0.0489	0.0069	0.0111	-0.0031	0.0019
rs7070797	G	0.5992	0.0635	0.0233	0.0169	-0.0046	0.0025
rs7107356	A	-0.4367	0.0441	-0.0109	0.0104	0.0055	0.0017
rs7123754	T	0.3299	0.0472	0.0157	0.0108	-0.0032	0.0018
rs7125196	T	0.4486	0.0693	0.0287	0.0143	0.0106	0.0026
rs71313932	G	-0.2825	0.0491	-0.0308	0.0117	-0.0010	0.0019
rs7200432	G	0.3351	0.0484	0.0168	0.0112	0.0025	0.0019
rs72683923	T	1.0062	0.1588	0.0812	0.0397	-0.0110	0.0062
rs72792829	C	0.3598	0.0534	0.0253	0.0116	-0.0010	0.0021
rs72812818	G	0.2708	0.0482	0.0082	0.0121	-0.0009	0.0018
rs72843959	C	0.2788	0.0471	-0.0234	0.0315	0.0031	0.0018
rs7302981	A	0.3398	0.0457	0.0036	0.0111	-0.0007	0.0018
rs73046792	G	0.4241	0.0595	0.0067	0.0180	0.0001	0.0024
rs73050466	G	0.8133	0.1316	0.0107	0.0394	-0.0064	0.0048
rs7306710	T	-0.2759	0.0445	-0.0223	0.0108	0.0082	0.0017
rs73075659	A	0.3378	0.0467	0.0130	0.0116	0.0026	0.0018
rs7315688	G	0.2693	0.0484	0.0103	0.0115	-0.0024	0.0019
rs73563812	G	0.3946	0.0521	0.0115	0.0119	0.0065	0.0020
rs7439366	T	-0.2487	0.0443	-0.0004	0.0113	0.0005	0.0017
rs7459106	A	0.3574	0.0562	0.0345	0.0130	-0.0045	0.0022
rs75271047	G	0.4691	0.0800	0.0350	0.0185	-0.0057	0.0031
rs7532726	A	0.2910	0.0500	0.0214	0.0117	-0.0002	0.0019
rs7553325	C	-0.2516	0.0443	0.0179	0.0107	0.0000	0.0017
rs75777337	T	-0.6027	0.0724	-0.0433	0.0174	0.0026	0.0027
rs75989961	T	-0.4855	0.0807	-0.0405	0.0236	0.0027	0.0032
rs77870048	C	-0.9363	0.0989	-0.0007	0.0339	-0.0063	0.0040
rs79349366	C	0.7946	0.1255	-0.0311	0.0388	0.0157	0.0048
rs7938342	T	-0.5398	0.0449	0.0004	0.0116	0.0015	0.0018
rs7963801	T	-0.2501	0.0448	-0.0309	0.0113	0.0107	0.0017
rs8027450	C	-0.5420	0.0474	-0.0602	0.0122	0.0012	0.0018
rs8076588	C	0.2841	0.0443	0.0129	0.0105	0.0050	0.0017

rs861585	G	-0.2584	0.0445	-0.0046	0.0104	0.0067	0.0017
rs891511	G	0.3979	0.0479	0.0175	0.0123	-0.0042	0.0019
rs9294987	T	-0.2568	0.0445	0.0146	0.0111	-0.0012	0.0017
rs9373523	T	0.2556	0.0450	-0.0131	0.0104	-0.0022	0.0017
rs940743	T	-0.2682	0.0444	-0.0340	0.0108	-0.0002	0.0017
rs9472040	A	-0.2797	0.0473	-0.0164	0.0106	-0.0006	0.0018
rs9773022	T	-0.2937	0.0443	0.0044	0.0120	-0.0008	0.0018
rs9907379	T	-0.4026	0.0542	-0.0229	0.0123	-0.0009	0.0021
rs9993149	T	0.2797	0.0442	-0.0017	0.0102	-0.0053	0.0017

Supplementary Table 15: Genetic association estimates for the two-sample regression-based MVMR analysis estimating the effect of SBP on stroke, adjusted for education. ea=effect allele; gx=SBP; gy=stroke; gz=education; se=standard error

SNP	ea	gx	gx_se	gy	gy_se	gz	gz_se
rs10024506	G	0.3036	0.0521	0.0048	0.0090	0.0004	0.0020
rs10048404	C	0.2683	0.0458	0.0130	0.0088	-0.0055	0.0018
rs1010064	A	0.4007	0.0571	-0.0100	0.0096	-0.0015	0.0022
rs10195405	A	0.2918	0.0486	0.0053	0.0087	-0.0035	0.0019
rs10212058	A	0.3278	0.0558	0.0045	0.0103	-0.0003	0.0022
rs1051447	C	0.2715	0.0489	0.0267	0.0088	0.0003	0.0019
rs10775143	C	0.2586	0.0453	-0.0040	0.0079	0.0075	0.0017
rs1077795	A	0.2939	0.0503	0.0310	0.0102	0.0000	0.0019
rs10817007	T	-0.4373	0.0661	0.0088	0.0108	0.0045	0.0026
rs10883543	G	-0.6091	0.0705	0.0279	0.0144	0.0000	0.0027
rs10977670	A	0.3011	0.0463	-0.0016	0.0082	0.0012	0.0018
rs10995311	C	0.3440	0.0446	-0.0038	0.0086	0.0059	0.0017
rs11070245	T	-0.2798	0.0444	0.0061	0.0079	-0.0052	0.0017
rs11145807	A	0.2734	0.0453	0.0168	0.0093	0.0017	0.0018
rs11146456	A	-0.4230	0.0695	-0.0037	0.0155	0.0026	0.0027
rs11187838	G	0.4660	0.0446	0.0166	0.0078	0.0028	0.0017
rs11191580	T	0.9647	0.0829	0.0023	0.0127	0.0022	0.0031
rs113044050	C	0.3637	0.0641	-0.0004	0.0118	-0.0046	0.0024
rs113523959	G	0.3852	0.0627	0.0234	0.0142	-0.0046	0.0025
rs113605702	G	0.4460	0.0798	0.0096	0.0142	-0.0134	0.0031
rs115262049	A	0.4875	0.0780	0.0043	0.0172	-0.0032	0.0030
rs11605215	G	-0.3191	0.0559	0.0003	0.0096	-0.0014	0.0021
rs11616710	C	-0.5056	0.0778	-0.0360	0.0165	0.0028	0.0029
rs11641308	T	-0.3272	0.0470	-0.0083	0.0085	0.0008	0.0018
rs11667829	G	-0.2636	0.0472	-0.0048	0.0098	0.0046	0.0018
rs116734066	C	0.4365	0.0764	-0.0196	0.0157	0.0024	0.0030
rs11697820	C	0.2499	0.0451	0.0053	0.0082	-0.0020	0.0017
rs11719057	G	0.2714	0.0467	0.0168	0.0089	-0.0014	0.0018
rs11721038	T	0.4978	0.0841	0.0224	0.0131	-0.0024	0.0032
rs1173771	A	-0.6369	0.0450	-0.0291	0.0081	0.0000	0.0017
rs11774829	T	0.4089	0.0739	0.0289	0.0144	0.0029	0.0028
rs11795365	G	-0.3642	0.0642	-0.0323	0.0108	-0.0015	0.0025
rs11821781	A	-0.3392	0.0507	0.0101	0.0093	0.0026	0.0020
rs11874	G	-0.6251	0.0642	-0.0502	0.0137	0.0068	0.0025
rs12035750	T	-0.3499	0.0454	-0.0154	0.0084	0.0085	0.0018
rs12055028	G	-0.3149	0.0479	0.0010	0.0085	0.0000	0.0018
rs12147852	G	-0.3294	0.0516	0.0128	0.0094	-0.0012	0.0020
rs12258967	C	0.6150	0.0484	0.0256	0.0098	0.0011	0.0019
rs1229984	T	-1.0787	0.1483	-0.0429	0.0174	0.0229	0.0057
rs1250258	C	0.3174	0.0501	0.0251	0.0094	0.0021	0.0019
rs12509892	T	-0.3709	0.0443	-0.0074	0.0082	0.0037	0.0017

rs12543884	A	-0.3423	0.0579	0.0012	0.0097	-0.0027	0.0022
rs12544711	G	0.2944	0.0449	-0.0157	0.0081	-0.0002	0.0017
rs12567136	C	0.7688	0.0595	0.0203	0.0109	0.0015	0.0023
rs12608469	T	-0.5593	0.0935	-0.0088	0.0167	-0.0057	0.0036
rs1262894	A	0.4733	0.0836	0.0165	0.0153	-0.0053	0.0034
rs1263146	G	0.2574	0.0444	0.0045	0.0079	-0.0045	0.0017
rs12643599	A	0.3161	0.0459	0.0185	0.0081	-0.0011	0.0018
rs12699561	G	-0.2481	0.0456	-0.0197	0.0081	0.0055	0.0018
rs12705390	G	-0.7711	0.0555	-0.0323	0.0107	0.0051	0.0021
rs12822344	C	-0.2893	0.0448	-0.0021	0.0081	0.0005	0.0017
rs12906962	T	-0.2770	0.0477	-0.0017	0.0086	-0.0041	0.0018
rs1290784	C	-0.3710	0.0446	-0.0161	0.0083	-0.0011	0.0017
rs13107325	C	0.6251	0.0841	0.0005	0.0193	0.0188	0.0034
rs13129779	C	0.2546	0.0446	-0.0006	0.0082	-0.0004	0.0017
rs13219548	C	-0.4362	0.0445	-0.0266	0.0079	0.0009	0.0017
rs13436194	A	0.3628	0.0446	0.0066	0.0081	0.0039	0.0017
rs1397985	G	0.3098	0.0473	0.0060	0.0084	0.0015	0.0018
rs1422279	C	-0.3138	0.0456	-0.0181	0.0080	-0.0045	0.0018
rs1436138	A	0.3017	0.0463	0.0036	0.0083	0.0029	0.0018
rs145339349	G	-1.1270	0.1676	-0.0159	0.0393	-0.0010	0.0065
rs1458038	C	-0.7179	0.0487	-0.0261	0.0087	-0.0028	0.0019
rs149932962	G	-0.3024	0.0511	0.0031	0.0104	0.0014	0.0020
rs150857355	G	-0.8776	0.1531	-0.0029	0.0384	-0.0029	0.0060
rs151157965	G	-0.4568	0.0751	0.0157	0.0133	0.0059	0.0029
rs1530558	T	0.3654	0.0643	-0.0020	0.0131	-0.0035	0.0027
rs1542977	G	-0.2805	0.0443	-0.0061	0.0079	0.0065	0.0017
rs1543270	C	0.3593	0.0445	0.0189	0.0077	-0.0028	0.0017
rs1543927	T	0.4427	0.0502	0.0160	0.0086	-0.0087	0.0019
rs167479	G	0.5274	0.0442	0.0214	0.0116	0.0009	0.0020
rs17011002	C	-0.5018	0.0638	-0.0068	0.0110	0.0041	0.0025
rs17173238	A	-0.2918	0.0489	-0.0132	0.0095	-0.0018	0.0019
rs1757915	G	-0.3347	0.0461	-0.0183	0.0084	-0.0007	0.0018
rs17677603	A	-0.3326	0.0454	-0.0275	0.0081	-0.0027	0.0018
rs1779240	G	0.3653	0.0521	0.0109	0.0094	-0.0032	0.0020
rs1887320	G	-0.3471	0.0444	-0.0269	0.0080	0.0020	0.0017
rs2017199	G	0.3274	0.0485	0.0005	0.0095	-0.0007	0.0019
rs2052692	A	0.2673	0.0475	-0.0298	0.0091	0.0064	0.0018
rs2062316	A	-0.3519	0.0445	-0.0299	0.0086	0.0030	0.0017
rs2111834	G	0.4380	0.0684	0.0198	0.0140	-0.0107	0.0027
rs2129561	A	-0.3354	0.0451	-0.0221	0.0082	0.0004	0.0017
rs2177843	C	-0.4184	0.0628	0.0042	0.0105	0.0024	0.0024
rs2237306	C	0.3706	0.0642	0.0106	0.0100	-0.0205	0.0024
rs2249105	A	0.3532	0.0458	0.0201	0.0081	-0.0039	0.0018
rs2285815	C	0.2952	0.0500	-0.0088	0.0087	-0.0039	0.0019
rs2288276	G	-0.4101	0.0729	0.0073	0.0115	-0.0017	0.0028
rs2301597	T	0.4335	0.0448	-0.0086	0.0081	-0.0052	0.0017

rs2303083	G	0.4692	0.0559	0.0021	0.0108	-0.0105	0.0022
rs2306363	G	0.4059	0.0546	0.0274	0.0098	-0.0052	0.0022
rs231708	G	0.2696	0.0476	0.0246	0.0086	0.0010	0.0018
rs2423514	A	0.3053	0.0443	0.0032	0.0084	0.0005	0.0017
rs2469133	G	-0.2652	0.0466	-0.0173	0.0081	-0.0036	0.0018
rs2469997	G	0.3808	0.0570	-0.0060	0.0110	0.0011	0.0022
rs2493296	C	-0.4144	0.0642	-0.0425	0.0124	-0.0003	0.0025
rs2499801	G	0.3601	0.0569	0.0074	0.0103	-0.0012	0.0022
rs2606738	T	-0.3227	0.0470	-0.0081	0.0081	0.0065	0.0018
rs2610989	T	-0.3378	0.0503	-0.0315	0.0091	0.0077	0.0019
rs2613203	A	-0.3618	0.0569	0.0139	0.0102	-0.0040	0.0022
rs263017	A	0.2394	0.0444	0.0037	0.0079	-0.0032	0.0017
rs2643826	C	-0.3654	0.0445	-0.0250	0.0083	0.0014	0.0017
rs2681492	T	0.7161	0.0589	0.0361	0.0099	0.0021	0.0023
rs268263	T	-0.5684	0.0517	-0.0027	0.0090	-0.0050	0.0020
rs2764834	T	-0.3148	0.0539	-0.0141	0.0092	0.0011	0.0021
rs2782652	T	-0.2821	0.0443	-0.0014	0.0082	-0.0121	0.0017
rs2782980	T	-0.3734	0.0492	0.0016	0.0089	-0.0027	0.0019
rs2867114	C	0.4161	0.0759	0.0020	0.0115	-0.0053	0.0025
rs2897515	T	0.2629	0.0445	0.0109	0.0079	0.0031	0.0017
rs2978456	T	-0.2517	0.0443	0.0036	0.0084	0.0066	0.0018
rs3101442	T	-0.3048	0.0443	-0.0065	0.0080	0.0017	0.0017
rs3211995	G	0.4331	0.0607	0.0082	0.0119	-0.0090	0.0024
rs34991912	T	0.2671	0.0449	0.0153	0.0081	-0.0003	0.0017
rs35443	G	0.3896	0.0454	0.0455	0.0083	0.0009	0.0018
rs35726503	A	0.3459	0.0447	-0.0003	0.0084	-0.0041	0.0017
rs35807464	G	-0.5299	0.0889	-0.0050	0.0151	-0.0087	0.0032
rs3786516	T	-0.3211	0.0538	0.0094	0.0098	-0.0023	0.0020
rs3790604	C	-0.7446	0.0846	-0.0671	0.0129	0.0033	0.0032
rs3826537	A	-0.2872	0.0447	-0.0034	0.0080	0.0018	0.0017
rs3827750	C	-0.5341	0.0705	-0.0319	0.0115	-0.0052	0.0027
rs3887266	C	-0.3884	0.0694	-0.0384	0.0133	-0.0007	0.0027
rs4141663	C	0.2757	0.0449	0.0173	0.0079	0.0018	0.0017
rs42038	C	0.3773	0.0484	0.0508	0.0095	0.0011	0.0019
rs4246616	C	-0.2718	0.0445	-0.0140	0.0083	-0.0041	0.0017
rs434578	C	0.3810	0.0634	0.0176	0.0127	-0.0012	0.0025
rs4480845	T	0.4017	0.0463	0.0002	0.0084	-0.0006	0.0018
rs4648815	G	0.2905	0.0447	0.0050	0.0090	-0.0074	0.0018
rs4666493	G	-0.3577	0.0448	0.0038	0.0082	-0.0007	0.0017
rs4690775	A	0.2629	0.0451	0.0257	0.0083	0.0031	0.0017
rs4937515	G	0.3455	0.0453	-0.0095	0.0089	-0.0046	0.0017
rs55924432	C	-0.2770	0.0451	-0.0339	0.0084	-0.0023	0.0018
rs56352102	C	-0.5273	0.0570	-0.0135	0.0105	-0.0004	0.0022
rs57139556	A	0.5824	0.0857	0.0172	0.0140	-0.0026	0.0033
rs57301765	G	-0.5131	0.0608	-0.0669	0.0096	0.0052	0.0023
rs597808	A	0.4025	0.0443	0.0576	0.0093	-0.0024	0.0017

rs604723	T	-0.6293	0.0497	-0.0158	0.0083	0.0049	0.0019
rs6062625	T	-0.3285	0.0512	-0.0117	0.0101	0.0035	0.0020
rs61148001	C	0.3262	0.0546	0.0024	0.0090	0.0000	0.0021
rs62162674	G	0.2691	0.0445	0.0249	0.0082	0.0006	0.0017
rs62229372	C	-0.3966	0.0672	-0.0182	0.0135	0.0037	0.0026
rs6453400	G	0.2515	0.0445	0.0079	0.0080	-0.0035	0.0017
rs6461992	A	-0.7171	0.0853	-0.0315	0.0125	0.0014	0.0032
rs6704991	A	-0.2749	0.0461	-0.0090	0.0083	0.0019	0.0018
rs6708660	T	0.2771	0.0452	0.0265	0.0091	-0.0002	0.0017
rs6775384	G	-0.2988	0.0441	-0.0093	0.0087	0.0249	0.0017
rs6787069	C	-0.3038	0.0496	-0.0033	0.0092	0.0085	0.0019
rs6815273	G	0.2850	0.0450	0.0125	0.0086	0.0002	0.0017
rs6963105	A	-0.2766	0.0446	-0.0369	0.0096	0.0092	0.0018
rs7019061	C	0.2794	0.0489	-0.0003	0.0086	-0.0031	0.0019
rs7070797	G	0.5992	0.0635	0.0315	0.0132	-0.0046	0.0025
rs7107356	A	-0.4367	0.0441	0.0154	0.0079	0.0055	0.0017
rs7123754	T	0.3299	0.0472	0.0156	0.0081	-0.0032	0.0018
rs7125196	T	0.4486	0.0693	0.0240	0.0108	0.0106	0.0026
rs71313932	G	-0.2825	0.0491	-0.0139	0.0090	-0.0010	0.0019
rs7200432	G	0.3351	0.0484	0.0150	0.0089	0.0025	0.0019
rs72683923	T	1.0062	0.1588	0.0357	0.0380	-0.0110	0.0062
rs72792829	C	0.3598	0.0534	-0.0132	0.0091	-0.0010	0.0021
rs72812818	G	0.2708	0.0482	0.0145	0.0091	-0.0009	0.0018
rs72843959	C	0.2788	0.0471	0.0223	0.0097	0.0031	0.0018
rs7302981	A	0.3398	0.0457	-0.0011	0.0089	-0.0007	0.0018
rs73046792	G	0.4241	0.0595	0.0098	0.0131	0.0001	0.0024
rs73050466	G	0.8133	0.1316	-0.0124	0.0287	-0.0064	0.0048
rs7306710	T	-0.2759	0.0445	-0.0109	0.0084	0.0082	0.0017
rs73075659	A	0.3378	0.0467	0.0144	0.0093	0.0026	0.0018
rs7315688	G	0.2693	0.0484	0.0338	0.0086	-0.0024	0.0019
rs73563812	G	0.3946	0.0521	0.0069	0.0089	0.0065	0.0020
rs7439366	T	-0.2487	0.0443	-0.0134	0.0090	0.0005	0.0017
rs7459106	A	0.3574	0.0562	0.0107	0.0095	-0.0045	0.0022
rs75271047	G	0.4691	0.0800	0.0234	0.0151	-0.0057	0.0031
rs7532726	A	0.2910	0.0500	0.0035	0.0098	-0.0002	0.0019
rs7553325	C	-0.2516	0.0443	-0.0008	0.0086	0.0000	0.0017
rs75777337	T	-0.6027	0.0724	-0.0126	0.0136	0.0026	0.0027
rs75989961	T	-0.4855	0.0807	-0.0035	0.0178	0.0027	0.0032
rs79349366	C	0.7946	0.1255	0.0165	0.0276	0.0157	0.0048
rs7938342	T	-0.5398	0.0449	-0.0329	0.0084	0.0015	0.0018
rs7963801	T	-0.2501	0.0448	-0.0044	0.0088	0.0107	0.0017
rs8027450	C	-0.5420	0.0474	-0.0303	0.0092	0.0012	0.0018
rs8076588	C	0.2841	0.0443	0.0144	0.0079	0.0050	0.0017
rs861585	G	-0.2584	0.0445	-0.0150	0.0081	0.0067	0.0017
rs891511	G	0.3979	0.0479	0.0233	0.0089	-0.0042	0.0019
rs9294987	T	-0.2568	0.0445	0.0030	0.0086	-0.0012	0.0017

rs9373523	T	0.2556	0.0450	-0.0203	0.0081	-0.0022	0.0017
rs940743	T	-0.2682	0.0444	-0.0004	0.0092	-0.0002	0.0017
rs9472040	A	-0.2797	0.0473	-0.0449	0.0081	-0.0006	0.0018
rs9773022	T	-0.2937	0.0443	-0.0174	0.0088	-0.0008	0.0018
rs9907379	T	-0.4026	0.0542	-0.0257	0.0093	-0.0009	0.0021
rs9993149	T	0.2797	0.0442	0.0133	0.0078	-0.0053	0.0017

Supplementary Table 16: Genetic association estimates for the two-sample regression-based MVMR analysis estimating the effect of smoking on CAD, adjusted for education. ea=effect allele; gx=smoking; gy=CAD; gz=education; se=standard error

SNP	ea	gx	gx_se	gy	gy_se	gz	gz_se
rs10052591	T	0.0084	0.001402	0.006695	0.009413	0.00182	0.00172
rs10226228	A	-0.01141	0.001437	-0.01588	0.009475	0.01162	0.00177
rs10282292	C	0.008962	0.001447	0.022719	0.009558	-0.00096	0.00179
rs1050847	C	0.00797	0.001405	0.026152	0.010334	-0.01075	0.00172
rs10823968	A	0.008135	0.001453	0.010107	0.010483	-0.00402	0.00178
rs10879871	T	-0.00958	0.001458	-0.00214	0.009462	0.00842	0.00179
rs10918701	G	0.008024	0.001432	0.023604	0.009568	-0.00096	0.00176
rs10922907	A	0.010175	0.001395	0.005847	0.00941	-0.01609	0.00171
rs11210229	A	0.011702	0.001423	0.000119	0.00942	-0.00434	0.00174
rs112282219	G	-0.02317	0.003504	0.001997	0.027316	0.00172	0.00436
rs11255908	T	-0.01007	0.001588	0.010164	0.0102	0.005	0.00198
rs113382419	C	-0.02823	0.002214	-0.02074	0.018989	-0.00533	0.00278
rs11768481	C	0.009012	0.001475	0.014147	0.010438	-0.00096	0.0018
rs11783093	C	0.01571	0.001897	0.034962	0.013831	-0.00236	0.00232
rs11861214	G	0.009451	0.001685	-0.00221	0.011195	-0.01138	0.00204
rs1193237	G	-0.00776	0.001397	-0.01151	0.009381	0.00855	0.00171
rs11948770	T	-0.01024	0.001645	0.005828	0.011319	-0.00275	0.00201
rs12202536	A	-0.00823	0.001386	-0.00243	0.009279	0.00194	0.0017
rs1221148	C	0.009165	0.001407	-0.00083	0.009497	9.00E-05	0.00172
rs12244388	G	-0.01325	0.001464	0.036276	0.009434	0.00652	0.00183
rs1246265	T	-0.00887	0.001509	-0.00911	0.010145	0.00481	0.00185
rs12481282	G	-0.00894	0.001549	0.026601	0.010388	0.00269	0.0019
rs12623702	A	-0.00977	0.001428	0.008648	0.00991	0.00993	0.00174
rs12708665	A	-0.00909	0.001539	-0.01072	0.010694	0.00886	0.00187
rs12831617	C	-0.00918	0.001633	-0.02378	0.011454	0.00478	0.00204
rs12967855	A	0.008189	0.001479	-0.00956	0.009712	-0.01935	0.00181
rs13009008	A	0.008633	0.001473	0.003304	0.01013	-0.00946	0.00181
rs13016665	C	-0.00849	0.001412	-0.00515	0.009797	0.00124	0.00172
rs13153393	A	-0.01375	0.002173	-0.00377	0.014748	0.00549	0.00268
rs13296519	G	-0.0097	0.001419	-0.01951	0.009926	0.00775	0.00174
rs136233	A	-0.00996	0.001769	-0.03069	0.011943	0.00581	0.00217
rs147412694	G	-0.01157	0.001949	-0.01447	0.01413	-0.00077	0.00243
rs17309874	G	-0.01129	0.001582	-0.02463	0.011799	0.00825	0.00194
rs17553262	A	-0.01273	0.002181	0.004595	0.016538	0.01119	0.00272
rs17576594	G	0.01095	0.001552	-0.00125	0.010264	-0.01126	0.00189
rs1922018	C	0.010033	0.001438	-0.00645	0.010022	-0.00567	0.00176
rs1931263	G	-0.00761	0.001386	-0.01382	0.009799	0.01204	0.0017
rs1933270	T	0.009218	0.001438	0.005744	0.009421	-0.00164	0.00176
rs202645	A	-0.01016	0.001725	-0.00722	0.011255	-9.00E-05	0.00214
rs2062882	G	-0.00811	0.00142	-0.00065	0.009505	0.008	0.00173
rs2080870	A	0.008633	0.001583	-0.0146	0.01119	-0.01576	0.00195

rs2254710	C	0.008996	0.001631	0.019446	0.010469	-0.00306	0.00198
rs2401924	G	0.010572	0.001389	0.006553	0.009175	0.00441	0.0017
rs245774	A	-0.00902	0.00156	-0.01129	0.011245	0.00702	0.00192
rs2675638	G	0.008499	0.0014	0.006059	0.009277	-0.0068	0.00172
rs2678670	A	0.008731	0.001388	0.009729	0.010196	-0.00987	0.0017
rs2838834	C	-0.00936	0.001515	-0.02171	0.009846	-0.00091	0.00186
rs28485305	C	0.008007	0.001439	0.01123	0.009415	-0.00075	0.00176
rs2867112	T	0.014783	0.001887	0.041434	0.012524	-0.00484	0.00229
rs2890772	G	-0.0137	0.001407	0.003933	0.009736	-0.00355	0.00173
rs2894808	T	-0.0153	0.002591	-0.00452	0.016658	0.00761	0.00314
rs317021	T	-0.01157	0.001791	-0.00082	0.012704	0.00569	0.0022
rs326341	G	0.009435	0.001392	-0.0037	0.009363	-0.00627	0.0017
rs329120	C	0.009657	0.001405	-0.02007	0.009347	-0.00833	0.00172
rs34866095	A	-0.00857	0.001506	-0.01303	0.010581	0.0019	0.00184
rs348809	A	-0.00828	0.001456	-0.00689	0.009731	0.00498	0.00179
rs35169606	T	0.008776	0.001444	0.012571	0.011193	-0.00248	0.00177
rs35175834	G	-0.0164	0.001698	0.011184	0.012028	0.01292	0.00208
rs35343344	C	0.009181	0.001596	0.01511	0.011241	-0.00588	0.00196
rs359243	T	-0.00872	0.001425	-0.00278	0.009843	0.00644	0.00174
rs369230	G	-0.00909	0.001511	-0.02648	0.012423	0.0052	0.00193
rs3742365	T	-0.01079	0.001416	-0.01621	0.009498	-0.0059	0.00173
rs3769949	T	-0.00826	0.001386	-0.01452	0.00953	0.00495	0.0017
rs3811038	T	-0.00954	0.001557	0.004533	0.010968	0.0066	0.0019
rs3896224	A	0.009627	0.001418	0.012017	0.009282	-0.0088	0.00173
rs421983	T	0.008707	0.001386	0.00063	0.009154	-0.00333	0.0017
rs4391802	A	0.010319	0.001528	0.004798	0.010222	-0.00716	0.00187
rs4473348	A	-0.01043	0.001597	-0.02015	0.011068	-0.00261	0.00197
rs4543592	T	-0.00866	0.001389	-0.00666	0.009601	0.00666	0.0017
rs4571506	C	0.007877	0.001392	0.015673	0.009403	-0.01154	0.00171
rs4671357	T	-0.00944	0.00139	-0.00826	0.009453	0.00913	0.0017
rs4731925	C	-0.00829	0.00149	-0.01431	0.009823	0.01057	0.00184
rs4814873	C	0.009712	0.001636	0.021137	0.010907	-0.00643	0.00203
rs4949465	T	-0.01161	0.002058	-0.02287	0.012214	0.01288	0.0025
rs4957528	A	-0.01012	0.001722	-0.01456	0.012746	0.00507	0.00212
rs549845	G	0.011261	0.001509	0.008157	0.009828	-0.01772	0.00186
rs57611503	G	0.007743	0.00141	0.00609	0.010376	-0.0033	0.00172
rs6011779	C	0.019115	0.001764	0.018733	0.013546	-0.00422	0.00223
rs60952428	T	0.013411	0.002419	0.014414	0.013882	-0.00787	0.00306
rs6119897	G	-0.0128	0.001627	-0.02016	0.010517	0.00691	0.00196
rs61796681	A	-0.01342	0.002448	-0.00117	0.017974	0.00053	0.00301
rs62098013	G	-0.00857	0.001457	-0.00806	0.010666	0.01256	0.00176
rs62135536	C	0.024344	0.003962	0.003779	0.029824	-0.00924	0.00487
rs62155874	A	-0.01691	0.002085	0.017265	0.01539	0.01416	0.00258
rs62175972	T	0.021746	0.003857	0.030329	0.030435	-0.02995	0.00475
rs624833	T	0.009286	0.001504	0.010431	0.010679	-0.01316	0.00184
rs6562474	C	0.008369	0.001461	0.005401	0.009565	-0.00427	0.00178

rs6598539	T	-0.00815	0.001389	-0.01495	0.009226	0.00651	0.00171
rs6741228	T	0.007933	0.001404	0.020156	0.009341	-0.00747	0.00172
rs67596067	G	-0.00888	0.001458	-0.01707	0.009959	0.00355	0.00178
rs6778080	T	0.011114	0.001566	0.0106	0.011441	-0.01385	0.00192
rs6779302	G	-0.00875	0.001439	0.002586	0.009792	0.01129	0.00176
rs6935954	A	0.009582	0.001402	-0.0109	0.009892	-0.01376	0.00172
rs6957896	C	-0.00758	0.001387	-0.00727	0.009552	0.00164	0.0017
rs6962772	A	0.011064	0.001916	0.008701	0.011447	-0.00404	0.00234
rs7039819	G	0.008734	0.001405	0.014408	0.009487	-0.01059	0.00172
rs7077678	C	0.00855	0.001436	0.011614	0.009336	0.00163	0.00174
rs71367545	G	-0.01032	0.001704	0.003924	0.012202	0.01246	0.0021
rs7155595	A	-0.00885	0.001485	-0.00862	0.010265	0.00734	0.00183
rs71627581	G	0.013257	0.002199	-0.00057	0.020734	-0.00962	0.00281
rs72674867	A	0.008988	0.001634	-0.01913	0.010288	-0.00674	0.00197
rs72678864	G	0.012383	0.001839	0.010335	0.014001	-0.01227	0.0023
rs7297175	T	-0.00812	0.001399	0.004107	0.009831	0.01559	0.00173
rs732083	G	0.008348	0.001473	0.020676	0.009828	-0.00814	0.0018
rs73220544	A	-0.01082	0.001913	-0.00821	0.013311	0.00692	0.00237
rs7333559	G	0.01074	0.001707	0.00524	0.012033	-0.00597	0.00212
rs74086911	G	0.014802	0.002642	0.001896	0.01897	-0.01837	0.0034
rs7519626	C	0.00842	0.001479	-0.00421	0.009825	-0.00688	0.0018
rs7528604	G	0.009653	0.001401	0.010029	0.009311	-0.00594	0.00172
rs7553348	G	0.009634	0.001396	-0.00333	0.00943	-0.00042	0.00172
rs7569203	A	-0.01076	0.0015	0.000949	0.009843	0.00257	0.00181
rs75742406	G	0.009619	0.001585	0.008049	0.010592	-0.00101	0.00192
rs76608582	C	0.021632	0.00344	-0.03298	0.037949	-0.02798	0.00445
rs775758	A	0.008014	0.001403	0.003609	0.009305	-0.00303	0.00171
rs7766610	C	0.012584	0.001793	0.012158	0.010987	0.00078	0.00218
rs7807019	A	-0.01042	0.001391	-0.01895	0.009188	0.01055	0.0017
rs8042134	T	-0.00994	0.001401	0.013937	0.009705	0.00794	0.00173
rs8042849	C	0.019216	0.001462	-0.00389	0.010106	0.00018	0.00178
rs860326	C	0.008338	0.001402	0.007768	0.009569	-0.00876	0.00172
rs8614	C	-0.01146	0.001797	-0.02126	0.011673	0.00407	0.00224
rs889398	C	0.009241	0.001414	0.026764	0.009708	-0.00182	0.00172
rs9435340	T	0.008348	0.001464	0.011075	0.009728	-0.00626	0.0018
rs9842947	C	-0.00877	0.001481	-0.00087	0.0099	0.00091	0.00182
rs986391	G	0.011139	0.001438	-0.001	0.009545	0.00161	0.00177

Supplementary Table 17: Genetic association estimates for the two-sample regression-based MVMR analysis estimating the effect of smoking on MI, adjusted for education. ea=effect allele; gx=smoking; gy=MI; gz=education; se=standard error

SNP	ea	gx	gx_se	gy	gy_se	gz	gz_se
rs10052591	T	0.0084	0.001402	0.002888	0.010404	0.00182	0.00172
rs10226228	A	-0.01141	0.001437	-0.01457	0.01063	0.01162	0.00177
rs10282292	C	0.008962	0.001447	0.015677	0.010653	-0.00096	0.00179
rs1050847	C	0.00797	0.001405	0.030359	0.011605	-0.01075	0.00172
rs10823968	A	0.008135	0.001453	-0.00486	0.011528	-0.00402	0.00178
rs10879871	T	-0.00958	0.001458	0.003259	0.010526	0.00842	0.00179
rs10918701	G	0.008024	0.001432	0.022638	0.010568	-0.00096	0.00176
rs10922907	A	0.010175	0.001395	0.010168	0.010502	-0.01609	0.00171
rs11210229	A	0.011702	0.001423	0.007425	0.010399	-0.00434	0.00174
rs112282219	G	-0.02317	0.003504	-0.00756	0.029899	0.00172	0.00436
rs11255908	T	-0.01007	0.001588	0.012542	0.011339	0.005	0.00198
rs113382419	C	-0.02823	0.002214	-0.01655	0.020816	-0.00533	0.00278
rs11768481	C	0.009012	0.001475	0.005505	0.011653	-0.00096	0.0018
rs11783093	C	0.01571	0.001897	0.025965	0.01513	-0.00236	0.00232
rs11861214	G	0.009451	0.001685	-0.0053	0.01252	-0.01138	0.00204
rs1193237	G	-0.00776	0.001397	-0.00879	0.010379	0.00855	0.00171
rs11948770	T	-0.01024	0.001645	0.001295	0.012656	-0.00275	0.00201
rs12202536	A	-0.00823	0.001386	0.003214	0.010225	0.00194	0.0017
rs1221148	C	0.009165	0.001407	0.001694	0.010595	9.00E-05	0.00172
rs12244388	G	-0.01325	0.001464	0.029727	0.01045	0.00652	0.00183
rs1246265	T	-0.00887	0.001509	-0.02026	0.01129	0.00481	0.00185
rs12481282	G	-0.00894	0.001549	0.022904	0.011549	0.00269	0.0019
rs12623702	A	-0.00977	0.001428	0.018979	0.010683	0.00993	0.00174
rs12708665	A	-0.00909	0.001539	-0.01855	0.011855	0.00886	0.00187
rs12831617	C	-0.00918	0.001633	-0.01416	0.01273	0.00478	0.00204
rs12967855	A	0.008189	0.001479	-0.01352	0.010726	-0.01935	0.00181
rs13009008	A	0.008633	0.001473	-0.00506	0.010939	-0.00946	0.00181
rs13016665	C	-0.00849	0.001412	-0.00289	0.010547	0.00124	0.00172
rs13153393	A	-0.01375	0.002173	0.008377	0.016227	0.00549	0.00268
rs13296519	G	-0.0097	0.001419	-0.02371	0.011082	0.00775	0.00174

rs136233	A	-0.00996	0.001769	-0.02782	0.01336	0.00581	0.00217
rs147412694	G	-0.01157	0.001949	-0.00648	0.015476	-0.00077	0.00243
rs17309874	G	-0.01129	0.001582	-0.01927	0.012987	0.00825	0.00194
rs17553262	A	-0.01273	0.002181	0.004333	0.018551	0.01119	0.00272
rs17576594	G	0.01095	0.001552	0.001002	0.011574	-0.01126	0.00189
rs1922018	C	0.010033	0.001438	-0.00122	0.011195	-0.00567	0.00176
rs1931263	G	-0.00761	0.001386	-0.0118	0.010742	0.01204	0.0017
rs1933270	T	0.009218	0.001438	0.001829	0.010426	-0.00164	0.00176
rs202645	A	-0.01016	0.001725	0.007252	0.012281	-9.00E-05	0.00214
rs2062882	G	-0.00811	0.00142	-0.00526	0.010499	0.008	0.00173
rs2080870	A	0.008633	0.001583	-0.0151	0.012274	-0.01576	0.00195
rs2254710	C	0.008996	0.001631	0.017441	0.011604	-0.00306	0.00198
rs2401924	G	0.010572	0.001389	0.007822	0.010183	0.00441	0.0017
rs245774	A	-0.00902	0.00156	-0.01828	0.012599	0.00702	0.00192
rs2675638	G	0.008499	0.0014	-0.0136	0.010362	-0.0068	0.00172
rs2678670	A	0.008731	0.001388	0.017725	0.010881	-0.00987	0.0017
rs2838834	C	-0.00936	0.001515	-0.01917	0.010974	-0.00091	0.00186
rs28485305	C	0.008007	0.001439	0.010152	0.010474	-0.00075	0.00176
rs2867112	T	0.014783	0.001887	0.021002	0.013766	-0.00484	0.00229
rs2890772	G	-0.0137	0.001407	-8.90E-05	0.010448	-0.00355	0.00173
rs2894808	T	-0.0153	0.002591	0.011612	0.018393	0.00761	0.00314
rs317021	T	-0.01157	0.001791	-0.00028	0.013927	0.00569	0.0022
rs326341	G	0.009435	0.001392	-0.00135	0.010444	-0.00627	0.0017
rs329120	C	0.009657	0.001405	-0.01014	0.010457	-0.00833	0.00172
rs34866095	A	-0.00857	0.001506	-0.01405	0.011558	0.0019	0.00184
rs348809	A	-0.00828	0.001456	0.001134	0.010787	0.00498	0.00179
rs35169606	T	0.008776	0.001444	0.010359	0.01226	-0.00248	0.00177
rs35175834	G	-0.0164	0.001698	0.00787	0.013336	0.01292	0.00208
rs35343344	C	0.009181	0.001596	0.011837	0.012454	-0.00588	0.00196
rs359243	T	-0.00872	0.001425	-0.00084	0.010491	0.00644	0.00174
rs369230	G	-0.00909	0.001511	-0.03158	0.013991	0.0052	0.00193
rs3742365	T	-0.01079	0.001416	-0.01225	0.010625	-0.0059	0.00173
rs3769949	T	-0.00826	0.001386	-0.00835	0.010232	0.00495	0.0017

rs3811038	T	-0.00954	0.001557	-0.0004	0.01177	0.0066	0.0019
rs3896224	A	0.009627	0.001418	0.017627	0.010271	-0.0088	0.00173
rs421983	T	0.008707	0.001386	-0.007	0.010203	-0.00333	0.0017
rs4391802	A	0.010319	0.001528	0.012032	0.011269	-0.00716	0.00187
rs4473348	A	-0.01043	0.001597	-0.02623	0.01187	-0.00261	0.00197
rs4543592	T	-0.00866	0.001389	-0.0039	0.010738	0.00666	0.0017
rs4571506	C	0.007877	0.001392	-0.00052	0.010298	-0.01154	0.00171
rs4671357	T	-0.00944	0.00139	-0.01323	0.01018	0.00913	0.0017
rs4731925	C	-0.00829	0.00149	-0.00911	0.011042	0.01057	0.00184
rs4814873	C	0.009712	0.001636	0.024003	0.012125	-0.00643	0.00203
rs4949465	T	-0.01161	0.002058	-0.02715	0.013472	0.01288	0.0025
rs4957528	A	-0.01012	0.001722	-0.00807	0.013959	0.00507	0.00212
rs549845	G	0.011261	0.001509	0.020238	0.01086	-0.01772	0.00186
rs57611503	G	0.007743	0.00141	0.00736	0.011476	-0.0033	0.00172
rs6011779	C	0.019115	0.001764	0.017484	0.015285	-0.00422	0.00223
rs60952428	T	0.013411	0.002419	0.016229	0.01554	-0.00787	0.00306
rs6119897	G	-0.0128	0.001627	-0.01595	0.011758	0.00691	0.00196
rs61796681	A	-0.01342	0.002448	-0.00199	0.019498	0.00053	0.00301
rs62098013	G	-0.00857	0.001457	-0.00688	0.011688	0.01256	0.00176
rs62135536	C	0.024344	0.003962	-0.00061	0.032802	-0.00924	0.00487
rs62155874	A	-0.01691	0.002085	0.016557	0.016566	0.01416	0.00258
rs62175972	T	0.021746	0.003857	0.010259	0.031785	-0.02995	0.00475
rs624833	T	0.009286	0.001504	0.019936	0.011763	-0.01316	0.00184
rs6562474	C	0.008369	0.001461	-0.00308	0.010605	-0.00427	0.00178
rs6598539	T	-0.00815	0.001389	-0.01902	0.010247	0.00651	0.00171
rs6741228	T	0.007933	0.001404	0.017175	0.010421	-0.00747	0.00172
rs67596067	G	-0.00888	0.001458	-0.00777	0.011022	0.00355	0.00178
rs6778080	T	0.011114	0.001566	0.009541	0.012778	-0.01385	0.00192
rs6779302	G	-0.00875	0.001439	0.002385	0.010831	0.01129	0.00176
rs6935954	A	0.009582	0.001402	-0.01734	0.010841	-0.01376	0.00172
rs6957896	C	-0.00758	0.001387	-0.01248	0.010778	0.00164	0.0017
rs6962772	A	0.011064	0.001916	0.008472	0.012975	-0.00404	0.00234

rs7039819	G	0.008734	0.001405	0.017474	0.010545	-0.01059	0.00172
rs7077678	C	0.00855	0.001436	0.019706	0.010338	0.00163	0.00174
rs71367545	G	-0.01032	0.001704	-0.01378	0.01344	0.01246	0.0021
rs7155595	A	-0.00885	0.001485	-0.01307	0.011338	0.00734	0.00183
rs71627581	G	0.013257	0.002199	-0.03481	0.022846	-0.00962	0.00281
rs72674867	A	0.008988	0.001634	-0.02439	0.011516	-0.00674	0.00197
rs72678864	G	0.012383	0.001839	0.007891	0.015289	-0.01227	0.0023
rs7297175	T	-0.00812	0.001399	-0.00404	0.011033	0.01559	0.00173
rs732083	G	0.008348	0.001473	0.018665	0.01091	-0.00814	0.0018
rs73220544	A	-0.01082	0.001913	-0.03053	0.014862	0.00692	0.00237
rs7333559	G	0.01074	0.001707	0.010289	0.013294	-0.00597	0.00212
rs74086911	G	0.014802	0.002642	-0.0037	0.021138	-0.01837	0.0034
rs7519626	C	0.00842	0.001479	-0.00407	0.010924	-0.00688	0.0018
rs7528604	G	0.009653	0.001401	0.006873	0.010295	-0.00594	0.00172
rs7553348	G	0.009634	0.001396	-0.00229	0.010475	-0.00042	0.00172
rs7569203	A	-0.01076	0.0015	-0.00116	0.010895	0.00257	0.00181
rs75742406	G	0.009619	0.001585	0.009601	0.011694	-0.00101	0.00192
rs76608582	C	0.021632	0.00344	-0.0376	0.042131	-0.02798	0.00445
rs775758	A	0.008014	0.001403	0.013796	0.010384	-0.00303	0.00171
rs7766610	C	0.012584	0.001793	0.014748	0.012369	0.00078	0.00218
rs7807019	A	-0.01042	0.001391	-0.02103	0.010215	0.01055	0.0017
rs8042134	T	-0.00994	0.001401	0.016923	0.010871	0.00794	0.00173
rs8042849	C	0.019216	0.001462	0.001274	0.011218	0.00018	0.00178
rs860326	C	0.008338	0.001402	-0.00041	0.010711	-0.00876	0.00172
rs8614	C	-0.01146	0.001797	-0.01818	0.012998	0.00407	0.00224
rs889398	C	0.009241	0.001414	0.018306	0.010877	-0.00182	0.00172
rs9435340	T	0.008348	0.001464	0.013712	0.010749	-0.00626	0.0018
rs9842947	C	-0.00877	0.001481	0.001092	0.010982	0.00091	0.00182
rs986391	G	0.011139	0.001438	0.003619	0.010535	0.00161	0.00177

Supplementary Table 18: Genetic association estimates for the two-sample regression-based MVMR analysis estimating the effect of smoking on stroke, adjusted for education. ea=effect allele; gx=smoking; gy=stroke; gz=education; se=standard error

SNP	ea	gx	gx_se	gy	gy_se	gz	gz_se
rs10052591	T	0.0084	0.001402	0.0108	0.0081	0.00182	0.00172
rs10226228	A	-0.01141	0.001437	-0.0055	0.008	0.01162	0.00177
rs10282292	C	0.008962	0.001447	0.0066	0.008	-0.00096	0.00179
rs1050847	C	0.00797	0.001405	0.0083	0.0091	-0.01075	0.00172
rs10823968	A	0.008135	0.001453	-0.0071	0.0091	-0.00402	0.00178
rs10879871	T	-0.00958	0.001458	-0.0047	0.0082	0.00842	0.00179
rs10918701	G	0.008024	0.001432	0.0012	0.0082	-0.00096	0.00176
rs10922907	A	0.010175	0.001395	0.0057	0.0078	-0.01609	0.00171
rs11210229	A	0.011702	0.001423	0.004	0.008	-0.00434	0.00174
rs112282219	G	-0.02317	0.003504	-0.0288	0.0236	0.00172	0.00436
rs11255908	T	-0.01007	0.001588	0.0025	0.009	0.005	0.00198
rs113382419	C	-0.02823	0.002214	-0.0028	0.0157	-0.00533	0.00278
rs11768481	C	0.009012	0.001475	8.00E-04	0.0099	-0.00096	0.0018
rs11783093	C	0.01571	0.001897	0.0017	0.0123	-0.00236	0.00232
rs11861214	G	0.009451	0.001685	-0.0093	0.0093	-0.01138	0.00204
rs1193237	G	-0.00776	0.001397	-9.00E-04	0.0083	0.00855	0.00171
rs11948770	T	-0.01024	0.001645	4.00E-04	0.0095	-0.00275	0.00201
rs12202536	A	-0.00823	0.001386	0.0053	0.0087	0.00194	0.0017
rs1221148	C	0.009165	0.001407	0.001	0.0078	9.00E-05	0.00172
rs12244388	G	-0.01325	0.001464	-0.0151	0.0079	0.00652	0.00183
rs1246265	T	-0.00887	0.001509	-0.0227	0.0087	0.00481	0.00185
rs12481282	G	-0.00894	0.001549	0.0061	0.0087	0.00269	0.0019
rs12623702	A	-0.00977	0.001428	0.0059	0.0082	0.00993	0.00174
rs12708665	A	-0.00909	0.001539	-0.0058	0.0093	0.00886	0.00187
rs12831617	C	-0.00918	0.001633	-0.0094	0.0108	0.00478	0.00204
rs12967855	A	0.008189	0.001479	-0.0056	0.0085	-0.01935	0.00181
rs13009008	A	0.008633	0.001473	0.0215	0.0083	-0.00946	0.00181
rs13016665	C	-0.00849	0.001412	-0.0108	0.008	0.00124	0.00172
rs13153393	A	-0.01375	0.002173	-0.0148	0.0134	0.00549	0.00268
rs13296519	G	-0.0097	0.001419	0.0084	0.0086	0.00775	0.00174
rs136233	A	-0.00996	0.001769	-9.00E-04	0.0104	0.00581	0.00217
rs147412694	G	-0.01157	0.001949	-0.0203	0.0127	-0.00077	0.00243
rs17309874	G	-0.01129	0.001582	-0.009	0.0102	0.00825	0.00194
rs17553262	A	-0.01273	0.002181	0.0083	0.0132	0.01119	0.00272
rs17576594	G	0.01095	0.001552	0.0043	0.0087	-0.01126	0.00189
rs1922018	C	0.010033	0.001438	-0.0109	0.0084	-0.00567	0.00176
rs1931263	G	-0.00761	0.001386	0.0091	0.0085	0.01204	0.0017
rs1933270	T	0.009218	0.001438	1.00E-04	0.0081	-0.00164	0.00176
rs202645	A	-0.01016	0.001725	8.00E-04	0.0108	-9.00E-05	0.00214
rs2062882	G	-0.00811	0.00142	0.013	0.0087	0.008	0.00173
rs2080870	A	0.008633	0.001583	0.0061	0.0099	-0.01576	0.00195

rs2254710	C	0.008996	0.001631	0.015	0.0091	-0.00306	0.00198
rs2401924	G	0.010572	0.001389	0.0077	0.0079	0.00441	0.0017
rs245774	A	-0.00902	0.00156	-0.0169	0.0093	0.00702	0.00192
rs2675638	G	0.008499	0.0014	0.0182	0.008	-0.0068	0.00172
rs2678670	A	0.008731	0.001388	0.01	0.0088	-0.00987	0.0017
rs2838834	C	-0.00936	0.001515	0.005	0.0086	-0.00091	0.00186
rs28485305	C	0.008007	0.001439	0.0155	0.008	-0.00075	0.00176
rs2867112	T	0.014783	0.001887	0.0031	0.0108	-0.00484	0.00229
rs2890772	G	-0.0137	0.001407	-0.004	0.0081	-0.00355	0.00173
rs2894808	T	-0.0153	0.002591	0.0112	0.0148	0.00761	0.00314
rs317021	T	-0.01157	0.001791	0.0075	0.0121	0.00569	0.0022
rs326341	G	0.009435	0.001392	0.0076	0.0078	-0.00627	0.0017
rs329120	C	0.009657	0.001405	0.0054	0.0078	-0.00833	0.00172
rs34866095	A	-0.00857	0.001506	-0.0153	0.0092	0.0019	0.00184
rs348809	A	-0.00828	0.001456	0.0062	0.0084	0.00498	0.00179
rs35169606	T	0.008776	0.001444	0.0102	0.0096	-0.00248	0.00177
rs35175834	G	-0.0164	0.001698	0.006	0.0103	0.01292	0.00208
rs35343344	C	0.009181	0.001596	0.0219	0.0095	-0.00588	0.00196
rs359243	T	-0.00872	0.001425	0.0015	0.0087	0.00644	0.00174
rs369230	G	-0.00909	0.001511	-0.0073	0.0093	0.0052	0.00193
rs3742365	T	-0.01079	0.001416	4.00E-04	0.008	-0.0059	0.00173
rs3769949	T	-0.00826	0.001386	-0.0131	0.008	0.00495	0.0017
rs3811038	T	-0.00954	0.001557	0.0065	0.0091	0.0066	0.0019
rs3896224	A	0.009627	0.001418	-0.0038	0.008	-0.0088	0.00173
rs421983	T	0.008707	0.001386	0.0063	0.0079	-0.00333	0.0017
rs4391802	A	0.010319	0.001528	0.0073	0.0087	-0.00716	0.00187
rs4473348	A	-0.01043	0.001597	-0.0096	0.0091	-0.00261	0.00197
rs4543592	T	-0.00866	0.001389	-3.00E-04	0.0082	0.00666	0.0017
rs4571506	C	0.007877	0.001392	0.0165	0.0079	-0.01154	0.00171
rs4671357	T	-0.00944	0.00139	-0.0041	0.0079	0.00913	0.0017
rs4731925	C	-0.00829	0.00149	0.0051	0.0085	0.01057	0.00184
rs4814873	C	0.009712	0.001636	0.0105	0.0093	-0.00643	0.00203
rs4949465	T	-0.01161	0.002058	0.0086	0.0109	0.01288	0.0025
rs4957528	A	-0.01012	0.001722	-0.0022	0.0104	0.00507	0.00212
rs549845	G	0.011261	0.001509	0.0124	0.0084	-0.01772	0.00186
rs57611503	G	0.007743	0.00141	-0.0044	0.0089	-0.0033	0.00172
rs6011779	C	0.019115	0.001764	0	0.0111	-0.00422	0.00223
rs60952428	T	0.013411	0.002419	-0.0149	0.012	-0.00787	0.00306
rs6119897	G	-0.0128	0.001627	0.0103	0.0092	0.00691	0.00196
rs61796681	A	-0.01342	0.002448	-0.0181	0.0159	0.00053	0.00301
rs62098013	G	-0.00857	0.001457	-0.0089	0.0092	0.01256	0.00176
rs62135536	C	0.024344	0.003962	-0.0021	0.0283	-0.00924	0.00487
rs62155874	A	-0.01691	0.002085	0.002	0.013	0.01416	0.00258
rs62175972	T	0.021746	0.003857	0.0276	0.028	-0.02995	0.00475
rs624833	T	0.009286	0.001504	0.0029	0.0093	-0.01316	0.00184
rs6562474	C	0.008369	0.001461	0.0074	0.0081	-0.00427	0.00178

rs6598539	T	-0.00815	0.001389	-0.0046	0.008	0.00651	0.00171
rs6741228	T	0.007933	0.001404	0.0106	0.008	-0.00747	0.00172
rs67596067	G	-0.00888	0.001458	-0.0035	0.0086	0.00355	0.00178
rs6778080	T	0.011114	0.001566	-0.0119	0.0102	-0.01385	0.00192
rs6779302	G	-0.00875	0.001439	0.0035	0.0088	0.01129	0.00176
rs6935954	A	0.009582	0.001402	-0.0043	0.0088	-0.01376	0.00172
rs6957896	C	-0.00758	0.001387	-0.0075	0.0084	0.00164	0.0017
rs6962772	A	0.011064	0.001916	0.0117	0.0104	-0.00404	0.00234
rs7039819	G	0.008734	0.001405	0.0091	0.0086	-0.01059	0.00172
rs7077678	C	0.00855	0.001436	-0.0048	0.008	0.00163	0.00174
rs71367545	G	-0.01032	0.001704	-0.009	0.0113	0.01246	0.0021
rs7155595	A	-0.00885	0.001485	-0.0117	0.0093	0.00734	0.00183
rs71627581	G	0.013257	0.002199	-0.0037	0.0167	-0.00962	0.00281
rs72674867	A	0.008988	0.001634	0.0019	0.0088	-0.00674	0.00197
rs72678864	G	0.012383	0.001839	-0.0055	0.0127	-0.01227	0.0023
rs7297175	T	-0.00812	0.001399	-0.0186	0.0083	0.01559	0.00173
rs732083	G	0.008348	0.001473	0.0131	0.0083	-0.00814	0.0018
rs73220544	A	-0.01082	0.001913	-0.009	0.0113	0.00692	0.00237
rs7333559	G	0.01074	0.001707	0.0014	0.0097	-0.00597	0.00212
rs74086911	G	0.014802	0.002642	0.0027	0.0168	-0.01837	0.0034
rs7519626	C	0.00842	0.001479	0.0112	0.0083	-0.00688	0.0018
rs7528604	G	0.009653	0.001401	0.0202	0.0079	-0.00594	0.00172
rs7553348	G	0.009634	0.001396	-0.0042	0.0081	-0.00042	0.00172
rs7569203	A	-0.01076	0.0015	0.0174	0.0085	0.00257	0.00181
rs75742406	G	0.009619	0.001585	-0.0236	0.0091	-0.00101	0.00192
rs775758	A	0.008014	0.001403	-0.0066	0.008	-0.00303	0.00171
rs7766610	C	0.012584	0.001793	-0.0033	0.0093	0.00078	0.00218
rs7807019	A	-0.01042	0.001391	-0.0091	0.0078	0.01055	0.0017
rs8042134	T	-0.00994	0.001401	0.0056	0.0082	0.00794	0.00173
rs8042849	C	0.019216	0.001462	0.0086	0.0086	0.00018	0.00178
rs860326	C	0.008338	0.001402	8.00E-04	0.0081	-0.00876	0.00172
rs8614	C	-0.01146	0.001797	-0.0133	0.0098	0.00407	0.00224
rs889398	C	0.009241	0.001414	0.0115	0.0083	-0.00182	0.00172
rs9435340	T	0.008348	0.001464	2.00E-04	0.0082	-0.00626	0.0018
rs9842947	C	-0.00877	0.001481	0.0015	0.0088	0.00091	0.00182
rs986391	G	0.011139	0.001438	0.0026	0.0085	0.00161	0.00177

Supplementary Table 19: Proxy SNPs for education instrument used in one-sample MR analysis

GWAS SNP (Okbay)	SNP in LD used (UKBB)
rs114598875	rs17538393
rs148734725	rs9878943
rs9320913	rs1487445
rs8005528	rs8008779
rs192818565	rs55943044

Supplementary Table 20: Genome-wide significant SNPs for SBP from split sample GWAS analysis in UK Biobank

Chromosome	RSID	Position	Beta	SE	Other Allele	P Value	Sample
1	rs5068	11905974	1.3283	0.1374	A	4.10E-22	1
1	rs448385	25395133	-0.3464	0.0630	G	3.80E-08	1
1	rs3790604	113046879	-0.8724	0.1204	C	4.30E-13	1
1	rs2765524	89417695	0.3965	0.0641	C	6.00E-10	1
2	rs953246	146335486	-0.3924	0.0685	T	1.00E-08	1
2	rs1344653	19730845	-0.3481	0.0626	A	2.70E-08	1
2	rs1009358	65276452	0.3693	0.0645	T	1.00E-08	1
2	rs268263	164954174	-0.5652	0.0735	T	1.50E-14	1
2	rs35021474	26916844	0.4639	0.0645	C	6.30E-13	1
2	rs2867114	651380	0.6149	0.1073	C	1.00E-08	1
3	rs3821843	53558012	-0.4129	0.0681	G	1.40E-09	1
3	rs1343040	169186293	-0.4317	0.0637	G	1.20E-11	1
3	rs2643826	27562988	-0.3780	0.0631	C	2.20E-09	1
3	rs263016	183502559	0.3475	0.0631	T	3.60E-08	1
4	rs10857147	81181072	-0.8157	0.0690	A	3.00E-32	1
4	rs6825268	26783453	-0.3573	0.0631	A	1.50E-08	1
4	rs13107325	103188709	0.7689	0.1190	C	1.00E-10	1
4	rs1842896	156511459	-0.4014	0.0626	G	1.40E-10	1
5	rs13436194	157803588	0.4570	0.0632	A	4.80E-13	1
5	rs12656497	32831939	-0.7161	0.0638	T	3.00E-29	1
6	rs2607015	31762843	-0.4232	0.0636	G	2.90E-11	1
6	rs2499801	96854594	0.4442	0.0806	G	3.60E-08	1
6	rs13219548	127165290	-0.4051	0.0630	C	1.30E-10	1
7	rs62481856	106412082	-0.8444	0.0789	G	9.30E-27	1
7	rs2854747	45959917	0.4198	0.0637	G	4.40E-11	1
7	rs10241964	19042114	-0.5983	0.1056	G	1.40E-08	1
7	rs10269774	92253972	0.3854	0.0669	G	8.40E-09	1
7	rs891511	150704843	0.3765	0.0681	G	3.20E-08	1
7	rs2023843	27243221	-0.8627	0.1198	C	6.00E-13	1
7	rs3823483	131010943	-0.3546	0.0634	T	2.30E-08	1
8	rs877116	10712945	0.4697	0.0636	G	1.50E-13	1
8	rs7463212	143991858	0.3776	0.0629	T	2.00E-09	1
8	rs73563812	25900405	0.4215	0.0737	G	1.10E-08	1
10	rs56137952	134376691	-0.5494	0.0985	G	2.40E-08	1
10	rs10883543	102552752	-0.6995	0.0996	G	2.20E-12	1
10	rs10995311	64564934	0.3941	0.0634	C	5.00E-10	1
10	rs11191580	104906211	1.0970	0.1180	T	1.50E-20	1
10	rs7076938	115789375	-0.4606	0.0711	C	9.30E-11	1
10	rs12258967	18727959	0.6582	0.0686	C	8.00E-22	1
10	rs7922049	63462365	0.5533	0.0869	G	1.90E-10	1
10	rs10786156	96014622	0.3767	0.0632	C	2.50E-09	1
11	rs55925664	10192809	-0.6415	0.0805	T	1.60E-15	1

11	rs7120737	47702395	0.6316	0.0891	A	1.30E-12	1
11	rs10750766	65473798	-0.3930	0.0691	C	1.30E-08	1
11	rs633185	100593538	-0.6945	0.0698	G	2.60E-23	1
11	rs12418543	1894163	0.5668	0.0645	A	1.50E-18	1
11	rs747249	130271647	0.3747	0.0656	A	1.10E-08	1
12	rs73437338	90054619	0.7678	0.0843	T	8.80E-20	1
12	rs4766578	111904371	0.4066	0.0626	T	8.60E-11	1
12	rs35444	115552437	0.3909	0.0643	A	1.20E-09	1
12	rs73073676	20351276	0.3773	0.0670	A	1.80E-08	1
15	rs8039305	91422543	-0.5943	0.0630	T	4.00E-21	1
15	rs1717200	41368334	-0.3912	0.0628	A	4.70E-10	1
15	rs1543927	75063573	0.4218	0.0712	T	3.10E-09	1
15	rs11634851	81028965	-0.4216	0.0628	C	1.90E-11	1
16	rs77870048	69965021	-0.9117	0.1399	C	7.20E-11	1
16	rs2188717	24730230	-0.5361	0.0792	T	1.30E-11	1
17	rs9907379	59489893	-0.4257	0.0769	T	3.20E-08	1
17	rs60289499	43218677	-0.4564	0.0708	G	1.10E-10	1
17	rs34710835	45146717	0.5223	0.0643	C	4.40E-16	1
17	rs11650511	1337960	-0.4085	0.0634	C	1.20E-10	1
19	rs73046792	49605705	0.4714	0.0843	G	2.30E-08	1
19	rs12978472	7257990	0.8468	0.0941	C	2.20E-19	1
20	rs74729242	57718690	-0.5901	0.1013	T	5.80E-09	1
20	rs2423514	10693337	0.3709	0.0628	A	3.50E-09	1
1	rs55857306	11895795	0.7687	0.0850	G	1.60E-19	2
1	rs4648815	1687152	0.3807	0.0638	G	2.40E-09	2
1	rs6541328	230833262	-0.5551	0.1010	A	3.80E-08	2
1	rs778121	56620268	-0.3887	0.0659	T	3.60E-09	2
1	rs6657049	115825531	-0.3665	0.0658	G	2.50E-08	2
2	rs268263	164954174	-0.5602	0.0738	T	3.20E-14	2
2	rs4666493	19765225	-0.3641	0.0639	G	1.20E-08	2
2	rs1275988	26914364	0.4835	0.0650	C	9.90E-14	2
2	rs6724607	191466532	0.3656	0.0630	A	6.40E-09	2
3	rs2307032	27432995	0.3879	0.0664	T	5.20E-09	2
3	rs6442260	11590751	0.3595	0.0659	G	4.90E-08	2
4	rs10024506	89764197	0.4173	0.0745	G	2.10E-08	2
4	rs11099097	81167309	-0.6371	0.0696	C	5.40E-20	2
4	rs4690974	156393641	-0.3741	0.0631	T	3.10E-09	2
4	rs17010961	86723103	-0.5775	0.0913	T	2.50E-10	2
5	rs10059884	32832474	-0.5859	0.0643	C	7.80E-20	2
5	rs12652819	121244520	0.3775	0.0677	A	2.50E-08	2
5	rs17715065	158261163	-0.3662	0.0631	C	6.60E-09	2
5	rs11241959	127787964	-0.3715	0.0631	A	4.00E-09	2
5	rs2964330	157743781	-0.3645	0.0641	G	1.30E-08	2
6	rs17080069	150989698	0.7199	0.1218	A	3.40E-09	2
6	rs6923947	127098553	-0.4974	0.0635	G	5.00E-15	2
6	rs7889	31605448	-0.3792	0.0657	C	7.80E-09	2

7	rs891511	150704843	0.4141	0.0683	G	1.30E-09	2
7	rs2392929	106414069	-0.7165	0.0790	T	1.20E-19	2
7	rs42032	92237426	0.3967	0.0720	G	3.60E-08	2
7	rs57301765	19052733	-0.5059	0.0866	G	5.20E-09	2
9	rs2780072	9340831	-0.5035	0.0902	A	2.40E-08	2
10	rs76443711	75449789	-0.5442	0.0915	G	2.70E-09	2
10	rs7070797	63551773	0.6290	0.0905	G	3.60E-12	2
10	rs11187838	96038686	0.5615	0.0637	G	1.20E-18	2
10	rs732998	104897901	0.8365	0.1184	T	1.60E-12	2
10	rs12258967	18727959	0.5893	0.0691	C	1.50E-17	2
11	rs4980379	1888614	-0.5896	0.0657	C	2.70E-19	2
11	rs12807950	107057190	-0.3790	0.0631	T	1.90E-09	2
11	rs7107356	47676170	-0.4793	0.0630	A	2.90E-14	2
11	rs1216743	100573120	-0.5662	0.0705	G	9.60E-16	2
12	rs2681492	90013089	0.6765	0.0840	T	8.00E-16	2
12	rs4767328	115929396	-0.3575	0.0640	G	2.30E-08	2
12	rs35427	115556307	0.4110	0.0664	T	6.00E-10	2
12	rs4883481	50574311	0.4218	0.0652	T	1.00E-10	2
12	rs597808	111973358	0.4310	0.0634	A	1.00E-11	2
15	rs7176022	75107880	0.4365	0.0713	A	9.10E-10	2
15	rs4932373	91429287	-0.5144	0.0672	A	2.00E-14	2
15	rs117539635	69682916	1.3387	0.2009	A	2.70E-11	2
16	rs77870048	69965021	-0.9216	0.1415	C	7.40E-11	2
16	rs11646987	24832408	0.3922	0.0709	G	3.20E-08	2
17	rs1436138	75316880	0.3615	0.0661	A	4.40E-08	2
17	rs7217916	76769434	0.3944	0.0650	A	1.30E-09	2
17	rs2301597	43173273	0.5081	0.0640	T	2.00E-15	2
17	rs11874	45017193	-0.5820	0.0917	G	2.20E-10	2
17	rs4480845	1958609	0.4024	0.0662	T	1.20E-09	2
19	rs167479	11526765	0.5743	0.0630	G	8.20E-20	2
20	rs75777337	57702450	-0.6125	0.1033	T	3.10E-09	2
20	rs913220	10966476	-0.4389	0.0649	C	1.40E-11	2

Supplementary Table 21: Genome-wide significant SNPs for lifetime smoking from split sample GWAS analysis in UK Biobank

Chromosome	RSID	Position	Beta	SE	Other Allele	P Value	Sample
1	rs71673396	107507403	.0159243	.0029062	T	4.30e-08	1
1	rs499257	44078384	.0146849	.0024706	T	2.80e-09	1
2	rs2890772	146175106	-.0140201	.0023608	G	2.90e-09	1
3	rs4856463	83638568	.0156642	.0028286	C	3.10e-08	1
3	rs326341	107811142	.013281	.0023333	G	1.30e-08	1
4	rs6852351	28064697	.0132797	.00241	C	3.60e-08	1
5	rs17159727	106632458	.0241372	.0040898	T	3.60e-09	1
5	rs986391	166993972	.0151657	.0024028	G	2.80e-10	1
6	rs16879271	16822974	-.0325007	.0059395	A	4.50e-08	1
7	rs10226228	32315613	-.0141999	.0024083	A	3.70e-09	1
7	rs10233018	117523709	-.0129883	.0023226	A	2.20e-08	1
8	rs10093628	9393379	-.0165694	.0027221	T	1.20e-09	1
9	rs113382419	136463019	-.0242553	.0036827	C	4.50e-11	1
11	rs10750016	112837740	-.0160683	.0023918	T	1.80e-11	1
11	rs11030088	27646247	-.0157839	.002668	G	3.30e-09	1
11	rs6590701	133315869	-.0144894	.0026473	G	4.40e-08	1
12	rs4763463	10355901	.0132272	.0023863	G	3.00e-08	1
15	rs7173514	78849918	.0224245	.0027791	C	7.10e-16	1
1	rs10922907	91193049	.0134411	.0023251	A	7.40e-09	2
2	rs1863161	60139524	-.0127982	.0023263	G	3.80e-08	2
2	rs16824949	146168208	-.0145352	.0023181	G	3.60e-10	2
2	rs7559547	615627	-.0219576	.0030462	C	5.70e-13	2
2	rs263771	185921692	-.0151079	.0027443	C	3.70e-08	2
3	rs62261249	49594060	-.0158442	.0026313	T	1.70e-09	2
7	rs17657924	96625589	.0132644	.0023312	C	1.30e-08	2
9	rs12553882	128195044	-.014292	.0024004	G	2.60e-09	2
9	rs56116178	136460224	-.0306714	.0038063	A	7.70e-16	2
11	rs7948789	112839532	-.0164578	.0023768	A	4.40e-12	2
14	rs12897150	104319530	-.0136766	.0023459	A	5.50e-09	2
15	rs28669908	78910267	.0237801	.0028216	C	3.50e-17	2
15	rs34794623	47680801	-.0195197	.0028221	C	4.60e-12	2
20	rs45577732	61983934	-.0386369	.0042941	C	2.30e-19	2
20	rs159058	31108108	-.0142308	.0025321	A	1.90e-08	2

Supplementary Table 22: Unadjusted estimates for the proportion mediated in individual level UK Biobank analyses estimated from the log OR scale

Outcome	Method	Mediator	Proportion Mediated (%) (95% CI)
CVD	Multivariable observational	BMI	10.93 (9.52, 12.34)
		SBP	19.18 (16.96, 21.40)
		Smoking	12.66 (10.80, 14.52)
	One-sample MR	BMI	9.47 (-8.18, 21.12)
		SBP	4.62 (-9.63, 18.88)
		Smoking	20.96 (-23.20, 65.12)
Stroke	Multivariable observational	BMI	5.76 (3.36, 8.16)
		SBP	21.68 (15.32, 28.05)
		Smoking	10.23 (6.71, 13.75)
	One-sample MR	BMI	13.58 (-251.31, 278.47)
		SBP	-4.88 (-66.78, 57.00)
		Smoking	24.00 (-2634.32, 2682.32)
MI	Multivariable observational	BMI	9.15 (6.03, 12.28)
		SBP	24.56 (17.42, 31.70)
		Smoking	18.35 (13.10, 23.59)
	One-sample MR	BMI	-4.93 (-18.47, 8.61)
		SBP	16.07 (-21.86, 54.00)
		Smoking	20.11 (-4.06, 44.27)
CHD	Multivariable observational	BMI	10.64 (8.66, 12.62)
		SBP	23.68 (20.42, 26.95)
		Smoking	13.88 (11.60, 16.19)
	One-sample MR	BMI	-4.96 (-16.75, 6.84)
		SBP	13.76 (-15.49, 43.01)
		Smoking	11.75 (-19.50, 43.00)

Supplementary Table 23: Age and sex adjusted estimates for mediation by BMI, SBP and smoking on the association of education to CVD in individual level UK Biobank analyses

Outcome	Method	Mediator	Proportion Mediated (%)
**CVD**	Multivariable observational	BMI	16.75 (13.73, 19.77)
		SBP	8.10 (6.50, 9.69)
		Smoking	19.36 (15.67, 23.05)
	One-sample MR	BMI	9.18 (-5.97, 24.34)
		SBP	5.95 (-10.04, 21.93)
		Smoking	23.26 (-6.26, 52.78)
**Stroke**	Multivariable observational	BMI	10.59 (3.05, 18.13)
		SBP	14.47 (5.77, 23.17)
		Smoking	19.83 (9.15, 30.51)
	One-sample MR	BMI	12.43 (-233324.91, 23349.77)
		SBP	-4.30 (-62.25, 53.66)
		Smoking	25.25 (-145.56, 196.07)
**MI**	Multivariable observational	BMI	9.85 (5.88, 13.83)
		SBP	10.78 (6.98, 14.58)
		Smoking	19.24 (12.99, 25.84)
	One-sample MR	BMI	-4.64 (-18.07, 8.79)
		SBP	20.23 (-26.15, 66.61)
		Smoking	18.95 (-9.84, 47.73)
**CHD**	Multivariable observational	BMI	15.37 (11.55, 19.18)
		SBP	11.26 (8.92, 13.60)
		Smoking	18.50 (14.45, 22.56)
	One-sample MR	BMI	-4.24 (-15.23, 6.74)
		SBP	16.97 (-17.84, 51.79)
		Smoking	11.75 (-19.50, 43.00)
